# Interventional‐ and amputation‐stage muscle proteomes in the chronically threatened ischemic limb

**DOI:** 10.1002/ctm2.658

**Published:** 2022-01-24

**Authors:** Terence E. Ryan, Kyoungrae Kim, Salvatore T. Scali, Scott A. Berceli, Trace Thome, Zachary R. Salyers, Kerri A. O'Malley, Thomas D. Green, Reema Karnekar, Kelsey H. Fisher‐Wellman, Dean J. Yamaguchi, Joseph M. McClung

**Affiliations:** ^1^ Department of Applied Physiology and Kinesiology University of Florida Gainesville Florida USA; ^2^ Center for Exercise Science University of Florida Gainesville Florida USA; ^3^ Myology Institute University of Florida Gainesville Florida USA; ^4^ Division of Vascular Surgery and Endovascular Therapy University of Florida Gainesville Florida USA; ^5^ Malcom Randall Veteran Affairs Medical Center Gainesville Florida USA; ^6^ Department of Physiology Brody School of Medicine East Carolina University Greenville North Carolina USA; ^7^ East Carolina Diabetes and Obesity Institute East Carolina University Greenville North Carolina USA; ^8^ Department of Cardiovascular Science East Carolina University Greenville North Carolina USA; ^9^ Division of Surgery East Carolina University Greenville North Carolina USA

**Keywords:** metabolism, peripheral artery disease, surgery, vascular disease

## Abstract

**Background:**

Despite improved surgical approaches for chronic limb‐threatening ischemia (CLTI), amputation rates remain high and contributing tissue‐level factors remain unknown. The purpose of this study was twofold: (1) to identify differences between the healthy adult and CLTI limb muscle proteome, and (2) to identify differences in the limb muscle proteome of CLTI patients prior to surgical intervention or at the time of amputation.

**Methods and results:**

Gastrocnemius muscle was collected from non‐ischemic controls (n = 19) and either pre‐interventional surgery (n = 10) or at amputation outcome (n = 29) CLTI patients. All samples were subjected to isobaric tandem‐mass‐tag‐assisted proteomics. The mitochondrion was the primary classification of downregulated proteins (> 70%) in CLTI limb muscles and paralleled robust functional mitochondrial impairment. Upregulated proteins (> 38%) were largely from the extracellular matrix. Across the two independent sites, 39 proteins were downregulated and 12 upregulated uniformly. Pre‐interventional CLTI muscles revealed a robust upregulation of mitochondrial proteins but modest functional impairments in fatty acid oxidation as compared with controls. Comparison of pre‐intervention and amputation CLTI limb muscles revealed mitochondrial proteome and functional deficits similar to that between amputation and non‐ischemic controls. Interestingly, these observed changes occurred despite 62% of the amputation CLTI patients having undergone a prior surgical intervention.

**Conclusions:**

The CLTI proteome supports failing mitochondria as a phenotype that is unique to amputation outcomes. The signature of pre‐intervention CLTI muscle reveals stable mitochondrial protein abundance that is insufficient to uniformly prevent functional impairments. Taken together, these findings support the need for future longitudinal investigations aimed to determine whether mitochondrial failure is causally involved in amputation outcomes from CLTI.

## Graphical abstract

Proteomic analyses of CLTI muscle specimens identify a distinct limb muscle proteome signature characterized by a severe mitochondriopathy in amputation‐stage specimens, which was discovered to differ from non‐PAD controls and CLTI pre‐surgical intervention limb muscles. Coincident with the proteome signature, mitochondrial function was severely impaired in amputation‐stage CLTI specimens. Compared to non‐PAD controls, interventional‐stage CLTI specimens displayed an upregulation of the mitochondrial proteome; however, this did not afford any improvement in mitochondrial oxidative phosphorylation.

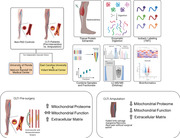

**Abbreviations**
ABI: ankle‐brachial index
CLTI: chronic limb threatening ischemia
MALE: major adverse limb event
OXPHOS: oxidative phosphorylation
PAD: peripheral artery disease

## 1 INTRODUCTION

The most severe manifestation of peripheral artery disease (PAD) is chronic limb‐threatening ischemia (CLTI). While this occurs in only 5–10% of patients, it carries substantially greater risk for major limb amputation, cardiovascular events, and mortality.^1^, ^2^ CLTI patients often present with more complex patterns of disease and although endovascular recanalization or revascularization (bypass) surgical procedures have improved technically, resolution of symptoms occurs in only 25% of CLTI patients.^3^ In fact, major adverse limb events remain unacceptably high,^4^, ^5^ highlighting the dire need to better understand CLTI pathobiology. Recent work using wide‐ranging “omics” and screening technologies have revealed tissue characteristics unique to the CLTI presentation (compared to non‐PAD or mild claudicants), including the transcriptome,^6^, ^7^, ^8^ cytokine profile,^9^ metabolome,^10^, ^11^ mitochondrial function,^6^, ^12^, ^13^ and lipid profiles^11^ of the limb muscle. Each of these studies has provided new insight into the biological underpinnings of the affected CLTI limb, a necessity to develop new ideas to improve lower limb tissue level perfusion and/or alleviate the severe myopathy that persists in these patients. The proteome of the CLTI peripheral limb muscle has not been described but is a likely functional outcome to the previously described changes. Given that mRNA changes do not necessarily result in differences in abundance, analysis of the CLTI proteome would provide a detailed biological portrait to drive functional therapeutic target design for CLTI. The purpose of this study was twofold: (1) to identify differences between the non‐ischemic control and CLTI limb muscle proteome, and (2) to identify differences in the limb muscle proteome of CLTI patients prior to surgical intervention and at the time of amputation. Non‐PAD control and CLTI patients were recruited across two independent vascular surgery practices and limb muscle tissues collected for isobaric tandem‐mass‐tag (TMT) assisted quantitative proteomics.

**HIGHLIGHTS**

- Proteomic analysis of CLTI muscle specimens identifies a distinct limb muscle proteome signature characterized by a severe mitochondriopathy in amputation‐stage specimens, which was discovered to differ from non‐PAD controls and CLTI presurgical intervention limb muscles. Coincident with the proteome signature, mitochondrial function was severely impaired in amputation‐stage CLTI specimens.
- Compared to non‐PAD controls, interventional‐stage CLTI specimens displayed an upregulation of the mitochondrial proteome; however this did not afford any improvement in mitochondrial oxidative phosphorylation.
- Regardless of CLTI treatment stage, the largest class of upregulated proteins were related to the extracellular matrix.

## 2 MATERIALS AND METHODS

### 2.1 Study populations and specimen collection

Gastrocnemius muscle specimens were collected from 26 older adult non‐PAD controls (Con) and 39 CLTI patients across two vascular clinics (University of Florida and East Carolina University). Five presurgery patients underwent bypass interventions and five underwent endovascular procedures. Muscle specimens were collected within the confines of the operating rooms (CLTI patients) or via percutaneous muscle biopsy using sterile procedures previously described.^6^, ^14^ A portion of the muscle was quickly trimmed of fat/connective tissue and snap‐frozen in liquid nitrogen for proteomic analysis. Another portion of the fresh muscle specimen was processed immediately for mitochondrial function assessment as described in detail previously.^6^ Non‐PAD control participants enrolled either had an ankle‐brachial index (ABI) greater than or equal to 1.0 (University of Florida) or based on diagnosis‐ and symptom‐free by self‐report (East Carolina University). This study was approved by the institutional review boards at the University of Florida, Malcom Randall VA Medical Center, and East Carolina University. All study procedures were carried out according to the Declaration of Helsinki and participants were fully informed about the research and informed consent was obtained.

### 2.2 TMT‐labeled proteomics analysis of skeletal muscle

University of Florida: Frozen muscle samples (∼10 mg) from each population (n = 10 control, n = 10 CLTI presurgery, and n = 10 CLTI amputation) were homogenized in ice‐cold CHAPS lysis buffer (150 mM KCl, 50 mM HEPES, 0.1% CHAPS, 1× cOmplete ULTRA mini EDTA‐free protease inhibitor tablet, pH 7.4) using a glass Teflon homogenizer (Wheaton). To release protein bound to DNA, the lysates were further processed with syringe lysis (lysate passaging 10 times through a 23‐gauge needle attached to a 1 ml syringe). Following centrifugation (16,000 × *g*) for 10 min at 4°C, the supernatant was collected and the protein concentration of each sample was determined using BCA protein assay kit (ThermoFisher Scientific Cat. no. SL256970). To achieve reduction and alkylation of cysteine residues, 5 μl of 200 mM Bond‐Breaker TCEP solution (ThermoFisher Scientific cat. no.77720) was added to 200 μg protein sample and incubated at 55°C for 1 h. This step was followed by addition of 5 μl of 375 mM iodoacetamide addition and incubation at room temperature for 30 min during which the samples were protected from light. The samples, thereafter, underwent chloroform/methanol/water precipitation and brief decanting (∼2 min) using a vacuum centrifuge. Following the precipitation, the pellets were re‐suspended with 100 μl of TEAB lysis buffer (ThermoFisher Scientific cat. no. 90114), 5 μl of trypsin was added, and incubated overnight at 37°C for protein digestion. Subsequently, the peptide samples were labeled using 11‐plex tandem mass tag (TMT)‐based isobaric stable isotope label reagents following the manufacturer's instructions (ThermoFisher Scientific cat. no. A34808). In brief, each TMT tag vial was dissolved with 41 μl of anhydrous acetonitrile (ThermoFisher Scientific ca. no. 51101) and the TMT label reagent was added to each peptide sample followed by 1 h incubation protected from light at room temperature with gentle shaking. After quenching (∼15 min) the reaction with 8 μl of 5% hydroxylamine (ThermoFisher Scientific ca. no. 90115), labeled samples were submitted to the UF ICBR Proteomics Core facility where the samples were combined and purified using C18 spin columns for mass spectroscopy quantification. Each 11‐plex TMT kit involved analysis of a pooled control sample that was used to account for batch variability and normalization across runs.

East Carolina University: Frozen muscle samples (∼10 mg) from each population (n = 16 control and n = 19 amputation) were combined into 9 grouped Control (7 samples contained 2 patients each) and 11 grouped amputation aliquots (8 samples contained 2 patients each) and lysed in ice‐cold 8 M Urea Lysis Buffer (8 M urea in 40 mM Tris, pH 8.0, 30 mM NaCl, 1 mM CaCl2, 1x cOmplete ULTRA mini EDTA‐free protease inhibitor tablet), as described previously.^15^ Pooling muscle specimens were performed to maximize patient specimen use across the available resources. Each individual TMT 10‐plex kit involved analysis of a pooled control sample that was used to account for batch variability and normalization across runs. Homogenates were subjected to three freeze‐thaw cycles and further disrupted by sonication with a probe sonicator in three 5 second bursts (Q Sonica #CL‐188; amplitude of 30). Centrifugation was performed at 10,000 × *g* for 10 min at 4°C to pellet insoluble material. Protein concentration was determined by BCA, and equal amounts of protein (100 μg) were subjected to reduction and alkylation (5 mM DTT at 32°C for 30 min, 15 mM iodoacetamide for 30 min in the dark). Unreacted iodoacetamide was quenched by the addition of DTT up to 15 mM. Digestion was first performed with Lys C (ThermoFisher Scientific, cat. no. 90307; 1:100 w:w; 1 μg enzyme per 100 μg protein) for 4 h at 32°C. Following dilution to 1.5 M urea with 40 mM Tris (pH 8.0), 30 mM NaCl, 1 mM CaCl_2_, the samples were trypsin digested overnight (Promega; cat. no. V5113; 50:1 w/w, protein:enzyme) at 32°C. Samples were acidified to 0.5% TFA and centrifuged at 4000 × *g* for 10 min at 4°C. Supernatant containing soluble peptides was desalted on a 50 mg tC18 SEP‐PAK solid‐phase extraction column (Waters, cat. no. WAT054955) and eluted (500 μL 25% acetonitrile/0.1% TFA and 2 × 500 μL 50% acetonitrile/0.1% TFA). Eluate was frozen and lyophilized. TMT labeling was performed as previously described.^15^ The 20 samples from skeletal muscle were re‐suspended in 100 μL of 200 mM triethylammonium bicarbonate (TEAB), mixed with a unique 10‐plex Tandem Mass Tag (TMT10) reagent (0.8 mg re‐suspended in 50 μL100% acetonitrile) from 2× TMT10 kits, and shaken for 4 h at room temperature (ThermoFisher Scientific). Following quenching with 0.8 μL 50% hydroxylamine, all samples from each kit were combined into respective tubes, frozen, and lyophilized. Multiplexed samples were re‐suspended in ∼1 mL of 0.5% TFA and again subjected to solid‐phase extraction, but with a 100 mg tC18 SEP‐PAK SPE column (Waters, cat. no. WAT023590). The multiplexed peptide samples were subjected to high pH reversed‐phase fractionation according to the manufacturer's instructions (ThermoFisher Scientific, cat. no. 84868). Following elution, fractions (8 total fractions per kit) were frozen and lyophilized.

### 2.3 LC‐MS/MS for TMT proteomics

University of Florida: Labeled peptides were desalted with C18‐solid‐phase extraction and dissolved in strong cation exchange (SCX) solvent A (25% (v/v) acetonitrile, 10 mM ammonium formate, and 0.1% (v/v) formic acid, pH 2.8). The peptides were fractionated using an Agilent HPLC 1260 with a polysulfoethyl A column (2.1 × 100 mm, 5 μm, 300 Å; PolyLC, Columbia, MD, USA). Peptides were eluted with a linear gradient of 0–20% solvent B (25% (v/v) acetonitrile and 500 mM ammonium formate, pH 6.8) over 50 min followed by ramping up to 100% solvent B in 5 min. The absorbance at 280 nm was monitored and a total of 16 fractions were collected. The fractions were lyophilized, desalted, and resuspended in LC solvent A (0.1% formic acid in 99.9% water (v/v)). A hybrid quadrupole Orbitrap (Q Exactive Plus) MS system (Thermo Fisher Scientific, Bremen, Germany) was used with high energy collision dissociation (HCD) in each MS and MS/MS cycle. The MS system was interfaced with an automated Easy‐nLC 1000 system (Thermo Fisher Scientific, Bremen, Germany). Each sample fraction was loaded onto an Acclaim Pepmap 100 pre‐column (20 mm  ×  75 μm; 3 μm‐C18) and separated on a PepMap RSLC analytical column (250 mm  ×  75 μm; 2 μm‐C18) at a flow rate at 350 nl/min during a linear gradient from solvent A (0.1% formic acid (v/v)) to 30% solvent B (0.1% formic acid (v/v) and 99.9% acetonitrile (v/v)) for 95 min, to 98% solvent B for 15 min, and hold 98% solvent B for additional 30 min. Full MS scans were acquired in the Orbitrap mass analyzer over *m/z* 400–2000 range with resolution 70,000 at 200 *m/z*. The top ten most intense peaks with charge state ≥ 3 were fragmented in the HCD collision cell normalized collision energy of 28%, (the isolation window was 0.7 *m/z*). The maximum ion injection times for the survey scan and the MS/MS scans were 250 ms, respectively and the ion target values were set to 3e6 and 1e6, respectively. Selected sequenced ions were dynamically excluded for 60 s.

East Carolina University: nLC‐MS/MS was performed as described previously.^15^ Peptide fractions were suspended in 0.1% formic acid (0.25 μg/μL) following peptide quantification (ThermoFisher Scientific). All samples were subjected to nanoLC‐MS/MS analysis using an UltiMate 3000 RSLCnano system (ThermoFisher Scientific) coupled to a Q Exactive PlusHybrid Quadrupole‐Orbitrap mass spectrometer (ThermoFisher Scientific) via nanoelectrospray ionization source. For each injection of 4 μL (1 μg), the sample was first trapped on an Acclaim PepMap 100 20 mm × 0.075 mm trapping column (ThermoFisher Scientific) 5 μl/min at 98/2 v/v water/acetonitrile with 0.1% formic acid, after which the analytical separation was performed over a 90‐min gradient (flow rate of 300 nanoliters/min) of 3–30% acetonitrile using a 2 μm EASY‐Spray PepMap RSLC C18 75 μm × 250 mm column (ThermoFisher Scientific). Column temperature was 55°C. MS1 was performed at 70,000 resolution, AGC target of 3 × 10^6^ ions and a maximum IT of 60 ms (scan range 300–1750 m/z). MS2 spectra were collected by data‐dependent acquisition (DDA) of the top 20 most abundant precursor ions with a charge greater than 1 per MS1 scan, with dynamic exclusion enabled for 45 s. Precursor ions filtered with a 0.7 *m/z* isolation window and fragmented with a normalized collision energy of 30. MS2 scans were performed at 35,000 resolution, AGC target of 1 × 10^5^ ions, and a maximum IT of 60 ms.

### 2.4 Data analysis for proteomics

University of Florida: The raw MS/MS data files were processed by a thorough database searching approach considering biological modification and amino acid substitution against Uniprot Homo sapiens [9606] database (downloaded on May 1, 2020; 188,558 entries) using the Proteome Discoverer v2.4 (Thermo Fisher Scientific), with the SEQUEST algorithm.^16^ The following parameters were used for all the searching: peptide tolerance at 10 ppm, tandem MS tolerance at ± 0.02 Da, peptide charges of 2+ to 5+, trypsin as the enzyme, allowing one missed cleavage, TMT label (229.163 Da on peptide N‐term and K) and carbamidomethyl (C) as fixed modifications, oxidation (15.995 Da on M) as a variable modification. The false discovery rate (FDR) was calculated using Percolator algorithm in the Proteome Discoverer workflow based on the search results against a decoy database and was set at 1% FDR. For protein quantification, only MS/MS spectra that were unique to a particular protein and where the sum of the signal‐to‐noise ratios for all the peak pairs > 9 were used for quantification. M2 reporter (TMT) intensities were summed together for each TMT channel, each channel's sum was divided by the average of all channels’ sums, resulting in channel‐specific loading control normalization factors to correct for any deviation from equal protein input in the 11‐plex experiments. Reporter intensities were divided by the loading control normalization factors for each respective TMT channel. All loading control‐normalized reporter intensities were converted to log_2_ space and the average value from the combined samples was subtracted from each sample specific measurement to normalize the relative measurements to the mean. For each comparison, protein abundances were analyzed for group average, standard deviation, two‐tailed Student's *t*‐test (equal variance), and a Benjamini‐Hochberg^17^ adjusted *P*‐value. All raw proteomics data are available online using accession numbers PXD021849 (Proteome Xchange^18^) or JPST000852 (jPOST Repository^19^). Gene ontology (GO) analyses were performed using PANTHER GO Enrichment analysis^20^, ^21^, ^22^ using *P* < 0.05.

East Carolina University: Proteome Discoverer 2.2 (PDv2.2) was used for raw data analysis, with default search parameters including oxidation (15.995 Da on M) as a variable modification and carbamidomethyl (57.021 Da on C) and TMT (229.163 Da on peptide N‐term and K) as fixed modifications, and two missed cleavages (full trypsin specificity). Data were searched against Uniprot Homo sapiens database. PSMs were filtered to a 1% FDR and then grouped into unique peptides while maintaining a 1% FDR. Peptides were grouped into proteins using the rules of strict parsimony and proteins were filtered to 1% FDR using the Protein FDR Validator node of PD2.2. MS2 reporter ion intensities for all PSMs having co‐isolation interference below 0.5 (50% of the ion current in the isolation window) and an average S/N > 10 for reporter ions were summed together at the peptide and protein level. Imputation was performed via low abundance resampling. The protein group tab in the PDv2.2 results was exported as tab delimited.txt. files, and analyzed as previously described.^15^ M2 reporter (TMT) intensities were summed together for each TMT channel, each channel's sum was divided by the average of all channels’ sums, resulting in channel‐specific loading control normalization factors to correct for any deviation from equal protein input in the 10‐plex experiments. Reporter intensities were divided by the loading control normalization factors for each respective TMT channel. All loading control‐normalized reporter intensities were converted to log_2_ space and the average value from the combined samples was subtracted from each sample‐specific measurement to normalize the relative measurements to the mean. For statistical comparison of Con to CTLI, condition average, standard deviation, *P*‐value (*P*, two‐tailed Student's *t*‐test, assuming equal variance), and adjusted *P*‐value (*P*_adjusted_, Benjamini Hochberg FDR correction) were calculated.^17^ For protein‐level quantification, only Master Proteins—or the most statistically significant protein representing a group of parsimonious proteins containing common peptides identified at 1% FDR—were used for quantitative comparison. All raw data are available online using accession number PXD025810 for Proteome Xchange^18^ and accession number JPST001157 for jPOST Repository.^19^ Gene ontology (GO) analyses were performed using PANTHER GO Enrichment analysis^20^, ^21^, ^22^ using P < 0.05.

### 2.5 Preparation of permeabilized muscle fibers

A portion of the muscle biopsy specimen was dissected and immediately placed in ice‐cold buffer X (50 mM K‐MES, 7.23 mM K_2_EGTA, 2.77 mM CaK_2_EGTA, 20 mM imidazole, 20 mM taurine, 5.7 mM ATP, 14.3 mM phosphocreatine, and 6.56 mM MgCl_2_‐6H_2_O, pH 7.1) for preparation of permeabilized fiber bundles.^6^, ^14^, ^23^ Fiber bundles were mechanically separated along their longitudinal axis using needle‐tipped forceps under a dissecting scope, and subsequently permeabilized with saponin (30 μg/ml) for 30 min at 4°C on a nutating mixer, and then washed in cold buffer Z (105 mM K‐MES, 30 mM KCl, 1 mM EGTA, 10 mM K_2_HPO_4_, 5 mM MgCl_2_‐6H_2_O, 0.5 mg/ml BSA, pH 7.1) for 15 min until analysis. Prior to loading fiber bundles for experimentation, they were gently blotted on a kim wipe for exactly 5 s and a wet weight was obtained using a Mettler Toledo MX5 microbalance.

### 2.6 Mitochondrial respiration measurements

High‐resolution O_2_ consumption measurements^6^, ^24^ were conducted at 37˚C in buffer Z (in mmol/l) (105 K‐MES, 30 KCl, 1 EGTA, 10 K_2_HPO_4_, 5 MgCl_2_6H_2_O, 0.5 mg/ml BSA, pH 7.1), supplemented with creatine monohydrate (5 mM), using the OROBOROS O2K Oxygraph. To assess mitochondrial function in physiologically relevant conditions, this study utilized a novel creatine‐kinase clamp system to set the level of cellular energy demand to which the fiber bundles were exposed.^25^, ^26^, ^27^, ^28^ First, bundles were energized with either carbohydrate (5 mM pyruvate and 2.5 mM malate) or fatty acid (0.2 mM octanoylcarnitine and 2.5 mM malate) and measurements of state 2 oxygen consumption were collected. Next, the creatine kinase clamp was added including 20 U/ml creatine kinase, 5 mM ATP, and 1 mM phosphocreatine (PCr) to mimic a near‐maximal exercise condition. Subsequent additions of PCr were added step‐wise to bring the cellular energy demand down to resting conditions. The slope of the relationship between cellular energy demand (∆G_ATP_) and oxygen consumption (JO_2_) was calculated. The rate of respiration was expressed as pmol/sec/mg fiber dry weight. All respiration measurements were conducted at 37°C and a working range [O_2_] of ∼350 to 200 μM.

### 2.7 Statistical analysis

This was cross‐sectional study in which participants were enrolled and analyses performed based on the available resources. Due to the exploratory nature, a priori sample size calculations were not performed. Data are presented as mean ± SD. Normality of all data was assessed using the Shapiro‐Wilk test. Data that were found to not be normally distributed were analyzed using a Kruskal‐Wallis test. Comparisons across the three groups were done using a one‐way ANOVA with Tukey's post hoc multiple comparisons when pairwise comparisons were appropriate. Chi‐Square analysis was used to determine differences in population proportions for relevant clinical characteristics. All statistical analysis was performed in GraphPad Prism (Version 8.0) or using Vassar Stats (http://vassarstats.net). For proteomics analysis, a Benjamini‐Hochberg^17^ adjusted *P* < 0.1 was considered significant with a false discovery rate (FDR) of 0.05. In all other cases, *P* < 0.05 was considered statistically significant.

## 3 RESULTS

### 3.1 Participant characteristics

In this study, limb muscle specimens were collected from non‐ischemic control participants and CLTI patients from two vascular clinics. All sample collection, protein digestion, and labeling, and mass spectrometry analysis were performed independently at each institution. Table 1 displays the physical and clinical characteristics of the 65 study participants across both sites. The physical characteristics for CLTI and non‐ischemic control groups were relatively well‐matched in age, although the control group had less comorbid conditions and less medication usage. Gastrocnemius muscle specimens from CLTI patients were harvested from the operating room immediately prior to a surgical intervention (n = 10, 50% endovascular and 50% revascularization) or amputation (n = 29). CLTI patient comorbidities across both sites included hypertension (90%), hyperlipidemia (82%), diabetes (69%), coronary artery disease (54%), chronic obstructive pulmonary disease (23%), and renal disease (33%). The majority (74%) of CLTI patients were either former or active smokers. Of the CLTI patients whose tissue was collected at the time of amputation, 62% had previously undergone a surgical intervention.

**TABLE 1:** Patient characteristics

	University of Florida				East Carolina University		
		CLTI				CLTI	
Characteristic	Control (N = 10)	Presurgery (N = 10)	Amputation (N = 10)	*P*‐value (Χ^2^ or ANOVA)	Control (N = 16)	Amputation (N = 19)	P value (Χ^2^ or *t*‐test)
Mean age (SD) ‐ yr	73.9 (7.8)	64.5 (9.4)	69.5 (6.2)	**0.043** ^†^	62.4 (8.4)	64.6 (10.7)	0.195^‡^
Female sex – no. (%)	4 (40)	0 (0)	1 (10)	**0.044**	9 (56)	6 (32)	0.269
Overweight/Obese (BMI ≥ 25) – no. (%)	9 (90)	7 (70)	8 (80)	0.535	12 (75)	12 (63)	0.476
Ankle‐brachial index (ABI) – (SD)	1.1 (0.1)	0.57 (0.27)^§^	0.54 (0.35)^§^	**0.017** ^†^	ND	0.13 (0.2)^§^	ND
Rutherford Classification – no. (%)							
0	10 (100)	0 (0)	0 (0)	**<0.001**	16 (100)	0 (0)	**<0.001**
3	0 (0)	2 (20)	0 (0)	0.093	0 (0)	0 (0)	ND
4	0 (0)	4 (40)	4 (40)	0.624	0 (0)	2 (11)	**<0.001**
5	0 (0)	4 (40)	4 (40)	0.646	0 (0)	10 (52)	**<0.001**
6	0 (0)	0 (0)	2(20)	0.454	0 (0)	7 (37)	**<0.001**
Hemoglobin A1C (SD)	ND	7.4 (1.1)	7.7 (1.2)	0.505^‡^	ND	5.7 (0.8)	ND
Medical history – no. (%)							
Diabetes mellitus type I or II	4 (40)	6 (60)	9 (90)	0.065	0 (0)	12 (63)	**0.002**
Hypertension	7 (70)	10 (100)	10 (100)	0.536	7 (21)	15 (79)	0.223
Hyperlipidemia	4 (40)	10 (100)	10 (100)	**0.006**	1 (6)	12 (63)	**0.014**
Coronary artery disease	1 (10)	6 (60)	9 (90)	**0.001**	0 (0)	6 (32)	**0.039**
Renal disease	0 (0)	1 (10)	3 (30)	0.133	0 (0)	9 (47)	**0.009**
Chronic obstructive pulmonary disease	1 (10)	4 (40)	3 (30)	0.303	0 (0)	2 (19)	0.315
Tobacco use – no. (%)	4 (40)	7 (70)	9 (90)	0.058	0 (0)	13 (68)	**0.002**
Former smoker	3 (30)	4 (40)	7 (70)	0.175	0 (0)	6 (32)	**0.039**
Current smoker	1 (10)	3 (30)	2 (20)	0.535	0 (0)	7 (36)	**0.024**
Medication used – no. (%)							
Aspirin	4 (40)	8 (80)	9 (90)	**0.035**	1 (6)	11 (58)	**0.019**
Statin	4 (40)	10 (100)	10 (100)	**<0.001**	2 (13)	11 (58)	0.052
ACE inhibitor	5 (50)	5 (50)	6 (60)	0.874	2 (13)	6 (32)	0.254
Cilostazol	0 (0)	3 (30)	4 (40)	0.089	0 (0)	0 (0)	ND
Metformin	4 (40)	3 (30)	4 (40)	0.865	0 (0)	1 (5)	0.920
Previous vascular intervention – no. (%)	0 (0)	0 (0)	5 (50)	**0.003**	0 (0)	13 (68)	ND

^†^ ANOVA was performed. Chi‐squared (X^2^) analysis was performed to determine differences in population proportions.

^‡^ *t*‐test (two‐tailed) was performed. SD, standard deviation.

^§^ Three amputation and two pre‐surgery CLTI patients at UF, and five CLTI patients at ECU had non‐compressible vessels precluding ABI measurement.

### 3.2 The CLTI limb muscle proteome includes unique alterations to the mitochondrion and extracellular matrix

**FIGURE 1:**
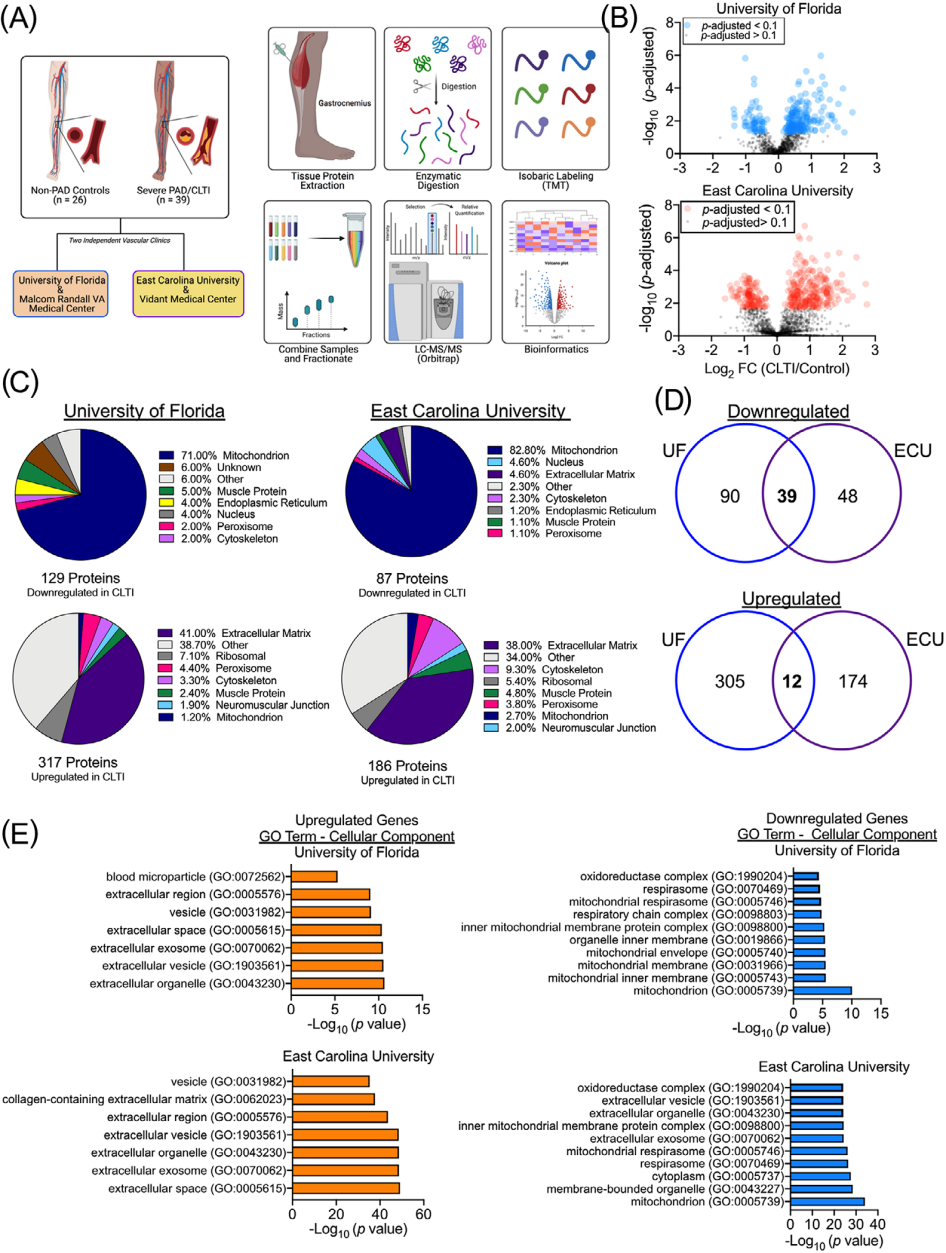
Proteomic Signature of Amputated CLTI limbs. (A) Graphical description of sample collection, processing, and bioinformatics analysis. (B) Volcano plots of proteins identified at both independent vascular clinics. (C) Pie charts depicting the proportions of differentially expressed proteins (upregulated and downregulated) by cellular organelle/component at both sites. (D) Venn diagrams showing similarities and differences in differentially expressed proteins in CLTI specimens across the two clinical sites. (E) Gene ontology analysis of differentially expressed proteins in CLTI demonstrate near identical proteome signatures across clinics

To get a comprehensive and unbiased view of the proteomic landscape in the limb muscles of CLTI patients, muscle specimens from CLTI patients undergoing amputation and non‐ischemic controls without PAD were collected in two independent vascular clinics in separate geographic locations, individually prepared, and independently analyzed (Figure 1A). Analysis of tissues from patients of the first site (University of Florida) yielded 1130 identified proteins with at least two unique peptides quantified across all 30 muscle samples. At the second site (East Carolina University), a total of 1871 proteins were detected across all 35 patient samples. Volcano plots showing differential protein abundances in CLTI specimens versus controls are shown for each independent clinic in Figure 1B. Gene ontology and protein class analysis of differentially expressed proteins indicated that more than 70% of the downregulated proteins were from the mitochondrion (Figure 1C,E). Venn diagram analysis of data (Figure 1D) identified 39 significantly downregulated proteins across both independent clinical sites (Table 2). The largest proportion (>38%) of CLTI specific upregulated proteins within each independent site population were related to the extracellular matrix/space. Venn diagram analysis revealed 12 proteins significantly upregulated at both vascular clinics (Table 3).

**TABLE 2:** Downregulated proteins in CLTI amputation specimens identified at both sites

		University of Florida		East Carolina University	
Gene ID	Gene description	Fold change (CLTI/Control)	*P*‐value	Fold change (CLTI/Control)	*P*‐value
ACAA2	3‐Ketoacyl‐CoA thiolase	0.64	0.00474	0.54	0.00273
ACADS	Short‐chain‐specific acyl‐CoA dehydrogenase	0.61	0.00000	0.43	0.00384
ACAT1	Acetyl‐CoA acetyltransferase	0.56	0.00006	0.48	0.00035
ACO2	Aconitate hydratase	0.73	0.00720	0.54	0.00260
AK3	GTP:AMP phosphotransferase AK3	0.55	0.00011	0.42	0.00224
ALDH5A1	Succinate‐semialdehyde dehydrogenase	0.62	0.00006	0.38	0.00172
APOO	MICOS complex subunit MIC26	0.62	0.00147	0.55	0.00412
CKMT2	creatine kinase, mitochondrial 2	0.50	0.00000	0.45	0.00145
COQ9	Ubiquinone biosynthesis protein	0.59	0.00078	0.51	0.00240
COX4I1	Cytochrome c oxidase subunit 4 isoform 1	0.72	0.00615	0.56	0.00030
COX5A	Cytochrome c oxidase subunit Va	0.82	0.02496	0.55	0.00035
COX7C	Cytochrome c oxidase subunit 7C	0.69	0.00104	0.57	0.00185
CRAT	Carnitine O‐acetyltransferase	0.63	0.00014	0.70	0.00246
CS	Citrate synthase	0.72	0.00493	0.58	0.00718
CYCS	Cytochrome c	0.82	0.03651	0.37	0.00085
DECR1	2,4‐dienoyl‐CoA reductase	0.75	0.03406	0.66	0.00052
DLST	Dihydrolipoyllysine‐residue succinyltransferase component of 2‐oxoglutarate dehydrogenase complex	0.76	0.01305	0.49	0.00653
ECHS1	Enoyl Coenzyme A hydratase, short chain, 1	0.70	0.00207	0.65	0.00100
FHL3	Four and a half LIM domains protein 3	0.80	0.02060	0.58	0.00687
GATD3B	Glutamine amidotransferase‐like class 1 domain‐containing protein 3B	0.53	0.00006	0.64	0.00061
GOT1	Aspartate aminotransferase	0.58	0.00014	0.55	0.00053
HSDL2	Hydroxysteroid dehydrogenase‐like protein 2	0.67	0.00024	0.56	0.00175
IDH2	Isocitrate dehydrogenase 2	0.68	0.00179	0.56	0.00032
MDH2	Malate dehydrogenase 2	0.61	0.00289	0.61	0.00393
NDUFA10	NADH dehydrogenase (Ubiquinone) 1 alpha subcomplex, 10	0.52	0.00047	0.39	0.00137
NDUFC2	NADH dehydrogenase [ubiquinone] 1 subunit C2	0.59	0.00058	0.63	0.00132
NDUFS1	NADH‐ubiquinone oxidoreductase 75 kDa subunit	0.74	0.00557	0.59	0.00091
NDUFS6	NADH dehydrogenase [ubiquinone] iron‐sulfur protein 6	0.65	0.00038	0.55	0.00299
NDUFV1	NADH dehydrogenase [ubiquinone] flavoprotein 1	0.68	0.00146	0.50	0.00062
NIPSNAP2	Protein NipSnap homolog 2	0.62	0.00855	0.68	0.00182
OGDH	2‐oxoglutarate dehydrogenase	0.74	0.00803	0.63	0.00681
PDHA1	Pyruvate dehydrogenase E1 component subunit alpha	0.76	0.02951	0.55	0.00341
SAMM50	Sorting and assembly machinery component 50 homolog	0.66	0.00070	0.53	0.00158
SLC25A11	Mitochondrial 2‐oxoglutarate/malate carrier protein	0.65	0.00622	0.59	0.00334
SLC25A4	ADP/ATP translocase 1	0.71	0.00079	0.56	0.00162
SUCLA2	Succinate–CoA ligase [ADP‐forming] subunit beta	0.62	0.00004	0.33	0.00509
UQCRB	Cytochrome b‐c1 complex subunit 7	0.69	0.00130	0.54	0.00341
UQCRC1	Cytochrome b‐c1 complex subunit 1	0.68	0.00134	0.59	0.00666
UQCRC2	Cytochrome b‐c1 complex subunit 2	0.62	0.00044	0.66	0.00440

**TABLE 3:** Upregulated proteins in CLTI amputation specimens identified at both sites

		University of Florida		East Carolina University	
Gene ID	Gene description	Fold change (CLTI/Control)	*P*‐value	Fold change (CLTI/Control)	*P*‐value
ADH1B	All‐trans‐retinol dehydrogenase [NAD(+)] ADH1B	2.2	0.00023	3.9	0.00002
AKR1C1	Aldo‐keto reductase family 1 member C1	1.8	0.00620	5.4	0.02791
AMBP	Protein AMBP	2.2	0.00974	4.5	<0.00001
CFL1	Cofilin‐1	1.3	0.00388	7.7	<0.00001
DDX39B	Isoform 2 of Spliceosome RNA helicase DDX39B	1.4	0.01493	5.7	<0.00001
F13A1	Coagulation factor XIII A chain	2.6	0.00048	18.7	<0.00001
FTL	Ferritin light chain	3.4	0.00001	18.4	<0.00001
MYH10	Isoform 4 of Myosin‐10	1.5	0.01277	32.6	<0.00001
MYO1C	Unconventional myosin‐Ic	1.5	0.00650	24.3	<0.00001
PA2G4	Proliferation‐associated protein 2G4	1.4	0.00163	4.8	<0.00001
PPIA	Peptidyl‐prolyl cis‐trans isomerase	1.9	0.00002	7.6	<0.00001
TNPO1	Transportin‐1	1.4	0.02530	3.3	<0.00001

### 3.3 Mitochondrial failure is the primary proteome signature of CLTI amputation

**FIGURE 2:**
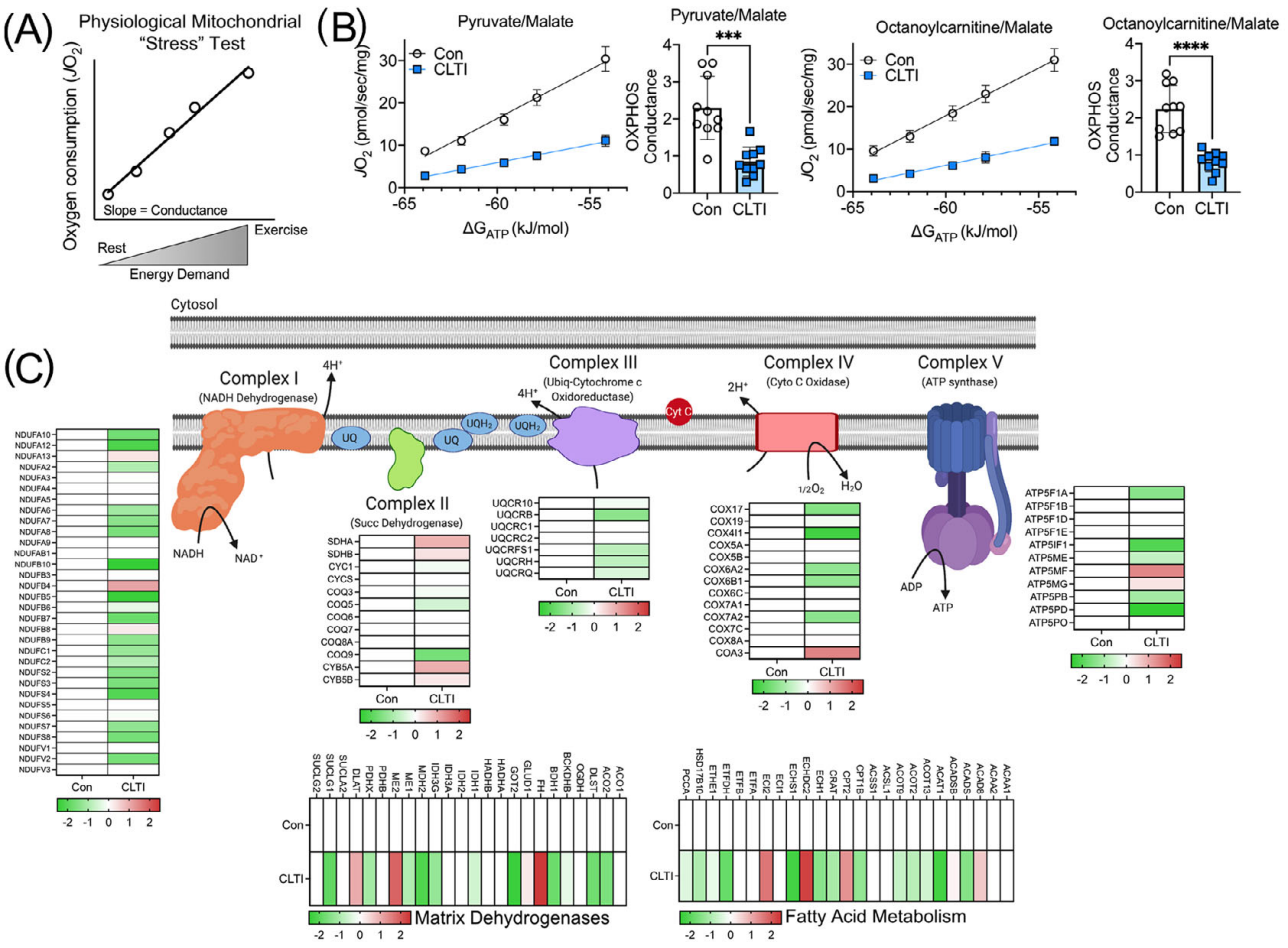
Proteomic changes impart a functional deficit in mitochondria in non‐salvageable CLTI. (A) Graphical depiction of novel mitochondrial “stress” test that mimics physiological levels of energy demand. (B) Quantitative analysis of muscle mitochondrial function supported by carbohydrate (pyruvate/malate) and fatty acid (octanoylcarnitine/malate) demonstrate substantial impairment in mitochondrial function in CLTI (n = 10/group). (C) Proteome differences (Log2 fold change) for non‐PAD control and CLTI amputation specimens according to their role in mitochondrial energy transduction. ***P < 0.001, ****P < 0.0001 using two‐tailed *t*‐test

To understand the consequences of the downregulated mitochondrial proteome in CLTI, we analyzed mitochondrial oxidative phosphorylation (OXPHOS) using high‐resolution respirometry in permeabilized myofibers preparations. Using these methods, we previously demonstrated a uniform mitochondrial functional signature amongst CLTI amputees which was distinguishable from non‐PAD controls and mild claudicating PAD patients.^6^ Here, we specifically analyzed OXPHOS function in permeabilized myofibers using a novel creatine‐kinase clamp protocol. This protocol more closely mimics a patient's physiologic stress‐test by analyzing mitochondrial oxygen consumption across a range of energy demands, similar to the conditions occurring when resting and transitioning to exercise (Figure 2A). Notably, OXPHOS performance in CLTI specimens using either carbohydrate or lipid fuel sources was substantially lower compared to non‐ischemic controls (Figure 2B). Affiliated heat maps (organized by their individual roles in mitochondrial OXPHOS) of the mitochondrial proteome demonstrate clearly divergent proteomic signatures between CLTI and controls (Figure 2C).

### 3.4 The CLTI limb muscle proteome prior to surgical intervention differs from non‐PAD control limbs

**FIGURE 3:**
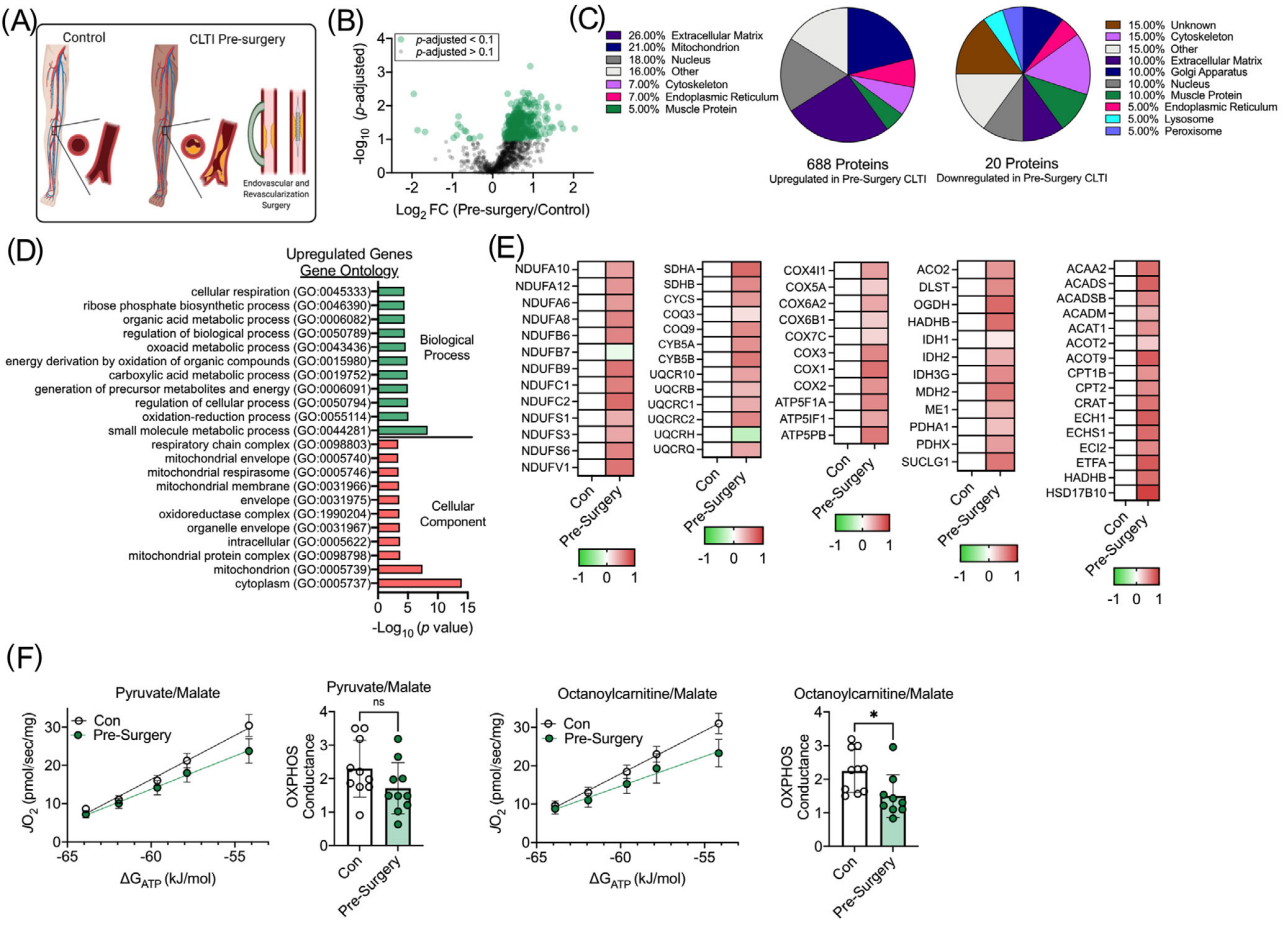
Mitochondrial signature is not present in CLTI prior to surgical intervention. (A) Proteome comparison and (B) Volcano plot between non‐PAD controls and CLTI patients prior to surgical intervention. (C) Pie charts depicting the proportions of differentially expressed proteins (upregulated and downregulated) by cellular organelle/component. (D) Gene ontology identifies upregulation of mitochondrial proteins in CLTI specimens prior to surgical intervention. (E) Heat maps of select mitochondrial protein abundances (Log_2_ fold change). (F) Functional analysis of muscle mitochondria identified a modest impairment in fatty acid oxidation but no difference in carbohydrate oxidation (n = 9‐10/group). *P < 0.05 using two‐tailed *t*‐test

The rates of successful limb salvage and resolution of CLTI symptoms resulting from endovascular or revascularization procedures are relatively low despite improvements in surgical techniques. For this experiment, we sought to examine whether differences in the CLTI limb muscle proteome immediately prior to surgical intervention would uniquely define this patient population and perhaps help identify new temporal clinical predictors of major adverse limb events. Compared to non‐ischemic control samples, those collected from presurgical CLTI limbs possessed 708 differentially abundant proteins (688 upregulated, and 20 downregulated) (Figure 3A,B). Consistent with CLTI amputation specimens, the largest proportion (26%) of upregulated proteins were related to the extracellular matrix (Figure 3C). However, in stark contrast to amputation specimens, the pre‐surgical tissues displayed a robust upregulation of mitochondrial proteins (Figure 3C). In fact, gene ontology analysis indicated the most significant pathways upregulated were related to mitochondrial metabolism (Figure 3D). Heat maps of quantified proteins involved in mitochondrial energy production are shown in Figure 3E. Interestingly, the compensatory upregulation of mitochondrial protein expression did not result in improved OXPHOS function. Functional analysis of mitochondrial OXPHOS indicated that mitochondrial function in CLTI patients before surgery was normal when fuel by carbohydrates (pyruvate/malate) but modestly (∼25%) impaired when fueled by lipid substrates (Figure 3F).

### 3.5 The mitochondrial proteome differentiates presurgery and amputation CLTI patient limb muscles

**FIGURE 4:**
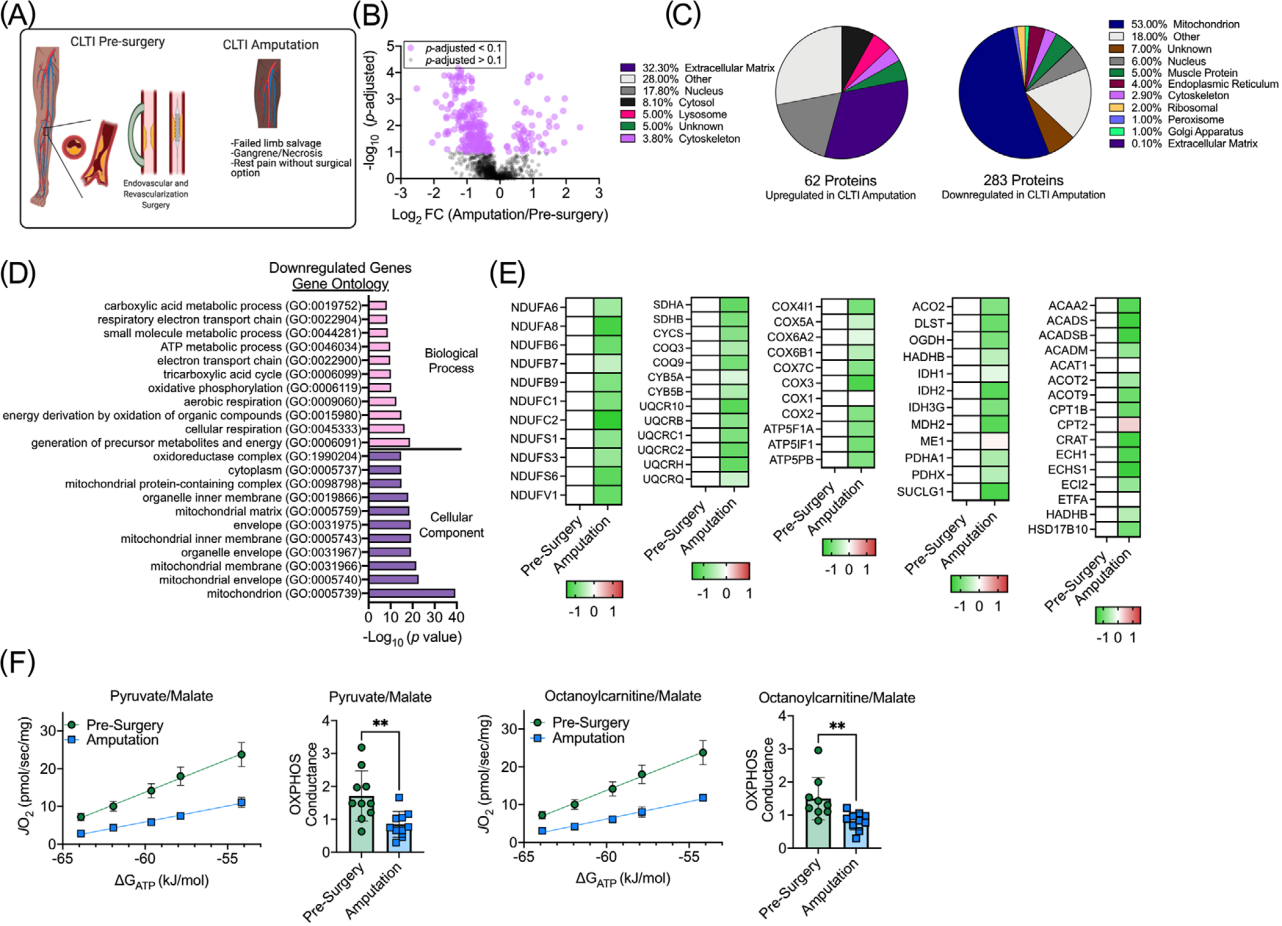
Proteomic signature distinguishes major adverse limb events from presurgery CLTI. (A) Proteome comparison and (B) Volcano plot between CLTI amputations and CLTI patients prior to surgical intervention. (C) Pie charts depicting the proportions of differentially expressed proteins (upregulated and downregulated) by cellular organelle/component. (D) Gene ontology identifies downregulated proteins CLTI amputations compared to CLTI specimens prior to surgical intervention. (E) Heat maps of select mitochondrial protein abundances (Log_2_ fold change). (F) Functional analysis of muscle mitochondria identified a severe impairment in mitochondrial oxidative phosphorylation in CLTI amputations that is not present prior to surgical intervention (*n* = 9‐10/group). **P < 0.01 using two‐tailed *t*‐test

We next directly compared the proteomic signatures of muscles from CLTI patients collected before surgical intervention to those acquired at the time of amputation (Figure 4A). Three hundred fourty‐five proteins were differentially abundant between these populations, with the majority (283 out of 345, 82%) downregulated (Figure 4B,C). Further evaluation of downregulated proteins indicated that 53% were mitochondrial‐related (Figure 4C) which was paralleled in gene ontology analysis (Figure 4D). Heat maps of proteins involved in mitochondrial energy production are shown in Figure 4E. Physiologically, mitochondrial function was significantly decreased in CLTI amputation muscles compared with those analyzed prior to surgery (Figure 4F). Our results indicate sweeping downregulation of the mitochondrial proteome and failure of mitochondrial oxidative phosphorylation defines amputation‐stage muscle specimens in CLTI.

## 4 DISCUSSION

The CLTI presentation of PAD is characterized by significant morbidity, mortality, and substantial health care costs.^1^, ^2^, ^29^ It is most often coupled with considerable comorbid conditions contributing to poor outcomes. These conditions include diabetes, renal disease, and heart disease. Revascularization or interventions focused on restorative blood flow are the cornerstone of therapy for CLTI.^4^, ^30^ Resolution of CLTI symptoms remains low despite endovascular and open surgical techniques and technologies improving significantly over the past two decades.^31^ A limited understanding of tissue‐level characteristics in these patients has undoubtedly led to blunted therapeutic design during this period. The results of this study reveal a distinct proteome signature at the time of major adverse limb events (i.e. amputations) in CLTI that is distinct from both non‐PAD control limbs and CLTI limbs prior to surgical intervention. Bioinformatics revealed biological themes and cellular components unique to CLTI amputations including decreased mitochondrial, oxidative phosphorylation, and cellular respiration. The largest class of upregulated proteins in limb muscle at amputation were related to extracellular matrix remodeling. Importantly, bioinformatics produced near‐identical gene ontology across tissues that were independently obtained, processed, and analyzed from two vascular clinics.

Skeletal muscle function is a strong predictor for morbidity and mortality in PAD patients, regardless of symptomatic presentation.^32^, ^33^, ^34^, ^35^, ^36^, ^37^, ^38^, ^39^ Walking performance and mitochondrial health specifically are strong predictors of disease severity in PAD patients.^6^, ^40^, ^41^, ^42^ Combined with our previous transcriptomics work,^6^ the proteomic data here supports a uniform downregulation of mitochondrial related genes and proteins in the CLTI limb muscle at the time of amputation. We previously established the unique functional deficits of CLTI patient limb muscle mitochondria at this disease stage.^6^ In an attempt to fully understand the functional impact of downregulated mitochondrial proteins identified herein we employed a different strategy, using a novel creatine‐kinase energy clamp system to assess mitochondrial function. Using this specific methodology, the myofibers are exposed to increasing cellular energy demand (mimicking a range contractile activity) akin to a stress test. The relationship between mitochondrial oxygen consumption and energy demands facilitates a calculation of conductance through the oxidative metabolic system (termed OXPHOS conductance herein).^25^, ^43^ Myofibers prepared from amputation limbs exhibited low OXPHOS conductance, fully demonstrating that the muscle mitochondria are incapable of adequately responding to changes of energy demand by increasing oxidative phosphorylation. This would lead to increased reliance on non‐oxidative metabolic pathways, especially during increased levels of energy demand, such as those required for normal activities of daily living. This is undoubtedly a problem, as we have also demonstrated decreases in the limb muscle transcriptome related to glycolytic flux in CLTI patients.^44^ Additionally, functional glycolytic flux is blunted in primary muscle myotubes from these patients in vitro. This wholesale failure of energy charge in the limb muscles of CLTI patients at amputation may provide bioenergetic insight into the exercise‐, and general physical activity‐ intolerance that plagues this clinical presentation.

Interestingly, we report that catastrophic deficits in oxidative metabolism and the mitochondrial proteome discovered at amputation do not uniformly characterize CLTI limbs prior to surgical intervention. Although there were some similarities with increased protein abundances between these populations related to extracellular matrix remodeling, the second‐largest class of upregulated proteins (21%) in CLTI presurgery specimens were related to mitochondrial health. Increased mitochondrial proteins in the CLTI patients prior to surgical intervention, in stark contrast to the widespread downregulation that occurs at the time of amputation, would seem to indicate that these tissues are still capable of mounting some sort of biological/bioenergetic response to the chronic ischemic insult. Of particular relevance to this idea, however, is that 62% of the CLTI amputation patients enrolled in this study across both clinical sites had previously undergone a surgical intervention of some kind to restore limb muscle blood flow. A sufficient mitochondrial proteomic signature in these patients at the time of intervention might logically lead one to assume that the limb tissues would be efficiently capable of utilizing oxidative metabolism to regenerate or restore muscle function after surgery. Unfortunately, this does not appear to be the case. Our data actually suggest that limb pathology in CLTI does not necessarily involve a progressive decline in mitochondrial health as a result of reduced blood flow to the limb muscles, but rather the wholesale collapse of mitochondrial health in non‐salvageable limbs. Frustratingly, in CLTI patients this seems to occur at high frequency regardless of the restoration of blood flow and independent of vessel patency after intervention. This also seems to occur in these patients despite relatively stable total mitochondrial numbers according to established biomarkers including citrate synthase activity, mtDNA/nDNA, and cardiolipin content.^6^ Collectively, these data paint a clear picture of the non‐salvageable limb with a microenvironment populated by fragile limb muscle mitochondria.

Development of fibrosis has been previously reported in muscle biopsy specimens from PAD patients.^45^, ^46^ Uniformity in expanded extracellular matrix protein abundance across the CLTI population supports the histological characterization of the limb muscles from these patients as pro‐fibrotic.^7^, ^47^ The expansion of non‐contractile tissues in response to limb ischemia is not limited to humans, as this is also a hallmark of inbred strains of mice that do not regenerate and recover limb muscle perfusion or function after hindlimb ischemia.^48^, ^49^, ^50^, ^51^ Fibrosis, in general, is a common occurrence in muscles that fail to adequately respond to a degenerative insult. Remodeling of the extracellular matrix has important functional roles to facilitate cell proliferation, migration, differentiation, and survival^52^ that are processes crucial for both angiogenesis and muscle regeneration. Additionally, emerging evidence has begun to link aberrant mitochondrial function to organ fibrosis^53^ suggesting that the observed proteome changes could be mechanistically linked histopathology in CLTI limb muscle. Currently, the functional impact of pathologic remodeling of extracellular compartment in the ischemic limb is unknown but would likely contribute to poor muscle contractile performance and activity intolerance in PAD/CLTI patients.^34^, ^54^ To this end, it is noteworthy to consider that numerous approved drugs exert their effects by modulating extracellular matrix remodeling.^52^ Whether these classes of drugs can improve limb function in CLTI has not been investigated to date but provides an interesting therapeutic avenue.

## 5 STUDY LIMITATIONS

There were some limitations to the current study. First, this work was cross‐sectional in nature, therefore no causal inferences should be made regarding the role of proteome and mitochondrial functions changes as tissue‐level mechanisms leading to limb amputation. Future studies to rigorously establish causality in limb muscle mitochondrial function in amputation outcomes would require several things, including: (1) establishment of mitochondrial dysfunction as a primary determinant of amputation/pathologic outcomes (clinically/pre‐clinically, respectively), (2) determination of sufficiency for limb muscle mitochondrial function in the prevention of the CLTI phenotype/presentation (preclinically/clinically, respectively), and/or (3) repeated limb muscle biopsies in the same patient cohorts coupled with proteomic and mitochondrial analyses. Second, this exploratory proteomics analysis was performed on a relatively small sample size (65 patients) and thus should be considered provisionary in nature. Despite the relatively small sample size, confidence in the identified proteome changes is enhanced by the fact that two independent patient cohorts from separate sites and independently‐conducted proteomic analyses identified common cellular components that were altered in CLTI. However, differences in the methods used for tissue lysis/digestion, TMT‐labeling, and mass spectrometry analysis across sites may have diminished the ability to identify more common protein targets across sites. Fourth, there may be modest differences in the clinical severity of presurgery and amputation CLTI patients, indicated by the Rutherford classification but not hemodynamic outcomes (ABI), which could have impacted the proteome signature.

## 6 CONCLUSIONS

Amputation outcomes in CLTI are defined by a failing cellular microenvironment that may occur despite technically successful surgical intervention. The underlying mechanisms contributing to the unacceptably high amputation rates in this patient population are still somewhat unclear, however this work contributes critical information to a recently expanding book on these patients.^6^, ^7^, ^8^, ^10^, ^11^, ^44^, ^47^, ^55^ This study, in particular, builds on transcriptomics data to highlight a deficit in limb muscle mitochondrial function and the corresponding mitochondrial proteome that defines the non‐salvageable ischemic limb. Although the exact clinical application of these results is unknown presently, several applications can be envisioned. First, the observation that amputation tissues display a mitochondrial deficit that is distinguishable from CLTI tissues prior to surgical intervention suggests that future studies involving longitudinal analysis of mitochondrial health both prior to‐ and following surgical intervention are needed to determine if mitochondrial health can identify patients with the greatest risk for amputation. While not as technically in depth as myofiber isolation and direct substrate‐driven OXPHOS interrogation, mitochondrial analyses can be more easily performed using non‐invasive technologies.^56^, ^57^ Given that a muscle mitochondrial deficit also distinguishes pathology in CLTI‐susceptible mice,^58^ and mitochondrial‐targeted therapy improves outcomes in mice with ischemic mitochondriopathy,^59^ it is reasonable to hypothesize that therapeutic targeting mitochondrial health in CLTI may prove beneficial. This is an exciting avenue for innovative advancement in patients with few interventional options at present.

## DISCLOSURES

The authors have no conflicts, financial or otherwise, to report.

## Supporting information

Supporting Information

## Data Availability

The data that support the findings of this study are available online using accession numbers PXD021849 and PXD025810 (Proteome Xchange) or JPST000852 and JPST001157 (jPOST Repository).
