# Supplementary material for: Interventional‐ and amputation‐stage muscle proteomes in the chronically threatened ischemic limb
Source: Clin Transl Med. 2022 Jan 24;12(1):e658. doi: 10.1002/ctm2.658 (PMC8785983; doi:10.1002/ctm2.658)
Supplement: Supplementary file 1 — Supporting Information [file CTM2-12-e658-s001.pdf]

Major Resources Table

Data & Code Availability

Description	Source / Repository	Persistent ID / URL
Proteomics Data	Proteome Exchange	PXD021849 and PXD025810
Proteomics Data	jPOST Repository	JPST000852 and JPST001157

Univ. of Florida Proteomics Results

Accession	Description	Log2 FC (CLTI Amputation/Control)	Log2 FC (CLTI Pre-surgery/Control)	Log2 FC (CLTI Amputation/CLTI Pre-surgery)	P (adjusted) Control vs. CLTI Amputation	P(adjusted) Control vs CLTI Pre-surgery	P (-adjusted) CLTI Pre-surgery vs. CLTI Amputation
A0A5F9ZHL1	Acetyl-CoA acetyltransferase, mitochondrial OS=Homo sapiens OX=9606 GN=ACAT1 PE=1 SV=1	-1.008392926	0.361612353	-1.37000528	0.000379606	0.035498249	1.75471E-05
P48735	Isocitrate dehydrogenase [NADP], mitochondrial OS=Homo sapiens OX=9606 GN=IDH2 PE=1 SV=2	-0.718434833	0.290882766	-1.009317599	0.000625638	0.031151566	1.35757E-05
Q76LA1	CSTB protein OS=Homo sapiens OX=9606 GN=CSTB PE=2 SV=1	0.839289908	0.03102149	0.808268419	0.000716822	0.848440485	2.75506E-05
Q05DB4	HEBP2 protein (Fragment) OS=Homo sapiens OX=9606 GN=HEBP2 PE=2 SV=1	0.607793758	-0.082914886	0.690708644	0.001057054	0.294330685	2.09914E-05
Q6S4P3	Ferritin OS=Homo sapiens OX=9606 GN=FTL PE=1 SV=1	1.751003692	0.679117951	1.071885741	0.00174434	0.003297071	0.011550688
Q6PK82	AP3D1 protein (Fragment) OS=Homo sapiens OX=9606 GN=AP3D1 PE=2 SV=1	1.106486222	1.194338905	-0.087852683	0.002219702	0.000528221	0.580127748
G3V2U7	Acylphosphatase OS=Homo sapiens	0.690579326	0.599740961	0.090838364	0.002392256	0.0070024	0.750044916

	OX=9606 GN=ACYP1 PE=1 SV=1						
Q6ZR44	cDNA FLJ46672 fis, clone TRACH3009008, highly similar to Thioredoxin reductase OS=Homo sapiens OX=9606 PE=2 SV=1	0.497843721	0.301898813	0.195944908	0.002490209	0.033775299	0.094716506
P62937	Peptidyl-prolyl cis-trans isomerase A OS=Homo sapiens OX=9606 GN=PPIA PE=1 SV=2	0.957498016	0.385953187	0.571544829	0.002554402	0.014779989	0.066989638
A4UJ43	Glutathione S-transferase OS=Homo sapiens OX=9606 GN=GSTM3 PE=2 SV=1	0.713146636	0.181863248	0.531283388	0.002571465	0.343491387	0.00621933
P15954	Cytochrome c oxidase subunit 7C, mitochondrial OS=Homo sapiens OX=9606 GN=COX7C PE=1 SV=1	-0.681911184	0.180826157	-0.862737341	0.003400105	0.023435126	1.086E-05
K7ER00	Phenylalanine--tRNA ligase alpha subunit OS=Homo sapiens OX=9606 GN=FARSA PE=1 SV=1	0.581685361	0.804104596	-0.222419235	0.003488447	0.003523884	0.255071081
X5D299	Succinate-semialdehyde dehydrogenase (Fragment) OS=Homo sapiens OX=9606 GN=ALDH5A1 PE=2 SV=1	-0.840761978	0.311756971	-1.152518949	0.003495566	0.098254128	8.8464E-05
Q6NS36	Ferritin (Fragment) OS=Homo sapiens OX=9606 GN=FTH1 PE=2 SV=1	1.241119294	0.073369067	1.167750227	0.003514329	0.591263606	0.000866285
P00441	Superoxide dismutase [Cu-Zn] OS=Homo sapiens OX=9606 GN=SOD1 PE=1 SV=2	0.691877998	-0.091232906	0.783110905	0.003583241	0.332191925	0.000149439
O60841	Eukaryotic translation initiation factor 5B OS=Homo sapiens OX=9606 GN=EIF5B PE=1 SV=4	0.947254626	0.815080197	0.132174429	0.003586046	0.001855827	0.767353579
A0A0B4J2D5	Glutamine amidotransferase-like class 1 domain-	-0.927770536	0.0735697	-1.001340236	0.003638512	0.306618248	0.000474571

	containing protein 3B, mitochondrial OS=Homo sapiens OX=9606 GN=GATD3B PE=1 SV=1						
Q5TAL4	U1 small nuclear ribonucleoprotein C OS=Homo sapiens OX=9606 GN=SNRPC PE=1 SV=1	1.025309665	0.979992938	0.045316727	0.003645417	0.002282416	0.927008677
P23786	Carnitine O-palmitoyltransferase 2, mitochondrial OS=Homo sapiens OX=9606 GN=CPT2 PE=1 SV=2	1.028570696	0.649945326	0.378625371	0.003667827	0.004815913	0.169514257
A0A024R6W0	Aspartate aminotransferase OS=Homo sapiens OX=9606 GN=GOT2 PE=4 SV=1	-1.002070341	-0.68533781	-0.316732531	0.003695163	0.086597677	0.373201297
F8WCI5	Protein C3orf33 OS=Homo sapiens OX=9606 GN=C3orf33 PE=1 SV=1	-0.884125009	0.313320769	-1.197445778	0.003713562	0.011391467	7.2838E-06
Q92800	Histone-lysine N-methyltransferase EZH1 OS=Homo sapiens OX=9606 GN=EZH1 PE=1 SV=2	0.647156443	0.020204686	0.626951757	0.003829042	0.84111823	0.000269379
P12235	ADP/ATP translocase 1 OS=Homo sapiens OX=9606 GN=SLC25A4 PE=1 SV=4	-0.681983786	0.095826934	-0.77781072	0.00386058	0.487400949	0.00087257
A0A384NPU2	Epididymis secretory sperm binding protein OS=Homo sapiens OX=9606 GN=HINT1 PE=2 SV=1	0.485302558	-0.381991812	0.867294371	0.004056482	0.002979893	6.06137E-06
H3BUZ6	Monoacylglycerol lipase ABHD2 (Fragment) OS=Homo sapiens OX=9606 GN=ABHD2 PE=1 SV=1	0.79543019	0.292287904	0.503142286	0.0042669	0.088350544	0.030454591
Q06124	Tyrosine-protein phosphatase non-receptor type 11 OS=Homo sapiens	0.68070583	0.528683303	0.152022527	0.004292224	0.002230976	0.753873657

	OX=9606 GN=PTPN11 PE=1 SV=3						
Q8NFW8	N-acylneuraminate cytidylyltransferase OS=Homo sapiens OX=9606 GN=CMAS PE=1 SV=2	0.687371755	0.801709559	-0.114337804	0.004328873	0.002199928	0.399004378
P17540	Creatine kinase S-type, mitochondrial OS=Homo sapiens OX=9606 GN=CKMT2 PE=1 SV=2	-0.866175513	0.508534006	-1.37470952	0.004348131	0.058319001	0.000320705
Q9HBS9	Uncharacterized protein OS=Homo sapiens OX=9606 PE=2 SV=1	-0.645749689	-0.488976135	-0.156773553	0.004349147	0.012181197	0.498861847
D3DSQ7	Exportin 7, isoform CRA_c OS=Homo sapiens OX=9606 GN=XPO7 PE=4 SV=1	-1.263737029	0.324890959	-1.588627987	0.004429072	0.205169502	0.000463573
P06703	Protein S100-A6 OS=Homo sapiens OX=9606 GN=S100A6 PE=1 SV=1	1.109660572	0.655609912	0.45405066	0.004507057	0.00979493	0.147965743
B3KT06	Tubulin alpha chain OS=Homo sapiens OX=9606 PE=2 SV=1	0.53486991	0.436103902	0.098766008	0.004511851	0.003335862	0.883211311
A0A024R8K7	Integrin beta OS=Homo sapiens OX=9606 GN=ITGB4 PE=3 SV=1	0.59023052	-0.070834413	0.661064933	0.00455515	0.301774692	4.52845E-05
Q59FM0	NADH dehydrogenase (Ubiquinone) 1 alpha subcomplex, 10, 42kDa variant (Fragment) OS=Homo sapiens OX=9606 PE=2 SV=1	-0.795153996	0.296072431	-1.091226427	0.004627273	0.03138095	2.71106E-05
E7CLZ5	Cytochrome c oxidase subunit 3 OS=Homo sapiens OX=9606 GN=COX3 PE=3 SV=1	-0.790105572	0.395454746	-1.185560319	0.004636652	0.012400074	6.62347E-05
A0A0S2Z2Z6	Annexin (Fragment) OS=Homo sapiens OX=9606 GN=ANXA6 PE=2 SV=1	0.45399904	0.398442883	0.055556157	0.004688633	0.002421639	0.852674058
A0A384MDW7	Enoyl Coenzyme A hydratase, short chain, 1, mitochondrial OS=Homo sapiens	-0.667814721	0.578553003	-1.246367724	0.004734887	0.002753961	5.90482E-06

	OX=9606 GN=ECHS1 PE=2 SV=1						
C1KH65	Alpha-1-antitrypsin null variant (Fragment) OS=Homo sapiens OX=9606 GN=AAT PE=2 SV=1	1.279807669	0.304309729	0.975497939	0.004813549	0.165273766	0.005686535
Q9UDW1	Cytochrome b-c1 complex subunit 9 OS=Homo sapiens OX=9606 GN=UQCR10 PE=1 SV=3	-0.66609305	0.451377344	-1.117470394	0.004898691	0.026839462	5.55326E-05
Q15365	Poly(rC)-binding protein 1 OS=Homo sapiens OX=9606 GN=PCBP1 PE=1 SV=2	0.399563888	0.335797266	0.063766622	0.005158643	0.008989841	0.943417803
Q567R0	UQCRH protein OS=Homo sapiens OX=9606 GN=UQCRH PE=2 SV=1	-1.333368801	-0.286635698	-1.046733103	0.005269508	0.545365068	0.0827293
H3BRG4	Cytochrome b-c1 complex subunit 2, mitochondrial OS=Homo sapiens OX=9606 GN=UQCRC2 PE=1 SV=1	-0.584139877	0.420111127	-1.004251004	0.00539596	0.011959755	4.60262E-05
P00325	All-trans-retinol dehydrogenase [NAD(+)] ADH1B OS=Homo sapiens OX=9606 GN=ADH1B PE=1 SV=2	1.165820764	0.722787583	0.443033181	0.005418852	0.00778205	0.531158749
Q6P1N4	IQGAP1 protein (Fragment) OS=Homo sapiens OX=9606 GN=IQGAP1 PE=2 SV=1	1.318955683	1.261582082	0.057373602	0.005450505	0.0004748	0.880037342
A0A059RPW0	Cytochrome c oxidase subunit 1 (Fragment) OS=Homo sapiens OX=9606 GN=COX1 PE=3 SV=1	-1.020750807	0.399325415	-1.420076222	0.005474529	0.005341427	1.40317E-05
F2Z2K0	NSFL1 cofactor p47 OS=Homo sapiens OX=9606 GN=NSFL1C PE=1 SV=1	0.48467294	0.320410458	0.164262482	0.005497433	0.006920095	0.500488395
Q9Y2J8	Protein-arginine deiminase type-2 OS=Homo sapiens	0.571562962	0.600607156	-0.029044194	0.005527427	0.002182549	0.672874578

	OX=9606 GN=PADI2 PE=1 SV=2					
B3KWP7	cDNA FLJ43538 fis, clone PLACE7008431, highly similar to Homo sapiens phosphatidylinositol-4-phosphate 5-kinase, type II, gamma (PIP5K2C), mRNA OS=Homo sapiens OX=9606 PE=2 SV=1	0.660615435	1.021020125	-0.36040469	0.005533475	0.002950027	0.199929038
A0A0S2Z3P9	Acyl-CoA dehydrogenase short/branched chain isoform 1 (Fragment) OS=Homo sapiens OX=9606 GN=ACADSB PE=2 SV=1	-0.78305518	0.329127116	-1.112182295	0.005568992	0.0126039	0.000250383
Q86WU2	Probable D-lactate dehydrogenase, mitochondrial OS=Homo sapiens OX=9606 GN=LDHD PE=1 SV=1	-0.439630514	0.325059129	-0.764689643	0.005644712	0.017772525	9.40282E-05
Q9Y237	Peptidyl-prolyl cis-trans isomerase NIMA-interacting 4 OS=Homo sapiens OX=9606 GN=PIN4 PE=1 SV=1	0.639332185	0.049749666	0.58958252	0.005656027	0.803962671	0.004437766
P29401	Transketolase OS=Homo sapiens OX=9606 GN=TKT PE=1 SV=3	0.829364199	0.753158349	0.07620585	0.005745834	0.002124034	0.745016279
A0A3B3ISG8	Ricin B-type lectin domain-containing protein OS=Homo sapiens OX=9606 PE=4 SV=1	-0.637029907	0.436019191	-1.073049098	0.006401112	0.006903474	7.51332E-06
J3QR68	Haptoglobin (Fragment) OS=Homo sapiens OX=9606 GN=HP PE=1 SV=1	1.39675367	-0.012924861	1.40967853	0.006430104	0.68422255	0.000753486
O60343	TBC1 domain family member 4 OS=Homo sapiens OX=9606 GN=TBC1D4 PE=1 SV=2	0.607061056	0.727364407	-0.120303352	0.006443611	0.001419035	0.447982729
F1T0I1	Protein transport protein sec16 OS=Homo	0.633777588	0.637485026	-0.003707438	0.006479793	0.003485113	0.657702538

	sapiens OX=9606 GN=SEC16A PE=1 SV=1						
F8VRL4	Diphosphoinositol polyphosphate phosphohydrolase 2 (Fragment) OS=Homo sapiens OX=9606 GN=NUDT4 PE=1 SV=1	0.630044076	0.383294357	0.246749719	0.006823208	0.005986463	0.391087629
A0A024R9T5	HCG20693, isoform CRA_a OS=Homo sapiens OX=9606 GN=hCG_20693 PE=4 SV=1	0.625119337	0.612185891	0.012933446	0.006853998	0.002207721	0.735100138
O75923	Dysferlin OS=Homo sapiens OX=9606 GN=DYSF PE=1 SV=1	0.393604192	0.500045249	-0.106441057	0.006854986	0.010234603	0.394882715
O75380	NADH dehydrogenase [ubiquinone] iron-sulfur protein 6, mitochondrial OS=Homo sapiens OX=9606 GN=NDUFS6 PE=1 SV=1	-0.621304105	0.460992126	-1.082296231	0.006915743	0.004532191	3.75881E-06
Q5JTC6	APC membrane recruitment protein 1 OS=Homo sapiens OX=9606 GN=AMER1 PE=1 SV=2	-1.290207167	0.548117573	-1.838324739	0.006924091	0.038322967	3.92274E-05
A0A2L0RI76	Isovalery CoA dehydrogenase OS=Homo sapiens OX=9606 GN=IVD PE=3 SV=1	-0.720482951	0.355725602	-1.076208553	0.006948058	0.027540319	0.000869022
D3DPU2	Adenylyl cyclase-associated protein OS=Homo sapiens OX=9606 GN=CAP1 PE=2 SV=1	0.796377308	0.80751864	-0.011141332	0.007004049	0.001068823	0.65636857
Q12899	Tripartite motif-containing protein 26 OS=Homo sapiens OX=9606 GN=TRIM26 PE=1 SV=1	0.812631253	0.678922186	0.133709067	0.007040944	0.002911245	0.872736983
A0A0D9SFW8	HLA class I histocompatibility antigen, alpha chain F (Fragment) OS=Homo	0.882431472	1.100738106	-0.218306634	0.007430956	0.007760911	0.464973449

	sapiens OX=9606 GN=HLA-F PE=1 SV=1						
Q9Y512	Sorting and assembly machinery component 50 homolog OS=Homo sapiens OX=9606 GN=SAMM50 PE=1 SV=3	-0.699159037	-0.342286103	-0.356872934	0.007456889	0.391375427	0.095036459
Q02750	Dual specificity mitogen-activated protein kinase kinase 1 OS=Homo sapiens OX=9606 GN=MAP2K1 PE=1 SV=2	0.555149678	0.22790335	0.327246328	0.007464885	0.067754431	0.074290832
A0A0S2Z3V1	EGF containing fibulin-like extracellular matrix protein 1 isoform 2 (Fragment) OS=Homo sapiens OX=9606 GN=EFEMP1 PE=1 SV=1	1.074777991	0.292577472	0.782200519	0.00754363	0.032802059	0.057299715
B3KUZ8	Aspartate aminotransferase OS=Homo sapiens OX=9606 PE=2 SV=1	-0.951359526	0.1102526	-1.061612125	0.007635784	0.585262329	0.013235817
B2R6V9	cDNA, FLJ93141, highly similar to Homo sapiens coagulation factor XIII, A1 polypeptide (F13A1), mRNA OS=Homo sapiens OX=9606 PE=2 SV=1	1.369934827	0.66511062	0.704824206	0.007756844	0.012059558	0.337788472
Q53HG0	Eukaryotic translation initiation factor 3, subunit 3 gamma, 40kDa variant (Fragment) OS=Homo sapiens OX=9606 PE=2 SV=1	0.709455724	0.65363319	0.055822534	0.007898636	0.011149113	0.767171967
P51991	Heterogeneous nuclear ribonucleoprotein A3 OS=Homo sapiens OX=9606 GN=HNRNPA3 PE=1 SV=2	0.795187878	0.514946407	0.280241471	0.008113812	0.004823769	0.57755943
Q16762	Thiosulfate sulfurtransferase OS=Homo sapiens OX=9606 GN=TST PE=1 SV=4	-0.654832841	0.565422667	-1.220255508	0.008280642	0.003313046	1.45503E-05

C9JZ80	Tubulin epsilon and delta complex protein 1 (Fragment) OS=Homo sapiens OX=9606 GN=TEDC1 PE=4 SV=1	-0.729365115	0.380854445	-1.11021956	0.008410595	0.036929857	1.26087E-05
V9HWA3	Epididymis secretory sperm binding protein Li 167mP OS=Homo sapiens OX=9606 GN=HEL-S-167mP PE=2 SV=1	0.713687742	-0.114127745	0.827815488	0.008411164	0.208829366	9.32716E-05
Q6YN16	Hydroxysteroid dehydrogenase-like protein 2 OS=Homo sapiens OX=9606 GN=HSDL2 PE=1 SV=1	-0.770053123	0.127574845	-0.897627968	0.00857327	0.173645717	0.001715637
P08758	Annexin A5 OS=Homo sapiens OX=9606 GN=ANXA5 PE=1 SV=2	0.930679033	0.773362081	0.157316952	0.009005415	0.018239627	0.702681068
Q4W4Y1	Dopamine receptor interacting protein 4 OS=Homo sapiens OX=9606 GN=DRIP4 PE=2 SV=1	0.441679981	0.433655216	0.008024765	0.009221655	0.002620197	0.943821473
A8K2M0	Proteasome (Prosome, macropain) 26S subunit, ATPase, 4, isoform CRA_b OS=Homo sapiens OX=9606 GN=PSMC4 PE=2 SV=1	0.425855836	0.466493723	-0.040637886	0.009409843	0.003692139	0.513429327
B7ZA42	cDNA, FLJ79056 (Fragment) OS=Homo sapiens OX=9606 PE=2 SV=1	0.577193387	0.637456374	-0.060262988	0.00943593	0.005069402	0.447616318
P05109	Protein S100-A8 OS=Homo sapiens OX=9606 GN=S100A8 PE=1 SV=1	1.117414653	0.087129945	1.030284708	0.009472193	0.605097399	0.01982765
Q6ZMS3	cDNA FLJ16726 fis, clone UTERU3014791 OS=Homo sapiens OX=9606 PE=2 SV=1	0.538964936	0.820153806	-0.281188869	0.009498039	0.003271554	0.125562982
P15121	Aldo-keto reductase family 1 member B1 OS=Homo sapiens OX=9606 GN=AKR1B1 PE=1 SV=3	0.359338952	-0.159602657	0.518941608	0.00949959	0.074511464	0.000336136

Q71V07	Signal recognition particle subunit SRP72 OS=Homo sapiens OX=9606 PE=2 SV=1	0.884361599	0.802896617	0.081464983	0.009510274	0.001268326	0.720319025
A0A1X7SBS1	Heterogeneous nuclear ribonucleoprotein U OS=Homo sapiens OX=9606 GN=HNRNPU PE=1 SV=1	1.213178785	1.085237157	0.127941628	0.009607513	0.001476693	0.963759845
E5RHG9	Cytochrome b-c1 complex subunit 7 OS=Homo sapiens OX=9606 GN=UQCRB PE=1 SV=1	-0.602934331	0.251623861	-0.854558193	0.009660642	0.039840554	3.95528E-05
O43209	Tubulin beta chain (Fragment) OS=Homo sapiens OX=9606 PE=2 SV=1	0.482300865	0.405066316	0.077234548	0.009709495	0.005351702	0.940094466
P31930	Cytochrome b-c1 complex subunit 1, mitochondrial OS=Homo sapiens OX=9606 GN=UQCRC1 PE=1 SV=3	-0.495171334	0.381585077	-0.876756411	0.009972623	0.00773055	2.76603E-05
B2R8A3	cDNA, FLJ93805, highly similar to Homo sapiens creatine kinase, mitochondrial 2 (sarcomeric) (CKMT2), nuclear gene encoding mitochondrial protein, mRNA OS=Homo sapiens OX=9606 PE=2 SV=1	-0.590299013	0.606417814	-1.196716827	0.01000408	0.03605931	0.00066042
O95298	NADH dehydrogenase [ubiquinone] 1 subunit C2 OS=Homo sapiens OX=9606 GN=NDUFC2 PE=1 SV=1	-0.770905773	0.521816323	-1.292722096	0.010004467	0.006244955	3.25695E-06
A0A2Y9D025	NADH dehydrogenase [ubiquinone] 1 alpha subcomplex subunit 6 OS=Homo sapiens OX=9606 GN=NDUFA6 PE=1 SV=1	-0.439579644	0.332787805	-0.772367449	0.010532548	0.01952446	0.000323978
A0A087X0X3	Heterogeneous nuclear ribonucleoprotein M OS=Homo sapiens	0.676544847	0.422342401	0.254202446	0.010589621	0.151749649	0.314102286

	OX=9606 GN=HNRNPM PE=1 SV=1						
B3GUD3	Protein tyrosine phosphatase-2 (Fragment) OS=Homo sapiens OX=9606 GN=PTPN11 PE=2 SV=1	0.419533297	0.41704593	0.002487367	0.010623623	0.003986942	0.564257063
F5GZZ9	Scavenger receptor cysteine-rich type 1 protein M130 OS=Homo sapiens OX=9606 GN=CD163 PE=1 SV=1	1.314557422	0.545537113	0.769020309	0.010640907	0.005198664	0.143805188
P22061	Protein-L-isoaspartate(D-aspartate) O-methyltransferase OS=Homo sapiens OX=9606 GN=PCMT1 PE=1 SV=4	0.40358503	0.40898581	-0.00540078	0.010853901	0.011193785	0.940713683
O94760	N(G),N(G)-dimethylarginine dimethylaminohydrolase 1 OS=Homo sapiens OX=9606 GN=DDAH1 PE=1 SV=3	0.633803243	0.182895397	0.450907846	0.011236246	0.19173781	0.036117171
H7C463	MICOS complex subunit MIC60 (Fragment) OS=Homo sapiens OX=9606 GN=IMMT PE=1 SV=1	-0.806546142	0.341332919	-1.147879061	0.011287053	0.03202675	7.98943E-05
Q6IS14	Eukaryotic translation initiation factor 5A-1-like OS=Homo sapiens OX=9606 GN=EIF5AL1 PE=2 SV=2	0.457060449	0.091864439	0.36519601	0.01139367	0.544959685	0.012619923
Q6PKG0	La-related protein 1 OS=Homo sapiens OX=9606 GN=LARP1 PE=1 SV=2	0.70096476	0.851767131	-0.150802371	0.011418981	0.002489663	0.435909199
A0A384NKS6	Clusterin OS=Homo sapiens OX=9606 PE=2 SV=1	1.060508277	0.342966757	0.71754152	0.011509951	0.248344554	0.323040487
A0A291FIZ5	NDUFS1 OS=Homo sapiens OX=9606 PE=3 SV=1	-0.539575589	0.206037099	-0.745612689	0.01163483	0.041333572	4.56964E-05

Q75MS4	Uncharacterized protein IFRD1 (Fragment) OS=Homo sapiens OX=9606 GN=IFRD1 PE=4 SV=1	-0.714606268	0.469790966	-1.184397233	0.011709813	0.017941462	3.64671E-05
Q59FK3	Annexin (Fragment) OS=Homo sapiens OX=9606 PE=2 SV=1	0.61609582	0.482099778	0.133996042	0.011771658	0.01252815	0.998054047
A4D1P0	Aldo_ket_red domain-containing protein OS=Homo sapiens OX=9606 GN=LOC402299 PE=4 SV=1	0.421715557	-0.255228827	0.676944384	0.011866906	0.036894517	0.000473067
P50135	Histamine N-methyltransferase OS=Homo sapiens OX=9606 GN=HNMT PE=1 SV=1	0.952586908	0.730338451	0.222248457	0.011942672	0.002466042	0.956685035
E9PQD6	Serum amyloid A protein OS=Homo sapiens OX=9606 GN=SAA1 PE=1 SV=1	1.549439765	0.535527771	1.013911995	0.013040806	0.003975004	0.099587603
A0A0K0K1J6	Epididymis secretory protein Li 96 (Fragment) OS=Homo sapiens OX=9606 GN=HEL-S-96 PE=2 SV=1	0.381551276	0.132976477	0.248574799	0.013154163	0.445053421	0.072346806
H3BS02	Protein-serine/threonine kinase OS=Homo sapiens OX=9606 GN=BCKDK PE=1 SV=1	-0.589001762	0.168467078	-0.75746884	0.013159687	0.124442284	0.000877145
A0A140VK39	Protein phosphatase methylesterase 1 OS=Homo sapiens OX=9606 PE=2 SV=1	0.472269609	0.377704871	0.094564738	0.013246127	0.0026658	0.942642955
P53597	Succinate--CoA ligase [ADP/GDP-forming] subunit alpha, mitochondrial OS=Homo sapiens OX=9606 GN=SUCLG1 PE=1 SV=4	-0.63433086	0.570645081	-1.204975941	0.013253087	0.004765687	5.9305E-06
Q6FHZ0	Malate dehydrogenase OS=Homo sapiens OX=9606 GN=MDH2 PE=2 SV=1	-0.536410283	0.580880784	-1.117291067	0.013266279	0.002955005	7.52521E-06

A0A024 R6I7	Alpha-1-antitrypsin OS=Homo sapiens OX=9606 GN=SERPINA1 PE=1 SV=1	1.18291031 7	- 0.05990 6753	1.242817 07	0.0133 11785	0.5681 2726	0.0031 46147
Q9BUR 5	MICOS complex subunit MIC26 OS=Homo sapiens OX=9606 GN=APOO PE=1 SV=1	- 0.55865976 6	0.14726 922	- 0.705928 986	0.0133 56479	0.6812 53461	0.0671 76845
P37837	Transaldolase OS=Homo sapiens OX=9606 GN=TALDO1 PE=1 SV=2	0.48228745 4	0.52360 2976	- 0.041315 522	0.0136 50665	0.0029 31062	0.4610 61955
H0YGS3	Microfibrillar-associated protein 5 (Fragment) OS=Homo sapiens OX=9606 GN=MFAP5 PE=1 SV=1	0.32596207 6	0.27823 7887	0.047724 189	0.0139 09786	0.0074 04873	0.8631 37252
B4E0R0	cDNA FLJ54220, highly similar to Long-chain-fatty-acid--CoA ligase 1 OS=Homo sapiens OX=9606 PE=2 SV=1	- 0.74539507 3	0.59703 6484	- 1.342431 557	0.0140 17728	0.0218 82664	0.0001 50861
P43155	Carnitine O-acetyltransferase OS=Homo sapiens OX=9606 GN=CRAT PE=1 SV=5	- 0.68196467 4	0.55062 1427	- 1.232586 101	0.0140 60114	0.0112 88682	2.8128 1E-05
A0A0U 1RQK4	[Protein ADP-ribosylarginine] hydrolase-like protein 1 OS=Homo sapiens OX=9606 GN=ADPRHL1 PE=1 SV=1	0.74036422 2	0.43474 3213	0.305621 009	0.0141 02918	0.0632 34092	0.1710 20776
P51970	NADH dehydrogenase [ubiquinone] 1 alpha subcomplex subunit 8 OS=Homo sapiens OX=9606 GN=NDUFA8 PE=1 SV=3	- 0.69126714 8	0.48667 1792	- 1.177938 94	0.0141 2128	0.0192 893	2.1592 7E-05
P13073	Cytochrome c oxidase subunit 4 isoform 1, mitochondrial OS=Homo sapiens OX=9606 GN=COX4I1 PE=1 SV=1	- 0.54934398 8	0.40284 6194	- 0.952190 182	0.0141 84572	0.0077 72435	2.7873 7E-05
H7C5H5	Mitofusin-1 (Fragment) OS=Homo sapiens OX=9606 GN=MFN1 PE=1 SV=1	- 0.90395397	0.51705 0475	- 1.421004 445	0.0142 19292	0.0731 14003	0.0003 23838

Q9NPP6	Immunoglobulin heavy chain variant (Fragment) OS=Homo sapiens OX=9606 PE=2 SV=1	1.263083093	0.006614383	1.25646871	0.014615168	0.958356671	0.008394229
A8K6Y1	cDNA FLJ75526, highly similar to Homo sapiens proliferation-associated 2G4, 38kDa (PA2G4), mRNA (Fragment) OS=Homo sapiens OX=9606 PE=2 SV=1	0.494414647	0.593849612	-0.099434965	0.01495874	0.006178955	0.589416937
A0A1B1HY12	Myomesin 1 variant 1 isoform a (Fragment) OS=Homo sapiens OX=9606 GN=MYOM1 PE=4 SV=1	0.722346517	0.861208914	-0.138862397	0.015016781	0.088838902	0.926384375
P01024	Complement C3 OS=Homo sapiens OX=9606 GN=C3 PE=1 SV=2	0.840368774	0.019689407	0.820679367	0.015085866	0.812202585	0.012373293
H0YE81	NADH dehydrogenase [ubiquinone] flavoprotein 1, mitochondrial (Fragment) OS=Homo sapiens OX=9606 GN=NDUFV1 PE=1 SV=1	-0.724764261	0.550391239	-1.2751555	0.015498164	0.004778339	5.93836E-06
B7ZAF6	Succinate--CoA ligase [ADP-forming] subunit beta, mitochondrial OS=Homo sapiens OX=9606 GN=SUCLA2 PE=2 SV=1	-0.559146615	0.572535832	-1.131682448	0.015689005	0.00433038	2.7105E-05
V9HW31	ATP synthase subunit beta OS=Homo sapiens OX=9606 GN=HEL-S-271 PE=1 SV=1	-0.416076038	0.399533904	-0.815609943	0.015720063	0.004221501	1.5433E-05
P02675	Fibrinogen beta chain OS=Homo sapiens OX=9606 GN=FGB PE=1 SV=2	1.104626698	0.401207715	0.703418983	0.015831178	0.003114742	0.119305675
Q8N3C0	Activating signal cointegrator 1 complex subunit 3 OS=Homo sapiens OX=9606 GN=ASCC3 PE=1 SV=3	0.73140708	0.914630516	-0.183223435	0.016001177	0.002262349	0.314215145

D6RTK6	Prune homolog 2 OS=Homo sapiens OX=9606 GN=PRUNE2 PE=2 SV=1	1.69664595	1.036865447	0.659780503	0.016072503	0.022060654	0.620544479
D3DP16	Fibrinogen gamma chain, isoform CRA_a OS=Homo sapiens OX=9606 GN=FGG PE=4 SV=1	1.198657366	0.135236495	1.063420871	0.016095382	0.206567346	0.032470412
A0A1W2PQS6	RPS10-NUDT3 readthrough OS=Homo sapiens OX=9606 GN=RPS10-NUDT3 PE=4 SV=1	0.502844362	0.318127801	0.184716561	0.016154498	0.018855675	0.705140029
O95139	NADH dehydrogenase [ubiquinone] 1 beta subcomplex subunit 6 OS=Homo sapiens OX=9606 GN=NDUFB6 PE=1 SV=3	-0.463273829	0.474750025	-0.938023854	0.016275556	0.00447585	2.50819E-06
H7C367	Non-POU domain-containing octamer-binding protein (Fragment) OS=Homo sapiens OX=9606 GN=NONO PE=1 SV=8	0.801533467	0.88501718	-0.083483714	0.016541616	0.002361642	0.405958948
Q5JXT2	Nucleolar protein 56 (Fragment) OS=Homo sapiens OX=9606 GN=NOP56 PE=1 SV=1	1.099932123	0.911291584	0.188640539	0.016610948	0.003474063	0.808274072
C9IYS5	Phytanoyl-CoA dioxygenase, peroxisomal (Fragment) OS=Homo sapiens OX=9606 GN=PHYH PE=1 SV=1	-0.552348319	0.34166364	-0.894011959	0.01671072	0.047845869	0.000251676
V9GYW0	Glucosamine-6-phosphate isomerase 2 (Fragment) OS=Homo sapiens OX=9606 GN=GNPDA2 PE=1 SV=1	0.379977913	0.444000972	-0.064023059	0.016745203	0.003128144	0.571124078
Q9HCL3	Zinc finger protein 14 homolog OS=Homo sapiens OX=9606 GN=ZFP14 PE=1 SV=2	0.491396617	0.732930982	-0.241534366	0.016756552	0.002475568	0.145754241

K7EME0	3-ketoacyl-CoA thiolase, mitochondrial OS=Homo sapiens OX=9606 GN=ACAA2 PE=1 SV=1	-0.524171788	0.54432261	-1.068494399	0.016835322	0.004907373	0.000136976
A0A024RDH6	SEC31-like 1 (S. cerevisiae), isoform CRA_b OS=Homo sapiens OX=9606 GN=SEC31L1 PE=4 SV=1	0.546978828	0.50316226	0.043816567	0.016864951	0.003962992	0.728661576
B4DEQ0	Electron transfer flavoprotein-ubiquinone oxidoreductase OS=Homo sapiens OX=9606 PE=2 SV=1	-0.505010606	0.112155764	-0.617166371	0.017244538	0.347141143	0.00957049
A0A384NPU5	Epididymis secretory sperm binding protein OS=Homo sapiens OX=9606 PE=2 SV=1	0.453531749	0.621553428	-0.168021679	0.017348985	0.00221293	0.110962225
B7Z9B8	cDNA FLJ56912, highly similar to Fibulin-2 OS=Homo sapiens OX=9606 PE=2 SV=1	0.945183261	0.723210186	0.221973075	0.01785664	0.011405004	0.702576104
F6KPG5	Albumin (Fragment) OS=Homo sapiens OX=9606 PE=2 SV=1	1.22714088	-0.196781359	1.423922239	0.018507375	0.184437663	0.001121039
Q6FHH6	LANCL1 protein (Fragment) OS=Homo sapiens OX=9606 GN=LANCL1 PE=2 SV=1	0.24064359	0.269673773	-0.029030183	0.018682763	0.012185193	0.594942896
Q8IWP6	Tubulin beta chain OS=Homo sapiens OX=9606 PE=2 SV=1	0.505565154	0.331478207	0.174086947	0.019146236	0.007796205	0.628357521
Q16778	Histone H2B type 2-E OS=Homo sapiens OX=9606 GN=H2BC21 PE=1 SV=3	0.431467662	0.388876029	0.042591632	0.019178738	0.023816705	0.675580292
Q16775	Hydroxyacylglutathione hydrolase, mitochondrial OS=Homo sapiens OX=9606 GN=HAGH PE=1 SV=2	0.261609703	0.184422493	0.07718721	0.019230626	0.147103415	0.499166545
O00571	ATP-dependent RNA helicase DDX3X OS=Homo sapiens OX=9606 GN=DDX3X PE=1 SV=3	0.492190827	0.534442726	-0.042251899	0.019236608	0.003968234	0.756974829

M1V490	KIF5B-RET(NM_020630)_K22R12 fusion protein OS=Homo sapiens OX=9606 GN=KIF5B-RET(NM_020630)_K22;R12 PE=2 SV=1	0.334179688	0.41122391	-0.077044222	0.019269338	0.003688194	0.346771574
A0A4D5RAJ5	Annexin OS=Homo sapiens OX=9606 PE=3 SV=1	0.66202005	0.644315612	0.017704437	0.019340547	0.0097615	0.602883918
Q16799	Reticulon-1 OS=Homo sapiens OX=9606 GN=RTN1 PE=1 SV=1	0.652411608	0.515919059	0.136492549	0.019556198	0.011276752	0.995970985
Q5T4U5	Acyl-Coenzyme A dehydrogenase, C-4 to C-12 straight chain, isoform CRA_a OS=Homo sapiens OX=9606 GN=ACADM PE=1 SV=1	-0.444782922	0.30934245	-0.754125372	0.019928637	0.063980179	0.003944582
B3KMK7	cDNA FLJ11291 fis, clone PLACE1009659, highly similar to Nck-associated protein 1 OS=Homo sapiens OX=9606 PE=2 SV=1	0.861498965	0.444919318	0.416579646	0.020073554	0.00774723	0.551204483
A0A140VK76	Testis secretory sperm-binding protein Li 203a OS=Homo sapiens OX=9606 PE=2 SV=1	-0.499000447	0.582637546	-1.081637993	0.020649757	0.004027828	6.60813E-06
G3V0I5	NADH dehydrogenase [ubiquinone] flavoprotein 1, mitochondrial OS=Homo sapiens OX=9606 GN=NDUFV1 PE=1 SV=1	-0.462241216	0.562109061	-1.024350278	0.020674472	0.003656316	1.10789E-05
A0A140VJG8	Catechol O-methyltransferase OS=Homo sapiens OX=9606 PE=2 SV=1	0.454884063	0.609277502	-0.154393439	0.020680403	0.002183166	0.17788172
A0A494C089	Zinc finger protein 283 OS=Homo sapiens OX=9606 GN=ZNF283 PE=1 SV=1	1.007696615	0.183381052	0.824315563	0.020785214	0.602560695	0.030078001
Q13011	Delta(3,5)-Delta(2,4)-dienoyl-CoA isomerase, mitochondrial OS=Homo	-0.473985069	0.629311988	-1.103297058	0.021171488	0.00528898	4.65258E-05

	sapiens OX=9606 GN=ECH1 PE=1 SV=2						
C9JB30	Microtubule-associated protein RP/EB family member 3 (Fragment) OS=Homo sapiens OX=9606 GN=MAPRE3 PE=1 SV=1	0.693858742	0.644024318	0.049834424	0.021409892	0.005459794	0.944836682
Q07954	Prolow-density lipoprotein receptor-related protein 1 OS=Homo sapiens OX=9606 GN=LRP1 PE=1 SV=2	0.718763653	0.566129356	0.152634297	0.021635979	0.006221393	0.9556506
Q00341	Vigilin OS=Homo sapiens OX=9606 GN=HDLBP PE=1 SV=2	0.697570951	0.972648731	-0.27507778	0.022022135	0.000408767	0.396722089
J3KN16	Proteasome adapter and scaffold protein ECM29 OS=Homo sapiens OX=9606 GN=ECPAS PE=1 SV=1	0.383312549	0.522501238	-0.139188688	0.022074512	0.006917343	0.354534599
E7ESB6	GRB10-interacting GYF protein 2 (Fragment) OS=Homo sapiens OX=9606 GN=GIGYF2 PE=1 SV=1	0.598360058	0.500649075	0.097710983	0.022102727	0.004238922	0.884319079
I3L3H2	Eukaryotic initiation factor 4A-III OS=Homo sapiens OX=9606 GN=EIF4A3 PE=1 SV=2	0.562293726	0.416402566	0.14589116	0.022536348	0.010995689	0.872216376
Q9NTK5	Obg-like ATPase 1 OS=Homo sapiens OX=9606 GN=OLA1 PE=1 SV=2	0.364001612	0.368079052	-0.004077439	0.022690083	0.00777327	0.949080842
P25705	ATP synthase subunit alpha, mitochondrial OS=Homo sapiens OX=9606 GN=ATP5F1A PE=1 SV=1	-0.389356088	0.442417535	-0.831773624	0.022778366	0.002422916	1.56044E-05
U6FIU7	Myoglobin (Fragment) OS=Homo sapiens OX=9606 GN=MB PE=2 SV=1	1.27734106	0.188138467	1.089202593	0.023292959	0.419980733	0.030806567
B3KU15	cDNA FLJ39063 fis, clone NT2RP7014348, highly similar to Homo sapiens	0.714815635	0.55700678	0.157808855	0.023436368	0.00778704	0.907578153

	transducer of regulated cAMP response element-binding protein (CREB) 2 (TORC2), mRNA OS=Homo sapiens OX=9606 PE=2 SV=1						
Q9NP98	Myozenin-1 OS=Homo sapiens OX=9606 GN=MYOZ1 PE=1 SV=1	-1.024401173	-0.611753033	-0.41264814	0.023459379	0.112158641	0.152298241
C9J1B5	Golgin subfamily A member 8B OS=Homo sapiens OX=9606 GN=GOLGA8B PE=4 SV=2	-1.723784981	-1.744868709	0.021083728	0.02356527	0.013372335	0.631699324
D6RF35	Vitamin D-binding protein OS=Homo sapiens OX=9606 GN=GC PE=1 SV=1	0.720305624	0.067716151	0.652589472	0.023702626	0.976312765	0.027660809
P26583	High mobility group protein B2 OS=Homo sapiens OX=9606 GN=HMGB2 PE=1 SV=2	0.853345978	0.521832001	0.331513977	0.023734754	0.040465751	0.243290348
A0A024R674	Spectrin beta chain OS=Homo sapiens OX=9606 GN=SPTB PE=3 SV=1	0.429443062	0.481169021	-0.051725959	0.024081297	0.004923842	0.589714703
I3L3Q4	Glyoxalase domain-containing protein 4 (Fragment) OS=Homo sapiens OX=9606 GN=GLOD4 PE=1 SV=1	0.499876194	0.112879205	0.38699699	0.024090321	0.102884442	0.175729601
A0A3B3ITK7	Phosphoglucomutase-1 OS=Homo sapiens OX=9606 GN=PGM1 PE=1 SV=1	0.366859951	0.477805222	-0.11094527	0.024113956	0.003708897	0.750046427
P01857	Immunoglobulin heavy constant gamma 1 OS=Homo sapiens OX=9606 GN=IGHG1 PE=1 SV=1	0.992098799	-0.161838902	1.153937701	0.024531828	0.323982814	0.002540047
Q5R372	Rab GTPase-activating protein 1-like OS=Homo sapiens OX=9606 GN=RABGAP1L PE=1 SV=1	0.539419094	0.523005298	0.016413796	0.024533521	0.001768864	0.596764009

E9PK25	Cofilin-1 OS=Homo sapiens OX=9606 GN=CFL1 PE=1 SV=1	0.422964627	-0.012306218	0.435270845	0.024623997	0.750340765	0.064844608
A0A024RB22	SWI/SNF related, matrix associated, actin dependent regulator of chromatin, subfamily c, member 2, isoform CRA_a OS=Homo sapiens OX=9606 GN=SMARCC2 PE=4 SV=1	0.789722348	0.95287283	-0.163150482	0.025036262	0.002765019	0.391581344
Q15910	Histone-lysine N-methyltransferase EZH2 OS=Homo sapiens OX=9606 GN=EZH2 PE=1 SV=2	0.497239768	-0.048481274	0.545721041	0.025071559	0.377157739	0.003269573
P07737	Profilin-1 OS=Homo sapiens OX=9606 GN=PFN1 PE=1 SV=2	0.514343189	0.230500814	0.283842375	0.025244514	0.011181711	0.421274849
E7EQB8	Isocitrate dehydrogenase [NAD] subunit, mitochondrial OS=Homo sapiens OX=9606 GN=IDH3G PE=1 SV=1	-0.512674049	0.636425004	-1.149099052	0.025387879	0.005498657	6.53058E-05
Q5UGI6	Serine/cysteine proteinase inhibitor clade G member 1 splice variant 2 (Fragment) OS=Homo sapiens OX=9606 GN=SERPING1 PE=2 SV=1	0.789390313	0.057173299	0.732217014	0.025828597	0.630507799	0.046400659
Q5U043	S-(hydroxymethyl)glutathione dehydrogenase OS=Homo sapiens OX=9606 PE=2 SV=1	0.575089332	0.454259236	0.120830095	0.025986663	0.005184333	0.959081395
D9IAI1	Epididymis secretory protein Li 34 OS=Homo sapiens OX=9606 GN=HEL-S-34 PE=2 SV=1	0.342061411	-0.134871227	0.476932638	0.026148658	0.168217483	0.000174586
P04198	N-myc proto-oncogene protein OS=Homo sapiens OX=9606 GN=MYCN PE=1 SV=2	0.945472264	0.634387985	0.311084279	0.026238787	0.004703435	0.766318866

Q9Y277	Voltage-dependent anion-selective channel protein 3 OS=Homo sapiens OX=9606 GN=VDAC3 PE=1 SV=1	-0.496087871	0.616893798	-1.11298167	0.026382781	0.003658792	3.31968E-05
Q0VG54	TNS1 protein OS=Homo sapiens OX=9606 GN=TNS1 PE=2 SV=1	0.42760835	0.567453411	-0.139845061	0.026456741	0.00314702	0.26124821
Q96HY7	Probable 2-oxoglutarate dehydrogenase E1 component DHKTD1, mitochondrial OS=Homo sapiens OX=9606 GN=DHTKD1 PE=1 SV=2	-0.532772998	0.605442262	-1.13821526	0.026802333	0.005214698	8.27189E-06
A0A5C2GC95	IGL c341_light_IGKV3D-20_IGKJ2 (Fragment) OS=Homo sapiens OX=9606 PE=2 SV=1	0.698399256	0.160103678	0.538295578	0.026937318	0.181273037	0.15618091
Q5T7C4	High mobility group protein B1 OS=Homo sapiens OX=9606 GN=HMGB1 PE=1 SV=1	0.502268432	0.603128629	-0.100860197	0.026958467	0.002478178	0.633121893
R4GMQ5	Cathepsin B (Fragment) OS=Homo sapiens OX=9606 GN=CTSB PE=1 SV=1	0.535376248	0.780607737	-0.24523149	0.026975735	0.00216714	0.157254475
U3KQK5	Uncharacterized protein OS=Homo sapiens OX=9606 PE=4 SV=1	-0.466673845	0.168722259	-0.635396104	0.027024164	0.035917708	0.000473318
V9HW96	Chaperonin containing TCP1, subunit 2 (Beta), isoform CRA_b OS=Homo sapiens OX=9606 GN=HEL-S-100n PE=1 SV=1	0.266646662	0.472411792	-0.20576513	0.027238401	0.002490933	0.095171795
H3BNT2	Ubiquinone biosynthesis protein (Fragment) OS=Homo sapiens OX=9606 GN=COQ9 PE=1 SV=8	-0.650507688	0.199265287	-0.849772975	0.027356184	0.278191158	0.016183529
V9HWC4	Epididymis secretory sperm binding protein Li 132P OS=Homo sapiens OX=9606 GN=HEL-S-132P PE=2 SV=1	0.309272833	-0.147851393	0.457124225	0.027390888	0.205164426	0.00348093

Q71UF1	Aconitate hydratase, mitochondrial OS=Homo sapiens OX=9606 GN=ACO2 PE=3 SV=1	-0.464211608	0.465898766	-0.930110374	0.027695902	0.010776925	0.000178053
J3KR44	Ubiquitin thioesterase OS=Homo sapiens OX=9606 GN=OTUB1 PE=1 SV=2	0.445906305	-0.071443572	0.517349877	0.027715971	0.173248906	0.001660154
B7Z9M9	cDNA, FLJ78893, highly similar to Destrin OS=Homo sapiens OX=9606 PE=2 SV=1	0.561785597	0.461450873	0.100334724	0.027799759	0.004525591	0.858280588
E9PF16	Medium-chain acyl-CoA ligase ACSF2, mitochondrial OS=Homo sapiens OX=9606 GN=ACSF2 PE=1 SV=1	-0.662516002	0.27318854	-0.935704541	0.02802623	0.04957396	0.003265099
F5H5P2	2-oxoisovalerate dehydrogenase subunit alpha OS=Homo sapiens OX=9606 PE=3 SV=1	-0.400528948	0.24566386	-0.646192808	0.028754258	0.16113634	0.016390339
A2A305	Ubiquitin-associated protein 2 (Fragment) OS=Homo sapiens OX=9606 GN=UBAP2 PE=1 SV=1	0.545900081	0.366393683	0.179506398	0.028840801	0.106667624	0.221449522
H0YL12	Electron transfer flavoprotein subunit alpha, mitochondrial (Fragment) OS=Homo sapiens OX=9606 GN=ETFA PE=1 SV=8	-0.696724503	0.536737408	-1.233461911	0.02889747	0.006282911	9.41534E-05
C9JBU9	Coiled-coil domain-containing protein 58 OS=Homo sapiens OX=9606 GN=CCDC58 PE=1 SV=1	-0.503635044	0.38293402	-0.886569064	0.029056883	0.078877742	0.0017978
A0A348GSI7	Guanylate-binding protein 2 (Fragment) OS=Homo sapiens OX=9606 GN=GBP2 PE=2 SV=1	0.559618277	0.333705633	0.225912644	0.029136797	0.012438736	0.943129721
A0A024RAC7	Multifunctional fusion protein OS=Homo sapiens OX=9606 GN=ALDH4A1 PE=2 SV=1	-0.279953595	0.41379518	-0.693748775	0.02930154	0.00442196	6.81989E-05

E5KLM0	Mitochondrial dynamin-like 120 kDa protein OS=Homo sapiens OX=9606 PE=3 SV=1	-0.304623595	0.436631396	-0.741254991	0.029411836	0.004692756	2.27616E-05
P36507	Dual specificity mitogen-activated protein kinase kinase 2 OS=Homo sapiens OX=9606 GN=MAP2K2 PE=1 SV=1	0.430223508	0.263582851	0.166640657	0.029446274	0.01544116	0.592902714
A0A024RB75	Citrate synthase OS=Homo sapiens OX=9606 GN=CS PE=3 SV=1	-0.438489299	0.54748703	-0.985976328	0.029713231	0.00436958	3.33863E-05
B7Z6Y2	cDNA FLJ54942, highly similar to Homo sapiens bridging integrator 1 (BIN1), transcript variant 10, mRNA OS=Homo sapiens OX=9606 PE=2 SV=1	0.523391834	0.633803589	-0.110411755	0.029723627	0.022337867	0.942574858
A0A1U9X8J2	SKIV2L OS=Homo sapiens OX=9606 PE=4 SV=1	0.44406207	0.611760219	-0.167698149	0.029803837	0.004927737	0.24416299
A0A384NKJ3	Epididymis secretory sperm binding protein OS=Homo sapiens OX=9606 PE=2 SV=1	-0.542282414	0.423646996	-0.96592941	0.029955296	0.004560069	6.65072E-05
D6RF44	Heterogeneous nuclear ribonucleoprotein D0 (Fragment) OS=Homo sapiens OX=9606 GN=HNRNPD PE=1 SV=8	0.580983269	0.479652806	0.101330463	0.030000877	0.008056735	0.829372834
E9PNR6	Rho GTPase-activating protein 1 (Fragment) OS=Homo sapiens OX=9606 GN=ARHGAP1 PE=1 SV=1	0.606320716	0.458274734	0.148045982	0.030078474	0.007782455	0.958793346
Q96EN8	Molybdenum cofactor sulfurase OS=Homo sapiens OX=9606 GN=MOCOS PE=1 SV=2	-0.793551445	-0.756088319	-0.037463126	0.030146716	0.131046064	0.577780114
O95661	GTP-binding protein Di-Ras3 OS=Homo sapiens OX=9606 GN=DIRAS3 PE=1 SV=1	0.76837889	0.997922447	-0.229543557	0.030192792	0.004603318	0.251239474

P17568	NADH dehydrogenase [ubiquinone] 1 beta subcomplex subunit 7 OS=Homo sapiens OX=9606 GN=NDUFB7 PE=1 SV=4	-0.360203613	-0.007132045	-0.353071568	0.030341828	0.468924792	0.211146394
Q96BW5	Phosphotriesterase-related protein OS=Homo sapiens OX=9606 GN=PTER PE=1 SV=1	0.341548091	0.510800695	-0.169252604	0.030388145	0.002382432	0.138698606
P82909	28S ribosomal protein S36, mitochondrial OS=Homo sapiens OX=9606 GN=MRPS36 PE=1 SV=2	-0.367992814	0.262557657	-0.630550471	0.030389662	0.026463577	8.36809E-05
Q53HG1	NADH dehydrogenase [ubiquinone] 1 alpha subcomplex subunit 12 (Fragment) OS=Homo sapiens OX=9606 PE=2 SV=1	-0.336090652	0.392102395	-0.728193048	0.03061512	0.003080529	8.41679E-06
Q04760	Lactoylglutathione lyase OS=Homo sapiens OX=9606 GN=GLO1 PE=1 SV=4	0.307722908	0.144878675	0.162844233	0.030728537	0.287972482	0.497499501
Q69YU5	Uncharacterized protein C12orf73 OS=Homo sapiens OX=9606 GN=C12orf73 PE=3 SV=2	-0.701242539	0.365295816	-1.066538355	0.030894469	0.050557109	0.000739812
E9PE82	Short-chain-specific acyl-CoA dehydrogenase, mitochondrial OS=Homo sapiens OX=9606 GN=ACADS PE=1 SV=1	-0.611285261	0.611658906	-1.222944167	0.030961547	0.008498036	1.60873E-05
Q02978	Mitochondrial 2-oxoglutarate/malate carrier protein OS=Homo sapiens OX=9606 GN=SLC25A11 PE=1 SV=3	-0.466063388	0.405924112	-0.871987501	0.031009143	0.016382275	0.000147942
Q06830	Peroxiredoxin-1 OS=Homo sapiens OX=9606 GN=PRDX1 PE=1 SV=1	0.448884204	0.211343395	0.23754081	0.03102053	0.093255578	0.41063564

Q04828	Aldo-keto reductase family 1 member C1 OS=Homo sapiens OX=9606 GN=AKR1C1 PE=1 SV=1	0.825060732	0.409952444	0.415108288	0.031155187	0.015968979	0.489851752
A0A2P9DU05	Rho-associated protein kinase OS=Homo sapiens OX=9606 GN=ROCK2 PE=2 SV=1	0.691930416	0.728244942	-0.036314526	0.031498618	0.003127414	0.686050511
O00159	Unconventional myosin-Ic OS=Homo sapiens OX=9606 GN=MYO1C PE=1 SV=4	0.607571915	0.496048354	0.111523561	0.031940982	0.017022428	0.724883241
P55072	Transitional endoplasmic reticulum ATPase OS=Homo sapiens OX=9606 GN=VCP PE=1 SV=4	0.325267929	0.494827272	-0.169559343	0.032359412	0.003950115	0.197625306
B2RCT6	cDNA, FLJ96276, highly similar to Homo sapiens G1 to S phase transition 1 (GSPT1), mRNA OS=Homo sapiens OX=9606 PE=2 SV=1	0.329882684	0.425133057	-0.095250374	0.032832995	0.002398638	0.419177044
Q1RMC9	ERBB2IP protein OS=Homo sapiens OX=9606 GN=ERBB2IP PE=2 SV=1	0.548911806	0.778534008	-0.229622201	0.03408877	0.003121978	0.196313617
A0A024R2Q5	Myosin, light polypeptide 3, alkali ventricular, skeletal, slow, isoform CRA_a OS=Homo sapiens OX=9606 GN=MYL3 PE=4 SV=1	-0.511718126	-0.247397586	-0.26432054	0.034138986	0.243259914	0.1354922
E5RIT6	60S ribosomal protein L26-like 1 (Fragment) OS=Homo sapiens OX=9606 GN=RPL26L1 PE=1 SV=1	0.648548451	0.3509441	0.297604351	0.034222984	0.041992424	0.723636538
O75323	Protein NipSnap homolog 2 OS=Homo sapiens OX=9606 GN=NIPSNAP2 PE=1 SV=1	-0.451947339	0.471700262	-0.923647601	0.034308154	0.065753308	0.002234139

Q00169	Phosphatidylinositol transfer protein alpha isoform OS=Homo sapiens OX=9606 GN=PITPNA PE=1 SV=2	0.39022935	0.545973825	-0.155744475	0.034389029	0.003475573	0.247376459
B0YJ81	Very-long-chain (3R)-3-hydroxyacyl-CoA dehydratase 1 OS=Homo sapiens OX=9606 GN=HACD1 PE=1 SV=1	0.325750268	0.375523016	-0.049772748	0.034407457	0.012122648	0.714980187
P02768	Serum albumin OS=Homo sapiens OX=9606 GN=ALB PE=1 SV=2	0.912314963	-0.257366913	1.169681876	0.034451712	0.209195023	0.000928983
P21333	Filamin-A OS=Homo sapiens OX=9606 GN=FLNA PE=1 SV=4	0.543812191	0.547406536	-0.003594345	0.034530767	0.007731392	0.519612813
H0Y8G5	Heterogeneous nuclear ribonucleoprotein D0 (Fragment) OS=Homo sapiens OX=9606 GN=HNRNPD PE=1 SV=8	0.620297418	0.77547847	-0.155181052	0.034995526	0.002386872	0.27314257
A0A096LPI3	Putative bifunctional UDP-N-acetylglucosamine transferase and deubiquitinase ALG13 (Fragment) OS=Homo sapiens OX=9606 GN=ALG13 PE=1 SV=1	-1.137007394	-2.40143419	1.264426796	0.035094431	0.001392856	0.114631649
Q8TAT6	Nuclear protein localization protein 4 homolog OS=Homo sapiens OX=9606 GN=NPLOC4 PE=1 SV=3	0.355267258	0.545365337	-0.190098079	0.035734966	0.004769177	0.25969226
B4DN60	cDNA FLJ52703, highly similar to Asparaginyl-tRNA synthetase, cytoplasmic (EC6.1.1.22) OS=Homo sapiens OX=9606 PE=2 SV=1	0.653450426	0.68925852	-0.035808094	0.036063381	0.001379301	0.768383171
P49753	Acyl-coenzyme A thioesterase 2, mitochondrial OS=Homo sapiens OX=9606 GN=ACOT2 PE=1 SV=6	-0.394599968	0.196000893	-0.590600861	0.036106328	0.052222464	0.000245148

A5PLL0	CPT1B protein OS=Homo sapiens OX=9606 GN=CPT1B PE=2 SV=1	-0.474660013	0.440319414	-0.914979427	0.036219895	0.010835028	0.00011737
A0A384MR25	Epididymis secretory sperm binding protein OS=Homo sapiens OX=9606 PE=2 SV=1	0.428976054	0.727405224	-0.29842917	0.036558599	0.002281942	0.077501118
Q02218	2-oxoglutarate dehydrogenase, mitochondrial OS=Homo sapiens OX=9606 GN=OGDH PE=1 SV=3	-0.426249396	0.596183615	-1.022433011	0.036570552	0.004529352	4.58958E-05
P07585	Decorin OS=Homo sapiens OX=9606 GN=DCN PE=1 SV=1	0.660684089	1.102471789	-0.441787699	0.037151599	0.001329803	0.081602406
A0A024RBX6	Pirin (Iron-binding nuclear protein), isoform CRA_a OS=Homo sapiens OX=9606 GN=PIR PE=3 SV=1	0.398941693	0.473914305	-0.074972612	0.037251654	0.007432856	0.407390146
A0A024R396	Chromosome 11 open reading frame 54, isoform CRA_a OS=Homo sapiens OX=9606 GN=C11orf54 PE=4 SV=1	0.344405159	0.117060685	0.227344474	0.037475495	0.64079215	0.09886465
Q8IX21	SMC5-SMC6 complex localization factor protein 2 OS=Homo sapiens OX=9606 GN=SLF2 PE=1 SV=2	1.771099151	0.271846119	1.499253031	0.037779729	0.201272764	0.089526949
Q13564	NEDD8-activating enzyme E1 regulatory subunit OS=Homo sapiens OX=9606 GN=NAE1 PE=1 SV=1	0.313646978	0.350340809	-0.036693831	0.038022253	0.018143513	0.707298948
Q9UIJ7	GTP:AMP phosphotransferase AK3, mitochondrial OS=Homo sapiens OX=9606 GN=AK3 PE=1 SV=4	-0.690602626	0.596083298	-1.286685924	0.038051898	0.006567918	9.28695E-05
Q9H2R7	NPD011 OS=Homo sapiens OX=9606 GN=NPD011 PE=2 SV=1	0.278346221	0.289716814	-0.011370593	0.040087075	0.001391451	0.51969151

Q53G34	Mitochondrial carrier homolog 2 variant (Fragment) OS=Homo sapiens OX=9606 PE=2 SV=1	-0.463283511	0.619177159	-1.082460669	0.040178661	0.003458228	1.44454E-05
A0A0S2Z3W7	Nucleotide diphosphatase (Fragment) OS=Homo sapiens OX=9606 GN=ITPA PE=2 SV=1	0.637930323	0.186897961	0.451032362	0.040185488	0.199337599	0.327775058
K7EQL4	Troponin T, slow skeletal muscle (Fragment) OS=Homo sapiens OX=9606 GN=TNNT1 PE=1 SV=1	-0.790785348	-0.847700883	0.056915534	0.041464514	0.013181049	0.948210811
P36269	Glutathione hydrolase 5 proenzyme OS=Homo sapiens OX=9606 GN=GGT5 PE=1 SV=2	0.831932053	0.71030878	0.121623273	0.041906705	0.008401491	0.80737898
Q9C063	LYST-interacting protein LIP5 (Fragment) OS=Homo sapiens OX=9606 PE=2 SV=1	0.480544741	0.272456456	0.208088285	0.04217822	0.030658902	0.64928921
P02760	Protein AMBP OS=Homo sapiens OX=9606 GN=AMBP PE=1 SV=1	1.13718131	0.56157272	0.575608591	0.042310989	0.008784278	0.440047647
A0A5C2GQA2	IG c556_heavy_IGHV5-51_IGHD6-19_IGHJ2 (Fragment) OS=Homo sapiens OX=9606 PE=2 SV=1	1.203230815	-0.355505604	1.558736419	0.042939681	0.275249299	0.00878481
B7ZLW0	LPP protein OS=Homo sapiens OX=9606 GN=LPP PE=2 SV=1	0.35354943	0.637887636	-0.284338206	0.043009106	0.003101348	0.082106986
P35080	Profilin-2 OS=Homo sapiens OX=9606 GN=PFN2 PE=1 SV=3	0.435762182	0.065933732	0.36982845	0.043014808	0.818525799	0.021585668
P28838	Cytosol aminopeptidase OS=Homo sapiens OX=9606 GN=LAP3 PE=1 SV=3	0.42292841	0.338527326	0.084401084	0.043015424	0.012411085	0.887883292
Q9Y4G6	Talin-2 OS=Homo sapiens OX=9606 GN=TLN2 PE=1 SV=4	0.378986618	0.654312068	-0.27532545	0.043036558	0.004517754	0.166326888
A8MXP9	Matrin-3 OS=Homo sapiens OX=9606 GN=MATR3 PE=1 SV=1	0.46800097	0.562365718	-0.094364748	0.043185832	0.002279835	0.403113824

Q09028	Histone-binding protein RBBP4 OS=Homo sapiens OX=9606 GN=RBBP4 PE=1 SV=3	0.346908646	0.416991012	-0.070082366	0.043802158	0.003987872	0.529700633
A0A3B3ITJ4	Heterogeneous nuclear ribonucleoprotein L (Fragment) OS=Homo sapiens OX=9606 GN=HNRNPL PE=1 SV=1	0.643213257	0.747504381	-0.104291125	0.044005281	0.002380498	0.345168227
P10644	cAMP-dependent protein kinase type I-alpha regulatory subunit OS=Homo sapiens OX=9606 GN=PRKAR1A PE=1 SV=1	0.49486343	0.481005855	0.013857575	0.044162298	0.008867199	0.720686498
J3KQN4	60S ribosomal protein L36a OS=Homo sapiens OX=9606 GN=RPL36A PE=1 SV=1	-0.792533579	-0.080782923	-0.711750655	0.04427208	0.569640839	0.032376937
F8W036	Uncharacterized protein OS=Homo sapiens OX=9606 PE=4 SV=1	0.615909826	0.866224052	-0.250314226	0.044409595	0.002777893	0.203868967
Q9BTV4	Transmembrane protein 43 OS=Homo sapiens OX=9606 GN=TMEM43 PE=1 SV=1	0.354866775	0.599623872	-0.244757097	0.044526344	0.004847062	0.111129687
Q9Y5P6	Mannose-1-phosphate guanyltransferase beta OS=Homo sapiens OX=9606 GN=GMPPB PE=1 SV=2	0.485757783	0.429995131	0.055762652	0.045390543	0.0354787 1	0.919649588
A0A2U3TZM0	Chromodomain-helicase-DNA-binding protein 4 OS=Homo sapiens OX=9606 GN=CHD4 PE=1 SV=1	0.603046505	0.520367843	0.082678662	0.047398155	0.019144423	0.86778244
E7EMG9	Lymphocyte-specific protein 1 (Fragment) OS=Homo sapiens OX=9606 GN=LSP1 PE=1 SV=1	0.582870062	0.452203878	0.130666184	0.047457951	0.010421461	0.726456524
B3KVX6	cDNA FLJ41699 fis, clone HCHON2004776, highly similar to Homo sapiens cytoskeleton-associated protein 4 (CKAP4), mRNA OS=Homo	0.539453641	0.554264369	-0.014810728	0.047568865	0.004435118	0.419867964

	sapiens OX=9606 PE=2 SV=1						
Q9Y490	Talin-1 OS=Homo sapiens OX=9606 GN=TLN1 PE=1 SV=3	0.425511994	0.822016818	-0.396504824	0.047849911	0.002203074	0.05218803
A0A024RAN2	Calpastatin, isoform CRA_a OS=Homo sapiens OX=9606 GN=CAST PE=4 SV=1	0.578165406	0.809292348	-0.231126942	0.047870047	0.003509882	0.21268154
B4DW52	cDNA FLJ55253, highly similar to Actin, cytoplasmic 1 OS=Homo sapiens OX=9606 PE=2 SV=1	-0.603481823	-0.467003648	-0.136478174	0.047994584	0.542642388	0.3381495
V9HWK1	Triosephosphate isomerase OS=Homo sapiens OX=9606 GN=HEL-S-49 PE=2 SV=1	0.357262629	0.301504657	0.055757972	0.048487572	0.064096107	0.515764461
A0A0A0MS01	T cell receptor gamma variable 10 (non-functional) (Fragment) OS=Homo sapiens OX=9606 GN=TRGV10 PE=4 SV=1	-0.497304366	0.733182159	-1.230486525	0.049349221	0.002758093	3.0922E-05
F5GXS0	Complement C4-B OS=Homo sapiens OX=9606 GN=C4B PE=1 SV=1	0.892163359	-0.051234665	0.943398024	0.049776275	0.777266959	0.030014013
O60664	Perilipin-3 OS=Homo sapiens OX=9606 GN=PLIN3 PE=1 SV=3	0.587777793	0.334100468	0.253677325	0.049814874	0.112349854	0.396113139
P35580	Myosin-10 OS=Homo sapiens OX=9606 GN=MYH10 PE=1 SV=3	0.597835204	0.640216138	-0.042380934	0.050469025	0.003787727	0.403369682
A0A024R694	Actinin, alpha 1, isoform CRA_a OS=Homo sapiens OX=9606 GN=ACTN1 PE=2 SV=1	0.52395799	0.611171769	-0.087213778	0.050574738	0.011182721	0.387752026
A0A024R5X2	HCG2001986, isoform CRA_a OS=Homo sapiens OX=9606 GN=hCG_2001986 PE=4 SV=1	0.368931121	0.276772459	0.092158662	0.050719146	0.018844539	0.871808303
O75489	NADH dehydrogenase [ubiquinone] iron-sulfur protein 3, mitochondrial OS=Homo sapiens	-0.41630985	0.303608433	-0.719918283	0.050965965	0.022478371	0.000321911

	OX=9606 GN=NDUFS3 PE=1 SV=1						
A0A0A0MSB7	Calcium-binding protein 8 OS=Homo sapiens OX=9606 GN=CALN1 PE=4 SV=1	-0.849154369	0.582527353	-1.431681722	0.050972212	0.031998852	0.000498926
Q13643	Four and a half LIM domains protein 3 OS=Homo sapiens OX=9606 GN=FHL3 PE=1 SV=4	-0.388125514	-0.281020025	-0.107105489	0.051009031	0.301316237	0.434107174
Q5T948	Serine/threonine-protein phosphatase 2A activator (Fragment) OS=Homo sapiens OX=9606 GN=PTPA PE=1 SV=8	0.313388393	0.068751736	0.244636657	0.051167951	0.310939029	0.273272238
Q9H2G2	STE20-like serine/threonine-protein kinase OS=Homo sapiens OX=9606 GN=SLK PE=1 SV=1	0.447697556	0.799550657	-0.351853101	0.053337137	0.00133923	0.043831602
A6NGQ3	Obscurin OS=Homo sapiens OX=9606 GN=OBSCN PE=1 SV=5	0.540462179	0.962184387	-0.421722208	0.053345479	0.004014739	0.166231732
B7Z2F5	cDNA FLJ55918, highly similar to Echinoderm microtubule-associated protein-like 2 OS=Homo sapiens OX=9606 PE=2 SV=1	0.417257906	0.039526697	0.37773121	0.053455289	0.46227268	0.314574557
P56192	Methionine--tRNA ligase, cytoplasmic OS=Homo sapiens OX=9606 GN=MARS1 PE=1 SV=2	0.418703651	0.625789878	-0.207086227	0.05349819	0.001475621	0.100432476
Q63HR2	Tensin-2 OS=Homo sapiens OX=9606 GN=TNS2 PE=1 SV=2	0.391375481	0.455086909	-0.063711428	0.053941821	0.022551486	0.54139359
P12270	Nucleoprotein TPR OS=Homo sapiens OX=9606 GN=TPR PE=1 SV=3	0.560647789	0.537124171	0.023523618	0.053960232	0.020437761	0.635815972
O75643	U5 small nuclear ribonucleoprotein 200 kDa helicase OS=Homo	0.555668737	0.640166141	-0.084497405	0.053982765	0.003673377	0.396506918

	sapiens OX=9606 GN=SNRNP200 PE=1 SV=2						
P30048	Thioredoxin-dependent peroxide reductase, mitochondrial OS=Homo sapiens OX=9606 GN=PRDX3 PE=1 SV=3	-0.422919996	0.506941045	-0.929861041	0.054048124	0.006689065	3.3199E-05
Q5TA01	Glutathione S-transferase omega-1 (Fragment) OS=Homo sapiens OX=9606 GN=GSTO1 PE=1 SV=1	0.393135871	0.258752468	0.134383403	0.054494943	0.037342849	0.737273048
V9HWB5	Epididymis secretory sperm binding protein Li 66p OS=Homo sapiens OX=9606 GN=HEL-S-66p PE=2 SV=1	0.477783034	0.424146686	0.053636347	0.054508232	0.004408443	0.617215837
A3RJH1	ATP-dependent RNA helicase DDX1 OS=Homo sapiens OX=9606 GN=DDX1 PE=2 SV=1	0.369517974	0.685986914	-0.31646894	0.055099041	0.001775256	0.034986056
P56378	ATP synthase subunit ATP5MPL, mitochondrial OS=Homo sapiens OX=9606 GN=ATP5MPL PE=1 SV=1	-0.386711806	0.316605791	-0.703317597	0.055119897	0.029694685	0.000629866
A0A087WTB8	Ubiquitin carboxyl-terminal hydrolase OS=Homo sapiens OX=9606 GN=UCHL3 PE=1 SV=1	0.397339251	0.049148053	0.348191198	0.05519097	0.658647912	0.094861574
A0A140VJZ1	Ubiquitin carboxyl-terminal hydrolase OS=Homo sapiens OX=9606 PE=2 SV=1	0.4254527	0.35224732	0.07320538	0.055231267	0.019321711	0.942372003
Q9H993	Damage-control phosphatase ARMT1 OS=Homo sapiens OX=9606 GN=ARMT1 PE=1 SV=1	0.298159742	0.136630432	0.161529311	0.055288669	0.19217471	0.377455797
F6WLT2	Spliceosome RNA helicase DDX39B (Fragment) OS=Homo sapiens OX=9606 GN=DDX39B PE=1 SV=1	0.436680334	0.578428498	-0.141748163	0.055318521	0.004216282	0.292081781

K7ELW0	Protein/nucleic acid deglycase DJ-1 OS=Homo sapiens OX=9606 GN=PARK7 PE=1 SV=1	0.279364158	0.042686851	0.236677307	0.055367276	0.734188646	0.195042449
B2R9K8	cDNA, FLJ94440, highly similar to Homo sapiens chaperonin containing TCP1, subunit 6A (zeta 1)(CCT6A), mRNA OS=Homo sapiens OX=9606 PE=2 SV=1	0.261944376	0.367605581	-0.105661205	0.055393077	0.003400901	0.195754107
O95210	Starch-binding domain-containing protein 1 OS=Homo sapiens OX=9606 GN=STBD1 PE=1 SV=1	0.52915494	0.798897432	-0.269742492	0.055951421	0.003157662	0.275614635
B3KP25	cDNA FLJ31014 fis, clone HLUNG2000255, highly similar to Protease serine 27 OS=Homo sapiens OX=9606 PE=2 SV=1	1.215086225	-0.270017734	1.485103959	0.056114841	0.337706379	0.006413807
A0A087WXS7	ATPase ASNA1 OS=Homo sapiens OX=9606 GN=ASNA1 PE=1 SV=1	0.364232064	0.35299669	0.011235373	0.056199617	0.014942289	0.739727672
P0DOX7	Immunoglobulin kappa light chain OS=Homo sapiens OX=9606 PE=1 SV=1	0.805462818	-0.224278342	1.02974116	0.056201399	0.620771016	0.046374436
Q8TAG3	Anion exchange protein OS=Homo sapiens OX=9606 GN=SLC4A2 PE=2 SV=1	0.463091256	0.74931389	-0.286222634	0.056287758	0.002165989	0.081131159
E9PFZ2	Ceruloplasmin OS=Homo sapiens OX=9606 GN=CP PE=1 SV=1	0.843846584	0.013812726	0.830033858	0.056603637	0.95799802	0.069939863
P05026	Sodium/potassium-transporting ATPase subunit beta-1 OS=Homo sapiens OX=9606 GN=ATP1B1 PE=1 SV=1	0.469221246	0.435726959	0.033494287	0.056642679	0.127666395	0.702931514

Q969X5	Endoplasmic reticulum-Golgi intermediate compartment protein 1 OS=Homo sapiens OX=9606 GN=ERGIC1 PE=1 SV=1	0.290198757	0.433734585	-0.143535828	0.056645018	0.020054715	0.25245161
Q5T7N2	LINE-1 type transposase domain-containing protein 1 OS=Homo sapiens OX=9606 GN=L1TD1 PE=1 SV=1	1.157438755	0.00643938	1.150999375	0.056691593	0.802783953	0.020443089
Q9BV79	Enoyl-[acyl-carrier-protein] reductase, mitochondrial OS=Homo sapiens OX=9606 GN=MECR PE=1 SV=2	-0.341308819	0.503986048	-0.845294867	0.056703574	0.009140542	4.65864E-05
Q13867	Bleomycin hydrolase OS=Homo sapiens OX=9606 GN=BLMH PE=1 SV=1	0.169100229	0.331378668	-0.162278438	0.056762937	0.006729403	0.080752923
A0A087X2I1	26S proteasome regulatory subunit 10B OS=Homo sapiens OX=9606 GN=PSMC6 PE=1 SV=1	0.223233751	0.467564567	-0.244330816	0.056810849	0.002255012	0.064490321
A0A384NYP3	Epididymis secretory sperm binding protein OS=Homo sapiens OX=9606 PE=2 SV=1	0.51069008	-0.360137691	0.870827771	0.056955401	0.261344801	0.023499145
Q15772	Striated muscle preferentially expressed protein kinase OS=Homo sapiens OX=9606 GN=SPEG PE=1 SV=4	0.376624192	0.741273623	-0.364649431	0.057044834	0.006674322	0.149006089
A0A2U3TZL5	CD59 glycoprotein (Fragment) OS=Homo sapiens OX=9606 GN=CD59 PE=1 SV=1	0.470330394	0.705018511	-0.234688117	0.057071341	0.011001546	0.467120213
P63316	Troponin C, slow skeletal and cardiac muscles OS=Homo sapiens OX=9606 GN=TNNC1 PE=1 SV=1	-0.869179183	-0.631410352	-0.23776883	0.057179466	0.292399097	0.23725213
B7Z5N7	cDNA FLJ58612, highly similar to Sec1 family domain-containing	0.382772723	0.572089098	-0.189316375	0.0572203	0.001831299	0.122801939

	protein 1 OS=Homo sapiens OX=9606 PE=2 SV=1						
E7EQ69	N-alpha-acetyltransferase 50 OS=Homo sapiens OX=9606 GN=NAA50 PE=1 SV=1	0.370571963	0.397470779	-0.026898816	0.057565997	0.006713293	0.582088056
A0A024R895	SET translocation (Myeloid leukemia-associated), isoform CRA_b OS=Homo sapiens OX=9606 GN=SET PE=3 SV=1	0.53121799	0.759904659	-0.228686669	0.057768075	0.003989271	0.207802766
A0A0G2JQJ7	Microtubule-associated protein OS=Homo sapiens OX=9606 GN=MAPT PE=1 SV=1	0.530599201	0.752251968	-0.221652768	0.057775726	0.002906481	0.425218262
A0A024R1N1	Myosin, heavy polypeptide 9, non-muscle, isoform CRA_a OS=Homo sapiens OX=9606 GN=MYH9 PE=3 SV=1	0.437285643	0.258590593	0.17869505	0.057805949	0.011862729	0.950383117
V9HW90	Glutathione reductase OS=Homo sapiens OX=9606 GN=HEL-75 PE=2 SV=1	0.49637881	0.244045169	0.252333641	0.058428109	0.106690691	0.527685528
A0A024RA42	Basic leucine zipper and W2 domains 2, isoform CRA_a OS=Homo sapiens OX=9606 GN=BZW2 PE=4 SV=1	0.767340593	0.244038666	0.523301927	0.058563989	0.443746139	0.140712514
A0A024R1Z6	Vesicle amine transport protein 1 homolog (T californica), isoform CRA_a OS=Homo sapiens OX=9606 GN=VAT1 PE=4 SV=1	0.550574001	0.5781391	-0.027565099	0.059221814	0.003332896	0.513176006
P10599	Thioredoxin OS=Homo sapiens OX=9606 GN=TXN PE=1 SV=3	0.556003465	-0.243285228	0.799288693	0.059334887	0.179458447	0.005476861
Q9UII2	ATPase inhibitor, mitochondrial OS=Homo sapiens OX=9606 GN=ATP5IF1 PE=1 SV=1	-0.57613993	0.154419349	-0.730559278	0.059345698	0.090754395	0.005093878

D6RD47	40S ribosomal protein S23 OS=Homo sapiens OX=9606 GN=RPS23 PE=1 SV=1	0.399055658	0.528495657	-0.12944	0.059448361	0.00456036	0.336200322
Q8IUZ5	5-phosphohydroxy-L-lysine phospho-lyase OS=Homo sapiens OX=9606 GN=PHYKPL PE=1 SV=1	0.265177558	0.22783479	0.037342768	0.059460959	0.101751001	0.927451468
B7Z2B0	cDNA FLJ53470, highly similar to Calcium/calmodulin-dependent protein kinase type II delta chain OS=Homo sapiens OX=9606 PE=2 SV=1	0.478787491	0.525135691	-0.0463482	0.059497892	0.020043317	0.907299057
B4DXI1	cDNA FLJ54333, highly similar to T-complex protein 1 subunit epsilon OS=Homo sapiens OX=9606 PE=2 SV=1	0.275808165	0.24486222	0.030945946	0.05966381	0.019542409	0.759103862
H0YDE4	Protein arginine N-methyltransferase 1 (Fragment) OS=Homo sapiens OX=9606 GN=PRMT1 PE=1 SV=1	0.498564534	-0.132025071	0.630589605	0.05972019	0.172112292	0.004487177
B4E1J8	cDNA FLJ56285, highly similar to ADP-ribosylation factor-like protein 8B OS=Homo sapiens OX=9606 PE=2 SV=1	0.386558623	0.551120315	-0.164561692	0.059728361	0.007445243	0.244309128
Q02952	A-kinase anchor protein 12 OS=Homo sapiens OX=9606 GN=AKAP12 PE=1 SV=4	0.518674335	0.689749063	-0.171074729	0.059832239	0.006458775	0.277600718
P62253	Ubiquitin-conjugating enzyme E2 G1 OS=Homo sapiens OX=9606 GN=UBE2G1 PE=1 SV=3	0.436394482	0.097025797	0.339368686	0.06065687	0.168065888	0.39882006
Q13784	APOA4 protein (Fragment) OS=Homo sapiens OX=9606 GN=APOA4 PE=2 SV=1	0.650230898	0.00221196	0.648018938	0.060873503	0.932596869	0.082279844

P21796	Voltage-dependent anion-selective channel protein 1 OS=Homo sapiens OX=9606 GN=VDAC1 PE=1 SV=2	-0.469917554	0.359292864	-0.829210418	0.061027475	0.027874746	0.000982543
A0A087WY61	Nuclear mitotic apparatus protein 1 OS=Homo sapiens OX=9606 GN=NUMA1 PE=1 SV=1	0.508644479	0.549549321	-0.040904842	0.061070543	0.021549485	0.461226678
P04003	C4b-binding protein alpha chain OS=Homo sapiens OX=9606 GN=C4BPA PE=1 SV=2	0.632374711	0.099055346	0.533319365	0.061800163	0.347268918	0.23254013
Q6TFL3	Coiled-coil domain-containing protein 171 OS=Homo sapiens OX=9606 GN=CCDC171 PE=2 SV=1	-0.604607299	-0.386358674	-0.218248625	0.06192046	0.582522282	0.232839464
P19387	DNA-directed RNA polymerase II subunit RPB3 OS=Homo sapiens OX=9606 GN=POLR2C PE=1 SV=2	0.388469304	0.60009434	-0.211625036	0.063382712	0.001187419	0.105667286
Q9NQW7	Xaa-Pro aminopeptidase 1 OS=Homo sapiens OX=9606 GN=XPNPEP1 PE=1 SV=3	0.259661923	0.525998056	-0.266336133	0.063773391	0.002589772	0.056265234
V9HWF4	Phosphoglycerate kinase OS=Homo sapiens OX=9606 GN=HEL-S-68p PE=2 SV=1	0.418476218	0.243427426	0.175048792	0.063825723	0.343364038	0.284324451
Q59FM8	RBR-type E3 ubiquitin transferase (Fragment) OS=Homo sapiens OX=9606 PE=2 SV=1	0.205608233	0.384028499	-0.178420266	0.064367156	0.004385554	0.089754344
P10155	60 kDa SS-A/Ro ribonucleoprotein OS=Homo sapiens OX=9606 GN=RO60 PE=1 SV=2	0.439624882	0.457835251	-0.01821037	0.06454908	0.005206678	0.447658762
P51888	Prolargin OS=Homo sapiens OX=9606 GN=PRELP PE=1 SV=1	0.447287938	0.933512598	-0.486224659	0.065190793	0.003061971	0.074845386
P08559	Pyruvate dehydrogenase E1 component subunit alpha, somatic form,	-0.31515607	0.259986453	-0.575142523	0.065191349	0.07323869	0.00418251

	mitochondrial OS=Homo sapiens OX=9606 GN=PDHA1 PE=1 SV=3						
Q13200	26S proteasome non-ATPase regulatory subunit 2 OS=Homo sapiens OX=9606 GN=PSMD2 PE=1 SV=3	0.336830034	0.554338692	-0.217508658	0.065246554	0.003398833	0.169548553
Q01432	AMP deaminase 3 OS=Homo sapiens OX=9606 GN=AMPD3 PE=1 SV=1	0.775744192	0.423980009	0.351764183	0.065258024	0.013446295	0.706591121
P00167	Cytochrome b5 OS=Homo sapiens OX=9606 GN=CYB5A PE=1 SV=2	0.255872448	0.496707353	-0.240834905	0.065365091	0.002990787	0.051503637
B3KR50	cDNA FLJ33691 fis, clone BRAWH2002976, highly similar to GROWTH FACTOR RECEPTOR-BOUND PROTEIN 2 OS=Homo sapiens OX=9606 PE=2 SV=1	0.238925416	0.392743086	-0.15381767	0.065589957	0.010771441	0.230273324
A4D0R4	B-cell receptor-associated protein 29 OS=Homo sapiens OX=9606 GN=BCAP29 PE=4 SV=1	0.325019534	0.511163565	-0.186144031	0.065869652	0.005165145	0.188275054
A0A4V1EJ16	IgK_IGKV2-40 (Fragment) OS=Homo sapiens OX=9606 PE=2 SV=1	0.762799154	-0.199952293	0.962751447	0.066827853	0.710250777	0.026534764
A0A0A0MTS7	Titin OS=Homo sapiens OX=9606 GN=TTN PE=1 SV=1	0.409317316	0.575791638	-0.166474322	0.066917554	0.043375786	0.495819493
C9JRD2	DnaJ homolog subfamily B member 2 (Fragment) OS=Homo sapiens OX=9606 GN=DNAJB2 PE=1 SV=1	0.246452117	0.326639829	-0.080187712	0.067037452	0.02544088	0.410451417
A0A024R5Q4	Glycine amidinotransferase (L-arginine:glycine amidinotransferase), isoform CRA_a OS=Homo sapiens	-0.797037796	-0.236606999	-0.560430797	0.067187449	0.986024819	0.11118834

	OX=9606 GN=GATM PE=4 SV=1						
Q9BRF8	Serine/threonine-protein phosphatase CPPED1 OS=Homo sapiens OX=9606 GN=CPPED1 PE=1 SV=3	0.335937977	-0.133826391	0.469764367	0.067378966	0.09945346	0.014999979
Q9BY44	Eukaryotic translation initiation factor 2A OS=Homo sapiens OX=9606 GN=EIF2A PE=1 SV=3	0.550569229	0.98767505	-0.437105821	0.067618012	0.002236461	0.066833149
B2RBE0	cDNA, FLJ95462, highly similar to Homo sapiens fatty-acid-Coenzyme A ligase, long-chain 3 (FACL3),mRNA OS=Homo sapiens OX=9606 PE=2 SV=1	0.456159148	0.971902893	-0.515743744	0.068687858	0.001427772	0.017764534
Q09666	Neuroblast differentiation-associated protein AHNAK OS=Homo sapiens OX=9606 GN=AHNAK PE=1 SV=2	0.510630698	0.471874148	0.03875655	0.069239679	0.002964422	0.63956036
E9PF58	Actin-related protein 2/3 complex subunit 1A OS=Homo sapiens OX=9606 GN=ARPC1A PE=1 SV=1	0.286480776	0.558750007	-0.272269231	0.069339722	0.0030385	0.042079483
Q3KNW1	Zinc finger protein SNAI3 OS=Homo sapiens OX=9606 GN=SNAI3 PE=2 SV=1	0.524279659	0.198507426	0.325772232	0.069782867	0.412066176	0.388198787
Q96CE4	Stathmin OS=Homo sapiens OX=9606 GN=STMN1 PE=1 SV=1	0.497772734	0.260830831	0.236941904	0.07028825	0.014696889	0.724242898
X5D2F4	Cytoplasmic FMR1 interacting protein 1 isoform A (Fragment) OS=Homo sapiens OX=9606 GN=CYFIP1 PE=2 SV=1	0.638627367	0.994136533	-0.355509167	0.071126877	0.002276333	0.128078336
P59998	Actin-related protein 2/3 complex subunit 4 OS=Homo sapiens	0.362013068	0.51288149	-0.150868422	0.07134506	0.003490992	0.199374551

	OX=9606 GN=ARPC4 PE=1 SV=3						
B3KX36	cDNA FLJ44620 fis, clone BRACE2013132 OS=Homo sapiens OX=9606 PE=2 SV=1	0.45377562	0.36899 9565	0.084776 055	0.0719 36512	0.0953 27361	0.6177 90325
Q96BU 1	S100P-binding protein OS=Homo sapiens OX=9606 GN=S100PBP PE=1 SV=1	- 0.35506551 1	0.74940 5842	- 1.104471 353	0.0721 04118	0.0022 13576	6.1930 2E-05
P54920	Alpha-soluble NSF attachment protein OS=Homo sapiens OX=9606 GN=NAPA PE=1 SV=3	0.37296781 9	0.75502 5073	- 0.382057 254	0.0721 50695	0.0034 71049	0.0863 29192
Q92547	DNA topoisomerase 2-binding protein 1 OS=Homo sapiens OX=9606 GN=TOPBP1 PE=1 SV=3	- 0.70602239 7	- 0.33945 4169	- 0.366568 228	0.0722 3919	0.6791 81261	0.1938 54886
Q3B7A 7	Trifunctional purine biosynthetic protein adenosine-3 OS=Homo sapiens OX=9606 GN=GART PE=2 SV=1	0.35223165 4	0.37209 5217	- 0.019863 563	0.0723 35127	0.0024 13161	0.6400 4203
B4DZ08	Aconitate hydratase, mitochondrial OS=Homo sapiens OX=9606 PE=2 SV=1	- 0.31527465 2	0.51146 7447	- 0.826742 098	0.0736 03118	0.0062 27329	0.0001 51526
Q96RL7	Vacuolar protein sorting-associated protein 13A OS=Homo sapiens OX=9606 GN=VPS13A PE=1 SV=2	-0.499382	0.06999 3786	- 0.569375 787	0.0739 12029	0.6412 59981	0.0460 26545
Q15555	Microtubule-associated protein RP/EB family member 2 OS=Homo sapiens OX=9606 GN=MAPRE2 PE=1 SV=1	0.43456314 9	0.41397 0473	0.020592 676	0.0739 73903	0.0093 85779	0.9432 57821
Q16698	2,4-dienoyl-CoA reductase, mitochondrial OS=Homo sapiens OX=9606 GN=DECR1 PE=1 SV=1	- 0.29161947 9	0.32569 3102	- 0.617312 581	0.0740 14353	0.1724 30037	0.0476 28676
Q5T5C7	Serine--tRNA ligase, cytoplasmic OS=Homo	0.38098485 7	0.58445 8479	- 0.203473 623	0.0740 96422	0.0015 45468	0.1205 46261

	sapiens OX=9606 GN=SARS1 PE=1 SV=1						
Q92973	Transportin-1 OS=Homo sapiens OX=9606 GN=TNPO1 PE=1 SV=2	0.428328612	0.723948985	-0.295620373	0.074464761	0.002977875	0.117247006
Q92621	Nuclear pore complex protein Nup205 OS=Homo sapiens OX=9606 GN=NUP205 PE=1 SV=3	0.414945671	0.542988365	-0.128042693	0.074553966	0.01408875	0.386657067
A0A024QZN4	Vinculin, isoform CRA_c OS=Homo sapiens OX=9606 GN=VCL PE=4 SV=1	0.287895717	0.464857612	-0.176961894	0.074591085	0.011459518	0.202947024
Q9UNL6	Hemoglobin gamma-G (Fragment) OS=Homo sapiens OX=9606 GN=HBG2 PE=2 SV=1	-0.553450518	-0.234743496	-0.318707023	0.074628302	0.329934952	0.592285186
V9HW74	Ubiquitin carboxyl-terminal hydrolase OS=Homo sapiens OX=9606 GN=HEL-117 PE=2 SV=1	0.74975285	0.452173471	0.297579379	0.074708569	0.093032093	0.286915914
B4DTY3	cDNA FLJ61403, highly similar to Nuclear ubiquitous casein andcyclin-dependent kinases substrate OS=Homo sapiens OX=9606 PE=2 SV=1	0.668773641	0.94302547	-0.274251829	0.074712173	0.007693283	0.42256534
A0ZT98	Testis tissue sperm-binding protein Li 55e OS=Homo sapiens OX=9606 GN=TSSK1 PE=2 SV=1	-0.641954185	-0.53321302	-0.108741166	0.075011287	0.208604267	0.352660904
Q3MIH3	Ubiquitin A-52 residue ribosomal protein fusion product 1 OS=Homo sapiens OX=9606 GN=UBA52 PE=2 SV=1	0.223492969	0.122177534	0.101315435	0.07582964	0.247488142	0.707235478
Q9H2U2	Inorganic pyrophosphatase 2, mitochondrial OS=Homo sapiens OX=9606 GN=PPA2 PE=1 SV=2	-0.412467042	0.576726228	-0.98919327	0.076245581	0.002606145	3.95383E-05

A0A140T902	Tenascin-X OS=Homo sapiens OX=9606 GN=TNXB PE=1 SV=1	0.365284519	0.604948145	-0.239663626	0.077602446	0.006210476	0.125672933
B4DDQ2	Biglycan OS=Homo sapiens OX=9606 PE=2 SV=1	0.749700683	1.079911877	-0.330211194	0.077611346	0.002827382	0.20857542
A0A1B0GU92	Peptidase A1 domain-containing protein OS=Homo sapiens OX=9606 PE=1 SV=1	0.68187321	0.401868051	0.280005159	0.077659396	0.113506449	0.777837655
E5KNY5	Leucine-rich PPR-motif containing OS=Homo sapiens OX=9606 GN=LRPPRC PE=4 SV=1	-0.395182017	0.720324984	-1.115507	0.077684152	0.002942209	0.0001583
B1AQT0	cDNA FLJ35042 fis, clone OCBBF2017489, highly similar to Homo sapiens ankyrin 3, node of Ranvier (ankyrin G) (ANK3), transcript variant 2, mRNA OS=Homo sapiens OX=9606 PE=2 SV=1	0.317035082	0.619154448	-0.302119365	0.077800635	0.003495217	0.124060973
B4DWC4	Chloride intracellular channel protein OS=Homo sapiens OX=9606 PE=2 SV=1	0.418382195	0.641031816	-0.222649622	0.077807349	0.001480905	0.132918274
P21912	Succinate dehydrogenase [ubiquinone] iron-sulfur subunit, mitochondrial OS=Homo sapiens OX=9606 GN=SDHB PE=1 SV=3	-0.42214517	0.458350802	-0.880495972	0.078016798	0.005199904	3.79319E-05
A0A024R962	HCG40889, isoform CRA_b OS=Homo sapiens OX=9606 GN=hCG_40889 PE=4 SV=1	0.472972706	0.028923071	0.444049635	0.078959056	0.526275095	0.120761762
A0A494C0G1	Phosphoacetylglucosamine mutase OS=Homo sapiens OX=9606 GN=PGM3 PE=1 SV=1	0.375837696	0.311453733	0.064383964	0.079764478	0.016640379	0.734987164
R4SBI6	Epoxide hydrolase OS=Homo sapiens OX=9606 GN=EPHX1 PE=2 SV=1	0.252829112	0.427382481	-0.174553369	0.079865096	0.006910739	0.129422567

A0A0K0K1H8	Epididymis secretory sperm binding protein Li 71p OS=Homo sapiens OX=9606 GN=HEL-S-71p PE=2 SV=1	0.486260236	-0.118831737	0.605091972	0.080552145	0.757147931	0.030505081
B5BUB5	Autoantigen La (Fragment) OS=Homo sapiens OX=9606 GN=SSB PE=2 SV=1	0.539094592	0.683108545	-0.144013953	0.080641763	0.002296165	0.342788829
B7Z792	cDNA FLJ53932, highly similar to NADH-ubiquinone oxidoreductase 49 kDa subunit, mitochondrial OS=Homo sapiens OX=9606 PE=2 SV=1	-0.377933496	0.369343976	-0.747277472	0.08128718	0.007392791	0.00010038
B7Z4V2	cDNA FLJ51907, highly similar to Stress-70 protein, mitochondrial OS=Homo sapiens OX=9606 PE=2 SV=1	-0.279285583	0.518659983	-0.797945566	0.081406014	0.003290781	0.000151609
Q71UP1	Cytochrome c oxidase subunit Va (Fragment) OS=Homo sapiens OX=9606 GN=COX5A PE=4 SV=1	-0.397576551	0.369289108	-0.766865658	0.081944858	0.174648182	0.028674241
P53618	Coatomer subunit beta OS=Homo sapiens OX=9606 GN=COPB1 PE=1 SV=3	0.36363615	0.578763973	-0.215127822	0.082465713	0.002387109	0.120324544
Q10471	Polypeptide N-acetylgalactosaminyltransferase 2 OS=Homo sapiens OX=9606 GN=GALNT2 PE=1 SV=1	0.383138731	0.598303276	-0.215164546	0.082697607	0.003180581	0.13536549
Q9NQU3	Uncharacterized protein (Fragment) OS=Homo sapiens OX=9606 PE=2 SV=1	-0.672788605	-0.54659283	-0.126195775	0.082973507	0.428166476	0.447337904
Q9BZV1	UBX domain-containing protein 6 OS=Homo sapiens OX=9606 GN=UBXN6 PE=1 SV=1	0.337439206	0.408244585	-0.070805379	0.083249156	0.003980868	0.393972913
O75339	Cartilage intermediate layer protein 1 OS=Homo sapiens	0.608743017	1.073273588	-0.464530571	0.083289651	0.003365949	0.120353595

	OX=9606 GN=CILP PE=1 SV=4						
Q3MI39	HNRPA1 protein (Fragment) OS=Homo sapiens OX=9606 GN=HNRPA1 PE=2 SV=1	0.669122611	0.903875268	-0.234752657	0.083816398	0.016752811	0.280854875
E7ENN3	Nesprin-1 OS=Homo sapiens OX=9606 GN=SYNE1 PE=1 SV=2	-0.663244249	-0.601433128	-0.06181112	0.083830313	0.512516312	0.465142272
A0A090N8G0	Glycyl-tRNA synthetase OS=Homo sapiens OX=9606 GN=GARS PE=4 SV=1	0.368358767	0.396862556	-0.028503789	0.083962636	0.003687251	0.517077464
A0A024R7J0	Protein kinase, cAMP-dependent, catalytic, alpha, isoform CRA_c OS=Homo sapiens OX=9606 GN=PRKACA PE=3 SV=1	0.246731028	0.368309132	-0.121578105	0.08411824	0.007781926	0.268358378
Q2F831	Tyrosine 3-monooxygenasea/tryptophan 5-monooxygenase activation protein zeta (Fragment) OS=Homo sapiens OX=9606 PE=2 SV=1	0.387154196	0.559356873	-0.172202677	0.085668585	0.002992451	0.180829731
E9PC52	Histone-binding protein RBBP7 OS=Homo sapiens OX=9606 GN=RBBP7 PE=1 SV=1	0.450802163	0.513044392	-0.062242229	0.085855419	0.00459329	0.584462385
B4DL67	cDNA FLJ53392, highly similar to Ubiquitin-activating enzyme E1 OS=Homo sapiens OX=9606 PE=2 SV=1	0.504663743	0.397857286	0.106806457	0.086040106	0.086356232	0.922529885
B2RB47	AMP deaminase OS=Homo sapiens OX=9606 PE=2 SV=1	0.352114325	0.57961791	-0.227503585	0.087117714	0.010986841	0.278028855
G3V180	Dipeptidyl peptidase 3 OS=Homo sapiens OX=9606 GN=DPP3 PE=1 SV=1	0.251451533	0.064635374	0.18681616	0.087166758	0.531127722	0.482345047
Q9UGI8	Testin OS=Homo sapiens OX=9606 GN=TES PE=1 SV=1	0.454059869	0.445172074	0.008887794	0.087279443	0.012977852	0.543499846

P24539	ATP synthase F(0) complex subunit B1, mitochondrial OS=Homo sapiens OX=9606 GN=ATP5PB PE=1 SV=2	-0.332925373	0.536963855	-0.869889228	0.089104845	0.004787948	3.14246E-05
P36957	Dihydrolipoyllysine-residue succinyltransferase component of 2-oxoglutarate dehydrogenase complex, mitochondrial OS=Homo sapiens OX=9606 GN=DLST PE=1 SV=4	-0.418703235	0.559068246	-0.977771482	0.090784257	0.026661314	0.000459514
P21695	Glycerol-3-phosphate dehydrogenase [NAD(+)], cytoplasmic OS=Homo sapiens OX=9606 GN=GPD1 PE=1 SV=4	0.47086862	0.451467805	0.019400815	0.090993601	0.046210619	0.879416189
Q6IAQ2	SDHC protein OS=Homo sapiens OX=9606 GN=SDHC PE=2 SV=1	-0.362132002	0.378608517	-0.740740518	0.09105087	0.009148955	0.000284018
Q9BXB4	Oxysterol-binding protein-related protein 11 OS=Homo sapiens OX=9606 GN=OSBPL11 PE=1 SV=2	0.264164694	0.735597415	-0.471432721	0.09121061	0.003883204	0.065009428
A0A024R3V8	Translin-associated factor X, isoform CRA_c OS=Homo sapiens OX=9606 GN=TSNAX PE=4 SV=1	0.435368902	0.403296637	0.032072265	0.092253868	0.006727038	0.841620327
B3KMV5	cDNA FLJ12728 fis, clone NT2RP2000040, highly similar to Protein FAM62A OS=Homo sapiens OX=9606 PE=2 SV=1	0.409224526	0.714185829	-0.304961303	0.092580305	0.003620986	0.070890756
A0A384ME17	Elongation factor Tu OS=Homo sapiens OX=9606 GN=TUFM PE=1 SV=1	-0.343390791	0.613293111	-0.956683903	0.09264253	0.003642346	6.661E-05
Q8NEG8	CTAGE1 protein (Fragment) OS=Homo	0.556687717	0.76432751	-0.207639793	0.093293062	0.004778524	0.486887626

	sapiens OX=9606 GN=CTAGE1 PE=2 SV=2						
Q53GN 4	WD repeat domain 1, isoform CRA_a (Fragment) OS=Homo sapiens OX=9606 GN=WDR1 PE=2 SV=1	0.31036475 4	0.50086 4642	- 0.190499 889	0.0937 92457	0.0060 00643	0.1947 47984
Q5T440	Putative transferase CAF17, mitochondrial OS=Homo sapiens OX=9606 GN=IBA57 PE=1 SV=1	- 0.33802634 2	0.82565 5823	- 1.163682 165	0.0938 4236	0.0012 98587	6.5828 3E-06
A4ZU86	Truncated nucleolar phosphoprotein B23 OS=Homo sapiens OX=9606 GN=NPM1 PE=2 SV=1	0.36900092 7	0.49476 0483	- 0.125759 557	0.0938 76198	0.0110 0686	0.3078 07377
G4XXL9	Cytochrome c OS=Homo sapiens OX=9606 GN=CYCS PE=3 SV=1	- 0.28288576 6	0.46852 4209	- 0.751409 976	0.0946 2361	0.0755 94175	0.0128 60946
C9J406	MICOS complex subunit MIC60 OS=Homo sapiens OX=9606 GN=IMMT PE=1 SV=1	-0.2651906	0.33374 9924	- 0.598940 524	0.0946 65184	0.0192 11832	0.0016 84034
Q9Y6M 9	NADH dehydrogenase [ubiquinone] 1 beta subcomplex subunit 9 OS=Homo sapiens OX=9606 GN=NDUFB9 PE=1 SV=3	- 0.35491206 6	0.53187 2603	- 0.886784 669	0.0961 99925	0.0045 19479	5.517E -05
Q9C037	E3 ubiquitin-protein ligase TRIM4 OS=Homo sapiens OX=9606 GN=TRIM4 PE=1 SV=2	0.37062577 6	0.43720 5057	- 0.066579 281	0.0962 63023	0.0095 92831	0.6577 62913
A0A087 WYT3	Prostaglandin E synthase 3 OS=Homo sapiens OX=9606 GN=PTGES3 PE=1 SV=1	0.37577296 6	0.30314 1418	0.072631 548	0.0964 78922	0.0794 48593	0.9004 73066
Q14515	SPARC-like protein 1 OS=Homo sapiens OX=9606 GN=SPARCL1 PE=1 SV=2	0.48279182 6	0.79789 8421	- 0.315106 595	0.0966 13653	0.0013 17608	0.1171 91864
E9PKB5	Protein phosphatase 1A (Fragment) OS=Homo sapiens OX=9606 GN=PPM1A PE=1 SV=1	0.26688338 3	0.28233 7835	- 0.015454 451	0.0968 27102	0.0045 05337	0.8895 14429

P68133	Actin, alpha skeletal muscle OS=Homo sapiens OX=9606 GN=ACTA1 PE=1 SV=1	-0.471681531	-0.444562554	-0.027118977	0.096906198	0.24428362	0.592570531
A8K690	cDNA FLJ76863, highly similar to Homo sapiens stress-induced-phosphoprotein 1 (Hsp70/Hsp90-organizing protein) (STIP1), mRNA OS=Homo sapiens OX=9606 PE=2 SV=1	0.209451084	0.200812877	0.008638207	0.097060828	0.1346961	0.872515103
A0A5H1ZRP8	Dystrophin OS=Homo sapiens OX=9606 GN=DMD PE=1 SV=1	0.455985925	0.689993144	-0.234007218	0.097223431	0.007420088	0.338478235
W8QEH3	Lamin A/C OS=Homo sapiens OX=9606 GN=LMNA PE=3 SV=1	0.44719958	0.51302981	-0.06583023	0.097429428	0.032976086	0.425218486
A0A024R8S5	Protein disulfide-isomerase OS=Homo sapiens OX=9606 GN=P4HB PE=2 SV=1	0.394730493	0.655834696	-0.261104202	0.09847608	0.007781519	0.155901169
Q6FGX9	Adenylate kinase isoenzyme 1 OS=Homo sapiens OX=9606 GN=AK1 PE=2 SV=1	0.50652058	0.152167703	0.354352877	0.101591177	0.939524346	0.090904223
J9R021	Eukaryotic translation initiation factor 3 subunit A OS=Homo sapiens OX=9606 GN=eIF3a PE=2 SV=1	0.374550482	0.766591009	-0.392040527	0.10161806	0.002797791	0.110967127
V9H1D9	Alpha globin OS=Homo sapiens OX=9606 PE=3 SV=1	-0.547878222	-0.293997945	-0.253880277	0.101737501	0.220243309	0.520170265
A0A286YFJ8	Immunoglobulin heavy constant gamma 4 (Fragment) OS=Homo sapiens OX=9606 GN=IGHG4 PE=1 SV=1	1.352663817	-0.272018304	1.624682121	0.102833415	0.172428977	0.011955008
D6W5C0	Spectrin beta chain OS=Homo sapiens OX=9606 GN=SPTBN1 PE=3 SV=1	0.325824656	0.527803191	-0.201978536	0.103775006	0.008764075	0.177653014
O43837	Isocitrate dehydrogenase [NAD] subunit beta,	-0.312801205	0.568520669	-0.881321874	0.103866799	0.004150195	9.38104E-05

	mitochondrial OS=Homo sapiens OX=9606 GN=IDH3B PE=1 SV=2						
O94903	Pyridoxal phosphate homeostasis protein OS=Homo sapiens OX=9606 GN=PLPBP PE=1 SV=1	0.284227493	0.466179139	-0.181951645	0.103902958	0.005293431	0.341703888
P31040	Succinate dehydrogenase [ubiquinone] flavoprotein subunit, mitochondrial OS=Homo sapiens OX=9606 GN=SDHA PE=1 SV=2	-0.429809816	0.539546524	-0.96935634	0.103978807	0.006746606	6.09748E-06
Q9H3Z4	DnaJ homolog subfamily C member 5 OS=Homo sapiens OX=9606 GN=DNAJC5 PE=1 SV=1	0.566782741	0.862206731	-0.295423991	0.104199265	0.002482879	0.161673793
Q02224	Centromere-associated protein E OS=Homo sapiens OX=9606 GN=CENPE PE=1 SV=2	-0.616472931	-0.372703605	-0.243769326	0.104277772	0.900289532	0.265428839
Q96Q62	Mitochondrial ribosomal protein S11 (Fragment) OS=Homo sapiens OX=9606 GN=MRPS11 PE=4 SV=1	-0.361692593	0.476216645	-0.837909238	0.1049554	0.007431637	0.001103291
Q53G71	Calreticulin variant (Fragment) OS=Homo sapiens OX=9606 PE=2 SV=1	0.38438005	0.527613394	-0.143233344	0.105786312	0.004322601	0.219164853
Q9BZQ8	Protein Niban 1 OS=Homo sapiens OX=9606 GN=NIBAN1 PE=1 SV=1	0.702991325	0.880393794	-0.177402469	0.105895187	0.013822994	0.391410417
A4UGR9	Xin actin-binding repeat-containing protein 2 OS=Homo sapiens OX=9606 GN=XIRP2 PE=1 SV=2	0.357615965	0.755381803	-0.397765838	0.107136327	0.018123897	0.137643113
F8W7U0	Intersectin-1 OS=Homo sapiens OX=9606 GN=ITSN1 PE=1 SV=1	0.398432477	0.65691894	-0.258486463	0.107137511	0.003140207	0.152775347
B0YIW5	Coatomer subunit delta OS=Homo sapiens	0.386195176	0.606336632	-0.220141456	0.107317538	0.004424832	0.161929982

	OX=9606 GN=ARCN1 PE=2 SV=1						
P28161	Glutathione S-transferase Mu 2 OS=Homo sapiens OX=9606 GN=GSTM2 PE=1 SV=2	0.44045824	-0.033356007	0.473814247	0.108052797	0.572377203	0.021020447
A0A024RA28	Heterogeneous nuclear ribonucleoprotein A2/B1, isoform CRA_d OS=Homo sapiens OX=9606 GN=HNRPA2B1 PE=4 SV=1	0.442068098	0.309615668	0.13245243	0.108129681	0.064646123	0.818097402
Q8TBT6	Uncharacterized protein (Fragment) OS=Homo sapiens OX=9606 PE=2 SV=1	-0.276637342	0.272160506	-0.548797847	0.108162822	0.020337273	0.002345892
B3KNK4	Phosphatidate cytidylyltransferase OS=Homo sapiens OX=9606 PE=2 SV=1	0.348033241	0.870411604	-0.522378363	0.108589027	0.002209799	0.012625285
P10916	Myosin regulatory light chain 2, ventricular/cardiac muscle isoform OS=Homo sapiens OX=9606 GN=MYL2 PE=1 SV=3	-0.401508153	-0.110480768	-0.291027386	0.108680432	0.921000467	0.080649925
B2RE74	cDNA, FLJ94876, highly similar to Homo sapiens fructose-1,6-bisphosphatase 1 (FBP1), mRNA OS=Homo sapiens OX=9606 PE=2 SV=1	0.534285441	0.803857685	-0.269572244	0.109342595	0.004221753	0.407207937
C9JRZ6	MICOS complex subunit OS=Homo sapiens OX=9606 GN=CHCHD3 PE=1 SV=2	-0.393321854	0.510370059	-0.903691913	0.109453092	0.012005495	0.000148631
H0YB09	D-glutamate cyclase, mitochondrial (Fragment) OS=Homo sapiens OX=9606 GN=DGLUCY PE=1 SV=1	-0.518101229	0.11236184	-0.630463069	0.109526088	0.475621101	0.043586778
P08779	Keratin, type I cytoskeletal 16	0.327995237	0.272562536	0.055432701	0.109753572	0.066258918	0.90878755

	OS=Homo sapiens OX=9606 GN=KRT16 PE=1 SV=4						
A2RRP1	Neuroblastoma-amplified sequence OS=Homo sapiens OX=9606 GN=NBAS PE=1 SV=2	0.301721368	0.550519131	-0.248797763	0.110706004	0.00374868	0.141743647
Q6ZMU5	Tripartite motif-containing protein 72 OS=Homo sapiens OX=9606 GN=TRIM72 PE=1 SV=2	0.24033245	0.546270277	-0.305937827	0.110790822	0.003732231	0.103357862
B4DEB0	cDNA FLJ56054, highly similar to 26S proteasome non-ATPase regulatory subunit 3 OS=Homo sapiens OX=9606 PE=2 SV=1	0.335377042	0.581068288	-0.245691246	0.111037529	0.003489866	0.110647474
B7Z3F9	cDNA FLJ61645, highly similar to Homo sapiens nitrilase family, member 2 (NIT2), mRNA OS=Homo sapiens OX=9606 PE=2 SV=1	-0.466900796	-0.155935766	-0.31096503	0.111182415	0.489399607	0.252445957
P48163	NADP-dependent malic enzyme OS=Homo sapiens OX=9606 GN=ME1 PE=1 SV=1	0.280656031	0.426186655	-0.145530623	0.111256832	0.006114294	0.531627029
A0A024R3C4	KDEL (Lys-Asp-Glu-Leu) containing 2, isoform CRA_a OS=Homo sapiens OX=9606 GN=KDELC2 PE=4 SV=1	0.441010416	0.741209951	-0.300199535	0.111899181	0.005793759	0.147982087
A0A087WX60	Myopalladin OS=Homo sapiens OX=9606 GN=MYPN PE=1 SV=1	0.277525371	0.496121187	-0.218595817	0.111985069	0.005179836	0.13232124
Q8N5L9	Ribosomal protein S2 OS=Homo sapiens OX=9606 GN=RPS2 PE=2 SV=1	0.329116185	0.504651808	-0.175535624	0.112211883	0.007688371	0.207655666
Q99729	Heterogeneous nuclear ribonucleoprotein A/B OS=Homo sapiens OX=9606 GN=HNRNPAB PE=1 SV=2	0.559738886	0.342140845	0.217598041	0.113054301	0.024784178	0.945222482

O60763	General vesicular transport factor p115 OS=Homo sapiens OX=9606 GN=USO1 PE=1 SV=2	0.290847282	0.550123843	-0.259276561	0.114398608	0.006223357	0.183944081
E5KSX8	Mitochondrial transcription factor A OS=Homo sapiens OX=9606 PE=4 SV=1	-0.316993317	0.303890744	-0.620884061	0.114850801	0.013078682	0.000324914
C9J066	Ninein OS=Homo sapiens OX=9606 GN=NIN PE=1 SV=1	-0.673603437	-0.467274789	-0.206328647	0.115105765	0.446310593	0.341696782
A0A024R687	Pleckstrin homology domain containing, family C (With FERM domain) member 1, isoform CRA_b OS=Homo sapiens OX=9606 GN=PLEKHC1 PE=4 SV=1	0.55905682	0.585932669	-0.026875849	0.115140671	0.003664835	0.807493151
A0A2R8Y6G6	Alpha-enolase OS=Homo sapiens OX=9606 GN=ENO1 PE=1 SV=1	0.309411781	0.383777361	-0.074365579	0.116642135	0.063057818	0.872192631
Q8IZP2	Putative protein FAM10A4 OS=Homo sapiens OX=9606 GN=ST13P4 PE=5 SV=1	-0.272954554	-0.257396713	-0.01555784	0.116659993	0.188460989	0.676495689
Q53EW4	Myotubularin-related protein 9 variant (Fragment) OS=Homo sapiens OX=9606 PE=2 SV=1	0.285622936	0.470697583	-0.185074647	0.118188169	0.003252254	0.117175627
Q20BI4	Cystic fibrosis transmembrane conductance regulator OS=Homo sapiens OX=9606 GN=CFTR PE=3 SV=1	0.318696192	0.448862361	-0.130166169	0.118832155	0.013969425	0.376779442
B2RTX2	Palladin, cytoskeletal associated protein OS=Homo sapiens OX=9606 GN=PALLD PE=2 SV=1	0.243024502	0.419191171	-0.176166668	0.119011845	0.012373585	0.201808793
A6XMH5	Beta-2-microglobulin OS=Homo sapiens OX=9606 PE=2 SV=1	1.007510206	0.410670997	0.596839209	0.119810405	0.027004016	0.657777003

B5MDF5	GTP-binding nuclear protein Ran OS=Homo sapiens OX=9606 GN=RAN PE=1 SV=1	0.295224891	0.290630779	0.004594112	0.121776903	0.023438769	0.809477901
A0A087WYF8	PDZ and LIM domain protein 3 OS=Homo sapiens OX=9606 GN=PDLIM3 PE=1 SV=2	0.36610217	-0.039513058	0.405615228	0.122547369	0.82130538	0.080551967
Q15075	Early endosome antigen 1 OS=Homo sapiens OX=9606 GN=EEA1 PE=1 SV=2	0.300897653	0.521135252	-0.220237599	0.12259839	0.003350324	0.095128365
A0A024R0G5	Zinc finger protein 607, isoform CRA_b OS=Homo sapiens OX=9606 GN=ZNF607 PE=4 SV=1	-0.385244832	-0.300566131	-0.084678701	0.12263442	0.79908524	0.395171689
K7EMF8	Very long-chain-specific acyl-CoA dehydrogenase, mitochondrial (Fragment) OS=Homo sapiens OX=9606 GN=ACADVL PE=1 SV=1	-0.316427752	0.582392363	-0.898820115	0.123446355	0.003365665	0.000180773
Q7Z4X2	Neuronal protein OS=Homo sapiens OX=9606 PE=2 SV=1	-0.281732308	0.49916092	-0.780893228	0.12417625	0.003022449	1.40828E-05
A8K7H3	cDNA FLJ77670, highly similar to Homo sapiens ribosomal protein S15a (RPS15A), mRNA OS=Homo sapiens OX=9606 PE=2 SV=1	0.550671249	0.477883934	0.072787315	0.124183954	0.191406666	0.795624067
A0A0C4DGA2	Enoyl-CoA delta isomerase 2, mitochondrial OS=Homo sapiens OX=9606 GN=ECI2 PE=1 SV=1	-0.284305736	0.385950133	-0.670255869	0.124233707	0.027440372	0.004278565
A0A024RBS1	GCN1 general control of amino-acid synthesis 1-like 1 (Yeast), isoform CRA_b OS=Homo sapiens OX=9606 GN=GCN1L1 PE=4 SV=1	0.292437478	0.39795556	-0.105518122	0.124649214	0.013931682	0.382225152
B4DUF1	cDNA FLJ59760, highly similar to 1,4-alpha-glucan branching	0.286321303	0.64045278	-0.354131477	0.125440485	0.00330364	0.041599293

	enzyme OS=Homo sapiens OX=9606 PE=2 SV=1						
A0A0S2Z4C3	Epididymis secretory sperm binding protein (Fragment) OS=Homo sapiens OX=9606 GN=FH PE=2 SV=1	-0.228033013	0.145338355	-0.373371368	0.125807669	0.086674376	0.015049067
Q5H924	HECT, UBA and WWE domain containing 1 (Fragment) OS=Homo sapiens OX=9606 GN=HUWE1 PE=4 SV=1	0.340908231	0.602964757	-0.262056526	0.125858074	0.001441022	0.133539905
Q10713	Mitochondrial-processing peptidase subunit alpha OS=Homo sapiens OX=9606 GN=PMPCA PE=1 SV=2	-0.268237704	0.550838251	-0.819075955	0.125999133	0.002972143	0.000146596
Q05639	Elongation factor 1-alpha 2 OS=Homo sapiens OX=9606 GN=EEF1A2 PE=1 SV=1	0.229005327	0.368183541	-0.139178214	0.126618371	0.006397136	0.231445229
Q86T62	Uncharacterized protein DKFZp451F173 OS=Homo sapiens OX=9606 GN=DKFZp451F173 PE=2 SV=1	-0.302063583	0.466317553	-0.768381136	0.126699027	0.016297818	0.000732286
Q16853	Membrane primary amine oxidase OS=Homo sapiens OX=9606 GN=AOC3 PE=1 SV=3	0.441421117	0.287633665	0.153787452	0.12679134	0.046823676	0.875720777
A0A024R3L8	SIR2_2 domain-containing protein OS=Homo sapiens OX=9606 GN=FLJ21103 PE=4 SV=1	0.414144381	0.69679938	-0.282654999	0.127688746	0.006185544	0.212796947
Q597H1	Transformation-related protein 14 OS=Homo sapiens OX=9606 GN=TRG14 PE=2 SV=1	0.399226533	0.497523882	-0.098297349	0.127887899	0.003322021	0.314251274
Q93009	Ubiquitin carboxyl-terminal hydrolase 7 OS=Homo sapiens OX=9606 GN=USP7 PE=1 SV=2	0.329263875	0.555218679	-0.225954805	0.128151255	0.002202689	0.125644687

Q13098	COP9 signalosome complex subunit 1 OS=Homo sapiens OX=9606 GN=GPS1 PE=1 SV=4	0.348967377	0.762494588	-0.413527211	0.130517901	0.003358034	0.08913421
Q13280	Calcium/calmodulin-dependent protein kinase II (Fragment) OS=Homo sapiens OX=9606 PE=2 SV=2	0.383807714	0.558955246	-0.175147532	0.132832445	0.007372746	0.275756275
L8ECF7	Alternative protein GLT25D2 OS=Homo sapiens OX=9606 GN=GLT25D2 PE=4 SV=1	0.516056532	0.6321344	-0.116077867	0.132848469	0.004890213	0.339530647
J3KN67	Tropomyosin alpha-3 chain OS=Homo sapiens OX=9606 GN=TPM3 PE=1 SV=1	-0.572459204	-0.687785438	0.115326234	0.132927852	0.073146721	0.955124517
Q59GJ4	Restin isoform a variant (Fragment) OS=Homo sapiens OX=9606 PE=2 SV=1	0.258907489	0.564351401	-0.305443912	0.133035576	0.00585907	0.113052821
X6RM59	5'-nucleotidase OS=Homo sapiens OX=9606 GN=NT5C3A PE=1 SV=1	0.255169342	0.326752753	-0.071583411	0.133047873	0.011264697	0.520138111
A0A140VKA9	Testis secretory sperm-binding protein Li 236P OS=Homo sapiens OX=9606 PE=2 SV=1	0.197859542	0.273136375	-0.075276833	0.134027092	0.033710481	0.658094673
Q562R0	Actin-like protein (Fragment) OS=Homo sapiens OX=9606 GN=ACT PE=4 SV=1	-0.370619991	-0.463130418	0.092510427	0.134096675	0.084542978	0.9763903
B0QY90	Eukaryotic translation initiation factor 3 subunit L OS=Homo sapiens OX=9606 GN=EIF3L PE=1 SV=1	0.254699714	0.108223498	0.146476217	0.135801083	0.242041068	0.716789565
Q06210	Glutamine--fructose-6-phosphate aminotransferase [isomerizing] 1 OS=Homo sapiens OX=9606 GN=GFPT1 PE=1 SV=3	0.271437551	0.456442848	-0.185005297	0.136385096	0.004019598	0.139392972

Q969H8	Myeloid-derived growth factor OS=Homo sapiens OX=9606 GN=MYDGF PE=1 SV=1	0.43944366	0.744903123	-0.305459463	0.136468623	0.004758322	0.121618858
A0A0K0K1L1	Epididymis secretory protein Li 282 OS=Homo sapiens OX=9606 GN=HEL-S-282 PE=2 SV=1	0.206831595	0.371580403	-0.164748808	0.136570233	0.026490547	0.568283012
B4DI08	cDNA FLJ60091, highly similar to Hypoxia-inducible factor 1 alpha inhibitor OS=Homo sapiens OX=9606 PE=2 SV=1	0.283080003	0.374453088	-0.091373085	0.136751234	0.007775171	0.335040648
E7EVA0	Microtubule-associated protein OS=Homo sapiens OX=9606 GN=MAP4 PE=1 SV=1	0.359150854	0.683989367	-0.324838513	0.137189592	0.005195473	0.132967829
Q9H2W6	39S ribosomal protein L46, mitochondrial OS=Homo sapiens OX=9606 GN=MRPL46 PE=1 SV=1	-0.42719253	0.724300568	-1.151493098	0.138739398	0.003249086	8.44603E-05
Q6IQ30	Polyadenylate-binding protein OS=Homo sapiens OX=9606 GN=PABPC4 PE=2 SV=1	0.38201018	0.322692645	0.059317536	0.139807709	0.058275561	0.784839316
P48059	LIM and senescent cell antigen-like-containing domain protein 1 OS=Homo sapiens OX=9606 GN=LIMS1 PE=1 SV=4	0.342284253	-0.176459788	0.518744041	0.140664181	0.531489501	0.099651341
A6NGQ2	Oocyte-expressed protein homolog OS=Homo sapiens OX=9606 GN=OOEP PE=1 SV=3	0.637709072	0.213921497	0.423787575	0.141645468	0.976667209	0.105876071
Q96HS1	Serine/threonine-protein phosphatase PGAM5, mitochondrial OS=Homo sapiens OX=9606 GN=PGAM5 PE=1 SV=2	0.361456914	0.697386568	-0.335929654	0.141793036	0.003999323	0.098839702

A0A024QZN7	Chromosome 10 open reading frame 70, isoform CRA_b OS=Homo sapiens OX=9606 GN=C10orf70 PE=4 SV=1	-0.265212764	0.657138208	-0.922350972	0.141983681	0.003010841	1.07109E-05
A0A087WZM5	Peptidylprolyl isomerase OS=Homo sapiens OX=9606 GN=FKBP1A PE=1 SV=1	0.286035854	-0.332399154	0.618435008	0.14223024	0.00920034	0.006394548
Q5T4S7	E3 ubiquitin-protein ligase UBR4 OS=Homo sapiens OX=9606 GN=UBR4 PE=1 SV=1	0.357991507	0.570497426	-0.212505919	0.14327762	0.003733406	0.268032098
Q53XJ5	Peptidylprolyl isomerase OS=Homo sapiens OX=9606 GN=FKBP2 PE=2 SV=1	0.354697848	0.520061418	-0.165363569	0.143442184	0.005070013	0.211257571
A0A590UJS6	Sarcolemmal membrane-associated protein (Fragment) OS=Homo sapiens OX=9606 GN=SLMAP PE=1 SV=1	0.343787708	0.684611265	-0.340823557	0.144305849	0.014707443	0.167938675
Q13061	Triadin OS=Homo sapiens OX=9606 GN=TRDN PE=1 SV=4	0.299245518	0.348097649	-0.048852131	0.144404899	0.037901741	0.831358606
P26373	60S ribosomal protein L13 OS=Homo sapiens OX=9606 GN=RPL13 PE=1 SV=4	-0.363567559	-0.006275729	-0.357291831	0.144774071	0.380279344	0.067083299
P51911	Calponin-1 OS=Homo sapiens OX=9606 GN=CNN1 PE=1 SV=2	0.386682631	1.042995089	-0.656312458	0.144803535	0.003380305	0.047495849
H3BRV9	Nuclear transport factor 2 (Fragment) OS=Homo sapiens OX=9606 GN=NUTF2 PE=1 SV=1	0.341625599	-0.28835868	0.62998428	0.145299422	0.139932742	0.039173844
B7Z2P6	cDNA FLJ55296, highly similar to Homo sapiens WD repeat domain 42A (WDR42A), mRNA OS=Homo sapiens OX=9606 PE=2 SV=1	0.349773806	0.518940037	-0.169166231	0.145314964	0.042678849	0.533114775
Q53S33	BolA-like protein 3 OS=Homo sapiens	-0.247276845	0.418648989	-0.665925834	0.145378754	0.037912228	0.006597642

	OX=9606 GN=BOLA3 PE=1 SV=1						
P53778	Mitogen-activated protein kinase 12 OS=Homo sapiens OX=9606 GN=MAPK12 PE=1 SV=3	0.186660356	0.033859857	0.152800499	0.145381274	0.887979016	0.107481041
X5DNC5	Androgen receptor isoform A (Fragment) OS=Homo sapiens OX=9606 GN=AR PE=2 SV=1	-0.409135301	0.552120805	-0.961256105	0.1454441	0.023868861	0.000501806
B2R8Y4	cDNA, FLJ94117, highly similar to Homo sapiens actinin, alpha 3 (ACTN3), mRNA OS=Homo sapiens OX=9606 PE=1 SV=1	0.881792828	0.782501441	0.099291387	0.14547309	0.216472614	0.872613973
Q17RP2	Tigger transposable element-derived protein 6 OS=Homo sapiens OX=9606 GN=TIGD6 PE=2 SV=2	-0.408259222	-0.151143813	-0.257115408	0.145724267	0.750455598	0.161221227
A0A024R462	Fibronectin 1, isoform CRA_n OS=Homo sapiens OX=9606 GN=FN1 PE=4 SV=1	0.444431146	0.442990922	0.001440224	0.14747362	0.032070976	0.607553631
G3V1R5	Nardilysin OS=Homo sapiens OX=9606 GN=NRDC PE=1 SV=1	0.308088269	0.524206756	-0.216118486	0.147687832	0.002378589	0.186407908
P01023	Alpha-2-macroglobulin OS=Homo sapiens OX=9606 GN=A2M PE=1 SV=3	0.537499649	-0.211302325	0.748801974	0.147711332	0.409618084	0.057334414
E0WMV9	MHC class I antigen (Fragment) OS=Homo sapiens OX=9606 GN=HLA-B PE=3 SV=1	0.35825143	0.347327388	0.010924042	0.147821144	0.019830472	0.586758929
E7EUC7	UTP--glucose-1-phosphate uridylyltransferase OS=Homo sapiens OX=9606 GN=UGP2 PE=1 SV=1	0.241570505	0.550272646	-0.308702141	0.147920484	0.007399541	0.080833519
Q9UHQ9	NADH-cytochrome b5 reductase 1 OS=Homo	-0.237437225	0.457559856	-0.694997081	0.147979014	0.006867627	0.000132581

	sapiens OX=9606 GN=CYB5R1 PE=1 SV=1						
B4DH02	cDNA FLJ50510, highly similar to Heat shock 70 kDa protein 4 OS=Homo sapiens OX=9606 PE=2 SV=1	0.239954166	0.416849274	-0.176895108	0.148448965	0.006227038	0.177133036
Q9NZJ6	Ubiquinone biosynthesis O-methyltransferase, mitochondrial OS=Homo sapiens OX=9606 GN=COQ3 PE=1 SV=3	-0.367114063	0.166482915	-0.533596978	0.151769149	0.049653569	0.006155186
Q5BKX8	Caveolae-associated protein 4 OS=Homo sapiens OX=9606 GN=CAVIN4 PE=1 SV=2	0.353304482	0.540803596	-0.187499114	0.1518838	0.019006566	0.263383762
D3DWB6	Ubiquitin specific peptidase 9, X-linked, isoform CRA_b OS=Homo sapiens OX=9606 GN=USP9X PE=3 SV=1	0.312583682	0.681255446	-0.368671763	0.155487386	0.002450507	0.095395976
H0Y9H1	Calnexin (Fragment) OS=Homo sapiens OX=9606 GN=CANX PE=1 SV=8	0.324614033	0.760643408	-0.436029375	0.157254144	0.002255599	0.032380459
A0A024R8D4	Mitochondrial ribosomal protein S2, isoform CRA_a OS=Homo sapiens OX=9606 GN=MRPS2 PE=3 SV=1	-0.272166654	0.616633508	-0.888800163	0.1607934	0.003636356	7.09186E-05
O00429	Dynamin-1-like protein OS=Homo sapiens OX=9606 GN=DNM1L PE=1 SV=2	-0.221852712	0.329423295	-0.551276007	0.161989464	0.011965434	0.001308982
Q14315	Filamin-C OS=Homo sapiens OX=9606 GN=FLNC PE=1 SV=3	0.283251482	0.710729711	-0.427478229	0.163677386	0.006258272	0.124215955
P06744	Glucose-6-phosphate isomerase OS=Homo sapiens OX=9606 GN=GPI PE=1 SV=4	0.265791525	0.313233784	-0.047442259	0.163817742	0.112752934	0.956404299
P05388	60S acidic ribosomal protein P0 OS=Homo sapiens OX=9606 GN=RPLP0 PE=1 SV=1	0.309300005	0.305524959	0.003775046	0.164011629	0.049099064	0.481373198

O75369	Filamin-B OS=Homo sapiens OX=9606 GN=FLNB PE=1 SV=2	0.421416821	0.730436109	-0.309019288	0.164765155	0.004006816	0.157506154
P07738	Bisphosphoglycerate mutase OS=Homo sapiens OX=9606 GN=BPGM PE=1 SV=2	0.563623114	-0.062141684	0.625764799	0.165230203	0.337473507	0.053763143
A7E2F9	SIPA1L2 protein OS=Homo sapiens OX=9606 GN=SIPA1L2 PE=2 SV=1	-0.300250459	0.654158851	-0.95440931	0.165516817	0.006037	0.00032408
B4DNH2	cDNA FLJ58596, highly similar to Eukaryotic initiation factor 4A-I OS=Homo sapiens OX=9606 PE=2 SV=1	0.368776572	0.504085626	-0.135309054	0.165598349	0.01322094	0.394476048
O95831	Apoptosis-inducing factor 1, mitochondrial OS=Homo sapiens OX=9606 GN=AIFM1 PE=1 SV=1	-0.299902254	0.652754292	-0.952656547	0.166464204	0.003638263	4.68997E-05
H0YN84	Serine/threonine-protein phosphatase 2A 56 kDa regulatory subunit gamma isoform (Fragment) OS=Homo sapiens OX=9606 GN=PPP2R5C PE=1 SV=1	0.291168142	0.60255929	-0.311391148	0.16661121	0.003015147	0.074324844
D3DT86	CD55 antigen, decay accelerating factor for complement (Cromer blood group), isoform CRA_g OS=Homo sapiens OX=9606 GN=CD55 PE=4 SV=1	0.383140196	0.607477217	-0.224337021	0.169543534	0.010377008	0.293995302
B4DDZ5	cDNA FLJ53969, highly similar to Trifunctional enzyme subunit alpha, mitochondrial OS=Homo sapiens OX=9606 PE=2 SV=1	-0.390429315	0.527432827	-0.917862143	0.169846737	0.013817135	0.002114033
B7Z3X7	U6 small nuclear RNA (adenine-(43)-N(6))-methyltransferase OS=Homo sapiens OX=9606 PE=2 SV=1	-0.385864912	0.461788855	-0.847653767	0.170458842	0.04921051	0.022137728

Q15642	Cdc42-interacting protein 4 OS=Homo sapiens OX=9606 GN=TRIP10 PE=1 SV=3	0.275362285	0.692870536	-0.417508251	0.170619211	0.003128719	0.06093511
P54578	Ubiquitin carboxyl-terminal hydrolase 14 OS=Homo sapiens OX=9606 GN=USP14 PE=1 SV=3	0.217949564	0.187766986	0.030182578	0.171042657	0.073076354	0.826764388
P43034	Platelet-activating factor acetylhydrolase IB subunit alpha OS=Homo sapiens OX=9606 GN=PAFAH1B1 PE=1 SV=2	0.319344777	0.527109668	-0.207764891	0.171209341	0.006668021	0.20359421
A0A343JRN0	Cytochrome c oxidase subunit 2 OS=Homo sapiens OX=9606 GN=COX2 PE=3 SV=1	-0.241291615	0.529957631	-0.771249246	0.171903859	0.004637379	4.33682E-05
Q53HB3	Proteasome 26S ATPase subunit 1 variant (Fragment) OS=Homo sapiens OX=9606 PE=1 SV=1	0.378533422	0.276601387	0.101932036	0.17191922	0.395585924	0.810201914
P07814	Bifunctional glutamate/proline--tRNA ligase OS=Homo sapiens OX=9606 GN=EPRS1 PE=1 SV=5	0.212604165	0.482142644	-0.269538479	0.172026784	0.004019431	0.07097284
A2RRN2	CAPS2 protein OS=Homo sapiens OX=9606 GN=CAPS2 PE=2 SV=1	-0.492455655	-0.314552299	-0.177903357	0.172066216	0.791999618	0.247704072
P13010	X-ray repair cross-complementing protein 5 OS=Homo sapiens OX=9606 GN=XRCC5 PE=1 SV=3	0.396540805	0.67567239	-0.279131585	0.173792304	0.003292221	0.141015192
E9PQH6	Rho-related GTP-binding protein RhoC (Fragment) OS=Homo sapiens OX=9606 GN=RHOC PE=1 SV=1	0.283311236	0.474546409	-0.191235173	0.173840535	0.003329754	0.176969015
Q08E77	UTP14, U3 small nucleolar ribonucleoprotein, homolog C (Yeast)	0.374764729	0.439835496	-0.065070767	0.174286164	0.01624427	0.598638093

	OS=Homo sapiens OX=9606 GN=UTP14C PE=1 SV=1						
Q8TAS0	ATP synthase subunit gamma (Fragment) OS=Homo sapiens OX=9606 PE=2 SV=1	-0.312776569	0.509120092	-0.821896661	0.17428634	0.004215921	0.000106602
B2RAM6	cDNA, FLJ95005, highly similar to Homo sapiens kinesin family member 11 (KIF11), mRNA OS=Homo sapiens OX=9606 PE=2 SV=1	-0.956227627	-1.33321033	0.376982703	0.174433006	0.02503473	0.434491299
A0A384P5S9	Epididymis secretory sperm binding protein OS=Homo sapiens OX=9606 PE=2 SV=1	0.271042633	0.494479495	-0.223436862	0.174434427	0.008034237	0.132909107
A0A087X1N7	Nebulin OS=Homo sapiens OX=9606 GN=NEB PE=1 SV=1	-0.231562127	0.302102038	-0.533664165	0.176913012	0.020090599	0.005661298
P54646	5'-AMP-activated protein kinase catalytic subunit alpha-2 OS=Homo sapiens OX=9606 GN=PRKAA2 PE=1 SV=2	0.186655798	0.418929666	-0.232273867	0.17719076	0.006854887	0.101066898
B4DHW9	4'-phosphopantetheine phosphatase OS=Homo sapiens OX=9606 PE=2 SV=1	-0.210806383	0.414411527	-0.625217909	0.179699369	0.014464896	0.003403268
O00330	Pyruvate dehydrogenase protein X component, mitochondrial OS=Homo sapiens OX=9606 GN=PDHX PE=1 SV=3	-0.312977357	0.52520326	-0.838180617	0.179843559	0.008048183	0.001400499
P62140	Serine/threonine-protein phosphatase PP1-beta catalytic subunit OS=Homo sapiens OX=9606 GN=PPP1CB PE=1 SV=3	0.173529337	0.317422958	-0.143893621	0.180486318	0.007457187	0.178858721
Q86VX4	Structural maintenance of chromosomes protein OS=Homo sapiens OX=9606 GN=SMC3 PE=2 SV=1	0.275803846	0.609562105	-0.333758259	0.181848296	0.011092088	0.118716761

P04792	Heat shock protein beta-1 OS=Homo sapiens OX=9606 GN=HSPB1 PE=1 SV=2	0.263293966	0.213070529	0.050223437	0.182194664	0.084493788	0.834276918
A0A087WT47	Troponin I, slow skeletal muscle OS=Homo sapiens OX=9606 GN=TNNI1 PE=1 SV=1	-0.562126991	-0.602615572	0.040488581	0.182548605	0.095306962	0.944474364
B4DVZ8	Leukotriene A(4) hydrolase OS=Homo sapiens OX=9606 PE=2 SV=1	0.12993281	0.082962973	0.046969837	0.184610257	0.086525956	0.99633582
Q9GZR6	Mutant desmin CSM-6 (Fragment) OS=Homo sapiens OX=9606 PE=3 SV=1	0.478178315	0.291724884	0.186453432	0.187863088	0.171956548	0.994421928
K7EQQ3	Keratin, type I cytoskeletal 9 OS=Homo sapiens OX=9606 GN=KRT9 PE=1 SV=1	0.290363539	0.274956829	0.01540671	0.189072361	0.143963665	0.966644408
C9JUN5	Coiled-coil domain-containing protein 12 (Fragment) OS=Homo sapiens OX=9606 GN=CCDC12 PE=1 SV=2	0.339721402	0.510847746	-0.171126345	0.189932692	0.008367492	0.242077619
I3L3Y1	Ketimine reductase mu-crystallin (Fragment) OS=Homo sapiens OX=9606 GN=CRYM PE=1 SV=1	-1.385800769	-0.788458458	-0.597342311	0.193232624	0.348492344	0.52780473
A0A2X0SFH1	PREX1 (Fragment) OS=Homo sapiens OX=9606 GN=PREX1 PE=4 SV=1	-0.366321346	0.028042339	-0.394363685	0.193392735	0.479584094	0.085308075
A0A1B0GV79	KIF-binding protein (Fragment) OS=Homo sapiens OX=9606 GN=KIFBP PE=1 SV=1	0.344300595	0.032068492	0.312232102	0.194074123	0.959197741	0.244090125
Q9BXS1	Isopentenyl-diphosphate delta-isomerase 2 OS=Homo sapiens OX=9606 GN=IDI2 PE=1 SV=1	-0.384475027	-0.740196741	0.355721714	0.199092133	0.004340011	0.152197731
B7Z6Z4	Myosin light polypeptide 6 OS=Homo sapiens OX=9606 GN=MYL6 PE=1 SV=1	0.365132889	0.652837522	-0.287704633	0.199210331	0.007047534	0.132407461

Q8TDR6	Cytoplasmic protein Ndr1 OS=Homo sapiens OX=9606 PE=2 SV=1	0.215074484	0.235975401	-0.020900917	0.199491393	0.089734834	0.787614326
Q12797	Aspartyl/asparaginyl beta-hydroxylase OS=Homo sapiens OX=9606 GN=ASPH PE=1 SV=3	0.309815796	0.384087033	-0.074271237	0.199731278	0.025058535	0.407314841
P02671	Fibrinogen alpha chain OS=Homo sapiens OX=9606 GN=FGA PE=1 SV=2	0.489943965	0.386975416	0.102968549	0.200410948	0.003411815	0.968524719
C9JD32	60S ribosomal protein L23 (Fragment) OS=Homo sapiens OX=9606 GN=RPL23 PE=1 SV=1	-0.402240771	0.223487187	-0.625727958	0.200599689	0.191290759	0.016801577
Q8IUG5	Unconventional myosin-XVIIIb OS=Homo sapiens OX=9606 GN=MYO18B PE=1 SV=2	0.319974632	0.270107334	0.049867298	0.201921851	0.126111713	0.844150327
E7EU96	Casein kinase II subunit alpha OS=Homo sapiens OX=9606 GN=CSNK2A1 PE=1 SV=1	0.23512253	0.153925701	0.081196829	0.201953716	0.58052295	0.482877443
V9HW98	Epididymis luminal protein 2 OS=Homo sapiens OX=9606 GN=HEL2 PE=1 SV=1	0.172176662	0.309834209	-0.137657546	0.205777593	0.027297304	0.219598824
Q9UFN0	Protein NipSnap homolog 3A OS=Homo sapiens OX=9606 GN=NIPSNAP3A PE=1 SV=2	-0.326781591	0.892066684	-1.218848275	0.206063285	0.002888087	0.000181393
B2RXG6	GALNT9 protein OS=Homo sapiens OX=9606 GN=GALNT9 PE=2 SV=1	0.247123702	0.762179691	-0.515055988	0.206088887	0.004207115	0.043726716
A0A384MEE7	Epididymis secretory sperm binding protein OS=Homo sapiens OX=9606 PE=2 SV=1	0.292373426	0.64291041	-0.350536984	0.2062367	0.002995111	0.043304241
P30041	Peroxiredoxin-6 OS=Homo sapiens OX=9606 GN=PRDX6 PE=1 SV=3	0.303553918	-0.14601729	0.449571208	0.207553415	0.420496494	0.06587532

E5RFP6	Voltage-dependent anion-selective channel protein 3 OS=Homo sapiens OX=9606 GN=VDAC3 PE=1 SV=1	-0.304397125	1.111635184	-1.416032308	0.207821967	0.022054575	0.009168439
A0A075X6P5	ATP synthase F0 subunit 8 (Fragment) OS=Homo sapiens OX=9606 GN=ATP8 PE=4 SV=1	-0.197158965	0.296709128	-0.493868093	0.209273048	0.007403862	0.00059714
P62906	60S ribosomal protein L10a OS=Homo sapiens OX=9606 GN=RPL10A PE=1 SV=2	0.204009712	0.428600549	-0.224590837	0.216367948	0.004775564	0.101885158
F8W7Q4	Protein FAM162A OS=Homo sapiens OX=9606 GN=FAM162A PE=1 SV=1	-0.235220283	0.533406429	-0.768626712	0.216464925	0.002887301	6.29835E-05
A0A499FIX8	Extended synaptotagmin-2 OS=Homo sapiens OX=9606 GN=ESYT2 PE=1 SV=1	0.365358526	0.809191708	-0.443833182	0.216815437	0.002827498	0.061376473
A0A0G2JIW1	Heat shock 70 kDa protein 1B OS=Homo sapiens OX=9606 GN=HSPA1B PE=1 SV=1	0.174731738	0.170385157	0.004346581	0.217451879	0.107679887	0.670096946
Q9Y4B3	R29828_1 (Fragment) OS=Homo sapiens OX=9606 PE=4 SV=1	0.487056789	-0.120825068	0.607881857	0.218521118	0.386507138	0.019720857
E9PNK6	Tumor protein D53 OS=Homo sapiens OX=9606 GN=TPD52L1 PE=1 SV=1	0.236910827	0.1484379	0.088472927	0.220222746	0.217058456	0.958950196
V9HWH2	Creatine kinase brain isoform 1 OS=Homo sapiens OX=9606 GN=HEL-S-29 PE=1 SV=1	-0.478718755	-0.092157623	-0.386561133	0.223884517	0.866050935	0.080835001
B4DHC4	cDNA FLJ51843, highly similar to 14-3-3 protein gamma OS=Homo sapiens OX=9606 PE=2 SV=1	0.170263628	0.325429847	-0.15516622	0.224185734	0.011253517	0.171210064
E9PCY7	Heterogeneous nuclear ribonucleoprotein H OS=Homo sapiens OX=9606 GN=HNRNPH1 PE=1 SV=1	0.306318193	0.761969542	-0.455651349	0.226469721	0.003476638	0.068699426

A0A248RGE3	40S ribosomal protein (Fragment) OS=Homo sapiens OX=9606 PE=2 SV=1	0.190723978	0.026554676	0.164169302	0.226639201	0.298723545	0.883407536
Q96EA2	ACOT9 protein OS=Homo sapiens OX=9606 GN=ACOT9 PE=2 SV=1	-0.28970849	0.652954682	-0.942663172	0.226872399	0.007389767	0.000805356
A0A3R5W301	UBA2/WTIP fusion protein OS=Homo sapiens OX=9606 PE=2 SV=1	0.352496218	0.490335203	-0.137838985	0.227737661	0.140686025	0.868320052
Q14204	Cytoplasmic dynein 1 heavy chain 1 OS=Homo sapiens OX=9606 GN=DYNC1H1 PE=1 SV=5	0.254000194	0.499898257	-0.245898063	0.22889177	0.004418575	0.142779599
A0A2R8Y5Q8	Tubulin-specific chaperone E OS=Homo sapiens OX=9606 GN=TBCE PE=1 SV=1	0.222683542	0.516170975	-0.293487433	0.22903747	0.003443434	0.063658533
Q00872	Myosin-binding protein C, slow-type OS=Homo sapiens OX=9606 GN=MYBPC1 PE=1 SV=2	-0.31598345	-0.075458561	-0.24052489	0.230271039	0.564121256	0.145922216
B4DKS0	cDNA FLJ53381, highly similar to Monocarboxylate transporter 1 OS=Homo sapiens OX=9606 PE=2 SV=1	-0.340207452	0.606358603	-0.946566055	0.23044361	0.004433404	0.000333412
Q9BR39	Junctophilin-2 OS=Homo sapiens OX=9606 GN=JPH2 PE=1 SV=2	0.265815404	0.491927779	-0.226112374	0.231757136	0.030680396	0.346677383
P23396	40S ribosomal protein S3 OS=Homo sapiens OX=9606 GN=RPS3 PE=1 SV=2	0.259376172	0.559778629	-0.300402457	0.231878848	0.004368736	0.096545806
E7EMN6	Protein phosphatase inhibitor 2 (Fragment) OS=Homo sapiens OX=9606 GN=PPP1R2 PE=1 SV=1	0.179363723	0.345156507	-0.165792784	0.232064318	0.011285128	0.321963701
Q15149	Plectin OS=Homo sapiens OX=9606 GN=PLEC PE=1 SV=3	0.303539208	0.605794128	-0.30225492	0.23248907	0.045333289	0.301022509

P39019	40S ribosomal protein S19 OS=Homo sapiens OX=9606 GN=RPS19 PE=1 SV=2	0.260595601	0.327122468	-0.066526866	0.232578097	0.201934704	0.90180868
Q6WG76	Antigen MLAA-20 (Fragment) OS=Homo sapiens OX=9606 PE=2 SV=1	-0.773511391	-0.625711673	-0.147799717	0.233283974	0.855519078	0.443469521
P02647	Apolipoprotein A-I OS=Homo sapiens OX=9606 GN=APOA1 PE=1 SV=1	0.306821989	-0.269956751	0.57677874	0.234142038	0.088637722	0.008551988
H9KV28	Protein diaphanous homolog 1 OS=Homo sapiens OX=9606 GN=DIAPH1 PE=1 SV=2	0.380614615	0.429342259	-0.048727644	0.236350469	0.002205565	0.410501566
A0A0G2JSD3	Potassium voltage-gated channel subfamily KQT member 2 (Fragment) OS=Homo sapiens OX=9606 GN=KCNQ2 PE=1 SV=1	-0.224488152	0.542440327	-0.766928479	0.236556008	0.003667519	0.000323505
B4DQ47	cDNA FLJ58036, highly similar to Homo sapiens sterile alpha motif and leucine zipper containing kinase AZK (ZAK), transcript variant 2, mRNA OS=Homo sapiens OX=9606 PE=2 SV=1	0.207940293	0.352125678	-0.144185385	0.237139368	0.010774047	0.194128409
Q53EP4	Dolichyl-diphosphooligosaccharide--protein glycosyltransferase subunit 1 (Fragment) OS=Homo sapiens OX=9606 PE=2 SV=1	0.252836043	0.511898851	-0.259062808	0.23728686	0.011188447	0.121444803
A0A0S2Z4R1	Tyrosine--tRNA ligase (Fragment) OS=Homo sapiens OX=9606 GN=YARS PE=2 SV=1	0.372270869	0.751508168	-0.379237299	0.237935842	0.001240832	0.094576421
V5J3L2	STIM1 OS=Homo sapiens OX=9606 GN=STIM1 PE=2 SV=1	0.30893288	0.618774735	-0.309841855	0.238236766	0.012408621	0.152365223

Q9NZ87	Uncharacterized bone marrow protein BM034 OS=Homo sapiens OX=9606 PE=2 SV=1	0.155819047	0.330708013	-0.174888966	0.238601482	0.012167174	0.088704475
P14854	Cytochrome c oxidase subunit 6B1 OS=Homo sapiens OX=9606 GN=COX6B1 PE=1 SV=2	-0.377297819	-0.068117852	-0.309179967	0.239104422	0.966005292	0.356613495
H3BNX8	Cytochrome c oxidase subunit 5A, mitochondrial OS=Homo sapiens OX=9606 GN=COX5A PE=1 SV=1	-0.461633731	0.24282776	-0.704461491	0.242394339	0.742624106	0.24414057
Q562R1	Beta-actin-like protein 2 OS=Homo sapiens OX=9606 GN=ACTBL2 PE=1 SV=2	-0.690825108	-1.377717985	0.686892877	0.243221331	0.006215664	0.172746673
Q8N766	ER membrane protein complex subunit 1 OS=Homo sapiens OX=9606 GN=EMC1 PE=1 SV=1	0.231679686	0.422111645	-0.190431959	0.243486112	0.007451998	0.143252907
Q1RMG2	Adenosylhomocysteinase OS=Homo sapiens OX=9606 GN=AHCY PE=2 SV=1	0.222471396	0.339954557	-0.117483161	0.248040401	0.008826365	0.228858571
P06732	Creatine kinase M-type OS=Homo sapiens OX=9606 GN=CKM PE=1 SV=2	-0.228800827	0.077242402	-0.306043229	0.250274001	0.990353807	0.323278096
H0YCI4	Nucleosome assembly protein 1-like 4 (Fragment) OS=Homo sapiens OX=9606 GN=NAP1L4 PE=1 SV=1	0.21647922	0.382564825	-0.166085605	0.250582959	0.00780038	0.196256586
B4DUI1	Myoglobin OS=Homo sapiens OX=9606 PE=2 SV=1	0.282181985	-0.396235768	0.678417753	0.250868001	0.032053653	0.001913001
A0A024RB38	SH3 and cysteine rich domain 3, isoform CRA_a OS=Homo sapiens OX=9606 GN=STAC3 PE=4 SV=1	0.293550574	0.445723106	-0.152172531	0.252366619	0.080722802	0.735341559
P21817	Ryanodine receptor 1 OS=Homo sapiens OX=9606 GN=RYR1 PE=1 SV=3	0.189103724	0.409392164	-0.22028844	0.252558817	0.024144089	0.379284277

A0A0S2Z3G9	Actinin alpha 4 isoform 1 (Fragment) OS=Homo sapiens OX=9606 GN=ACTN4 PE=2 SV=1	0.275371675	0.542494414	-0.267122738	0.252624851	0.007409952	0.143046511
B4DV94	cDNA FLJ58285, highly similar to Homo sapiens pre-B-cell leukemia transcription factor interacting protein 1 (PBXIP1), mRNA OS=Homo sapiens OX=9606 PE=2 SV=1	0.220301868	0.403863967	-0.183562099	0.25276208	0.052830528	0.342089951
P25786	Proteasome subunit alpha type-1 OS=Homo sapiens OX=9606 GN=PSMA1 PE=1 SV=1	0.15805365	0.382533351	-0.224479701	0.252866879	0.002990837	0.040986812
A0A024R4X0	NADH-cytochrome b5 reductase OS=Homo sapiens OX=9606 GN=CYB5R3 PE=3 SV=1	0.242033334	0.471447229	-0.229413895	0.256707472	0.014955891	0.172803522
A0A024RCY9	Chromosome 6 open reading frame 130, isoform CRA_b OS=Homo sapiens OX=9606 GN=C6orf130 PE=4 SV=1	0.209068965	0.388465972	-0.179397007	0.257602777	0.010089889	0.210355673
D6REX9	Centrosomal protein of 120 kDa (Fragment) OS=Homo sapiens OX=9606 GN=CEP120 PE=1 SV=2	0.302033175	0.289462476	0.012570699	0.25768578	0.261235583	0.777339846
G3V4Y7	Kinectin OS=Homo sapiens OX=9606 GN=KTN1 PE=1 SV=1	0.272678972	0.582593364	-0.309914392	0.258289231	0.00678825	0.139621724
B4DHE8	RNA-binding protein Musashi homolog 2 OS=Homo sapiens OX=9606 GN=MSI2 PE=1 SV=1	0.217373286	0.448467936	-0.23109465	0.259889247	0.004147564	0.144262509
P30533	Alpha-2-macroglobulin receptor-associated protein OS=Homo sapiens OX=9606 GN=LRPAP1 PE=1 SV=1	0.248678153	0.455531029	-0.206852875	0.260122562	0.038484354	0.308369975
F5H345	Porphobilinogen deaminase OS=Homo	-0.252043818	0.064582156	-0.316625974	0.260387022	0.456928776	0.03535961

	sapiens OX=9606 GN=HMBS PE=1 SV=1						
A0A650F0N3	Beta-globin (Fragment) OS=Homo sapiens OX=9606 PE=4 SV=1	-0.687015922	-0.852546361	0.165530439	0.261116145	0.075166788	0.673252227
Q14974	Importin subunit beta-1 OS=Homo sapiens OX=9606 GN=KPNB1 PE=1 SV=2	0.197890404	0.403642724	-0.205752319	0.261301119	0.002594725	0.107274238
F8VRE9	Coronin (Fragment) OS=Homo sapiens OX=9606 GN=CORO1C PE=1 SV=1	0.374920048	0.475835095	-0.100915047	0.261403169	0.021223768	0.336231017
A0A024R8I5	Family with sequence similarity 69, member B, isoform CRA_a OS=Homo sapiens OX=9606 GN=FAM69B PE=4 SV=1	0.351713151	0.99516699	-0.643453839	0.261490907	0.008573536	0.099026304
P11047	Laminin subunit gamma-1 OS=Homo sapiens OX=9606 GN=LAMC1 PE=1 SV=3	0.281861671	0.641261229	-0.359399558	0.262195226	0.003669249	0.07860507
Q5VTT5	Myomesin-3 OS=Homo sapiens OX=9606 GN=MYOM3 PE=1 SV=1	0.348044349	0.533213236	-0.185168888	0.26237355	0.066257405	0.757768807
H0YHA7	60S ribosomal protein L18 (Fragment) OS=Homo sapiens OX=9606 GN=RPL18 PE=1 SV=1	0.194582748	0.210892521	-0.016309772	0.264165482	0.150168113	0.761435045
Q7KZ24	Nuclease sensitive element binding protein-1 OS=Homo sapiens OX=9606 PE=2 SV=1	-0.260871555	-0.042144136	-0.218727419	0.264499326	0.508113365	0.15824583
Q96AE4	Far upstream element-binding protein 1 OS=Homo sapiens OX=9606 GN=FUBP1 PE=1 SV=3	0.281889777	0.577554499	-0.295664722	0.264520948	0.003368407	0.140854403
B4DUL3	cDNA FLJ53661, highly similar to Mus musculus LIM domain binding 3 (Ldb3), transcript variant 6, mRNA OS=Homo	-0.2153895	0.344022224	-0.559411724	0.264574727	0.041094028	0.015404934

	sapiens OX=9606 PE=2 SV=1						
P31689	DnaJ homolog subfamily A member 1 OS=Homo sapiens OX=9606 GN=DNAJA1 PE=1 SV=2	0.297104314	0.597869937	-0.300765623	0.269411411	0.003998001	0.109300061
Q8TD19	Serine/threonine-protein kinase Nek9 OS=Homo sapiens OX=9606 GN=NEK9 PE=1 SV=2	0.188268976	0.398655006	-0.21038603	0.272379548	0.007745863	0.157388658
K7ENI8	Carbonic anhydrase 4 (Fragment) OS=Homo sapiens OX=9606 GN=CA4 PE=1 SV=1	-0.34164004	0.214775112	-0.556415152	0.2751824	0.19646896	0.06935667
P50238	Cysteine-rich protein 1 OS=Homo sapiens OX=9606 GN=CRIP1 PE=1 SV=3	0.279495725	0.080689567	0.198806158	0.275363952	0.400326297	0.830602102
E9PBS1	Multifunctional protein ADE2 (Fragment) OS=Homo sapiens OX=9606 GN=PAICS PE=1 SV=1	0.202046375	0.277963328	-0.075916953	0.275712717	0.002161086	0.339580293
E1B2D1	Hemoglobin alpha-1 globin chain variant (Fragment) OS=Homo sapiens OX=9606 GN=HBA1 PE=3 SV=1	-0.668616965	-0.615523559	-0.053093406	0.276092421	0.198192315	0.829643886
Q8NDH3	Probable aminopeptidase NPEPL1 OS=Homo sapiens OX=9606 GN=NPEPL1 PE=1 SV=3	0.194351245	0.593681479	-0.399330234	0.27612586	0.00318196	0.034639182
P52179	Myomesin-1 OS=Homo sapiens OX=9606 GN=MYOM1 PE=1 SV=2	0.280749918	0.75621536	-0.475465442	0.27628353	0.016821783	0.198822662
A8K5W7	cDNA FLJ75180, highly similar to Homo sapiens mitochondrial isoleucine tRNA synthetase, mRNA OS=Homo sapiens OX=9606 PE=2 SV=1	-0.209751336	0.585907933	-0.795659269	0.276504405	0.004024519	0.000469284
Q92637	High affinity immunoglobulin gamma Fc receptor IB OS=Homo	-0.313229544	0.075444592	-0.388674135	0.277302883	0.361092829	0.161127576

	sapiens OX=9606 GN=FCGR1B PE=2 SV=1						
P62837	Ubiquitin-conjugating enzyme E2 D2 OS=Homo sapiens OX=9606 GN=UBE2D2 PE=1 SV=1	0.221205091	0.238420707	-0.017215617	0.278028513	0.425943282	0.723525085
Q59GW5	Tripartite motif-containing 25 variant (Fragment) OS=Homo sapiens OX=9606 PE=2 SV=1	0.279023345	0.512898082	-0.233874736	0.279723201	0.003091492	0.143242577
Q0VAK6	Leiomodin-3 OS=Homo sapiens OX=9606 GN=LMOD3 PE=1 SV=1	-0.268450606	0.108496311	-0.376946916	0.281566366	0.126172817	0.043348266
P41227	N-alpha-acetyltransferase 10 OS=Homo sapiens OX=9606 GN=NAA10 PE=1 SV=1	0.302284127	0.544058177	-0.24177405	0.281750586	0.019577413	0.264183899
A4D209	Uncharacterized protein LOC402244 OS=Homo sapiens OX=9606 GN=LOC402244 PE=4 SV=1	0.313782752	-0.429655038	0.74343779	0.282023046	0.023895052	0.000660313
O14558	Heat shock protein beta-6 OS=Homo sapiens OX=9606 GN=HSPB6 PE=1 SV=2	-0.277209598	0.07129047	-0.348500068	0.282450855	0.321689389	0.023750048
P25788	Proteasome subunit alpha type-3 OS=Homo sapiens OX=9606 GN=PSMA3 PE=1 SV=2	0.148219287	0.42194109	-0.273721803	0.282697184	0.003679525	0.032558035
B2RWP4	TACC2 protein OS=Homo sapiens OX=9606 GN=TACC2 PE=1 SV=1	-0.257564942	0.028580797	-0.286145739	0.284541963	0.478044846	0.114125432
A0A384MDP3	Epididymis secretory sperm binding protein OS=Homo sapiens OX=9606 PE=2 SV=1	0.378133291	0.386315419	-0.008182128	0.284634928	0.19601549	0.728323071
P32119	Peroxiredoxin-2 OS=Homo sapiens OX=9606 GN=PRDX2 PE=1 SV=5	-0.298344245	-0.213181435	-0.08516281	0.286244131	0.244364075	0.994287913
K7EJV9	60S ribosomal protein L23a (Fragment) OS=Homo sapiens	-0.225940183	0.021924659	-0.247864841	0.289888512	0.607215034	0.17292084

	OX=9606 GN=RPL23A PE=1 SV=1						
A8K4W0	40S ribosomal protein S3a OS=Homo sapiens OX=9606 GN=RPS3A PE=2 SV=1	0.198052919	0.252123847	-0.054070928	0.291257662	0.019862026	0.601481535
Q5TCQ9	Membrane-associated guanylate kinase, WW and PDZ domain-containing protein 3 OS=Homo sapiens OX=9606 GN=MAGI3 PE=1 SV=3	0.378285398	0.008359119	0.369926279	0.292673943	0.985291932	0.41097579
P02765	Alpha-2-HS-glycoprotein OS=Homo sapiens OX=9606 GN=AHSG PE=1 SV=2	0.441099122	-0.396527404	0.837626525	0.293309159	0.201970815	0.044728196
Q7L7L0	Histone H2A type 3 OS=Homo sapiens OX=9606 GN=H2AW PE=1 SV=3	0.300190258	0.211812117	0.088378141	0.293596597	0.264818543	0.980724114
Q8IWX7	Protein unc-45 homolog B OS=Homo sapiens OX=9606 GN=UNC45B PE=1 SV=1	0.317859749	0.479824972	-0.161965224	0.293883151	0.018146045	0.322662326
P54296	Myomesin-2 OS=Homo sapiens OX=9606 GN=MYOM2 PE=1 SV=2	0.305541621	0.774413237	-0.468871616	0.294350136	0.032245296	0.251040818
A0A024R0S6	EH-domain containing 2, isoform CRA_a OS=Homo sapiens OX=9606 GN=EHD2 PE=3 SV=1	0.279583805	0.823317007	-0.543733202	0.294528685	0.002192332	0.02412336
Q8NBU5	ATPase family AAA domain-containing protein 1 OS=Homo sapiens OX=9606 GN=ATAD1 PE=1 SV=1	0.393789297	0.634070455	-0.240281158	0.298846052	0.004019034	0.442977099
Q13884	Beta-1-syntrophin OS=Homo sapiens OX=9606 GN=SNTB1 PE=1 SV=3	0.325869394	0.539103613	-0.213234219	0.300940266	0.018839265	0.302479118
V9HW32	Epididymis secretory protein Li 298 OS=Homo sapiens OX=9606 GN=HEL-S-298 PE=2 SV=1	0.32540659	0.16537504	0.16003155	0.301161731	0.516449167	0.734324604

A0A0A0MR02	Voltage-dependent anion-selective channel protein 2 (Fragment) OS=Homo sapiens OX=9606 GN=VDAC2 PE=1 SV=1	-0.311538433	0.886305082	-1.197843515	0.302153435	0.010389645	0.003453098
Q4G0E9	Carboxylic ester hydrolase (Fragment) OS=Homo sapiens OX=9606 GN=CES2 PE=2 SV=1	0.270688521	0.702820815	-0.432132294	0.302374285	0.004692829	0.061071627
O76071	Probable cytosolic iron-sulfur protein assembly protein CIAO1 OS=Homo sapiens OX=9606 GN=CIAO1 PE=1 SV=1	0.144460277	0.417256018	-0.272795742	0.302519242	0.006212461	0.065803364
P62424	60S ribosomal protein L7a OS=Homo sapiens OX=9606 GN=RPL7A PE=1 SV=2	0.228030179	0.248942406	-0.020912227	0.302780976	0.064669141	0.558477876
B2R491	40S ribosomal protein S4 OS=Homo sapiens OX=9606 GN=RPS4X PE=2 SV=1	0.241646909	0.659399058	-0.417752149	0.302885842	0.005787444	0.06288702
P25325	3-mercaptopyruvate sulfurtransferase OS=Homo sapiens OX=9606 GN=MPST PE=1 SV=3	-0.157321743	0.123166918	-0.280488661	0.304495866	0.123308963	0.012567685
Q6IAZ9	NDUFC1 protein OS=Homo sapiens OX=9606 GN=NDUFC1 PE=2 SV=1	-0.293107082	0.513268555	-0.806375636	0.310195275	0.019383025	0.000665565
P55060	Exportin-2 OS=Homo sapiens OX=9606 GN=CSE1L PE=1 SV=3	0.158159914	0.299060213	-0.140900298	0.310259285	0.004914457	0.157539241
Q59FD4	Hexokinase 1 isoform HKI variant (Fragment) OS=Homo sapiens OX=9606 PE=2 SV=1	-0.248059888	0.395347272	-0.64340716	0.310938505	0.059691361	0.003059864
Q6P587	Acylpyruvase FAHD1, mitochondrial OS=Homo sapiens OX=9606 GN=FAHD1 PE=1 SV=2	-0.261862821	0.55693278	-0.818795601	0.314864092	0.009166345	9.35938E-05
B4DDN9	cDNA FLJ60412, highly similar to Leucine-rich repeat protein SHOC-2	0.292401568	0.856278953	-0.563877385	0.315289723	0.002586198	0.046398652

	OS=Homo sapiens OX=9606 PE=2 SV=1						
Q562T9	Actin-like protein (Fragment) OS=Homo sapiens OX=9606 GN=ACT PE=4 SV=1	-0.393822015	-0.502906645	0.10908463	0.316857908	0.337069025	0.792712077
Q7Z532	Osteoglycin OG OS=Homo sapiens OX=9606 PE=2 SV=1	0.324316923	0.644439676	-0.320122752	0.321195559	0.008402518	0.175103362
Q13228	Methanethiol oxidase OS=Homo sapiens OX=9606 GN=SELENBP1 PE=1 SV=2	-0.169982144	-0.232974851	0.062992707	0.32478143	0.244298443	0.957489089
Q53TH1	Uncharacterized protein PSMD14 (Fragment) OS=Homo sapiens OX=9606 GN=PSMD14 PE=4 SV=1	0.17324259	0.396046748	-0.222804158	0.324798058	0.017594084	0.18371108
Q8TB95	Rab GDP dissociation inhibitor (Fragment) OS=Homo sapiens OX=9606 PE=2 SV=1	0.18524124	0.243135691	-0.057894451	0.326754135	0.080097681	0.707783112
A8KA83	Epididymis secretory sperm binding protein OS=Homo sapiens OX=9606 PE=1 SV=1	0.16858663	0.366263222	-0.197676591	0.327171474	0.013496666	0.112932167
P46926	Glucosamine-6-phosphate isomerase 1 OS=Homo sapiens OX=9606 GN=GNPDA1 PE=1 SV=1	0.167895762	0.274638103	-0.106742341	0.330812489	0.011016984	0.212975942
O00232	26S proteasome non-ATPase regulatory subunit 12 OS=Homo sapiens OX=9606 GN=PSMD12 PE=1 SV=3	0.22502199	0.461717044	-0.236695053	0.336434362	0.013966371	0.143345297
B4DRH6	cDNA FLJ54509, highly similar to Trifunctional enzyme subunit alpha, mitochondrial OS=Homo sapiens OX=9606 PE=2 SV=1	-0.182809728	0.529052741	-0.711862469	0.336505915	0.007421982	0.001096694
Q53GY0	Plastin 3 variant (Fragment) OS=Homo sapiens OX=9606 PE=2 SV=1	0.241267832	0.639507585	-0.398239753	0.337470938	0.003186038	0.045156933

A0A024R1T9	ATP-citrate synthase OS=Homo sapiens OX=9606 GN=ACLY PE=3 SV=1	0.182177926	0.463645227	-0.281467301	0.337910728	0.007437165	0.087643923
Q15369	Elongin-C OS=Homo sapiens OX=9606 GN=ELOC PE=1 SV=1	0.123070512	0.277724656	-0.154654144	0.339681318	0.029849105	0.185432808
A0A087X090	Farnesyl diphosphate synthase (Farnesyl pyrophosphate synthetase, dimethylallyltranstransferase, geranyltranstransferase), isoform CRA_a OS=Homo sapiens OX=9606 GN=FDPS PE=1 SV=1	0.188347353	0.260442002	-0.072094649	0.339812464	0.009306565	0.335413216
P61201	COP9 signalosome complex subunit 2 OS=Homo sapiens OX=9606 GN=COPS2 PE=1 SV=1	0.133911034	0.360752941	-0.226841907	0.340077061	0.011673773	0.11021
A0A0U1RQE8	Putative glycine N-acyltransferase-like protein 1B OS=Homo sapiens OX=9606 GN=GLYATL1B PE=3 SV=1	0.280130482	0.430897097	-0.150766614	0.341542126	0.074765154	0.410951833
A0A024RD80	Heat shock protein 90kDa alpha (Cytosolic), class B member 1, isoform CRA_a OS=Homo sapiens OX=9606 GN=HSP90AB1 PE=3 SV=1	0.179314321	0.339872587	-0.160558267	0.344894243	0.011177068	0.254567585
Q96DP0	cDNA FLJ31479 fis, clone NT2NE2001634, moderately similar to NADH-UBIQUINONE OXIDOREDUCTASE 9 KD SUBUNIT OS=Homo sapiens OX=9606 PE=2 SV=1	0.257444513	0.529401421	-0.271956908	0.345360683	0.003118354	0.099453929
Q7KYX9	PFKM protein OS=Homo sapiens OX=9606 GN=PFKM PE=2 SV=1	0.2361407	0.242070602	-0.005929902	0.345496658	0.135147314	0.657972846

P55039	Developmentally-regulated GTP-binding protein 2 OS=Homo sapiens OX=9606 GN=DRG2 PE=1 SV=1	0.153911926	0.645969771	-0.492057845	0.345562624	0.000949185	0.00264871
A8MUD9	60S ribosomal protein L7 OS=Homo sapiens OX=9606 GN=RPL7 PE=1 SV=1	0.19535112	0.292278714	-0.096927594	0.346539714	0.032626115	0.435114917
A0A140VK02	Testis tissue sperm-binding protein Li 35a OS=Homo sapiens OX=9606 PE=2 SV=1	0.291440601	0.003309633	0.288130968	0.350192871	0.481751274	0.888340975
P11216	Glycogen phosphorylase, brain form OS=Homo sapiens OX=9606 GN=PYGB PE=1 SV=5	0.214709057	0.489156453	-0.274447396	0.350224219	0.031992912	0.221795655
Q13043	Serine/threonine-protein kinase 4 OS=Homo sapiens OX=9606 GN=STK4 PE=1 SV=2	0.251681454	-0.332180255	0.583861709	0.350392078	0.098229477	0.000941427
Q8N142	Adenylosuccinate synthetase isozyme 1 OS=Homo sapiens OX=9606 GN=ADSS1 PE=1 SV=1	-0.13029005	0.478121829	-0.608411879	0.351495472	0.003237397	0.001894823
P78356	Phosphatidylinositol 5-phosphate 4-kinase type-2 beta OS=Homo sapiens OX=9606 GN=PIP4K2B PE=1 SV=1	0.160392379	0.546380696	-0.385988317	0.351551275	0.007741316	0.067231566
Q3LIE7	cDNA FLJ55321, highly similar to 24-dehydrocholesterol reductase OS=Homo sapiens OX=9606 GN=Nbla03646 PE=1 SV=1	0.181976141	0.480962761	-0.29898662	0.357507745	0.013605468	0.157300437
Q53FS6	TNF receptor-associated protein 1 variant (Fragment) OS=Homo sapiens OX=9606 PE=2 SV=1	-0.170330282	0.504262298	-0.674592581	0.358504917	0.007570518	0.00074245

P13645	Keratin, type I cytoskeletal 10 OS=Homo sapiens OX=9606 GN=KRT10 PE=1 SV=6	0.18786935 3	0.26489 748	- 0.077028 127	0.3593 73557	0.1859 54125	0.8119 5362
Q6FHU 2	Phosphoglycerate mutase (Fragment) OS=Homo sapiens OX=9606 GN=PGAM1 PE=2 SV=1	0.17319391 6	0.19316 2411	- 0.019968 495	0.3594 27258	0.0866 53982	0.6955 57124
Q96AX1	Vacuolar protein sorting-associated protein 33A OS=Homo sapiens OX=9606 GN=VPS33A PE=1 SV=1	0.26088804 1	- 0.52144 4026	0.782332 068	0.3595 59824	0.0419 34364	0.0048 30933
Q9GZS3	WD repeat-containing protein 61 OS=Homo sapiens OX=9606 GN=WDR61 PE=1 SV=1	0.19458080 4	0.21493 7726	- 0.020356 922	0.3595 8965	0.3215 09099	0.9439 3182
C9JUP7	Transitional endoplasmic reticulum ATPase (Fragment) OS=Homo sapiens OX=9606 GN=VCP PE=1 SV=1	0.23654898 6	0.41365 7029	- 0.177108 043	0.3597 45047	0.0171 36324	0.2760 14792
E9PKE3	Heat shock cognate 71 kDa protein OS=Homo sapiens OX=9606 GN=HSPA8 PE=1 SV=1	0.10751541	0.39669 9723	- 0.289184 313	0.3597 63603	0.0046 93088	0.0459 33028
A8K503	cDNA FLJ76182, highly similar to Homo sapiens ubiquitin-conjugating enzyme E2I (UBC9 homolog, yeast) (UBE2I), transcript variant 1, mRNA OS=Homo sapiens OX=9606 PE=2 SV=1	0.18007636 5	0.41475 2904	- 0.234676 538	0.3597 98854	0.0065 57042	0.0806 87672
Q9H1R 3	Myosin light chain kinase 2, skeletal/cardiac muscle OS=Homo sapiens OX=9606 GN=MYLK2 PE=1 SV=3	- 0.24837354 4	0.01404 8179	- 0.262421 723	0.3598 2616	0.8823 11302	0.4809 75375
Q5JXI8	Four and a half LIM domains protein 1 (Fragment) OS=Homo	0.23413847 3	- 0.17627 0882	0.410409 356	0.3613 16604	0.2816 49069	0.0952 66274

	sapiens OX=9606 GN=FHL1 PE=1 SV=1						
J3QRU8	ARF GTPase-activating protein GIT1 OS=Homo sapiens OX=9606 GN=GIT1 PE=1 SV=1	0.190597842	0.243387066	-0.052789224	0.362836844	0.076730682	0.481950734
H0YBI4	PDZ and LIM domain protein 5 (Fragment) OS=Homo sapiens OX=9606 GN=PDLIM5 PE=1 SV=1	-0.219121882	0.341135343	-0.560257225	0.363331468	0.169143983	0.080217228
B4DVR4	cDNA FLJ60912, highly similar to Vinexin OS=Homo sapiens OX=9606 PE=2 SV=1	0.243229437	0.509505447	-0.26627601	0.364849796	0.014105781	0.201112878
P13639	Elongation factor 2 OS=Homo sapiens OX=9606 GN=EEF2 PE=1 SV=4	0.154370481	0.336103157	-0.181732675	0.365964375	0.007797621	0.157333724
B4DKG7	cDNA FLJ60633, highly similar to Homo sapiens secernin 3 (SCRN3), mRNA OS=Homo sapiens OX=9606 PE=2 SV=1	0.141261099	0.303141231	-0.161880132	0.369645405	0.036902447	0.308472414
A0A5F9ZHX4	Peregrin OS=Homo sapiens OX=9606 GN=BRPF1 PE=1 SV=1	-0.229065097	0.73097661	-0.960041707	0.370353018	0.00437818	0.000210806
E2RVJ0	Anion exchange protein OS=Homo sapiens OX=9606 GN=SLC4A1 PE=2 SV=1	-0.346701438	-0.064710032	-0.281991406	0.371646754	0.790448766	0.589085491
Q8N5Y3	GYG1 protein (Fragment) OS=Homo sapiens OX=9606 GN=GYG1 PE=2 SV=2	-0.309945692	-0.960819379	0.650873687	0.371680018	0.003831967	0.099362786
A0A2R8Y7Z0	Catenin beta-1 OS=Homo sapiens OX=9606 GN=CTNNB1 PE=1 SV=1	0.163494823	0.330758085	-0.167263261	0.371999848	0.027359176	0.212531454
O14949	Cytochrome b-c1 complex subunit 8 OS=Homo sapiens OX=9606 GN=UQCRQ PE=1 SV=4	-0.202763874	0.245912008	-0.448675882	0.375982002	0.287960584	0.119325772

A0A1W2PQL4	Uncharacterized protein OS=Homo sapiens OX=9606 PE=4 SV=1	-0.186608846	0.511224104	-0.69783295	0.382089116	0.016517694	0.004836818
A0A5C2GHK5	IG c672_light_IGKV1-16_IGKJ4 (Fragment) OS=Homo sapiens OX=9606 PE=2 SV=1	0.363049206	-0.264533354	0.62758256	0.383074887	0.824908899	0.376730262
E5RI99	60S ribosomal protein L30 (Fragment) OS=Homo sapiens OX=9606 GN=RPL30 PE=1 SV=1	0.219323308	0.205062358	0.01426095	0.383934027	0.081383141	0.667410776
P54652	Heat shock-related 70 kDa protein 2 OS=Homo sapiens OX=9606 GN=HSPA2 PE=1 SV=1	0.209144297	0.201714038	0.007430259	0.384241012	0.459772376	0.821244987
K9JA46	Epididymis luminal secretory protein 52 OS=Homo sapiens OX=9606 GN=EL52 PE=2 SV=1	0.162171684	0.353614592	-0.191442908	0.384415788	0.011420164	0.159942133
Q9NRN7	L-aminoadipate-semialdehyde dehydrogenase-phosphopantetheinyl transferase OS=Homo sapiens OX=9606 GN=AASDHPPT PE=1 SV=2	0.117025427	0.056505827	0.0605196	0.384435525	0.257717337	0.950398828
P35609	Alpha-actinin-2 OS=Homo sapiens OX=9606 GN=ACTN2 PE=1 SV=1	-0.151899672	-0.09037393	-0.061525742	0.384507647	0.7644394	0.3947891
A0A2R8Y6L5	Dual-specificity tyrosine-phosphorylation-regulated kinase 1A (Fragment) OS=Homo sapiens OX=9606 GN=DYRK1A PE=1 SV=1	0.193427093	0.447760974	-0.254333882	0.385954611	0.019797255	0.099688502
Q9BS10	Similar to ribosomal protein S8 (Fragment) OS=Homo sapiens OX=9606 PE=2 SV=1	0.172333926	0.486965453	-0.314631527	0.388396491	0.005485121	0.056812077
D9ZGF2	Collagen, type VI, alpha 3 OS=Homo sapiens OX=9606 GN=COL6A3 PE=2 SV=1	0.347744218	1.069627019	-0.721882801	0.389381695	0.008993772	0.067473681

Q5QTS3	FWP004 OS=Homo sapiens OX=9606 PE=2 SV=1	0.273660533	0.521884375	-0.248223843	0.389833118	0.0101791	0.16987188
P50990	T-complex protein 1 subunit theta OS=Homo sapiens OX=9606 GN=CCT8 PE=1 SV=4	0.212285601	0.389221033	-0.176935432	0.393032577	0.010854933	0.247547376
H0YN18	Proteasome endopeptidase complex OS=Homo sapiens OX=9606 GN=PSMA4 PE=1 SV=1	0.13640538	0.426780584	-0.290375204	0.394607807	0.00518837	0.037790688
B3KU09	cDNA FLJ39034 fis, clone NT2RP7008085, highly similar to Homo sapiens ring finger protein 123 (RNF123), mRNA OS=Homo sapiens OX=9606 PE=2 SV=1	-0.134891483	-0.009980941	-0.124910541	0.394735349	0.414765594	0.179542764
O95817	BAG family molecular chaperone regulator 3 OS=Homo sapiens OX=9606 GN=BAG3 PE=1 SV=3	0.143902902	0.455794692	-0.31189179	0.396708934	0.00539026	0.020201478
A0A024RDB9	NAD(P)(+)--arginine ADP-ribosyltransferase OS=Homo sapiens OX=9606 GN=ART3 PE=3 SV=1	0.21719117	0.760755615	-0.543564445	0.397612026	0.003363991	0.058419252
Q5QTR4	MSTP002 OS=Homo sapiens OX=9606 PE=2 SV=1	-0.217569952	0.316392181	-0.533962133	0.397755165	0.052788482	0.016016066
A0A2R8Y5S7	Radixin OS=Homo sapiens OX=9606 GN=RDX PE=1 SV=1	0.137504909	0.340585269	-0.20308036	0.39799841	0.116654017	0.44774311
B4DXW2	cDNA FLJ60947, highly similar to Coiled-coil domain-containing protein 9 OS=Homo sapiens OX=9606 PE=2 SV=1	0.294698276	0.872433383	-0.577735108	0.39808836	0.022556912	0.186308986
A0A0U1RQH4	EH domain-binding protein 1-like protein 1 (Fragment) OS=Homo sapiens OX=9606 GN=EHBP1L1 PE=1 SV=1	0.261584926	0.688917751	-0.427332825	0.401716413	0.007417835	0.107527777

B4DJU4	cDNA FLJ53344, highly similar to Splicing factor 1 OS=Homo sapiens OX=9606 PE=2 SV=1	0.228780252	0.347050132	-0.11826988	0.402010915	0.022542477	0.325790296
P17174	Aspartate aminotransferase, cytoplasmic OS=Homo sapiens OX=9606 GN=GOT1 PE=1 SV=3	-0.118097079	0.024140123	-0.142237202	0.404134919	0.973254786	0.498505293
V9HW92	Epididymis secretory protein Li 112 OS=Homo sapiens OX=9606 GN=HEL-S-112 PE=2 SV=1	-0.190582233	-0.356525323	0.165943091	0.406004494	0.310996087	0.955570871
P08237	ATP-dependent 6-phosphofructokinase, muscle type OS=Homo sapiens OX=9606 GN=PFKM PE=1 SV=2	0.237651119	0.417441308	-0.179790189	0.407812272	0.078267914	0.471266814
A0A024R702	Brain specific protein, isoform CRA_a OS=Homo sapiens OX=9606 GN=CGI-38 PE=4 SV=1	0.299172694	0.092121688	0.207051006	0.408987944	0.436860188	0.801990713
P51452	Dual specificity protein phosphatase 3 OS=Homo sapiens OX=9606 GN=DUSP3 PE=1 SV=1	0.105134074	0.211863704	-0.10672963	0.411541504	0.062347761	0.338473519
Q99832	T-complex protein 1 subunit eta OS=Homo sapiens OX=9606 GN=CCT7 PE=1 SV=2	0.159217839	0.41223966	-0.253021821	0.411743647	0.006236896	0.093453667
T2DN57	DNAJA2/NETO2 fusion protein 2 OS=Homo sapiens OX=9606 PE=2 SV=1	0.180822549	0.554080829	-0.37325828	0.411745927	0.007405379	0.09684444
Q9Y6N9	Harmonin OS=Homo sapiens OX=9606 GN=USH1C PE=1 SV=3	0.241606175	0.466334757	-0.224728581	0.411809465	0.017927578	0.373903244
Q711Q0	Cardiac-enriched FHL2-interacting protein OS=Homo sapiens OX=9606 GN=CEFIP PE=1 SV=2	-0.14300813	0.47203313	-0.615041261	0.414400269	0.005355695	0.002260968

A5D6W6	Fat storage-inducing transmembrane protein 1 OS=Homo sapiens OX=9606 GN=FITM1 PE=2 SV=1	-0.179943624	0.414609811	-0.594553435	0.414636093	0.006601897	0.002350678
P13929	Beta-enolase OS=Homo sapiens OX=9606 GN=ENO3 PE=1 SV=5	0.283679538	0.458066582	-0.174387044	0.414768229	0.22449563	0.96592884
E9PAV3	Nascent polypeptide-associated complex subunit alpha, muscle-specific form OS=Homo sapiens OX=9606 GN=NACA PE=1 SV=1	0.184282967	0.630180779	-0.445897813	0.414774304	0.004129516	0.034961677
Q7Z675	Calcium-transporting ATPase OS=Homo sapiens OX=9606 GN=DKFZp779O2152 PE=2 SV=1	0.379341681	0.504623179	-0.125281498	0.415064838	0.221555134	0.863044649
A8K3H8	cDNA FLJ77680, highly similar to Homo sapiens protein phosphatase 2 (formerly 2A), regulatory subunit A (PR 65), alpha isoform (PPP2R1A), mRNA OS=Homo sapiens OX=9606 PE=2 SV=1	0.152469969	0.176605676	-0.024135707	0.416085007	0.177828761	0.85932546
Q6FHV6	ENO2 protein OS=Homo sapiens OX=9606 GN=ENO2 PE=1 SV=1	0.138371677	0.351476735	-0.213105058	0.418485957	0.020918091	0.272790095
A0A0S2Z3L2	ATPase Ca++ transporting cardiac muscle slow twitch 2 isoform 1 (Fragment) OS=Homo sapiens OX=9606 GN=ATP2A2 PE=2 SV=1	-0.147505651	0.384997425	-0.532503076	0.418650393	0.026638326	0.016578276
A0A087WVI1	PDZ and LIM domain protein 2 OS=Homo sapiens OX=9606 GN=PDLIM2 PE=1 SV=1	-0.246228202	-0.467161941	0.22093374	0.418979079	0.068489642	0.703259954
A0A1B1PFW5	Unc-45 myosin chaperone B (Fragment) OS=Homo sapiens OX=9606 GN=UNC45B PE=4 SV=1	0.171026638	0.389360401	-0.218333763	0.42690377	0.014973714	0.169334707

P68871	Hemoglobin subunit beta OS=Homo sapiens OX=9606 GN=HBB PE=1 SV=2	-0.443474532	-0.430705831	-0.0127687	0.427597114	0.266345267	0.883137353
H7C5U8	39S ribosomal protein L27, mitochondrial (Fragment) OS=Homo sapiens OX=9606 GN=MRPL27 PE=1 SV=1	-0.172798125	0.733703779	-0.906501904	0.427619144	0.001326872	4.84037E-05
H0Y4R1	Inosine-5'-monophosphate dehydrogenase 2 (Fragment) OS=Homo sapiens OX=9606 GN=IMPDH2 PE=1 SV=1	0.182076918	0.346812175	-0.164735257	0.428572702	0.038377919	0.166239262
A0A140VK93	Adenylate kinase 2, mitochondrial OS=Homo sapiens OX=9606 GN=AK2 PE=2 SV=1	-0.178965998	0.772083179	-0.951049177	0.428727841	0.002752798	0.000123959
A0A0K2BMD8	Mutant hemoglobin alpha 2 globin chain OS=Homo sapiens OX=9606 GN=HBA2 PE=3 SV=1	-0.519125459	-0.454438805	-0.064686654	0.429005274	0.288322883	0.899755531
A0A0S2Z4I4	Tropomyosin 3 isoform 3 (Fragment) OS=Homo sapiens OX=9606 GN=TPM3 PE=2 SV=1	-0.211636023	-0.455756438	0.244120414	0.429737857	0.415438512	0.94387044
A0A0C4DH48	Nesprin-1 (Fragment) OS=Homo sapiens OX=9606 GN=SYNE1 PE=1 SV=1	0.258326633	0.401580694	-0.143254061	0.432465555	0.240814025	0.946284597
Q53SB5	Desmin, isoform CRA_a OS=Homo sapiens OX=9606 GN=tmp_locus_29 PE=3 SV=1	0.216703665	0.431458863	-0.214755197	0.432612768	0.065253296	0.323171911
Q96DG6	Carboxymethylenebutenolidase homolog OS=Homo sapiens OX=9606 GN=CMBL PE=1 SV=1	0.160398946	-0.339696774	0.50009572	0.433191652	0.031987032	0.005177009
A0A2R8Y849	40S ribosomal protein S24 OS=Homo sapiens OX=9606 GN=RPS24 PE=1 SV=1	0.162656133	0.379071087	-0.216414954	0.433363268	0.024883563	0.17320574

P31415	Calsequestrin-1 OS=Homo sapiens OX=9606 GN=CASQ1 PE=1 SV=3	0.282828482	0.611485995	-0.328657512	0.436727408	0.051566798	0.242518192
H0YLY7	Calcineurin B homologous protein 1 (Fragment) OS=Homo sapiens OX=9606 GN=CHP1 PE=1 SV=1	0.152784088	0.445790983	-0.293006895	0.436742283	0.018402727	0.063516438
Q96B43	Methionine aminopeptidase (Fragment) OS=Homo sapiens OX=9606 PE=2 SV=1	0.149978464	0.309373121	-0.159394658	0.437801228	0.15634931	0.548409875
B4DEA8	cDNA FLJ56425, highly similar to Very-long-chain specific acyl-CoAdehydrogenase, mitochondrial OS=Homo sapiens OX=9606 PE=2 SV=1	-0.146562549	0.692360113	-0.838922662	0.439898499	0.00219444	0.00084107
Q08J23	RNA cytosine C(5)-methyltransferase NSUN2 OS=Homo sapiens OX=9606 GN=NSUN2 PE=1 SV=2	0.172876209	0.391198418	-0.21832221	0.440359269	0.022477235	0.109367196
B4DFL1	Dihydrolipoyl dehydrogenase OS=Homo sapiens OX=9606 PE=2 SV=1	-0.1288391	0.402078776	-0.530917877	0.445321809	0.03834581	0.018765705
E5RHE9	Inositol monophosphatase 1 OS=Homo sapiens OX=9606 GN=IMPA1 PE=1 SV=1	0.130379142	-0.053580943	0.183960085	0.446881219	0.682431999	0.131399282
H7C125	Ras-related protein Rab-2A (Fragment) OS=Homo sapiens OX=9606 GN=RAB2A PE=1 SV=1	0.151499055	0.338848412	-0.187349357	0.446932617	0.006617225	0.099468428
V9HWB7	Epididymis luminal protein 60 OS=Homo sapiens OX=9606 GN=HEL60 PE=2 SV=1	0.106207192	0.147322175	-0.041114983	0.447065163	0.104373071	0.568204094
A0A140VJR3	Phosphoglycerate kinase OS=Homo sapiens OX=9606 PE=2 SV=1	0.198483138	0.25220662	-0.053723482	0.447075007	0.191789845	0.753753319

A0A0A0MT01	Gelsolin OS=Homo sapiens OX=9606 GN=GSN PE=1 SV=1	0.366912741	-0.275684149	0.64259689	0.447387651	0.990924844	0.520434522
B2RDN9	cDNA, FLJ96699, highly similar to Homo sapiens thyroid autoantigen 70kDa (Ku antigen) (G22P1), mRNA OS=Homo sapiens OX=9606 PE=2 SV=1	0.237021997	0.481874944	-0.244852947	0.447791751	0.011961936	0.189766726
A0A0S2Z614	Minichromosome maintenance complex binding protein isoform 3 OS=Homo sapiens OX=9606 GN=MCMBP PE=2 SV=1	-0.298414142	-0.375845033	0.077430891	0.44855971	0.162012919	0.735059411
P07951	Tropomyosin beta chain OS=Homo sapiens OX=9606 GN=TPM2 PE=1 SV=1	-0.428612096	-0.629501969	0.200889873	0.448621431	0.119337297	0.88059018
Q9Y680	Peptidyl-prolyl cis-trans isomerase FKBP7 OS=Homo sapiens OX=9606 GN=FKBP7 PE=1 SV=2	0.154161215	0.585921784	-0.431760569	0.448730123	0.005381303	0.017303582
H6VRF8	Keratin 1 OS=Homo sapiens OX=9606 GN=KRT1 PE=3 SV=1	0.237598602	0.116798454	0.120800148	0.449399817	0.827709622	0.498235602
Q08257	Quinone oxidoreductase OS=Homo sapiens OX=9606 GN=CRYZ PE=1 SV=1	0.137743115	0.385693607	-0.247950492	0.454934286	0.03338818	0.150486305
P30154	Serine/threonine-protein phosphatase 2A 65 kDa regulatory subunit A beta isoform OS=Homo sapiens OX=9606 GN=PPP2R1B PE=1 SV=3	-0.544368483	-0.324908026	-0.219460457	0.45527696	0.497002923	0.952359554
Q14214	Nebulin (Fragment) OS=Homo sapiens OX=9606 PE=2 SV=1	0.258313341	0.572187347	-0.313874006	0.45532697	0.058431146	0.341989795
B7U472	High molecular weight microtubule-associated protein tau (Fragment) OS=Homo sapiens OX=9606 PE=2 SV=1	0.271657541	0.123400339	0.148257202	0.456424127	0.776841729	0.597190895

H7C123	60S ribosomal protein L10 (Fragment) OS=Homo sapiens OX=9606 GN=RPL10 PE=1 SV=1	0.133620818	0.314543255	-0.180922437	0.457595825	0.041906687	0.118571221
Q6P442	HMGXB3 protein (Fragment) OS=Homo sapiens OX=9606 GN=HMGXB3 PE=2 SV=1	-0.183575665	-0.13565122	-0.047924445	0.458352653	0.805805563	0.470773618
P00338	L-lactate dehydrogenase A chain OS=Homo sapiens OX=9606 GN=LDHA PE=1 SV=2	0.356692836	0.466399612	-0.109706776	0.458820719	0.255774414	0.898153406
Q9NP72	Ras-related protein Rab-18 OS=Homo sapiens OX=9606 GN=RAB18 PE=1 SV=1	0.148304191	0.573740157	-0.425435966	0.461258132	0.006747653	0.045229053
Q13619	Cullin-4A OS=Homo sapiens OX=9606 GN=CUL4A PE=1 SV=3	0.111293849	0.472558145	-0.361264296	0.46463435	0.006059172	0.047224037
B4DLP4	Ribosomal protein L15 OS=Homo sapiens OX=9606 PE=2 SV=1	0.203451183	0.240396217	-0.036945034	0.464848117	0.20535495	0.703512509
A0A1B0GUA3	KIF-binding protein OS=Homo sapiens OX=9606 GN=KIFBP PE=1 SV=1	0.145277985	0.362446129	-0.217168145	0.467405092	0.031177736	0.065869033
H7C2W9	60S ribosomal protein L31 (Fragment) OS=Homo sapiens OX=9606 GN=RPL31 PE=1 SV=1	0.214500835	0.544818715	-0.33031788	0.468062257	0.016888455	0.11865833
Q96C01	Protein FAM136A OS=Homo sapiens OX=9606 GN=FAM136A PE=1 SV=1	-0.12453556	0.520223431	-0.644758991	0.468872755	0.004825253	0.002967491
B7Z1V9	cDNA FLJ53310, highly similar to Puromycin-sensitive aminopeptidase OS=Homo sapiens OX=9606 PE=2 SV=1	-0.158364976	-0.301716066	0.14335109	0.472823413	0.062908496	0.310815906
O60662	Kelch-like protein 41 OS=Homo sapiens OX=9606 GN=KLHL41 PE=1 SV=2	-0.18957465	0.398098866	-0.587673515	0.47837344	0.01495669	0.017306118

A0A024R5Z9	Pyruvate kinase OS=Homo sapiens OX=9606 GN=PKM2 PE=3 SV=1	0.18071267	0.355465583	-0.174752913	0.478638772	0.173693545	0.793208506
P23109	AMP deaminase 1 OS=Homo sapiens OX=9606 GN=AMPD1 PE=1 SV=2	0.12428295	0.427449138	-0.303166189	0.480590722	0.017009469	0.132394668
Q96S19	Methyltransferase-like 26 OS=Homo sapiens OX=9606 GN=METTL26 PE=1 SV=2	0.128079169	0.11113107	0.016948099	0.480629448	0.473123859	0.988655828
P40123	Adenylyl cyclase-associated protein 2 OS=Homo sapiens OX=9606 GN=CAP2 PE=1 SV=1	0.156564143	0.472351043	-0.3157869	0.483148066	0.008573037	0.111159017
O43765	Small glutamine-rich tetratricopeptide repeat-containing protein alpha OS=Homo sapiens OX=9606 GN=SGTA PE=1 SV=1	0.10918003	0.413235145	-0.304055115	0.483370927	0.002111475	0.01130062
A0A2R8Y5Y7	60S ribosomal protein L9 OS=Homo sapiens OX=9606 GN=RPL9 PE=1 SV=1	0.202678894	0.472983833	-0.270304939	0.483589768	0.018648416	0.190032984
P27338	Amine oxidase [flavin-containing] B OS=Homo sapiens OX=9606 GN=MAOB PE=1 SV=3	-0.147087236	0.035978341	-0.183065576	0.488111575	0.748358952	0.354012378
P35237	Serpin B6 OS=Homo sapiens OX=9606 GN=SERPINB6 PE=1 SV=3	0.092115104	0.238402094	-0.14628699	0.490241824	0.089938878	0.335488901
Q9UKV8	Protein argonaute-2 OS=Homo sapiens OX=9606 GN=AGO2 PE=1 SV=3	0.142132976	0.404755997	-0.26262302	0.490496375	0.007417054	0.090469248
Q5XTR9	Hemoglobin delta-beta fusion protein (Fragment) OS=Homo sapiens OX=9606 GN=HBD/HBB PE=3 SV=1	-0.455683134	-0.185736508	-0.269946626	0.490710024	0.927284589	0.63024581

D6RA82	Annexin OS=Homo sapiens OX=9606 GN=ANXA3 PE=1 SV=1	0.162824131	0.803858037	-0.641033906	0.490818095	0.000983669	0.019662087
B4DNL3	cDNA FLJ53145, highly similar to Ankycorbin (Fragment) OS=Homo sapiens OX=9606 PE=2 SV=1	0.385884911	0.162846021	0.22303889	0.491531584	0.909216539	0.516455077
P19823	Inter-alpha-trypsin inhibitor heavy chain H2 OS=Homo sapiens OX=9606 GN=ITIH2 PE=1 SV=2	0.260616743	-0.122601677	0.383218419	0.493771116	0.821620903	0.372436451
Q02127	Dihydroorotate dehydrogenase (quinone), mitochondrial OS=Homo sapiens OX=9606 GN=DHODH PE=1 SV=3	-0.139384823	0.578574156	-0.717958979	0.499683158	0.003941297	0.000743747
O00757	Fructose-1,6-bisphosphatase isozyme 2 OS=Homo sapiens OX=9606 GN=FBP2 PE=1 SV=2	0.181102452	0.496284128	-0.315181676	0.500522125	0.028467251	0.360344242
B7Z3X3	cDNA FLJ56097, highly similar to Homo sapiens dystrobrevin, alpha (DTNA), transcript variant 2, mRNA OS=Homo sapiens OX=9606 PE=2 SV=1	0.143949966	0.398891092	-0.254941126	0.501475611	0.025039368	0.202946846
A0A5K1VW95	Malate dehydrogenase OS=Homo sapiens OX=9606 GN=MDH1 PE=1 SV=1	-0.164858384	-0.019697473	-0.145160911	0.501557124	0.646018849	0.859501071
Q9NQZ6	Zinc finger C4H2 domain-containing protein OS=Homo sapiens OX=9606 GN=ZC4H2 PE=1 SV=1	-0.200887832	0.281742092	-0.482629924	0.511138629	0.292047267	0.100865552
V9HWE3	Carbonic anhydrase I, isoform CRA_a OS=Homo sapiens OX=9606 GN=HEL-S-11 PE=2 SV=1	-0.308952968	-0.242589878	-0.06636309	0.513348866	0.460138344	0.958264424

P02511	Alpha-crystallin B chain OS=Homo sapiens OX=9606 GN=CRYAB PE=1 SV=2	0.126301486	0.329299784	-0.201998298	0.513901549	0.036955723	0.116664697
H0YG30	Heat shock protein beta-8 (Fragment) OS=Homo sapiens OX=9606 GN=HSPB8 PE=1 SV=1	0.121522587	-0.293824981	0.415347569	0.513981028	0.312390402	0.209044548
D3DSQ1	N-acylsphingosine amidohydrolase (Acid ceramidase) 1, isoform CRA_c OS=Homo sapiens OX=9606 GN=ASAH1 PE=4 SV=1	0.192909665	0.446757653	-0.253847988	0.515537043	0.080904169	0.208770676
P04040	Catalase OS=Homo sapiens OX=9606 GN=CAT PE=1 SV=3	0.183990229	0.161058604	0.022931625	0.521239516	0.630864564	0.872106615
C6EVS4	Calpain 3 transcription variant 11 OS=Homo sapiens OX=9606 GN=CAPN3 PE=2 SV=1	0.178083227	0.672188231	-0.494105003	0.521521818	0.00421718	0.054921129
P05413	Fatty acid-binding protein, heart OS=Homo sapiens OX=9606 GN=FABP3 PE=1 SV=4	-0.207077432	-0.418880867	0.211803434	0.522091097	0.13138877	0.344993302
Q86UH8	Pol protein OS=Homo sapiens OX=9606 PE=4 SV=1	0.177651555	0.145295672	0.032355883	0.522906744	0.46319386	0.937108756
Q9ULC4	Malignant T-cell-amplified sequence 1 OS=Homo sapiens OX=9606 GN=MCTS1 PE=1 SV=1	0.126282475	-0.060332561	0.186615035	0.524666151	0.643655947	0.259926019
B4DU42	cDNA FLJ56153, highly similar to Homo sapiens transforming growth factor beta regulator 4 (TBRG4), transcript variant 1, mRNA OS=Homo sapiens OX=9606 PE=2 SV=1	-0.225271447	0.694404	-0.919675447	0.528087391	0.002273219	0.003420213
B1AKY9	Sodium/potassium-transporting ATPase subunit alpha OS=Homo sapiens OX=9606 GN=ATP1A2 PE=1 SV=1	0.182019219	0.402712061	-0.220692842	0.528605947	0.102752048	0.594800864

B4DEN3	cDNA FLJ61101, highly similar to Eukaryotic translation initiation factor 5 OS=Homo sapiens OX=9606 PE=2 SV=1	0.14710866	0.331906007	-0.184797347	0.532028912	0.061248995	0.344757586
O43719	HIV Tat-specific factor 1 OS=Homo sapiens OX=9606 GN=HTATSF1 PE=1 SV=1	0.105530119	0.50430765	-0.398777531	0.532111973	0.001726188	0.014943853
B4DTS5	cDNA FLJ58882, highly similar to 26S proteasome non-ATPase regulatory subunit 11 OS=Homo sapiens OX=9606 PE=2 SV=1	0.148951546	0.41900374	-0.270052193	0.533466503	0.015494599	0.096869947
Q8WW22	DnaJ homolog subfamily A member 4 OS=Homo sapiens OX=9606 GN=DNAJA4 PE=1 SV=1	-0.146499863	0.34856408	-0.495063943	0.533587548	0.007797316	0.010141301
B5A248	CREBBP (Fragment) OS=Homo sapiens OX=9606 GN=CREBBP PE=4 SV=1	-0.289395052	-0.328139338	0.038744286	0.534876359	0.342927302	0.784395978
A8KAK1	cDNA FLJ77398, highly similar to Homo sapiens UDP-glucose ceramide glucosyltransferase-like 1, transcript variant 2, mRNA OS=Homo sapiens OX=9606 PE=2 SV=1	0.238466733	0.230545358	0.007921375	0.536962981	0.683761482	0.940470928
A0A5F9ZHM4	L-lactate dehydrogenase OS=Homo sapiens OX=9606 GN=LDHB PE=1 SV=1	-0.139996091	0.173374836	-0.313370928	0.537206426	0.173511542	0.143161065
B3KU11	Oxysterol-binding protein OS=Homo sapiens OX=9606 PE=2 SV=1	-0.102882947	0.185220246	-0.288103193	0.537234607	0.032002184	0.03063905
A0A494C0U1	Tripeptidyl-peptidase 2 (Fragment) OS=Homo sapiens OX=9606 GN=TPP2 PE=1 SV=1	0.09390393	0.532345781	-0.438441851	0.540679169	0.004429791	0.005755579
Q13442	28 kDa heat- and acid-stable phosphoprotein OS=Homo sapiens	0.181486509	0.418056	-0.236569491	0.542416088	0.066678044	0.504512903

	OX=9606 GN=PDAP1 PE=1 SV=1						
A0A0S2A4E4	Amylo-1, 6-glucosidase, 4-alpha-glucanotransferase (Glycogen debranching enzyme, glycogen storage disease type III), isoform CRA_b OS=Homo sapiens OX=9606 GN=AGL PE=4 SV=1	-0.164716248	0.326173858	-0.490890107	0.54272622	0.155628333	0.082644781
A2TKE3	Cellular titin isoform PEVK variant 1 (Fragment) OS=Homo sapiens OX=9606 PE=2 SV=1	-0.135222021	0.163905037	-0.299127058	0.54277113	0.279630042	0.11648946
P82933	28S ribosomal protein S9, mitochondrial OS=Homo sapiens OX=9606 GN=MRPS9 PE=1 SV=2	-0.131295062	0.376374533	-0.507669595	0.546179049	0.028126701	0.018839411
O43929	Origin recognition complex subunit 4 OS=Homo sapiens OX=9606 GN=ORC4 PE=1 SV=2	-0.225068536	0.521988119	-0.747056656	0.547829512	0.068096784	0.026599086
P45378	Troponin T, fast skeletal muscle OS=Homo sapiens OX=9606 GN=TNNT3 PE=1 SV=3	-0.449127103	-0.334143568	-0.114983535	0.548047135	0.80342592	0.657513271
B4DL48	cDNA FLJ53382, highly similar to ADAM 23 (A disintegrin and metalloproteinase domain 23) OS=Homo sapiens OX=9606 PE=2 SV=1	-0.220180182	0.001960283	-0.222140465	0.548123112	0.25739422	0.221517267
Q6IRW3	Semenogelin II OS=Homo sapiens OX=9606 GN=SEMG2 PE=2 SV=1	0.164993119	0.398885069	-0.23389195	0.5487033	0.18138106	0.577455794
Q96MG2	Junctional sarcoplasmic reticulum protein 1 OS=Homo sapiens OX=9606 GN=JSRP1 PE=1 SV=1	0.163734781	0.542697528	-0.378962747	0.549137235	0.026805786	0.101293594

A0A024QZJ6	Myosin, heavy polypeptide 11, smooth muscle, isoform CRA_b OS=Homo sapiens OX=9606 GN=MYH11 PE=3 SV=1	0.166942217	0.446864834	-0.279922617	0.5501625	0.045404231	0.17508621
Q05682	Caldesmon OS=Homo sapiens OX=9606 GN=CALD1 PE=1 SV=3	-0.224382636	0.427005917	-0.651388552	0.550731659	0.042993052	0.024986223
Q3SXM5	Inactive hydroxysteroid dehydrogenase-like protein 1 OS=Homo sapiens OX=9606 GN=HSDL1 PE=1 SV=3	-0.124530777	0.60652976	-0.731060537	0.550916722	0.008435083	0.001413713
Q7Z682	Uncharacterized protein DKFZp779I2251 (Fragment) OS=Homo sapiens OX=9606 GN=DKFZp779I2251 PE=2 SV=1	0.210055165	0.538400027	-0.328344863	0.55098162	0.102772732	0.398908933
F8W6I7	Heterogeneous nuclear ribonucleoprotein A1 OS=Homo sapiens OX=9606 GN=HNRNPA1 PE=1 SV=2	0.26098747	0.095883802	0.165103668	0.551097777	0.249254425	0.797166789
V5IRT4	Ubiquinol-cytochrome-c reductase complex assembly factor 2 (Fragment) OS=Homo sapiens OX=9606 GN=UQCC2 PE=1 SV=1	-0.157205175	0.586610064	-0.743815239	0.551519598	0.006917422	0.00138267
M0QZU8	Troponin T, slow skeletal muscle (Fragment) OS=Homo sapiens OX=9606 GN=TNNT1 PE=1 SV=8	-0.149872938	-0.070030099	-0.079842839	0.551609514	0.492469581	0.967067345
Q9Y3P9	Rab GTPase-activating protein 1 OS=Homo sapiens OX=9606 GN=RABGAP1 PE=1 SV=3	0.117634813	0.207380516	-0.089745703	0.552135956	0.043148318	0.342332289
B3KPZ7	cDNA FLJ32517 fis, clone SMINT1000117, highly similar to Pyruvate dehydrogenase (lipoamide)-phosphatase 1	-0.142784815	0.699587788	-0.842372603	0.5532483	0.002432118	0.000211889

	OS=Homo sapiens OX=9606 PE=2 SV=1						
P30711	Glutathione S-transferase theta-1 OS=Homo sapiens OX=9606 GN=GSTT1 PE=1 SV=4	0.209337861	0.233236765	-0.023898903	0.561112122	0.273752166	0.855080665
A8K292	cDNA FLJ78318, highly similar to Homo sapiens sestrin 1 (SESN1), mRNA OS=Homo sapiens OX=9606 PE=2 SV=1	-0.125174891	0.165830133	-0.291005024	0.565732516	0.750059654	0.547835559
A0A384MDT0	Epididymis secretory sperm binding protein OS=Homo sapiens OX=9606 PE=2 SV=1	0.092763702	0.333831505	-0.241067802	0.567067816	0.116618207	0.287472503
Q6F4B5	Adenosine monophosphate deaminase 1 (Fragment) OS=Homo sapiens OX=9606 GN=AMPD1 PE=4 SV=1	0.132855703	0.533546473	-0.40069077	0.567533145	0.007582664	0.066873754
D6RG15	Twinfilin-2 OS=Homo sapiens OX=9606 GN=TWF2 PE=1 SV=1	-0.0877921	0.42137407	-0.50916617	0.567573477	0.007740909	0.005459373
B4DRW6	Alpha-1,4 glucan phosphorylase OS=Homo sapiens OX=9606 PE=2 SV=1	-0.199965699	0.45139829	-0.651363989	0.573058806	0.1780394	0.11690484
A0A384NY64	Epididymis secretory sperm binding protein OS=Homo sapiens OX=9606 PE=2 SV=1	0.164799068	0.358454172	-0.193655104	0.581688182	0.008491214	0.142999784
Q7Z4X0	MO25-like protein OS=Homo sapiens OX=9606 PE=2 SV=1	0.121087954	0.343778464	-0.22269051	0.586601343	0.033739126	0.145742586
P50991	T-complex protein 1 subunit delta OS=Homo sapiens OX=9606 GN=CCT4 PE=1 SV=4	0.122286473	0.119329576	0.002956897	0.587932161	0.503678168	0.897660295
Q9BV19	Uncharacterized protein C1orf50 OS=Homo sapiens OX=9606 GN=C1orf50 PE=1 SV=2	0.091782527	0.075975154	0.015807373	0.588541655	0.357387769	0.720339948
Q96D46	60S ribosomal export protein NMD3 OS=Homo sapiens	0.101289936	0.540883372	-0.439593436	0.589040558	0.003171587	0.009607199

	OX=9606 GN=NMD3 PE=1 SV=1						
B4DP80	NAD(P)H-hydrate epimerase OS=Homo sapiens OX=9606 GN=APOA1BP PE=2 SV=1	0.093961774	0.193291497	-0.099329723	0.589057781	0.082676964	0.345319299
Q6B514	Septin 6 isoform E OS=Homo sapiens OX=9606 PE=2 SV=1	0.146951873	0.265785642	-0.118833769	0.590279515	0.019971257	0.267414825
Q8IZ03	Interferon-induced protein with tetratricopeptide repeats 2 OS=Homo sapiens OX=9606 GN=IFIT2 PE=1 SV=2	0.202634335	0.426188936	-0.223554601	0.591116966	0.148273944	0.587170827
Q32MK0	Myosin light chain kinase 3 OS=Homo sapiens OX=9606 GN=MYLK3 PE=1 SV=3	0.1055091	0.31603034	-0.21052124	0.594684565	0.018853637	0.157166457
B7Z5V0	cDNA FLJ53647, highly similar to Four and a half LIM domains protein 1 OS=Homo sapiens OX=9606 PE=2 SV=1	0.110555873	-0.359609834	0.470165706	0.594724491	0.059896335	0.029953632
Q7Z7D3	V-set domain-containing T-cell activation inhibitor 1 OS=Homo sapiens OX=9606 GN=VTCN1 PE=1 SV=1	0.263768161	0.177597718	0.086170443	0.596280524	0.515491951	0.993957621
P51172	Amiloride-sensitive sodium channel subunit delta OS=Homo sapiens OX=9606 GN=SCNN1D PE=1 SV=3	-0.119748385	-0.707356677	0.587608292	0.596847298	0.003173522	0.012583793
A0A024R8I2	Ubiquitin associated domain containing 1, isoform CRA_c OS=Homo sapiens OX=9606 GN=UBADC1 PE=4 SV=1	0.083947715	0.397167812	-0.313220097	0.599479702	0.00958545	0.064563178
Q99615	DnaJ homolog subfamily C member 7 OS=Homo sapiens OX=9606 GN=DNAJC7 PE=1 SV=2	0.127914039	-0.132523871	-0.26043791	0.599631937	0.203891205	0.110583373

B4E186	cDNA FLJ56834 OS=Homo sapiens OX=9606 PE=2 SV=1	0.357883955	0.844165973	-0.486282018	0.599719061	0.110277143	0.389194103
Q6Y288	Beta-1,3-glucosyltransferase OS=Homo sapiens OX=9606 GN=B3GLCT PE=1 SV=2	-0.122772719	0.134015402	-0.256788121	0.599801147	0.150841746	0.047041644
Q53H40	Endomucin variant (Fragment) OS=Homo sapiens OX=9606 PE=2 SV=1	-0.108534722	0.313796929	-0.422331651	0.603058291	0.01633965	0.021407378
B4DS66	MICOS complex subunit MIC60 OS=Homo sapiens OX=9606 PE=2 SV=1	-0.12141743	0.566600102	-0.688017532	0.609373961	0.003969132	0.00078189
A0A384MTJ7	Epididymis secretory sperm binding protein OS=Homo sapiens OX=9606 PE=2 SV=1	-0.158249457	0.234376645	-0.392626102	0.612319675	0.141774637	0.060610206
Q549N0	Cofilin 2 (Muscle), isoform CRA_a OS=Homo sapiens OX=9606 GN=CFL2 PE=1 SV=1	-0.055729001	-0.084315532	0.028586531	0.612856822	0.485077936	0.865680664
P13535	Myosin-8 OS=Homo sapiens OX=9606 GN=MYH8 PE=1 SV=3	-0.253995271	-0.502019599	0.248024328	0.616606192	0.288490045	0.877958286
B2R7Y3	cDNA, FLJ93657, highly similar to Homo sapiens ribosomal protein L3-like (RPL3L), mRNA OS=Homo sapiens OX=9606 PE=2 SV=1	0.128156342	0.583903723	-0.455747381	0.619166008	0.006624584	0.028727863
Q14324	Myosin-binding protein C, fast-type OS=Homo sapiens OX=9606 GN=MYBPC2 PE=1 SV=2	0.251577217	0.361601825	-0.110024608	0.622780873	0.318151133	0.710518494
A0A3B3IU69	Echinoderm microtubule-associated protein-like 1 OS=Homo sapiens OX=9606 GN=EML1 PE=1 SV=1	0.13100868	0.384526476	-0.253517796	0.626573491	0.04057077	0.281146058
Q9Y4E8	Ubiquitin carboxyl-terminal hydrolase 15 OS=Homo sapiens	0.069448843	0.084296552	-0.014847709	0.627747978	0.999022783	0.780901789

	OX=9606 GN=USP15 PE=1 SV=3						
A6NK59	Ankyrin repeat and SOCS box protein 14 OS=Homo sapiens OX=9606 GN=ASB14 PE=2 SV=2	0.108240267	0.371048604	-0.262808337	0.628168896	0.023803375	0.267686231
A0A087WTG3	Cullin-3 OS=Homo sapiens OX=9606 GN=CUL3 PE=1 SV=1	0.081398026	0.551477904	-0.470079878	0.628346245	0.002232205	0.003431935
Q53H78	Calcium/calmodulin-dependent protein kinase IIB isoform 2 variant (Fragment) OS=Homo sapiens OX=9606 PE=2 SV=1	-0.17146853	0.243226281	-0.414694812	0.6286224	0.18041912	0.01585362
A8K6K7	Glycogen [starch] synthase OS=Homo sapiens OX=9606 PE=2 SV=1	0.108048085	0.698791708	-0.590743623	0.631472136	0.003662514	0.007689925
E9PIE3	Caveolae-associated protein 3 OS=Homo sapiens OX=9606 GN=CAVIN3 PE=1 SV=1	0.201984505	0.921438724	-0.719454219	0.636796508	0.004710457	0.041715689
H0Y3N9	Histone lysine demethylase PHF8 (Fragment) OS=Homo sapiens OX=9606 GN=PHF8 PE=1 SV=1	-0.183811573	-0.41227309	0.228461517	0.637067032	0.247451586	0.757146144
A0A0U1RQX8	E3 ubiquitin-protein ligase CBL OS=Homo sapiens OX=9606 GN=CBL PE=1 SV=1	0.072830046	0.346957604	-0.274127557	0.638005789	0.014080107	0.042954529
P04406	Glyceraldehyde-3-phosphate dehydrogenase OS=Homo sapiens OX=9606 GN=GAPDH PE=1 SV=3	0.142097629	0.361931778	-0.219834149	0.638190159	0.183597505	0.667588285
A0A2R8YCW8	Protein 4.1 OS=Homo sapiens OX=9606 GN=EPB41 PE=1 SV=1	-0.096493035	0.108231703	-0.204724738	0.64182862	0.367301804	0.25653714
H7C2T1	Cyclin-dependent kinase inhibitor 1B (Fragment) OS=Homo sapiens OX=9606 GN=CDKN1B PE=1 SV=1	0.120045766	-0.01290838	0.132954145	0.642146533	0.922568114	0.689858746

B2RB07	Ubiquitin carboxyl-terminal hydrolase OS=Homo sapiens OX=9606 PE=2 SV=1	0.125582291	0.547896375	-0.422314083	0.643222791	0.00560234	0.106827945
A0A0J9YYL3	Poly(U)-binding-splicing factor PUF60 (Fragment) OS=Homo sapiens OX=9606 GN=PUF60 PE=1 SV=1	0.125610051	0.268915261	-0.143305211	0.643839193	0.042037433	0.24730581
E9PLK3	Aminopeptidase OS=Homo sapiens OX=9606 GN=NPEPPS PE=1 SV=1	0.06191221	0.057041786	0.004870424	0.649235709	0.753962129	0.926257099
Q96GX9	Methylthioribulose-1-phosphate dehydratase OS=Homo sapiens OX=9606 GN=APIP PE=1 SV=1	0.142658003	0.450584727	-0.307926724	0.651748215	0.029343319	0.145723852
H7C5J3	Ubiquitin carboxyl-terminal hydrolase 13 (Fragment) OS=Homo sapiens OX=9606 GN=USP13 PE=1 SV=1	-0.10274779	0.079313112	-0.182060902	0.652188585	0.637486298	0.463691781
A0A384MR27	Galectin OS=Homo sapiens OX=9606 PE=2 SV=1	-0.126884404	-0.683722263	0.556837859	0.65589578	0.016644963	0.122700864
Q9UBQ0	Vacuolar protein sorting-associated protein 29 OS=Homo sapiens OX=9606 GN=VPS29 PE=1 SV=1	-0.107299271	0.288411356	-0.395710627	0.655959508	0.014586779	0.024159465
Q6PK32	ANK1 protein OS=Homo sapiens OX=9606 GN=ANK1 PE=1 SV=1	0.122687239	0.843088016	-0.720400777	0.656270305	0.008386277	0.023701608
O00629	Importin subunit alpha-3 OS=Homo sapiens OX=9606 GN=KPNA4 PE=1 SV=1	-0.094104075	0.202388127	-0.296492202	0.659367777	0.011613775	0.030734056
B4DGN3	cDNA FLJ59198, highly similar to Homo sapiens heterogeneous nuclear ribonucleoprotein D-like (HNRPDL), transcript variant 2, mRNA OS=Homo sapiens OX=9606 PE=2 SV=1	0.145122433	0.385050609	-0.239928176	0.659435145	0.017243119	0.171747042

A0A1W2PNP0	GPI transamidase component PIG-T (Fragment) OS=Homo sapiens OX=9606 GN=PIGT PE=1 SV=1	0.088614575	0.495356659	-0.406742084	0.659452786	0.009768212	0.011540397
O43488	Aflatoxin B1 aldehyde reductase member 2 OS=Homo sapiens OX=9606 GN=AKR7A2 PE=1 SV=3	0.070298314	0.392001826	-0.321703511	0.665309142	0.004545664	0.026076234
V9HWB2	Guanidinoacetate N-methyltransferase OS=Homo sapiens OX=9606 GN=HEL-S-20 PE=2 SV=1	0.126256716	-0.067714588	0.193971304	0.666606321	0.850422026	0.721274471
J3KQ18	D-dopachrome decarboxylase OS=Homo sapiens OX=9606 GN=DDT PE=1 SV=1	0.147690134	0.031549551	0.116140583	0.66682206	0.807903394	0.778902363
P48147	Prolyl endopeptidase OS=Homo sapiens OX=9606 GN=PREP PE=1 SV=2	0.066971304	0.004218271	0.062753033	0.67065061	0.900801113	0.607793477
H0YAF2	Leukemia inhibitory factor receptor (Fragment) OS=Homo sapiens OX=9606 GN=LIFR PE=1 SV=1	-0.162427427	0.486321981	-0.648749408	0.671711967	0.00239913	0.012821881
B4DR25	Oxysterol-binding protein OS=Homo sapiens OX=9606 PE=2 SV=1	-0.078335031	0.195206674	-0.273541705	0.67435294	0.106662152	0.107602095
H3BT58	Coactosin-like protein OS=Homo sapiens OX=9606 GN=COTL1 PE=1 SV=1	-0.384468844	-0.696756772	0.312287928	0.674522919	0.304788685	0.63378849
Q9UKX3	Myosin-13 OS=Homo sapiens OX=9606 GN=MYH13 PE=2 SV=2	-0.278413391	-0.550950766	0.272537375	0.675140114	0.251312471	0.73057258
Q9BXI3	Cytosolic 5'-nucleotidase 1A OS=Homo sapiens OX=9606 GN=NT5C1A PE=1 SV=1	-0.16277478	0.364421816	-0.527196597	0.675529167	0.241875968	0.193986235
A0A126GV90	Olfactory receptor OS=Homo sapiens	0.115110782	-0.574209849	0.689320631	0.678532703	0.00752793	0.006330145

	OX=9606 GN=OR8D4 PE=3 SV=1						
O95479	GDH/6PGL endoplasmic bifunctional protein OS=Homo sapiens OX=9606 GN=H6PD PE=1 SV=2	0.097340718	0.387208618	-0.2898679	0.678874434	0.006166167	0.047308186
Q4ZGM8	Hemoglobin alpha-2 globin mutant (Fragment) OS=Homo sapiens OX=9606 PE=3 SV=1	-0.199273363	-0.34417171	0.144898348	0.681955982	0.268885275	0.604061039
P16499	Rod cGMP-specific 3',5'-cyclic phosphodiesterase subunit alpha OS=Homo sapiens OX=9606 GN=PDE6A PE=1 SV=4	-0.161637332	0.376068775	-0.537706107	0.682071328	0.3659272	0.246785432
O75298	Reticulon-2 OS=Homo sapiens OX=9606 GN=RTN2 PE=1 SV=1	-0.092809592	0.231304131	-0.324113723	0.682903362	0.27507105	0.240825886
P61457	Pterin-4-alpha-carbinolamine dehydratase OS=Homo sapiens OX=9606 GN=PCBD1 PE=1 SV=2	0.059088102	0.111900583	-0.052812482	0.687029409	0.117350734	0.434154197
Q00688	Peptidyl-prolyl cis-trans isomerase FKBP3 OS=Homo sapiens OX=9606 GN=FKBP3 PE=1 SV=1	0.068634614	0.312109201	-0.243474587	0.687424375	0.087894979	0.30840433
A0A087X1T8	Isoaspartyl peptidase/L-asparaginase (Fragment) OS=Homo sapiens OX=9606 GN=ASRGL1 PE=4 SV=1	0.098835213	0.468145462	-0.369310249	0.69244514	0.038560897	0.119408248
Q99735	Microsomal glutathione S-transferase 2 OS=Homo sapiens OX=9606 GN=MGST2 PE=1 SV=1	-0.0887801	0.498017881	-0.586797981	0.697850272	0.002794113	0.001803976
A0A0S2Z4B5	Serine/threonine-protein phosphatase (Fragment) OS=Homo sapiens OX=9606 GN=PPP3CA PE=2 SV=1	0.159585374	0.37835286	-0.218767486	0.704198657	0.168751619	0.433197173

Q9NZR1	Tropomodulin-2 OS=Homo sapiens OX=9606 GN=TMOD2 PE=1 SV=1	-0.158298452	-0.004662862	-0.15363559	0.705464641	0.674935222	0.406395899
D3DP78	Aspartyl-tRNA synthetase, isoform CRA_b OS=Homo sapiens OX=9606 GN=DARS PE=3 SV=1	0.080824376	0.524917904	-0.444093528	0.705882536	0.003496123	0.013152061
A8MYV0	Doublecortin domain-containing protein 2C OS=Homo sapiens OX=9606 GN=DCDC2C PE=1 SV=4	0.149662783	-0.132218239	0.281881022	0.706513071	0.684542561	0.520396664
Q5M7Z5	GRHPR protein (Fragment) OS=Homo sapiens OX=9606 GN=GRHPR PE=2 SV=1	-0.058281102	0.1566877	-0.214968802	0.711375907	0.529898204	0.371132944
M0QWZ7	Serine--tRNA ligase, mitochondrial OS=Homo sapiens OX=9606 GN=SARS2 PE=1 SV=1	-0.113227319	0.491083809	-0.604311128	0.711971891	0.02949324	0.017265393
A0A385HW21	Mutant hemoglobin subunit alpha 2 OS=Homo sapiens OX=9606 GN=HBA2 PE=3 SV=1	-0.197356338	0.106561964	-0.303918302	0.712265328	0.947478613	0.813798863
Q13617	Cullin-2 OS=Homo sapiens OX=9606 GN=CUL2 PE=1 SV=2	0.069805044	0.442837858	-0.373032814	0.713853609	0.009153954	0.045215119
Q6IAW5	CALU protein OS=Homo sapiens OX=9606 GN=CALU PE=2 SV=1	0.083037974	0.588544202	-0.505506228	0.713897344	0.006782072	0.00919309
B4DQB3	Signal transducer and activator of transcription OS=Homo sapiens OX=9606 PE=2 SV=1	-0.319155983	0.514228729	-0.833384713	0.714248402	0.146973504	0.109186233
H0Y8C6	Importin-5 (Fragment) OS=Homo sapiens OX=9606 GN=IPO5 PE=1 SV=1	0.067326801	0.342844163	-0.275517363	0.715477851	0.00780933	0.065783135
Q9NTJ4	Alpha-mannosidase 2C1 OS=Homo sapiens OX=9606 GN=MAN2C1 PE=1 SV=1	0.095145687	0.230995006	-0.135849319	0.717678939	0.303388632	0.591870096

B4DYL7	cDNA FLJ58490, highly similar to Homo sapiens CCR4-NOT transcription complex, subunit 1 (CNOT1), transcript variant 1, mRNA OS=Homo sapiens OX=9606 PE=2 SV=1	-0.196055754	-0.660159547	0.464103793	0.719728323	0.108476088	0.487101109
A0A0A0MRN9	Ankyrin repeat domain-containing protein 2 OS=Homo sapiens OX=9606 GN=ANKRD2 PE=1 SV=1	0.1005159	0.026294261	0.074221639	0.723595062	0.684620434	0.951268138
Q9HC74	Uncharacterized protein SBBI23 OS=Homo sapiens OX=9606 GN=SBBI23 PE=2 SV=1	-0.055578393	0.379030459	-0.434608851	0.724711045	0.075130274	0.083239304
Q4G0F5	Vacuolar protein sorting-associated protein 26B OS=Homo sapiens OX=9606 GN=VPS26B PE=1 SV=2	0.108995968	0.498506897	-0.38951093	0.726064729	0.010777192	0.061879277
M0QX76	40S ribosomal protein S16 (Fragment) OS=Homo sapiens OX=9606 GN=RPS16 PE=1 SV=1	0.091750249	0.471260956	-0.379510707	0.726842909	0.007726804	0.046961608
O14958	Calsequestrin-2 OS=Homo sapiens OX=9606 GN=CASQ2 PE=1 SV=2	0.103314817	0.040079617	0.0632352	0.726897789	0.435595512	0.820879572
A0A024QZR3	Protein pelota homolog OS=Homo sapiens OX=9606 GN=hCG_2002731 PE=3 SV=1	0.073472009	0.443807319	-0.37033531	0.729274899	0.018551842	0.070771777
Q5GJ33	Hypothetical rhabdomyosarcoma antigen MU-RMS-40.9A (Fragment) OS=Homo sapiens OX=9606 GN=SMYD1 PE=2 SV=1	0.04841076	0.502244281	-0.453833521	0.737275238	0.013201585	0.034610166
A0A024R2W4	Dystroglycan 1 (Dystrophin-associated glycoprotein 1), isoform CRA_a OS=Homo	0.078077399	0.781495956	-0.703418557	0.737415091	0.002254518	0.000552098

	sapiens OX=9606 GN=DAG1 PE=4 SV=1						
A0A0U1RRK1	Calcium uptake protein 1, mitochondrial OS=Homo sapiens OX=9606 GN=MICU1 PE=1 SV=1	-0.062619525	0.458698737	-0.521318263	0.743577318	0.003674126	0.008776111
Q86TD4	Sarcalumenin OS=Homo sapiens OX=9606 GN=SRL PE=1 SV=2	0.057549292	0.616234982	-0.55868569	0.743954639	0.003803741	0.003438937
A0A087WZJ0	Exophilin-5 (Fragment) OS=Homo sapiens OX=9606 GN=EXPH5 PE=1 SV=1	0.115548337	0.37963904	-0.264090703	0.744241119	0.187739053	0.589781834
P55084	Trifunctional enzyme subunit beta, mitochondrial OS=Homo sapiens OX=9606 GN=HADHB PE=1 SV=3	-0.058435263	0.511974825	-0.570410087	0.74493536	0.003663378	3.80233E-05
Q9BQ69	ADP-ribose glycohydrolase MACROD1 OS=Homo sapiens OX=9606 GN=MACROD1 PE=1 SV=2	0.055140681	0.447036491	-0.39189581	0.746187199	0.018624929	0.066910198
K7EQJ5	40S ribosomal protein S15 OS=Homo sapiens OX=9606 GN=RPS15 PE=1 SV=2	-0.073074257	0.319327252	-0.392401509	0.746234714	0.098200054	0.099454445
A0A1S5UZ39	Hemoglobin subunit alpha OS=Homo sapiens OX=9606 GN=HBA2 PE=2 SV=1	-0.114196861	-0.217158737	0.102961875	0.746251575	0.357428745	0.629903423
A0A1B0GTP8	Voltage-dependent L-type calcium channel subunit alpha-1D OS=Homo sapiens OX=9606 GN=CACNA1D PE=3 SV=1	0.084397113	0.692716618	-0.608319505	0.746811295	0.006580876	0.01502922
A2TDB8	Cysteine and glycine-rich protein 3 (Cardiac LIM protein) OS=Homo sapiens OX=9606 GN=CSRP3 PE=2 SV=1	0.071208643	-0.26123194	0.332440583	0.757546734	0.295570807	0.147995115
P16989	Y-box-binding protein 3 OS=Homo sapiens	0.071572907	0.284070145	-0.212497238	0.759428447	0.136107795	0.308636227

	OX=9606 GN=YBX3 PE=1 SV=4						
V9HW89	Epididymis secretory sperm binding protein Li 95n OS=Homo sapiens OX=9606 GN=HEL-S-95n PE=2 SV=1	0.063960829	0.034521327	0.029439502	0.76223496	0.615701214	0.958296691
A0A1W2PR74	GPI ethanolamine phosphate transferase 1 (Fragment) OS=Homo sapiens OX=9606 GN=PIGN PE=1 SV=1	0.105405521	0.709903225	-0.604497704	0.762287281	0.027958829	0.144331913
B2RE88	cDNA, FLJ96465, highly similar to Homo sapiens solute carrier family 25 (mitochondrial carrier;phosphate carrier), member 3 (SLC25A3), nuclear gene encodingmitochondrial protein, transcript variant... OS=Homo sapiens OX=9606 PE=2 SV=1	-0.045443902	0.531210389	-0.576654291	0.76237046	0.003143522	0.000635681
Q16619	Cardiotrophin-1 OS=Homo sapiens OX=9606 GN=CTF1 PE=1 SV=1	0.06828854	0.332341944	-0.264053404	0.764602559	0.062903434	0.205478328
E7ET17	Peroxisomal multifunctional enzyme type 2 OS=Homo sapiens OX=9606 GN=HSD17B4 PE=1 SV=1	0.073053824	0.686795186	-0.613741361	0.769438354	0.003501589	0.000953088
P15259	Phosphoglycerate mutase 2 OS=Homo sapiens OX=9606 GN=PGAM2 PE=1 SV=3	0.065106213	0.147273691	-0.082167478	0.770120775	0.855409004	0.942644137
F6VCX5	Phosphorylase b kinase gamma catalytic chain, skeletal muscle/heart isoform OS=Homo sapiens OX=9606 GN=PHKG1 PE=1 SV=1	-0.117446767	0.735446088	-0.852892855	0.770354452	0.042954309	0.054370593
I3L4C3	Sperm-associated antigen 7 OS=Homo sapiens OX=9606 GN=SPAG7 PE=1 SV=1	0.066709341	0.537679629	-0.470970287	0.770457557	0.00239226	0.013009843

O75874	Isocitrate dehydrogenase [NADP] cytoplasmic OS=Homo sapiens OX=9606 GN=IDH1 PE=1 SV=2	-0.072883427	0.018802963	-0.091686391	0.770560353	0.318472498	0.369205869
Q9NQE9	Histidine triad nucleotide-binding protein 3 OS=Homo sapiens OX=9606 GN=HINT3 PE=1 SV=1	-0.061765714	0.53643845	-0.598204164	0.770613814	0.0042204	0.002387174
H3BVA8	Lysine--tRNA ligase (Fragment) OS=Homo sapiens OX=9606 GN=KARS1 PE=1 SV=1	0.061766713	0.532735831	-0.470969118	0.770854814	0.004807	0.001757679
A0A140VK07	Testicular secretory protein Li 7 OS=Homo sapiens OX=9606 PE=2 SV=1	-0.070685358	0.220599605	-0.291284963	0.771932631	0.061167184	0.103534362
Q75MQ1	Uncharacterized protein CUL1 (Fragment) OS=Homo sapiens OX=9606 GN=CUL1 PE=3 SV=1	0.046731585	0.253978262	-0.207246677	0.772047937	0.019642287	0.118680282
A0A0A0MRM2	Nebulin-related-anchoring protein OS=Homo sapiens OX=9606 GN=NRAP PE=1 SV=1	-0.07653095	0.608648982	-0.685179932	0.781064909	0.006862764	0.022076905
P36551	Oxygen-dependent coproporphyrinogen-III oxidase, mitochondrial OS=Homo sapiens OX=9606 GN=CPOX PE=1 SV=3	0.070473588	0.550535595	-0.480062007	0.784800934	0.008705762	0.037120472
B4E0A1	cDNA FLJ56277, highly similar to Toll-like receptor 9 OS=Homo sapiens OX=9606 PE=2 SV=1	-0.066214579	0.530926751	-0.59714133	0.789420622	0.005196731	0.00447548
I3L2M9	Phosphatidylcholine transfer protein OS=Homo sapiens OX=9606 GN=PCTP PE=1 SV=1	0.06334393	0.002351806	0.060992124	0.794777403	0.754744683	0.649988458
Q9BVR0	Putative HERC2-like protein 3 OS=Homo	-0.096232657	0.543620694	-0.639853351	0.795177037	0.019930145	0.048677949

	sapiens OX=9606 GN=HERC2P3 PE=5 SV=2						
P14649	Myosin light chain 6B OS=Homo sapiens OX=9606 GN=MYL6B PE=1 SV=1	0.106913541	0.318971271	-0.21205773	0.79569256	0.209173613	0.283656472
A0A0A0MR85	Glutathione S-transferase Mu 4 OS=Homo sapiens OX=9606 GN=GSTM4 PE=1 SV=1	0.195036412	-0.007740885	0.202777297	0.798090581	0.706324896	0.548182246
Q9NXX4	cDNA FLJ20005 fis, clone ADKA02526 OS=Homo sapiens OX=9606 PE=2 SV=1	0.09048138	-0.32970032	0.4201817	0.798541478	0.433407687	0.394747525
P35499	Sodium channel protein type 4 subunit alpha OS=Homo sapiens OX=9606 GN=SCN4A PE=1 SV=4	0.048671672	0.276246676	-0.227575004	0.798796327	0.101726175	0.157804625
Q9Y235	C->U-editing enzyme APOBEC-2 OS=Homo sapiens OX=9606 GN=APOBEC2 PE=1 SV=1	0.09582244	-0.413539024	0.509361464	0.799220993	0.036793916	0.096942951
D6R9Z1	Receptor of-activated protein C kinase 1 (Fragment) OS=Homo sapiens OX=9606 GN=RACK1 PE=1 SV=8	0.0736351	0.428044174	-0.354409075	0.800707574	0.018373189	0.111438784
Q9NZU5	LIM and cysteine-rich domains protein 1 OS=Homo sapiens OX=9606 GN=LMCD1 PE=1 SV=1	0.046037829	-0.051486375	0.097524204	0.802638859	0.452404678	0.434400309
B1AXG1	Non-specific serine/threonine protein kinase OS=Homo sapiens OX=9606 GN=RPS6KA3 PE=1 SV=2	0.051772015	0.490838401	-0.439066386	0.80268331	0.00476288	0.034886672
A8MU46	Smoothelin-like protein 1 OS=Homo sapiens OX=9606 GN=SMTNL1 PE=1 SV=2	0.071642828	0.242121593	-0.170478765	0.8040116	0.357315617	0.57970854
Q13505	Metaxin-1 OS=Homo sapiens OX=9606 GN=MTX1 PE=1 SV=3	-0.065414898	0.66669539	-0.732110289	0.805529466	0.004785523	0.00201842

V9HWC1	Epididymis luminal protein 71 OS=Homo sapiens OX=9606 GN=HEL71 PE=2 SV=1	0.205877889	0.172480849	0.03339704	0.811215858	0.484665215	0.784757495
Q9BRC7	1-phosphatidylinositol 4,5-bisphosphate phosphodiesterase delta-4 OS=Homo sapiens OX=9606 GN=PLCD4 PE=1 SV=1	0.062451117	0.411921424	-0.349470307	0.816162462	0.042084798	0.210751419
Q9NR28	Diablo homolog, mitochondrial OS=Homo sapiens OX=9606 GN=DIABLO PE=1 SV=1	-0.044599357	0.530066178	-0.574665536	0.816189314	0.004437717	0.001311187
Q8IWR8	Ribosomal protein L19 (Fragment) OS=Homo sapiens OX=9606 PE=2 SV=1	0.061501315	0.356977457	-0.295476142	0.816497378	0.033759981	0.087632053
Q9Y508	E3 ubiquitin-protein ligase RNF114 OS=Homo sapiens OX=9606 GN=RNF114 PE=1 SV=1	0.039447247	-0.123359315	0.162806562	0.816515623	0.356303005	0.307927924
Q0QEL2	Citrate synthase (Fragment) OS=Homo sapiens OX=9606 GN=CS PE=2 SV=1	-0.055269439	0.763770857	-0.819040295	0.825631843	0.003177017	0.001805089
Q9H0N5	Pterin-4-alpha-carbinolamine dehydratase 2 OS=Homo sapiens OX=9606 GN=PCBD2 PE=1 SV=4	-0.038146924	0.204101046	-0.242247971	0.826310752	0.082409176	0.10570348
B4DDI0	cDNA FLJ55951, highly similar to Homo sapiens nexilin (F actin binding protein) (NEXN), mRNA OS=Homo sapiens OX=9606 PE=2 SV=1	0.077735043	-0.269481916	0.347216959	0.827184964	0.907555169	0.981852189
Q7Z7M5	Superoxide dismutase (Fragment) OS=Homo sapiens OX=9606 GN=SOD2 PE=2 SV=1	-0.064112266	0.583019397	-0.647131664	0.82890289	0.006026307	0.004819727
V5T7C5	LIM domain binding 3 transcript variant 8 OS=Homo sapiens OX=9606 GN=LDB3 PE=2 SV=1	0.042238909	0.036826882	0.005412027	0.829157447	0.799597761	0.945986213

Q14215	Nebulin (Fragment) OS=Homo sapiens OX=9606 PE=2 SV=1	0.048533591	0.431914582	-0.383380991	0.832372	0.056612	0.142808178
A0A024RC16	CD99 antigen-like 2, isoform CRA_d OS=Homo sapiens OX=9606 GN=CD99L2 PE=2 SV=1	-0.101504789	0.033034311	-0.1345391	0.832671857	0.893272364	0.769749478
H7C579	Omega-amidase NIT2 (Fragment) OS=Homo sapiens OX=9606 GN=NIT2 PE=1 SV=8	0.058370048	0.184848146	-0.126478098	0.834757959	0.13457847	0.421619235
B3KRY5	cDNA FLJ35087 fis, clone PLACE6005546, highly similar to Polymerase I and transcript release factor OS=Homo sapiens OX=9606 PE=2 SV=1	-0.037836219	0.289178312	-0.327014531	0.836977567	0.018180503	0.052041836
G3V2Y4	Serine hydroxymethyltransferase, mitochondrial (Fragment) OS=Homo sapiens OX=9606 GN=SHMT2 PE=1 SV=1	0.039080659	0.616313698	-0.577233039	0.842726995	0.001785799	0.001485327
A8KAH7	cDNA FLJ75444, highly similar to Homo sapiens protein kinase, cAMP-dependent, regulatory, type II, alpha (PRKAR2A), mRNA OS=Homo sapiens OX=9606 PE=2 SV=1	-0.037619613	0.349787381	-0.387406994	0.843203392	0.009807994	0.028776984
Q96S66	Chloride channel CLIC-like protein 1 OS=Homo sapiens OX=9606 GN=CLCC1 PE=1 SV=1	0.040371943	0.431278327	-0.390906384	0.843463584	0.004691015	0.017301737
Q9UKX2	Myosin-2 OS=Homo sapiens OX=9606 GN=MYH2 PE=1 SV=1	-0.139864289	0.090695598	-0.230559887	0.843501617	0.710157874	0.561022514
Q96CV8	Thimet oligopeptidase 1 OS=Homo sapiens OX=9606 GN=THOP1 PE=1 SV=1	0.046961959	0.263246053	-0.216284094	0.843717727	0.018405233	0.090525233
Q5W0H4	Translationally-controlled tumor protein OS=Homo	0.038370856	0.136630167	-0.098259311	0.844008778	0.175260843	0.418640649

	sapiens OX=9606 GN=TPT1 PE=1 SV=1						
V9HWB4	Epididymis secretory sperm binding protein Li 89n OS=Homo sapiens OX=9606 GN=HEL-S-89n PE=2 SV=1	-0.103144758	0.078419377	-0.181564135	0.847047944	0.824735921	0.716808104
B2RA52	cDNA, FLJ94700, highly similar to Homo sapiens interleukin-1 receptor-associated kinase 3 (IRAK3), mRNA OS=Homo sapiens OX=9606 PE=2 SV=1	-0.04659027	-0.510925373	0.464335103	0.849156885	0.063889776	0.185336711
Q8N141	Zinc finger protein 82 homolog OS=Homo sapiens OX=9606 GN=ZFP82 PE=1 SV=1	-0.040298139	-0.296128421	0.255830282	0.853581101	0.218755824	0.353782469
H9KVA9	Lys-63-specific deubiquitinase BRCC36 OS=Homo sapiens OX=9606 GN=BRCC3 PE=1 SV=2	-0.041647517	0.147087376	-0.188734893	0.85359652	0.160767527	0.228977453
F5H5N2	Iron-sulfur cluster assembly enzyme ISCU, mitochondrial OS=Homo sapiens OX=9606 GN=ISCU PE=1 SV=1	-0.033606915	0.280066774	-0.313673689	0.853894659	0.017163529	0.00877574
A8K3D6	cDNA FLJ75512, highly similar to Homo sapiens p53 regulated PA26-T2 nuclear protein (PA26) mRNA OS=Homo sapiens OX=9606 PE=2 SV=1	-0.041926958	0.044872722	-0.086799679	0.854482305	0.628914274	0.579345608
A6NMN0	Phosphorylase b kinase regulatory subunit OS=Homo sapiens OX=9606 GN=PHKA1 PE=1 SV=1	-0.056612388	0.574616132	-0.63122852	0.855814104	0.028483091	0.047014215
O60706	ATP-binding cassette sub-family C member 9 OS=Homo sapiens OX=9606 GN=ABCC9 PE=1 SV=2	0.047764961	0.749578976	-0.701814014	0.857819528	0.003349916	0.004801312

P13533	Myosin-6 OS=Homo sapiens OX=9606 GN=MYH6 PE=1 SV=5	0.105908416	-0.00435694	0.110265355	0.858239847	0.994694139	0.829847047
A0A075B7B1	Desmuslin, isoform CRA_a OS=Homo sapiens OX=9606 GN=SYNM PE=1 SV=1	0.061415168	0.329535558	-0.26812039	0.858280434	0.121028787	0.284327891
Q8TE73	Dynein heavy chain 5, axonemal OS=Homo sapiens OX=9606 GN=DNAH5 PE=1 SV=3	-0.027917039	-0.102457881	0.074540842	0.858623573	0.991680688	0.889868398
H0YLA1	WD repeat-containing protein 61 (Fragment) OS=Homo sapiens OX=9606 GN=WDR61 PE=1 SV=1	0.028343078	0.360719266	-0.332376188	0.858644931	0.00744917	0.01236447
E5RH53	Regulator of microtubule dynamics protein 1 OS=Homo sapiens OX=9606 GN=RMDN1 PE=1 SV=1	0.057194601	0.871043902	-0.813849301	0.859251706	0.002895311	0.001854677
Q05086	Ubiquitin-protein ligase E3A OS=Homo sapiens OX=9606 GN=UBE3A PE=1 SV=4	-0.037063747	0.159286766	-0.196350513	0.860770227	0.36657341	0.163486516
F2Z2Y4	Pyridoxal kinase OS=Homo sapiens OX=9606 GN=PDXK PE=1 SV=1	0.040071095	0.134865228	-0.094794133	0.865554288	0.195409962	0.374675289
C9J5P6	Nitric oxide synthase OS=Homo sapiens OX=9606 GN=NOS1 PE=1 SV=2	-0.041099218	0.622115427	-0.663214645	0.866149107	0.010377669	0.005054721
A0A087WVQ6	Clathrin heavy chain OS=Homo sapiens OX=9606 GN=CLTC PE=1 SV=1	0.040580351	-0.002171235	0.042751586	0.869033474	0.99815682	0.854273523
Q5SRE7	Phytanoyl-CoA dioxygenase domain-containing protein 1 OS=Homo sapiens OX=9606 GN=PHYHD1 PE=1 SV=2	0.085426769	-0.359741429	0.445168198	0.870141907	0.987263194	0.907111139
A0A024R5W6	Tropomyosin 1 (Alpha), isoform CRA_a OS=Homo sapiens	-0.108692245	-0.050778077	-0.057914169	0.872414743	0.724709205	0.65645214

	OX=9606 GN=TPM1 PE=3 SV=1						
A0A1B0GW23	ABHD14A-ACY1 readthrough (Fragment) OS=Homo sapiens OX=9606 GN=ABHD14A-ACY1 PE=4 SV=1	0.029661247	0.247930458	-0.218269211	0.882396923	0.087689046	0.20293709
Q13907	Isopentenyl-diphosphate Delta-isomerase 1 OS=Homo sapiens OX=9606 GN=IDI1 PE=1 SV=2	-0.032944784	0.200910151	-0.233854935	0.882615417	0.135118946	0.135427486
Q6P1N9	Putative deoxyribonuclease TATDN1 OS=Homo sapiens OX=9606 GN=TATDN1 PE=1 SV=2	-0.031131825	0.399217055	-0.43034888	0.883792706	0.004560873	0.004538808
B7Z809	cDNA FLJ56016, highly similar to C-1-tetrahydrofolate synthase, cytoplasmic OS=Homo sapiens OX=9606 PE=2 SV=1	-0.024332429	0.424748008	-0.449080437	0.884810168	0.004400823	0.002646633
B4DPT5	cDNA FLJ50023, highly similar to Alanine aminotransferase 1 OS=Homo sapiens OX=9606 PE=2 SV=1	-0.056747659	0.211049285	-0.267796944	0.884840178	0.481771368	0.44724948
P46939	Utrophin OS=Homo sapiens OX=9606 GN=UTRN PE=1 SV=2	-0.06382252	0.540248106	-0.604070625	0.888284656	0.090421249	0.120614998
Q9ULT8	E3 ubiquitin-protein ligase HECTD1 OS=Homo sapiens OX=9606 GN=HECTD1 PE=1 SV=3	-0.047019619	0.171354183	-0.218373801	0.890737618	0.328511237	0.372375273
Q8IW45	ATP-dependent (S)-NAD(P)H-hydrate dehydratase OS=Homo sapiens OX=9606 GN=NAXD PE=1 SV=1	-0.032257147	0.371906349	-0.404163496	0.891002666	0.012397912	0.020563438
Q2TNI1	Caveolin OS=Homo sapiens OX=9606 GN=CAV1 PE=2 SV=1	0.040046091	0.588967432	-0.548921341	0.892036948	0.01382291	0.043827698
B4DYR7	cDNA FLJ57854, highly similar to Synaptophysin-like protein 2 OS=Homo	0.037942914	0.388057325	-0.350114411	0.892552457	0.121518434	0.182244791

	sapiens OX=9606 PE=2 SV=1						
Q96GX3	KIAA0118 protein (Fragment) OS=Homo sapiens OX=9606 PE=2 SV=1	-0.031663899	0.398906833	-0.430570731	0.892762122	0.01433772	0.006238744
P47755	F-actin-capping protein subunit alpha-2 OS=Homo sapiens OX=9606 GN=CAPZA2 PE=1 SV=3	0.033717522	0.110769896	-0.077052373	0.892777696	0.420848345	0.487459827
Q96A32	Myosin regulatory light chain 2, skeletal muscle isoform OS=Homo sapiens OX=9606 GN=MYLPF PE=2 SV=1	-0.073965082	0.812050086	-0.886015168	0.892926102	0.048656951	0.045632492
B4DN01	cDNA FLJ57332, highly similar to COP9 signalosome complex subunit 3 OS=Homo sapiens OX=9606 PE=2 SV=1	0.014457222	0.417568107	-0.403110885	0.893587383	0.002167998	0.00174742
Q00G26	Perilipin-5 OS=Homo sapiens OX=9606 GN=PLIN5 PE=1 SV=2	-0.092126948	0.737908638	-0.830035586	0.895852809	0.199434742	0.251258675
H6SG13	Cytochrome c oxidase subunit OS=Homo sapiens OX=9606 GN=COX6A2 PE=2 SV=1	-0.026590708	0.288343801	-0.314934509	0.896307774	0.063717802	0.022955518
P02585	Troponin C, skeletal muscle OS=Homo sapiens OX=9606 GN=TNNC2 PE=1 SV=2	0.054518688	0.387203177	-0.33268449	0.896805265	0.179354635	0.22448152
Q7KZA3	Ferrochelatase OS=Homo sapiens OX=9606 GN=DKFZp686P18130 PE=2 SV=1	0.031285068	0.311576952	-0.280291884	0.901731701	0.034156487	0.09414825
H0Y6I0	Golgin subfamily A member 4 (Fragment) OS=Homo sapiens OX=9606 GN=GOLGA4 PE=1 SV=1	-0.055656872	0.490047494	-0.545704365	0.906759836	0.113813347	0.203211525
P12883	Myosin-7 OS=Homo sapiens OX=9606 GN=MYH7 PE=1 SV=5	-0.054277655	-0.531677421	0.477399766	0.910033496	0.095642606	0.336579514

V9HW62	Lactoylglutathione lyase OS=Homo sapiens OX=9606 GN=HEL-S-74 PE=2 SV=1	-0.050179424	-0.266640035	0.216460611	0.912581121	0.289811096	0.535412824
D6RFN0	COP9 signalosome complex subunit 4 OS=Homo sapiens OX=9606 GN=COPS4 PE=1 SV=1	0.023270701	0.259299438	-0.236028737	0.916264958	0.04204564	0.109344354
P11055	Myosin-3 OS=Homo sapiens OX=9606 GN=MYH3 PE=1 SV=3	-0.053146099	-0.136759195	0.083613096	0.918949158	0.79267091	0.687948432
H0YDD2	UPF0577 protein KIAA1324 (Fragment) OS=Homo sapiens OX=9606 GN=KIAA1324 PE=1 SV=1	-0.037968499	-0.18153016	0.143561661	0.922563718	0.827419385	0.940596424
P11586	C-1-tetrahydrofolate synthase, cytoplasmic OS=Homo sapiens OX=9606 GN=MTHFD1 PE=1 SV=3	-0.023693445	0.460637782	-0.484331227	0.923370427	0.006545098	0.002955209
P30043	Flavin reductase (NADPH) OS=Homo sapiens OX=9606 GN=BLVRB PE=1 SV=3	0.025095009	0.057967995	-0.032872986	0.923491026	0.948547746	0.888511478
F1BXA6	Beta-actin (Fragment) OS=Homo sapiens OX=9606 PE=2 SV=1	0.035224442	-0.711204443	0.746428885	0.932116189	0.017033639	0.064756135
P26641	Elongation factor 1-gamma OS=Homo sapiens OX=9606 GN=EEF1G PE=1 SV=3	-0.021462901	0.60880603	-0.630268931	0.932867621	0.013612263	0.032274564
A0A2R8Y7B1	Girdin OS=Homo sapiens OX=9606 GN=CCDC88A PE=1 SV=1	-0.044800989	-0.343863983	0.299062994	0.939114227	0.524201116	0.780747727
Q9BX66	Sorbin and SH3 domain-containing protein 1 OS=Homo sapiens OX=9606 GN=SORBS1 PE=1 SV=3	0.02305407	0.883775276	-0.860721206	0.939143278	0.00172964	0.001198305
B1AK20	DnaJ homolog subfamily C member 11 (Fragment) OS=Homo sapiens OX=9606 GN=DNAJC11 PE=1 SV=1	0.023384152	0.468002107	-0.444617954	0.939150082	0.003993758	0.028820425

Q9Y6I3	Epsin-1 OS=Homo sapiens OX=9606 GN=EPN1 PE=1 SV=2	0.016140531	0.31788389	-0.301743359	0.939422628	0.041954673	0.110898514
Q59GD3	Calcium channel, voltage-dependent, beta 1 subunit isoform 1 variant (Fragment) OS=Homo sapiens OX=9606 PE=2 SV=1	-0.015226985	0.350469388	-0.365696373	0.939514673	0.022442779	0.037303718
Q9UBF9	Myotilin OS=Homo sapiens OX=9606 GN=MYOT PE=1 SV=2	-0.015906103	0.3577679	-0.373674003	0.945171121	0.023033129	0.181147323
Q93100	Phosphorylase b kinase regulatory subunit beta OS=Homo sapiens OX=9606 GN=PHKB PE=1 SV=3	-0.031176209	0.869741249	-0.900917459	0.945641463	0.014692073	0.038273691
D6W5C2	Reticulon OS=Homo sapiens OX=9606 GN=RTN4 PE=4 SV=1	-0.010132488	0.37941633	-0.389548817	0.946316636	0.00401593	0.00074078
B4YAH7	ALDH2 (Fragment) OS=Homo sapiens OX=9606 GN=ALDH2 PE=3 SV=1	-0.026229337	0.143994461	-0.170223798	0.946695197	0.227061543	0.328126938
Q8N3K9	Cardiomyopathy-associated protein 5 OS=Homo sapiens OX=9606 GN=CMYA5 PE=1 SV=3	0.011457161	0.378662625	-0.367205465	0.947202214	0.027670505	0.043743492
Q8N7G1	Purine nucleoside phosphorylase OS=Homo sapiens OX=9606 PE=2 SV=1	-0.023425756	0.010439126	-0.033864882	0.951455669	0.987363401	0.945270753
P16157	Ankyrin-1 OS=Homo sapiens OX=9606 GN=ANK1 PE=1 SV=3	0.016882087	0.224673724	-0.207791636	0.951855266	0.311253544	0.46461461
I3L4J1	Uncharacterized protein (Fragment) OS=Homo sapiens OX=9606 PE=1 SV=3	-0.009548694	0.257512462	-0.267061156	0.95717888	0.007442998	0.019818562
E9PNP3	Mth938 domain-containing protein OS=Homo sapiens OX=9606 GN=AAMDC PE=1 SV=1	-0.013458343	-0.140804848	0.127346505	0.95870751	0.151734113	0.261834847

P48788	Troponin I, fast skeletal muscle OS=Homo sapiens OX=9606 GN=TNNI2 PE=1 SV=2	-0.044523815	-0.091465653	0.046941838	0.960388521	0.920451793	0.886191636
Q96MI9	Cytosolic carboxypeptidase 4 OS=Homo sapiens OX=9606 GN=AGBL1 PE=1 SV=3	0.015760882	0.330877663	-0.315116781	0.961685189	0.086999	0.17497587
H7C0G1	Transmembrane protein 245 (Fragment) OS=Homo sapiens OX=9606 GN=TMEM245 PE=1 SV=1	-0.012453255	0.582648196	-0.595101451	0.966617382	0.003657571	0.008758086
A0A2R8YE50	cGMP-dependent protein kinase 1 OS=Homo sapiens OX=9606 GN=PRKG1 PE=1 SV=1	-0.013849527	0.497498767	-0.511348294	0.968131141	0.014614727	0.053520906
Q02790	Peptidyl-prolyl cis-trans isomerase FKBP4 OS=Homo sapiens OX=9606 GN=FKBP4 PE=1 SV=3	0.009754833	0.191396869	-0.181642036	0.970675777	0.06067737	0.193590688
D6RAW2	ADP-ribose pyrophosphatase, mitochondrial (Fragment) OS=Homo sapiens OX=9606 GN=NUDT9 PE=1 SV=1	-0.007387278	0.292277822	-0.2996651	0.974201509	0.019451475	0.065935517
P49257	Protein ERGIC-53 OS=Homo sapiens OX=9606 GN=LMAN1 PE=1 SV=2	0.007093997	0.625644054	-0.618550057	0.977586912	0.003177601	0.001904906
B4YUQ1	Prostate-specific transglutaminase 4 OS=Homo sapiens OX=9606 GN=TGM4 PE=4 SV=1	-0.01045841	0.35838613	-0.36884454	0.977715715	0.177991714	0.201311142
B4DWN2	cDNA FLJ56164, highly similar to Homo sapiens ring finger protein 10 (RNF10), mRNA OS=Homo sapiens OX=9606 PE=2 SV=1	0.026295065	-0.064297425	0.09059249	0.978015626	0.620599679	0.731583392

R4GMQ8	Protein phosphatase 1 regulatory subunit 1A OS=Homo sapiens OX=9606 GN=PPP1R1A PE=1 SV=1	0.006996738	0.672470355	-0.665473617	0.978359215	0.005988391	0.011586333
F5H5T6	Uncharacterized protein (Fragment) OS=Homo sapiens OX=9606 PE=3 SV=1	-0.0084263	0.492646026	-0.501072326	0.978435161	0.035799711	0.086155803
B7Z3Y2	cDNA FLJ51879, highly similar to Prenylcysteine oxidase OS=Homo sapiens OX=9606 PE=2 SV=1	0.005997058	0.406921573	-0.400924516	0.978729735	0.01886552	0.019460141
P30044	Peroxiredoxin-5, mitochondrial OS=Homo sapiens OX=9606 GN=PRDX5 PE=1 SV=4	-0.005310474	0.306043275	-0.311353748	0.978934302	0.075087261	0.094858827
B3KY78	cDNA FLJ46304 fis, clone TESTI4037949, highly similar to Kelch-like protein 8 OS=Homo sapiens OX=9606 PE=2 SV=1	0.013958366	0.307307358	-0.293348991	0.981289249	0.244149071	0.344989362
A0A0S2Z434	Hydroxysteroid dehydrogenase 10 isoform 2 OS=Homo sapiens OX=9606 GN=HSD17B10 PE=2 SV=1	0.004460606	0.843665165	-0.839204559	0.984536507	0.002241355	0.000888137
Q16821	Protein phosphatase 1 regulatory subunit 3A OS=Homo sapiens OX=9606 GN=PPP1R3A PE=1 SV=3	0.005734288	0.572199009	-0.566464721	0.984914843	0.037857092	0.090547844
P49755	Transmembrane emp24 domain-containing protein 10 OS=Homo sapiens OX=9606 GN=TMED10 PE=1 SV=2	-0.004270856	0.472022377	-0.476293233	0.985261494	0.071329867	0.080594667
C7DUW4	Mitogen activated protein kinase kinase 3 OS=Homo sapiens OX=9606 GN=MAP2K3 PE=2 SV=1	0.004925979	0.067872498	-0.062946519	0.986034598	0.910427933	0.941880017

P05976	Myosin light chain 1/3, skeletal muscle isoform OS=Homo sapiens OX=9606 GN=MYL1 PE=1 SV=3	-0.010673741	0.438404945	-0.449078687	0.986125172	0.161830236	0.201369095
J3QT56	Huntingtin-interacting protein K OS=Homo sapiens OX=9606 GN=HYPK PE=1 SV=1	-0.00441808	0.26680015	-0.27121823	0.986139277	0.111142619	0.157457088
P51570	Galactokinase OS=Homo sapiens OX=9606 GN=GALK1 PE=1 SV=1	-0.003249814	0.25443653	-0.257686345	0.987917826	0.086568282	0.208957225
B4DP06	cDNA FLJ57133, highly similar to Bifunctional purine biosynthesis protein PURH OS=Homo sapiens OX=9606 PE=2 SV=1	0.002094162	0.10085274	-0.098758578	0.988248554	0.323563696	0.446484474
V9HWN7	Fructose-bisphosphate aldolase OS=Homo sapiens OX=9606 GN=HEL-S-87p PE=2 SV=1	-0.004123373	-0.024078866	0.019955493	0.988619249	0.663914802	0.791349202
O43169	Cytochrome b5 type B OS=Homo sapiens OX=9606 GN=CYB5B PE=1 SV=3	0.00159686	0.480221136	-0.478624276	0.990227075	0.003881456	0.001917605
Q16531	DNA damage-binding protein 1 OS=Homo sapiens OX=9606 GN=DDB1 PE=1 SV=1	0.001225421	0.304723268	-0.303497847	0.994011559	0.005329392	0.071531377

East Carolina University Proteomics Results

Accession	Description	Log2FC (CLTI/Control)	P(adjusted) CLTI vs Control
P68871	Hemoglobin subunit beta OS=Homo sapiens OX=9606 GN=HBB PE=1 SV=2	-8.770097708	5.06828E-07
P69905	Hemoglobin subunit alpha OS=Homo sapiens OX=9606 GN=HBA1 PE=1 SV=2	-7.095729532	1.08072E-06
Q53GG5-2	Isoform 2 of PDZ and LIM domain protein 3 OS=Homo sapiens OX=9606 GN=PDLIM3	2.804469214	1.95986E-06
P35625	Metalloproteinase inhibitor 3 OS=Homo sapiens OX=9606 GN=TIMP3 PE=1 SV=2	4.799090463	2.85741E-06
P14927	Cytochrome b-c1 complex subunit 7 OS=Homo sapiens OX=9606 GN=UQCRB PE=1 SV=2	-2.191392655	3.95168E-06

P24310	Cytochrome c oxidase subunit 7A1, mitochondrial OS=Homo sapiens OX=9606 GN=COX7A1 PE=1 SV=2	-3.117005044	7.16549E-06
P23284	Peptidyl-prolyl cis-trans isomerase B OS=Homo sapiens OX=9606 GN=PPIB PE=1 SV=2	4.379962351	1.11302E-05
P24752	Acetyl-CoA acetyltransferase, mitochondrial OS=Homo sapiens OX=9606 GN=ACAT1 PE=1 SV=1	-3.57670824	1.64005E-05
P12235	ADP/ATP translocase 1 OS=Homo sapiens OX=9606 GN=SLC25A4 PE=1 SV=4	-5.431865602	1.71989E-05
P30040	Endoplasmic reticulum resident protein 29 OS=Homo sapiens OX=9606 GN=ERP29 PE=1 SV=4	4.982358893	1.93729E-05
P02042	Hemoglobin subunit delta OS=Homo sapiens OX=9606 GN=HBD PE=1 SV=2	-5.379743724	2.25165E-05
P31930	Cytochrome b-c1 complex subunit 1, mitochondrial OS=Homo sapiens OX=9606 GN=UQCRC1 PE=1 SV=3	-4.683892218	2.29339E-05
Q9UKY7	Protein CDV3 homolog OS=Homo sapiens OX=9606 GN=CDV3 PE=1 SV=1	4.248287062	2.49666E-05
P13073	Cytochrome c oxidase subunit 4 isoform 1, mitochondrial OS=Homo sapiens OX=9606 GN=COX4I1 PE=1 SV=1	-4.053968587	2.63713E-05
Q99735	Microsomal glutathione S-transferase 2 OS=Homo sapiens OX=9606 GN=MGST2 PE=1 SV=1	2.280543579	2.63885E-05
Q08623-4	Isoform 4 of Pseudouridine-5'-phosphatase OS=Homo sapiens OX=9606 GN=PUDP	4.011279802	2.70628E-05
Q96C23	Aldose 1-epimerase OS=Homo sapiens OX=9606 GN=GALM PE=1 SV=1	4.228670746	2.80526E-05
P68400	Casein kinase II subunit alpha OS=Homo sapiens OX=9606 GN=CSNK2A1 PE=1 SV=1	5.577627408	2.85172E-05
P23142	Fibulin-1 OS=Homo sapiens OX=9606 GN=FBLN1 PE=1 SV=4	5.826970121	3.09588E-05
Q96QK1	Vacuolar protein sorting-associated protein 35 OS=Homo sapiens OX=9606 GN=VPS35 PE=1 SV=2	2.3067394	3.59795E-05
Q9P2R7	Succinate--CoA ligase [ADP-forming] subunit beta, mitochondrial OS=Homo sapiens OX=9606 GN=SUCLA2 PE=1 SV=3	-5.889027699	3.74839E-05
P13929	Beta-enolase OS=Homo sapiens OX=9606 GN=ENO3 PE=1 SV=5	-6.573221262	3.75112E-05
Q9Y6C2	EMILIN-1 OS=Homo sapiens OX=9606 GN=EMILIN1 PE=1 SV=3	3.422469247	4.0183E-05
Q9UKX2	Myosin-2 OS=Homo sapiens OX=9606 GN=MYH2 PE=1 SV=1	-5.771274028	4.07706E-05
P21397	Amine oxidase [flavin-containing] A OS=Homo sapiens OX=9606 GN=MAOA PE=1 SV=1	7.389675137	4.10513E-05
P05156	Complement factor I OS=Homo sapiens OX=9606 GN=CFI PE=1 SV=2	4.66900719	4.18336E-05

O75323	Protein NipSnap homolog 2 OS=Homo sapiens OX=9606 GN=NIPSNAP2 PE=1 SV=1	-2.449951617	4.56514E-05
O43181	NADH dehydrogenase [ubiquinone] iron-sulfur protein 4, mitochondrial OS=Homo sapiens OX=9606 GN=NDUFS4 PE=1 SV=1	-2.073672008	4.80516E-05
Q5JRX3-2	Isoform 2 of Presequence protease, mitochondrial OS=Homo sapiens OX=9606 GN=PITRM1	5.72805911	4.82548E-05
P15259	Phosphoglycerate mutase 2 OS=Homo sapiens OX=9606 GN=PGAM2 PE=1 SV=3	-5.711493255	4.87892E-05
Q05639	Elongation factor 1-alpha 2 OS=Homo sapiens OX=9606 GN=EEF1A2 PE=1 SV=1	-6.145726977	4.96745E-05
P62310	U6 snRNA-associated Sm-like protein LSm3 OS=Homo sapiens OX=9606 GN=LSM3 PE=1 SV=2	4.451779192	5.16803E-05
Q96HN2	Adenosylhomocysteinase 3 OS=Homo sapiens OX=9606 GN=AHCYL2 PE=1 SV=1	2.490989635	5.28334E-05
P11279	Lysosome-associated membrane glycoprotein 1 OS=Homo sapiens OX=9606 GN=LAMP1 PE=1 SV=3	6.53563199	5.33252E-05
P14555	Phospholipase A2, membrane associated OS=Homo sapiens OX=9606 GN=PLA2G2A PE=1 SV=2	5.951691484	5.35256E-05
P24821	Tenascin OS=Homo sapiens OX=9606 GN=TNC PE=1 SV=3	4.758693968	5.70963E-05
Q9NP74	Palmdelphin OS=Homo sapiens OX=9606 GN=PALMD PE=1 SV=1	3.716329927	5.77248E-05
O60493	Sorting nexin-3 OS=Homo sapiens OX=9606 GN=SNX3 PE=1 SV=3	4.814027111	5.94523E-05
Q13243	Serine/arginine-rich splicing factor 5 OS=Homo sapiens OX=9606 GN=SRSF5 PE=1 SV=1	7.475255597	6.03941E-05
P06732	Creatine kinase M-type OS=Homo sapiens OX=9606 GN=CKM PE=1 SV=2	-8.151197034	6.04665E-05
P78527	DNA-dependent protein kinase catalytic subunit OS=Homo sapiens OX=9606 GN=PRKDC PE=1 SV=3	4.192713987	6.13987E-05
P05090	Apolipoprotein D OS=Homo sapiens OX=9606 GN=APOD PE=1 SV=1	2.521588313	6.44473E-05
Q16891-4	Isoform 4 of MICOS complex subunit MIC60 OS=Homo sapiens OX=9606 GN=IMMT	7.210488572	6.53387E-05
P00918	Carbonic anhydrase 2 OS=Homo sapiens OX=9606 GN=CA2 PE=1 SV=2	-6.349249531	6.71705E-05
P80748	Immunoglobulin lambda variable 3-21 OS=Homo sapiens OX=9606 GN=IGLV3-21 PE=1 SV=2	7.706264579	6.88834E-05
Q9NZ08-2	Isoform 2 of Endoplasmic reticulum aminopeptidase 1 OS=Homo sapiens OX=9606 GN=ERAP1	7.823736537	6.91528E-05
P17174	Aspartate aminotransferase, cytoplasmic OS=Homo sapiens OX=9606 GN=GOT1 PE=1 SV=3	-7.719096684	6.95986E-05

P48735	Isocitrate dehydrogenase [NADP], mitochondrial OS=Homo sapiens OX=9606 GN=IDH2 PE=1 SV=2	-8.321819391	6.98984E-05
P51649-2	Isoform 2 of Succinate-semialdehyde dehydrogenase, mitochondrial OS=Homo sapiens OX=9606 GN=ALDH5A1	-3.665131383	7.04888E-05
P68104	Elongation factor 1-alpha 1 OS=Homo sapiens OX=9606 GN=EEF1A1 PE=1 SV=1	4.224702854	7.37221E-05
Q8TCS8	Polyribonucleotide nucleotidyltransferase 1, mitochondrial OS=Homo sapiens OX=9606 GN=PNPT1 PE=1 SV=2	4.676527011	7.39926E-05
O60662	Kelch-like protein 41 OS=Homo sapiens OX=9606 GN=KLHL41 PE=1 SV=2	-8.136986259	7.49103E-05
P60174	Triosephosphate isomerase OS=Homo sapiens OX=9606 GN=TPI1 PE=1 SV=3	-6.037853492	7.54412E-05
P40926	Malate dehydrogenase, mitochondrial OS=Homo sapiens OX=9606 GN=MDH2 PE=1 SV=3	-3.748555279	7.68458E-05
Q12805	EGF-containing fibulin-like extracellular matrix protein 1 OS=Homo sapiens OX=9606 GN=EFEMP1 PE=1 SV=2	6.277683858	7.9749E-05
P30405	Peptidyl-prolyl cis-trans isomerase F, mitochondrial OS=Homo sapiens OX=9606 GN=PPIF PE=1 SV=1	-3.406967011	8.11441E-05
P11217	Glycogen phosphorylase, muscle form OS=Homo sapiens OX=9606 GN=PYGM PE=1 SV=6	-6.343839674	8.19176E-05
P38919	Eukaryotic initiation factor 4A-III OS=Homo sapiens OX=9606 GN=EIF4A3 PE=1 SV=4	3.64211745	8.63543E-05
Q86V88	Magnesium-dependent phosphatase 1 OS=Homo sapiens OX=9606 GN=MDP1 PE=1 SV=1	5.886461401	8.66899E-05
O60240	Perilipin-1 OS=Homo sapiens OX=9606 GN=PLIN1 PE=1 SV=2	2.123183356	8.98173E-05
O94901-9	Isoform 9 of SUN domain-containing protein 1 OS=Homo sapiens OX=9606 GN=SUN1	2.176468948	9.13004E-05
P02741	C-reactive protein OS=Homo sapiens OX=9606 GN=CRP PE=1 SV=1	7.985070062	9.14672E-05
P02792	Ferritin light chain OS=Homo sapiens OX=9606 GN=FTL PE=1 SV=2	4.198977194	9.30668E-05
Q15008	26S proteasome non-ATPase regulatory subunit 6 OS=Homo sapiens OX=9606 GN=PSMD6 PE=1 SV=1	2.172544516	9.3539E-05
P04406	Glyceraldehyde-3-phosphate dehydrogenase OS=Homo sapiens OX=9606 GN=GAPDH PE=1 SV=3	-5.259192696	9.57459E-05
P05413	Fatty acid-binding protein, heart OS=Homo sapiens OX=9606 GN=FABP3 PE=1 SV=4	-5.939624069	9.67616E-05
O75891-3	Isoform 3 of Cytosolic 10-formyltetrahydrofolate dehydrogenase OS=Homo sapiens OX=9606 GN=ALDH1L1	3.208614773	9.69852E-05
P06576	ATP synthase subunit beta, mitochondrial OS=Homo sapiens OX=9606 GN=ATP5F1B PE=1 SV=3	-8.160177238	9.76986E-05

Q06124-1	Isoform 2 of Tyrosine-protein phosphatase non-receptor type 11 OS=Homo sapiens OX=9606 GN=PTPN11	6.904448615	0.000100181
P26373	60S ribosomal protein L13 OS=Homo sapiens OX=9606 GN=RPL13 PE=1 SV=4	2.207730351	0.000102071
P02585	Troponin C, skeletal muscle OS=Homo sapiens OX=9606 GN=TNNC2 PE=1 SV=2	-7.520320436	0.000105155
O00423-3	Isoform 3 of Echinoderm microtubule-associated protein-like 1 OS=Homo sapiens OX=9606 GN=EML1	4.673211973	0.000106635
O75746	Calcium-binding mitochondrial carrier protein Aralar1 OS=Homo sapiens OX=9606 GN=SLC25A12 PE=1 SV=2	-3.805882043	0.000109397
Q49AH0	Cerebral dopamine neurotrophic factor OS=Homo sapiens OX=9606 GN=CDNF PE=1 SV=2	4.255037709	0.000109465
P61604	10 kDa heat shock protein, mitochondrial OS=Homo sapiens OX=9606 GN=HSPE1 PE=1 SV=2	-4.854108365	0.00011485
O00483	Cytochrome c oxidase subunit NDUFA4 OS=Homo sapiens OX=9606 GN=NDUFA4 PE=1 SV=1	-3.694037519	0.000116371
P15374	Ubiquitin carboxyl-terminal hydrolase isozyme L3 OS=Homo sapiens OX=9606 GN=UCHL3 PE=1 SV=1	3.289799106	0.000117848
P00915	Carbonic anhydrase 1 OS=Homo sapiens OX=9606 GN=CA1 PE=1 SV=2	-4.713469699	0.000118972
P12956	X-ray repair cross-complementing protein 6 OS=Homo sapiens OX=9606 GN=XRCC6 PE=1 SV=2	4.58620348	0.000120954
P06753	Tropomyosin alpha-3 chain OS=Homo sapiens OX=9606 GN=TPM3 PE=1 SV=2	-7.227105008	0.000124901
Q99497	Protein/nucleic acid deglycase DJ-1 OS=Homo sapiens OX=9606 GN=PARK7 PE=1 SV=2	-3.475182186	0.000131833
O00560	Syntenin-1 OS=Homo sapiens OX=9606 GN=SDCBP PE=1 SV=1	8.24325253	0.000134001
P11413-2	Isoform Long of Glucose-6-phosphate 1-dehydrogenase OS=Homo sapiens OX=9606 GN=G6PD	3.6353406	0.000134101
P21796	Voltage-dependent anion-selective channel protein 1 OS=Homo sapiens OX=9606 GN=VDAC1 PE=1 SV=2	-5.837302958	0.000138158
P22695	Cytochrome b-c1 complex subunit 2, mitochondrial OS=Homo sapiens OX=9606 GN=UQCRC2 PE=1 SV=3	-6.403884225	0.000138397
P15954	Cytochrome c oxidase subunit 7C, mitochondrial OS=Homo sapiens OX=9606 GN=COX7C PE=1 SV=1	-4.898672888	0.000138812
Q13867	Bleomycin hydrolase OS=Homo sapiens OX=9606 GN=BLMH PE=1 SV=1	3.289018184	0.000139334
Q99627	COP9 signalosome complex subunit 8 OS=Homo sapiens OX=9606 GN=COPS8 PE=1 SV=1	4.539793097	0.000141357

P36957	Dihydrolipoyllysine-residue succinyltransferase component of 2-oxoglutarate dehydrogenase complex, mitochondrial OS=Homo sapiens OX=9606 GN=DLST PE=1 SV=4	-3.030252475	0.000141478
P63151-2	Isoform 2 of Serine/threonine-protein phosphatase 2A 55 kDa regulatory subunit B alpha isoform OS=Homo sapiens OX=9606 GN=PPP2R2A	7.091327965	0.000141512
P35573	Glycogen debranching enzyme OS=Homo sapiens OX=9606 GN=AGL PE=1 SV=3	-4.077870092	0.000144407
P04075-2	Isoform 2 of Fructose-bisphosphate aldolase A OS=Homo sapiens OX=9606 GN=ALDOA	-4.862871008	0.000145149
Q6JBY9	CapZ-interacting protein OS=Homo sapiens OX=9606 GN=RCSD1 PE=1 SV=1	8.017734764	0.000147044
O75390	Citrate synthase, mitochondrial OS=Homo sapiens OX=9606 GN=CS PE=1 SV=2	-4.400013686	0.000147128
P62937	Peptidyl-prolyl cis-trans isomerase A OS=Homo sapiens OX=9606 GN=PPIA PE=1 SV=2	2.924508114	0.000147156
P62314	Small nuclear ribonucleoprotein Sm D1 OS=Homo sapiens OX=9606 GN=SNRPD1 PE=1 SV=1	5.44182102	0.000148759
Q15056	Eukaryotic translation initiation factor 4H OS=Homo sapiens OX=9606 GN=EIF4H PE=1 SV=5	5.640655793	0.00014982
P10916	Myosin regulatory light chain 2, ventricular/cardiac muscle isoform OS=Homo sapiens OX=9606 GN=MYL2 PE=1 SV=3	-6.156796123	0.000157431
P49257	Protein ERGIC-53 OS=Homo sapiens OX=9606 GN=LMAN1 PE=1 SV=2	2.091642557	0.000158146
Q9Y2Q3	Glutathione S-transferase kappa 1 OS=Homo sapiens OX=9606 GN=GSTK1 PE=1 SV=3	-2.333501479	0.000164447
P25398	40S ribosomal protein S12 OS=Homo sapiens OX=9606 GN=RPS12 PE=1 SV=3	1.907948346	0.000165615
P99999	Cytochrome c OS=Homo sapiens OX=9606 GN=CYCS PE=1 SV=2	-5.448100805	0.000170646
P12883	Myosin-7 OS=Homo sapiens OX=9606 GN=MYH7 PE=1 SV=5	-7.296904822	0.000171479
P16401	Histone H1.5 OS=Homo sapiens OX=9606 GN=H1-5 PE=1 SV=3	5.637687269	0.000172608
Q86X76-3	Isoform 4 of Deaminated glutathione amidase OS=Homo sapiens OX=9606 GN=NIT1	2.792858415	0.000181738
Q13642-5	Isoform 5 of Four and a half LIM domains protein 1 OS=Homo sapiens OX=9606 GN=FHL1	-4.217095923	0.000183446
Q9Y6E2	Basic leucine zipper and W2 domain-containing protein 2 OS=Homo sapiens OX=9606 GN=BZW2 PE=1 SV=1	3.216894017	0.000185484
P30041	Peroxiredoxin-6 OS=Homo sapiens OX=9606 GN=PRDX6 PE=1 SV=3	-4.982196424	0.000185513
O00757	Fructose-1,6-bisphosphatase isozyme 2 OS=Homo sapiens OX=9606 GN=FBP2 PE=1 SV=2	-3.782732444	0.000188256

P02748	Complement component C9 OS=Homo sapiens OX=9606 GN=C9 PE=1 SV=2	4.237006801	0.000189638
P45378	Troponin T, fast skeletal muscle OS=Homo sapiens OX=9606 GN=TNNT3 PE=1 SV=3	-4.43721112	0.000194869
P21399	Cytoplasmic aconitate hydratase OS=Homo sapiens OX=9606 GN=ACO1 PE=1 SV=3	-4.110715108	0.000197251
P02768	Serum albumin OS=Homo sapiens OX=9606 GN=ALB PE=1 SV=2	-4.450142827	0.000197422
O00571	ATP-dependent RNA helicase DDX3X OS=Homo sapiens OX=9606 GN=DDX3X PE=1 SV=3	4.599004189	0.00020065
Q96EY1	DnaJ homolog subfamily A member 3, mitochondrial OS=Homo sapiens OX=9606 GN=DNAJA3 PE=1 SV=2	6.369906211	0.00020265
Q9UN36	Protein NDRG2 OS=Homo sapiens OX=9606 GN=NDRG2 PE=1 SV=2	-5.276196624	0.000204872
Q9UBY9	Heat shock protein beta-7 OS=Homo sapiens OX=9606 GN=HSPB7 PE=1 SV=1	-3.794515061	0.000208654
P39060	Collagen alpha-1(XVIII) chain OS=Homo sapiens OX=9606 GN=COL18A1 PE=1 SV=5	3.972858831	0.00020912
Q9UM22	Mammalian ependymin-related protein 1 OS=Homo sapiens OX=9606 GN=EPDR1 PE=1 SV=2	1.45563258	0.000210184
Q9P2E9	Ribosome-binding protein 1 OS=Homo sapiens OX=9606 GN=RRBP1 PE=1 SV=5	6.572406437	0.000210492
P07451	Carbonic anhydrase 3 OS=Homo sapiens OX=9606 GN=CA3 PE=1 SV=3	-8.897819528	0.000212961
Q14980	Nuclear mitotic apparatus protein 1 OS=Homo sapiens OX=9606 GN=NUMA1 PE=1 SV=2	3.706022097	0.000213837
C9J798	Ras GTPase-activating protein 4B OS=Homo sapiens OX=9606 GN=RASA4B PE=3 SV=2	3.559765003	0.000216263
Q96PE7	Methylmalonyl-CoA epimerase, mitochondrial OS=Homo sapiens OX=9606 GN=MCEE PE=1 SV=1	4.783316737	0.000216375
Q6NVY1	3-hydroxyisobutyryl-CoA hydrolase, mitochondrial OS=Homo sapiens OX=9606 GN=HIBCH PE=1 SV=2	-2.750987919	0.000218683
P02144	Myoglobin OS=Homo sapiens OX=9606 GN=MB PE=1 SV=2	-5.515053495	0.000224826
P15121	Aldo-keto reductase family 1 member B1 OS=Homo sapiens OX=9606 GN=AKR1B1 PE=1 SV=3	-5.346592482	0.000225049
P38117	Electron transfer flavoprotein subunit beta OS=Homo sapiens OX=9606 GN=ETFB PE=1 SV=3	-6.151206748	0.000230104
Q15847	Adipogenesis regulatory factor OS=Homo sapiens OX=9606 GN=ADIRF PE=1 SV=1	3.147701618	0.000234813
P06753-5	Isoform 5 of Tropomyosin alpha-3 chain OS=Homo sapiens OX=9606 GN=TPM3	6.007304283	0.000238424
Q6UXN9	WD repeat-containing protein 82 OS=Homo sapiens OX=9606 GN=WDR82 PE=1 SV=1	4.997525247	0.00024681
O75190	DnaJ homolog subfamily B member 6 OS=Homo sapiens OX=9606 GN=DNAJB6 PE=1 SV=2	2.612063353	0.000247023

Q96A32	Myosin regulatory light chain 2, skeletal muscle isoform OS=Homo sapiens OX=9606 GN=MYLPF PE=2 SV=1	-7.146052234	0.000247396
P55884-2	Isoform 2 of Eukaryotic translation initiation factor 3 subunit B OS=Homo sapiens OX=9606 GN=EIF3B	3.168968657	0.000247733
Q8NI60	Atypical kinase COQ8A, mitochondrial OS=Homo sapiens OX=9606 GN=COQ8A PE=1 SV=1	-4.483049758	0.000249539
Q16718	NADH dehydrogenase [ubiquinone] 1 alpha subcomplex subunit 5 OS=Homo sapiens OX=9606 GN=NDUFA5 PE=1 SV=3	-4.602412988	0.000257804
O94919	Endonuclease domain-containing 1 protein OS=Homo sapiens OX=9606 GN=ENDOD1 PE=1 SV=2	3.02971996	0.000261487
P82979	SAP domain-containing ribonucleoprotein OS=Homo sapiens OX=9606 GN=SARNP PE=1 SV=3	7.090772411	0.000269698
P00488	Coagulation factor XIII A chain OS=Homo sapiens OX=9606 GN=F13A1 PE=1 SV=4	4.228634974	0.00028233
Q9NX46	ADP-ribose glycohydrolase ARH3 OS=Homo sapiens OX=9606 GN=ADPRHL2 PE=1 SV=1	7.819899161	0.000284273
Q9UKM9	RNA-binding protein Raly OS=Homo sapiens OX=9606 GN=RALY PE=1 SV=1	3.42739751	0.000289342
P40121	Macrophage-capping protein OS=Homo sapiens OX=9606 GN=CAPG PE=1 SV=2	6.969802091	0.000291711
P48643	T-complex protein 1 subunit epsilon OS=Homo sapiens OX=9606 GN=CCT5 PE=1 SV=1	-4.001843246	0.000292598
Q9Y2Q5	Ragulator complex protein LAMTOR2 OS=Homo sapiens OX=9606 GN=LAMTOR2 PE=1 SV=1	2.466419016	0.000293821
P25325-2	Isoform 2 of 3-mercaptopyruvate sulfurtransferase OS=Homo sapiens OX=9606 GN=MPST	6.022427385	0.000299531
Q9ULA0	Aspartyl aminopeptidase OS=Homo sapiens OX=9606 GN=DNPEP PE=1 SV=1	4.333364227	0.000300452
Q4VC31	Coiled-coil domain-containing protein 58 OS=Homo sapiens OX=9606 GN=CCDC58 PE=1 SV=1	6.90499602	0.000301261
A0A0B4J1Y9	Immunoglobulin heavy variable 3-72 OS=Homo sapiens OX=9606 GN=IGHV3-72 PE=3 SV=1	5.432007787	0.000308348
O75947	ATP synthase subunit d, mitochondrial OS=Homo sapiens OX=9606 GN=ATP5PD PE=1 SV=3	-2.497069147	0.000313434
P28161	Glutathione S-transferase Mu 2 OS=Homo sapiens OX=9606 GN=GSTM2 PE=1 SV=2	-4.904816468	0.000319288
Q96AB3-2	Isoform 2 of Isochorismatase domain-containing protein 2 OS=Homo sapiens OX=9606 GN=ISOC2	6.864516808	0.000319919
P20700	Lamin-B1 OS=Homo sapiens OX=9606 GN=LMNB1 PE=1 SV=2	4.468655397	0.000331924

P09622	Dihydrolipoyl dehydrogenase, mitochondrial OS=Homo sapiens OX=9606 GN=DLD PE=1 SV=2	-4.474562418	0.000340966
P29992	Guanine nucleotide-binding protein subunit alpha-11 OS=Homo sapiens OX=9606 GN=GNA11 PE=1 SV=2	5.286577985	0.000341052
P08651	Nuclear factor 1 C-type OS=Homo sapiens OX=9606 GN=NFIC PE=1 SV=2	5.145503012	0.000354488
P14324	Farnesyl pyrophosphate synthase OS=Homo sapiens OX=9606 GN=FDPS PE=1 SV=4	4.325204615	0.000359116
P35609	Alpha-actinin-2 OS=Homo sapiens OX=9606 GN=ACTN2 PE=1 SV=1	-7.204091788	0.000362344
O95425	Supervillin OS=Homo sapiens OX=9606 GN=SVIL PE=1 SV=2	3.448262127	0.000366588
P08133	Annexin A6 OS=Homo sapiens OX=9606 GN=ANXA6 PE=1 SV=3	-6.214661013	0.000374456
P00746	Complement factor D OS=Homo sapiens OX=9606 GN=CFD PE=1 SV=5	7.340238163	0.000380882
Q15185	Prostaglandin E synthase 3 OS=Homo sapiens OX=9606 GN=PTGES3 PE=1 SV=1	3.639779252	0.000381088
P40925-3	Isoform 3 of Malate dehydrogenase, cytoplasmic OS=Homo sapiens OX=9606 GN=MDH1	-6.198391403	0.000381126
O43414	ERI1 exoribonuclease 3 OS=Homo sapiens OX=9606 GN=ERI3 PE=1 SV=2	6.501611472	0.000382572
P62879	Guanine nucleotide-binding protein G(I)/G(S)/G(T) subunit beta-2 OS=Homo sapiens OX=9606 GN=GNB2 PE=1 SV=3	3.020803454	0.000388615
P02766	Transthyretin OS=Homo sapiens OX=9606 GN=TTR PE=1 SV=1	3.315722598	0.000391507
P13804	Electron transfer flavoprotein subunit alpha, mitochondrial OS=Homo sapiens OX=9606 GN=ETFA PE=1 SV=1	-4.732357291	0.000395305
P17540	Creatine kinase S-type, mitochondrial OS=Homo sapiens OX=9606 GN=CKMT2 PE=1 SV=2	-6.484670649	0.000403357
O75208	Ubiquinone biosynthesis protein COQ9, mitochondrial OS=Homo sapiens OX=9606 GN=COQ9 PE=1 SV=1	-2.647769727	0.000409137
Q92973	Transportin-1 OS=Homo sapiens OX=9606 GN=TNPO1 PE=1 SV=2	1.73766512	0.000412889
P15170-3	Isoform 3 of Eukaryotic peptide chain release factor GTP-binding subunit ERF3A OS=Homo sapiens OX=9606 GN=GSPT1	3.543650896	0.000417104
P09871	Complement C1s subcomponent OS=Homo sapiens OX=9606 GN=C1S PE=1 SV=1	6.231611797	0.00042231
P07951	Tropomyosin beta chain OS=Homo sapiens OX=9606 GN=TPM2 PE=1 SV=1	-7.289343142	0.000423115
P55084	Trifunctional enzyme subunit beta, mitochondrial OS=Homo sapiens OX=9606 GN=HADHB PE=1 SV=3	-6.05721769	0.000427089
P20674	Cytochrome c oxidase subunit 5A, mitochondrial OS=Homo sapiens OX=9606 GN=COX5A PE=1 SV=2	-6.392988822	0.000427518

Q8WX93	Palladin OS=Homo sapiens OX=9606 GN=PALLD PE=1 SV=3	2.158090237	0.000433558
O76070	Gamma-synuclein OS=Homo sapiens OX=9606 GN=SNCG PE=1 SV=2	3.336273578	0.000438408
Q5VTT5	Myomesin-3 OS=Homo sapiens OX=9606 GN=MYOM3 PE=1 SV=1	-7.609905126	0.000449864
Q13838-2	Isoform 2 of Spliceosome RNA helicase DDX39B OS=Homo sapiens OX=9606 GN=DDX39B	2.522132981	0.000454526
P62854	40S ribosomal protein S26 OS=Homo sapiens OX=9606 GN=RPS26 PE=1 SV=3	3.382537743	0.000460699
Q9UQ80	Proliferation-associated protein 2G4 OS=Homo sapiens OX=9606 GN=PA2G4 PE=1 SV=3	2.253602718	0.000474446
O00264	Membrane-associated progesterone receptor component 1 OS=Homo sapiens OX=9606 GN=PGRMC1 PE=1 SV=3	6.069856431	0.000475195
O43242	26S proteasome non-ATPase regulatory subunit 3 OS=Homo sapiens OX=9606 GN=PSMD3 PE=1 SV=2	2.084666258	0.000484451
Q9BYV2	Tripartite motif-containing protein 54 OS=Homo sapiens OX=9606 GN=TRIM54 PE=1 SV=3	4.258554258	0.000486243
P01877	Immunoglobulin heavy constant alpha 2 OS=Homo sapiens OX=9606 GN=IGHA2 PE=1 SV=4	5.169284047	0.000493622
P13639	Elongation factor 2 OS=Homo sapiens OX=9606 GN=EEF2 PE=1 SV=4	-6.516855302	0.000495014
P06733	Alpha-enolase OS=Homo sapiens OX=9606 GN=ENO1 PE=1 SV=2	-4.702487612	0.000496261
P02760	Protein AMBP OS=Homo sapiens OX=9606 GN=AMBP PE=1 SV=1	2.187167109	0.000497435
P50991	T-complex protein 1 subunit delta OS=Homo sapiens OX=9606 GN=CCT4 PE=1 SV=4	3.244448785	0.00050016
P14618-2	Isoform M1 of Pyruvate kinase PKM OS=Homo sapiens OX=9606 GN=PKM	-3.504874387	0.000501339
P52179-2	Isoform 2 of Myomesin-1 OS=Homo sapiens OX=9606 GN=MYOM1	-6.550865727	0.000516236
Q5TDH0-3	Isoform 3 of Protein DDI1 homolog 2 OS=Homo sapiens OX=9606 GN=DDI2	5.250988859	0.000529627
P31948-2	Isoform 2 of Stress-induced-phosphoprotein 1 OS=Homo sapiens OX=9606 GN=STIP1	-3.261431376	0.000534217
P60953	Cell division control protein 42 homolog OS=Homo sapiens OX=9606 GN=CDC42 PE=1 SV=2	3.065783656	0.000543243
P05976	Myosin light chain 1/3, skeletal muscle isoform OS=Homo sapiens OX=9606 GN=MYL1 PE=1 SV=3	-3.915619221	0.000550233
P38159	RNA-binding motif protein, X chromosome OS=Homo sapiens OX=9606 GN=RBMX PE=1 SV=3	3.451503687	0.000562818
P08237-3	Isoform 3 of ATP-dependent 6-phosphofructokinase, muscle type OS=Homo sapiens OX=9606 GN=PFKM	-7.917296683	0.000576382

Q8NDY3	[Protein ADP-ribosylarginine] hydrolase-like protein 1 OS=Homo sapiens OX=9606 GN=ADPRHL1 PE=2 SV=1	-2.273113828	0.000576797
P13611-2	Isoform V1 of Versican core protein OS=Homo sapiens OX=9606 GN=VCAN	5.851276938	0.000591637
P35232	Prohibitin OS=Homo sapiens OX=9606 GN=PHB PE=1 SV=1	-4.424401713	0.000593526
Q9NP98	Myozenin-1 OS=Homo sapiens OX=9606 GN=MYOZ1 PE=1 SV=1	-3.205072651	0.000595749
Q15327	Ankyrin repeat domain-containing protein 1 OS=Homo sapiens OX=9606 GN=ANKRD1 PE=1 SV=2	10.27069379	0.000629496
P23297	Protein S100-A1 OS=Homo sapiens OX=9606 GN=S100A1 PE=1 SV=2	-6.343792767	0.000635438
Q6YN16	Hydroxysteroid dehydrogenase-like protein 2 OS=Homo sapiens OX=9606 GN=HSDL2 PE=1 SV=1	-4.09205822	0.000637636
Q86TD4	Sarcalumenin OS=Homo sapiens OX=9606 GN=SRL PE=2 SV=2	-7.091387899	0.00064363
P04217	Alpha-1B-glycoprotein OS=Homo sapiens OX=9606 GN=A1BG PE=1 SV=4	4.632943668	0.000644335
P15502-4	Isoform 4 of Elastin OS=Homo sapiens OX=9606 GN=ELN	6.919007018	0.000648169
P80723	Brain acid soluble protein 1 OS=Homo sapiens OX=9606 GN=BASP1 PE=1 SV=2	8.975088939	0.000666605
P22061-2	Isoform 2 of Protein-L-isoaspartate(D-aspartate) O-methyltransferase OS=Homo sapiens OX=9606 GN=PCMT1	-5.467217412	0.000675262
Q6P587-3	Isoform 3 of Acylpyruvase FAHD1, mitochondrial OS=Homo sapiens OX=9606 GN=FAHD1	5.890272131	0.000684543
P15311	Ezrin OS=Homo sapiens OX=9606 GN=EZR PE=1 SV=4	3.693608448	0.000685791
Q92614	Unconventional myosin-XVIIIa OS=Homo sapiens OX=9606 GN=MYO18A PE=1 SV=3	-2.553090653	0.000686335
P0C0L4	Complement C4-A OS=Homo sapiens OX=9606 GN=C4A PE=1 SV=2	5.238909229	0.000694207
P09110	3-ketoacyl-CoA thiolase, peroxisomal OS=Homo sapiens OX=9606 GN=ACAA1 PE=1 SV=2	3.601887399	0.000703791
P30566	Adenylosuccinate lyase OS=Homo sapiens OX=9606 GN=ADSL PE=1 SV=2	2.052049642	0.000704221
P07195	L-lactate dehydrogenase B chain OS=Homo sapiens OX=9606 GN=LDHB PE=1 SV=2	-5.559419169	0.000708747
Q9UMS6	Synaptopodin-2 OS=Homo sapiens OX=9606 GN=SYNPO2 PE=1 SV=2	-3.02687125	0.000730353
P02787	Serotransferrin OS=Homo sapiens OX=9606 GN=TF PE=1 SV=3	-6.670673784	0.00073652
Q9UBF9	Myotilin OS=Homo sapiens OX=9606 GN=MYOT PE=1 SV=2	-2.456518701	0.000740927
Q9NVI7	ATPase family AAA domain-containing protein 3A OS=Homo sapiens OX=9606 GN=ATAD3A PE=1 SV=2	4.496804801	0.000742017

P49411	Elongation factor Tu, mitochondrial OS=Homo sapiens OX=9606 GN=TUFM PE=1 SV=2	-4.738353799	0.000751073
P40939	Trifunctional enzyme subunit alpha, mitochondrial OS=Homo sapiens OX=9606 GN=HADHA PE=1 SV=2	-3.488093235	0.000756125
Q9Y6E0	Serine/threonine-protein kinase 24 OS=Homo sapiens OX=9606 GN=STK24 PE=1 SV=1	5.782661115	0.000758749
Q99807	5-demethoxyubiquinone hydroxylase, mitochondrial OS=Homo sapiens OX=9606 GN=COQ7 PE=1 SV=3	5.450421635	0.000758894
Q9NQW7-3	Isoform 3 of Xaa-Pro aminopeptidase 1 OS=Homo sapiens OX=9606 GN=XPNPEP1	4.461224713	0.000761805
O75251	NADH dehydrogenase [ubiquinone] iron-sulfur protein 7, mitochondrial OS=Homo sapiens OX=9606 GN=NDUFS7 PE=1 SV=3	-1.353331636	0.000762796
Q14324	Myosin-binding protein C, fast-type OS=Homo sapiens OX=9606 GN=MYBPC2 PE=1 SV=2	-5.794591882	0.000770797
Q16363	Laminin subunit alpha-4 OS=Homo sapiens OX=9606 GN=LAMA4 PE=1 SV=4	5.234804311	0.00078438
Q96GK7	Fumarylacetoacetate hydrolase domain-containing protein 2A OS=Homo sapiens OX=9606 GN=FAHD2A PE=1 SV=1	4.478136062	0.000807813
Q9Y2J8	Protein-arginine deiminase type-2 OS=Homo sapiens OX=9606 GN=PADI2 PE=1 SV=2	-4.693225445	0.000816964
P13640	Metallothionein-1G OS=Homo sapiens OX=9606 GN=MT1G PE=1 SV=2	7.158033982	0.000878593
Q12931	Heat shock protein 75 kDa, mitochondrial OS=Homo sapiens OX=9606 GN=TRAP1 PE=1 SV=3	3.228597276	0.000913838
P56381	ATP synthase subunit epsilon, mitochondrial OS=Homo sapiens OX=9606 GN=ATP5F1E PE=1 SV=2	-4.329077698	0.000914341
O14958	Calsequestrin-2 OS=Homo sapiens OX=9606 GN=CASQ2 PE=1 SV=2	-4.9837394	0.000933868
Q06830	Peroxiredoxin-1 OS=Homo sapiens OX=9606 GN=PRDX1 PE=1 SV=1	-4.343540281	0.000959365
Q5VW32	BRO1 domain-containing protein BROX OS=Homo sapiens OX=9606 GN=BROX PE=1 SV=1	2.412790264	0.000959684
Q9NR56	Muscleblind-like protein 1 OS=Homo sapiens OX=9606 GN=MBNL1 PE=1 SV=2	2.830104605	0.000978767
P22102	Trifunctional purine biosynthetic protein adenosine-3 OS=Homo sapiens OX=9606 GN=GART PE=1 SV=1	4.351114143	0.001010215
E9PAV3	Nascent polypeptide-associated complex subunit alpha, muscle-specific form OS=Homo sapiens OX=9606 GN=NACA PE=1 SV=1	-6.90781228	0.001016852
Q58WW2-3	Isoform 3 of DDB1- and CUL4-associated factor 6 OS=Homo sapiens OX=9606 GN=DCAF6	5.141169053	0.001018232

Q9UHA4	Ragulator complex protein LAMTOR3 OS=Homo sapiens OX=9606 GN=LAMTOR3 PE=1 SV=1	4.139941719	0.001030343
A6NNS2	Dehydrogenase/reductase SDR family member 7C OS=Homo sapiens OX=9606 GN=DHRS7C PE=2 SV=3	3.486791007	0.001039699
Q9HCU5	Prolactin regulatory element-binding protein OS=Homo sapiens OX=9606 GN=PREB PE=1 SV=2	6.015105999	0.001051393
P55786	Puromycin-sensitive aminopeptidase OS=Homo sapiens OX=9606 GN=NPEPPS PE=1 SV=2	-5.862528714	0.001052191
Q9Y371-2	Isoform 2 of Endophilin-B1 OS=Homo sapiens OX=9606 GN=SH3GLB1	2.055702561	0.001052624
Q8WWI1	LIM domain only protein 7 OS=Homo sapiens OX=9606 GN=LMO7 PE=1 SV=3	4.596635005	0.001053097
P08238	Heat shock protein HSP 90-beta OS=Homo sapiens OX=9606 GN=HSP90AB1 PE=1 SV=4	-2.140430551	0.0010701
P48444	Coatomer subunit delta OS=Homo sapiens OX=9606 GN=ARCN1 PE=1 SV=1	4.841285077	0.001071958
P12081	Histidine--tRNA ligase, cytoplasmic OS=Homo sapiens OX=9606 GN=HARS PE=1 SV=2	2.596591361	0.001094871
P48047	ATP synthase subunit O, mitochondrial OS=Homo sapiens OX=9606 GN=ATP5PO PE=1 SV=1	-5.775215905	0.00114701
P08590	Myosin light chain 3 OS=Homo sapiens OX=9606 GN=MYL3 PE=1 SV=3	-6.816295083	0.00115118
Q8TB45	DEP domain-containing mTOR-interacting protein OS=Homo sapiens OX=9606 GN=DEPTOR PE=1 SV=2	3.690447586	0.001165829
P12111-4	Isoform 4 of Collagen alpha-3(VI) chain OS=Homo sapiens OX=9606 GN=COL6A3	6.367473568	0.001187351
P55042	GTP-binding protein RAD OS=Homo sapiens OX=9606 GN=RRAD PE=1 SV=2	3.642880794	0.001228321
P54296	Myomesin-2 OS=Homo sapiens OX=9606 GN=MYOM2 PE=1 SV=2	-7.002305375	0.001237596
O96000	NADH dehydrogenase [ubiquinone] 1 beta subcomplex subunit 10 OS=Homo sapiens OX=9606 GN=NDUFB10 PE=1 SV=3	-2.455555936	0.001239116
Q9H3K6	BolA-like protein 2 OS=Homo sapiens OX=9606 GN=BOLA2 PE=1 SV=1	2.474378052	0.001244881
O15273	Telethonin OS=Homo sapiens OX=9606 GN=TCAP PE=1 SV=1	4.188463733	0.001245754
O60234	Glia maturation factor gamma OS=Homo sapiens OX=9606 GN=GMFG PE=1 SV=1	2.897613651	0.001261726
Q9NX14-2	Isoform 2 of NADH dehydrogenase [ubiquinone] 1 beta subcomplex subunit 11, mitochondrial OS=Homo sapiens OX=9606 GN=NDUFB11	5.766632514	0.001286906
Q12955	Ankyrin-3 OS=Homo sapiens OX=9606 GN=ANK3 PE=1 SV=3	3.790332208	0.001292244

P53597	Succinate--CoA ligase [ADP/GDP-forming] subunit alpha, mitochondrial OS=Homo sapiens OX=9606 GN=SUCLG1 PE=1 SV=4	-3.202659859	0.001305934
P54727	UV excision repair protein RAD23 homolog B OS=Homo sapiens OX=9606 GN=RAD23B PE=1 SV=1	-3.567146034	0.001314867
P30046	D-dopachrome decarboxylase OS=Homo sapiens OX=9606 GN=DDT PE=1 SV=3	-3.033177911	0.001320362
P50895	Basal cell adhesion molecule OS=Homo sapiens OX=9606 GN=BCAM PE=1 SV=2	3.461693448	0.001340419
Q99471	Prefoldin subunit 5 OS=Homo sapiens OX=9606 GN=PFDN5 PE=1 SV=2	4.486123764	0.001393689
Q13508	Ecto-ADP-ribosyltransferase 3 OS=Homo sapiens OX=9606 GN=ART3 PE=1 SV=2	3.362847717	0.001406966
Q8NBS9	Thioredoxin domain-containing protein 5 OS=Homo sapiens OX=9606 GN=TXNDC5 PE=1 SV=2	4.824470568	0.001440472
Q8WZ42-6	Isoform 6 of Titin OS=Homo sapiens OX=9606 GN=TTN	4.824936494	0.001496379
Q9H511	Kelch-like protein 31 OS=Homo sapiens OX=9606 GN=KLHL31 PE=2 SV=1	4.624839636	0.001513852
P00966	Argininosuccinate synthase OS=Homo sapiens OX=9606 GN=ASS1 PE=1 SV=2	-1.464340349	0.001521962
Q99816	Tumor susceptibility gene 101 protein OS=Homo sapiens OX=9606 GN=TSG101 PE=1 SV=2	2.686452104	0.001533463
P10809	60 kDa heat shock protein, mitochondrial OS=Homo sapiens OX=9606 GN=HSPD1 PE=1 SV=2	-6.013809677	0.001543188
Q15075	Early endosome antigen 1 OS=Homo sapiens OX=9606 GN=EEA1 PE=1 SV=2	2.634792816	0.001545156
P35580-4	Isoform 4 of Myosin-10 OS=Homo sapiens OX=9606 GN=MYH10	5.026904297	0.001552293
Q01432-4	Isoform 2 of AMP deaminase 3 OS=Homo sapiens OX=9606 GN=AMPD3	9.231497161	0.001571387
O00159	Unconventional myosin-Ic OS=Homo sapiens OX=9606 GN=MYO1C PE=1 SV=4	4.603007211	0.001578017
Q71U36	Tubulin alpha-1A chain OS=Homo sapiens OX=9606 GN=TUBA1A PE=1 SV=1	3.323514054	0.00160625
P11310-2	Isoform 2 of Medium-chain specific acyl-CoA dehydrogenase, mitochondrial OS=Homo sapiens OX=9606 GN=ACADM	-3.808789523	0.001606373
P30038	Delta-1-pyrroline-5-carboxylate dehydrogenase, mitochondrial OS=Homo sapiens OX=9606 GN=ALDH4A1 PE=1 SV=3	-5.693505491	0.001666099
Q9BUR5	MICOS complex subunit MIC26 OS=Homo sapiens OX=9606 GN=APOO PE=1 SV=1	5.921873323	0.001668947
P04264	Keratin, type II cytoskeletal 1 OS=Homo sapiens OX=9606 GN=KRT1 PE=1 SV=6	-4.319587227	0.001682178
O43772	Mitochondrial carnitine/acylcarnitine carrier protein OS=Homo sapiens OX=9606 GN=SLC25A20 PE=1 SV=1	2.658700966	0.001682723
P35241-5	Isoform 5 of Radixin OS=Homo sapiens OX=9606 GN=RDX	-3.123202942	0.001708924

P08559-4	Isoform 4 of Pyruvate dehydrogenase E1 component subunit alpha, somatic form, mitochondrial OS=Homo sapiens OX=9606 GN=PDHA1	-3.115417554	0.001724298
Q9UNZ2-5	Isoform 3 of NSFL1 cofactor p47 OS=Homo sapiens OX=9606 GN=NSFL1C	1.911469241	0.001756724
P47914	60S ribosomal protein L29 OS=Homo sapiens OX=9606 GN=RPL29 PE=1 SV=2	2.201830106	0.001761769
O75431	Metaxin-2 OS=Homo sapiens OX=9606 GN=MTX2 PE=1 SV=1	4.146722145	0.001779946
P49354	Protein farnesyltransferase/geranylgeranyltransferase type-1 subunit alpha OS=Homo sapiens OX=9606 GN=FNTA PE=1 SV=1	4.997850605	0.001797689
P30740	Leukocyte elastase inhibitor OS=Homo sapiens OX=9606 GN=SERPINB1 PE=1 SV=1	-2.138420228	0.001822905
O43676	NADH dehydrogenase [ubiquinone] 1 beta subcomplex subunit 3 OS=Homo sapiens OX=9606 GN=NDUFB3 PE=1 SV=3	-3.250030537	0.001827602
Q9H9P8	L-2-hydroxyglutarate dehydrogenase, mitochondrial OS=Homo sapiens OX=9606 GN=L2HGDH PE=1 SV=3	2.981777629	0.001863609
P36959	GMP reductase 1 OS=Homo sapiens OX=9606 GN=GMPR PE=1 SV=1	4.026940406	0.001864101
P07737	Profilin-1 OS=Homo sapiens OX=9606 GN=PFN1 PE=1 SV=2	-4.564637432	0.001871584
O15511-2	Isoform 2 of Actin-related protein 2/3 complex subunit 5 OS=Homo sapiens OX=9606 GN=ARPC5	4.257685004	0.001920216
Q14192	Four and a half LIM domains protein 2 OS=Homo sapiens OX=9606 GN=FHL2 PE=1 SV=3	4.49212171	0.001964295
P47755	F-actin-capping protein subunit alpha-2 OS=Homo sapiens OX=9606 GN=CAPZA2 PE=1 SV=3	-4.916044357	0.001977827
Q8N335	Glycerol-3-phosphate dehydrogenase 1-like protein OS=Homo sapiens OX=9606 GN=GPD1L PE=1 SV=1	2.818793095	0.00201272
P11142	Heat shock cognate 71 kDa protein OS=Homo sapiens OX=9606 GN=HSPA8 PE=1 SV=1	-6.196397631	0.00205731
O95210	Starch-binding domain-containing protein 1 OS=Homo sapiens OX=9606 GN=STBD1 PE=1 SV=1	-4.752116932	0.002092632
O95816	BAG family molecular chaperone regulator 2 OS=Homo sapiens OX=9606 GN=BAG2 PE=1 SV=1	6.062750323	0.002107622
Q969G5	Caveolae-associated protein 3 OS=Homo sapiens OX=9606 GN=CAVIN3 PE=1 SV=3	3.249141363	0.002127018
P05388	60S acidic ribosomal protein P0 OS=Homo sapiens OX=9606 GN=RPLP0 PE=1 SV=1	-4.635316851	0.002132837
P21695	Glycerol-3-phosphate dehydrogenase [NAD(+)], cytoplasmic OS=Homo sapiens OX=9606 GN=GPD1 PE=1 SV=4	-3.698979229	0.002262578

P49247	Ribose-5-phosphate isomerase OS=Homo sapiens OX=9606 GN=RPIA PE=1 SV=3	4.241401256	0.002306147
Q9BS26	Endoplasmic reticulum resident protein 44 OS=Homo sapiens OX=9606 GN=ERP44 PE=1 SV=1	5.8197698	0.002318498
Q9NQC3-3	Isoform C of Reticulon-4 OS=Homo sapiens OX=9606 GN=RTN4	4.360788553	0.002335605
A7E2Y1	Myosin-7B OS=Homo sapiens OX=9606 GN=MYH7B PE=1 SV=4	4.062554326	0.002395526
Q96DG6	Carboxymethylenebutenolidase homolog OS=Homo sapiens OX=9606 GN=CMBL PE=1 SV=1	-3.505117023	0.002430517
Q92688	Acidic leucine-rich nuclear phosphoprotein 32 family member B OS=Homo sapiens OX=9606 GN=ANP32B PE=1 SV=1	4.034759597	0.002453634
O95573	Long-chain-fatty-acid--CoA ligase 3 OS=Homo sapiens OX=9606 GN=ACSL3 PE=1 SV=3	3.465883252	0.002467139
P31415	Calsequestrin-1 OS=Homo sapiens OX=9606 GN=CASQ1 PE=1 SV=3	-5.032290323	0.00248851
Q99541	Perilipin-2 OS=Homo sapiens OX=9606 GN=PLIN2 PE=1 SV=2	3.017824043	0.00250145
O75521	Enoyl-CoA delta isomerase 2, mitochondrial OS=Homo sapiens OX=9606 GN=ECI2 PE=1 SV=4	3.588892898	0.002540592
Q99584	Protein S100-A13 OS=Homo sapiens OX=9606 GN=S100A13 PE=1 SV=1	1.791328544	0.002560059
P46926	Glucosamine-6-phosphate isomerase 1 OS=Homo sapiens OX=9606 GN=GNPDA1 PE=1 SV=1	4.513806258	0.002594743
O43813	Glutathione S-transferase LANCL1 OS=Homo sapiens OX=9606 GN=LANCL1 PE=1 SV=1	1.189520163	0.002651352
Q14353	Guanidinoacetate N-methyltransferase OS=Homo sapiens OX=9606 GN=GAMT PE=1 SV=1	-3.038553531	0.002657854
P01860	Immunoglobulin heavy constant gamma 3 OS=Homo sapiens OX=9606 GN=IGHG3 PE=1 SV=2	3.12881617	0.002757709
P12004	Proliferating cell nuclear antigen OS=Homo sapiens OX=9606 GN=PCNA PE=1 SV=1	4.428517036	0.002769003
Q00325	Phosphate carrier protein, mitochondrial OS=Homo sapiens OX=9606 GN=SLC25A3 PE=1 SV=2	-1.867540219	0.002781401
P17858-2	Isoform 2 of ATP-dependent 6-phosphofructokinase, liver type OS=Homo sapiens OX=9606 GN=PFKL	2.59794469	0.002849597
P31949	Protein S100-A11 OS=Homo sapiens OX=9606 GN=S100A11 PE=1 SV=2	2.021684611	0.002953867
P62979	Ubiquitin-40S ribosomal protein S27a OS=Homo sapiens OX=9606 GN=RPS27A PE=1 SV=2	4.117662502	0.00300637
P45381	Aspartoacylase OS=Homo sapiens OX=9606 GN=ASPA PE=1 SV=1	3.312374024	0.003015259

P00441	Superoxide dismutase [Cu-Zn] OS=Homo sapiens OX=9606 GN=SOD1 PE=1 SV=2	-2.621562927	0.00303357
Q8N3D4	EH domain-binding protein 1-like protein 1 OS=Homo sapiens OX=9606 GN=EHBP1L1 PE=1 SV=2	5.457138775	0.003059794
P42765	3-ketoacyl-CoA thiolase, mitochondrial OS=Homo sapiens OX=9606 GN=ACAA2 PE=1 SV=2	-5.431160177	0.003080801
P19367-3	Isoform 3 of Hexokinase-1 OS=Homo sapiens OX=9606 GN=HK1	-5.231218362	0.003108197
Q13423	NAD(P) transhydrogenase, mitochondrial OS=Homo sapiens OX=9606 GN=NNT PE=1 SV=3	-3.606282985	0.003136478
P05997	Collagen alpha-2(V) chain OS=Homo sapiens OX=9606 GN=COL5A2 PE=1 SV=3	2.530989103	0.003197568
Q9BUF5	Tubulin beta-6 chain OS=Homo sapiens OX=9606 GN=TUBB6 PE=1 SV=1	4.730043555	0.0032617
P23528	Cofilin-1 OS=Homo sapiens OX=9606 GN=CFL1 PE=1 SV=3	2.948979837	0.003359871
Q9NVA2-2	Isoform 2 of Septin-11 OS=Homo sapiens OX=9606 GN=SEPTIN11	5.600183185	0.003381761
P68133	Actin, alpha skeletal muscle OS=Homo sapiens OX=9606 GN=ACTA1 PE=1 SV=1	-5.943328718	0.003393258
Q6ZVM7	TOM1-like protein 2 OS=Homo sapiens OX=9606 GN=TOM1L2 PE=1 SV=1	3.881259914	0.003396861
Q9UN37	Vacuolar protein sorting-associated protein 4A OS=Homo sapiens OX=9606 GN=VPS4A PE=1 SV=1	3.703197574	0.003425385
O43920	NADH dehydrogenase [ubiquinone] iron-sulfur protein 5 OS=Homo sapiens OX=9606 GN=NDUFS5 PE=1 SV=3	-4.915204384	0.003428525
P15104	Glutamine synthetase OS=Homo sapiens OX=9606 GN=GLUL PE=1 SV=4	3.141296362	0.003576125
Q9Y235	C->U-editing enzyme APOBEC-2 OS=Homo sapiens OX=9606 GN=APOBEC2 PE=1 SV=1	-3.448336839	0.003606386
P30153	Serine/threonine-protein phosphatase 2A 65 kDa regulatory subunit A alpha isoform OS=Homo sapiens OX=9606 GN=PPP2R1A PE=1 SV=4	-3.432403636	0.003624122
O95292	Vesicle-associated membrane protein-associated protein B/C OS=Homo sapiens OX=9606 GN=VAPB PE=1 SV=3	-3.334972046	0.003634782
Q9NWU1	3-oxoacyl-[acyl-carrier-protein] synthase, mitochondrial OS=Homo sapiens OX=9606 GN=OXSM PE=1 SV=1	8.32229015	0.003711032
Q8NBU5	ATPase family AAA domain-containing protein 1 OS=Homo sapiens OX=9606 GN=ATAD1 PE=1 SV=1	2.595005768	0.003716371
P48788	Troponin I, fast skeletal muscle OS=Homo sapiens OX=9606 GN=TNNI2 PE=1 SV=2	-4.802459734	0.003813214
Q16821	Protein phosphatase 1 regulatory subunit 3A OS=Homo sapiens OX=9606 GN=PPP1R3A PE=1 SV=3	2.463107551	0.00386824

Q6ZMU5	Tripartite motif-containing protein 72 OS=Homo sapiens OX=9606 GN=TRIM72 PE=1 SV=2	-3.933254899	0.003983203
P07951-3	Isoform 3 of Tropomyosin beta chain OS=Homo sapiens OX=9606 GN=TPM2	2.213326687	0.004002828
P46976	Glycogenin-1 OS=Homo sapiens OX=9606 GN=GYG1 PE=1 SV=4	-3.922823396	0.004094172
Q9H1R3	Myosin light chain kinase 2, skeletal/cardiac muscle OS=Homo sapiens OX=9606 GN=MYLK2 PE=1 SV=3	-2.892821352	0.004109687
P21589	5'-nucleotidase OS=Homo sapiens OX=9606 GN=NT5E PE=1 SV=1	4.690227818	0.00413204
Q8NFV4	Protein ABHD11 OS=Homo sapiens OX=9606 GN=ABHD11 PE=1 SV=1	3.353181734	0.004189513
P11216	Glycogen phosphorylase, brain form OS=Homo sapiens OX=9606 GN=PYGB PE=1 SV=5	3.528320894	0.004231489
P43652	Afamin OS=Homo sapiens OX=9606 GN=AFM PE=1 SV=1	3.750451961	0.004309501
O75923-8	Isoform 8 of Dysferlin OS=Homo sapiens OX=9606 GN=DYSF	-4.287591266	0.004412601
P31946	14-3-3 protein beta/alpha OS=Homo sapiens OX=9606 GN=YWHAB PE=1 SV=3	1.839820224	0.004484319
P0DMV9	Heat shock 70 kDa protein 1B OS=Homo sapiens OX=9606 GN=HSPA1B PE=1 SV=1	-3.483884932	0.00452417
Q14204	Cytoplasmic dynein 1 heavy chain 1 OS=Homo sapiens OX=9606 GN=DYNC1H1 PE=1 SV=5	-4.601613883	0.004537475
O15371	Eukaryotic translation initiation factor 3 subunit D OS=Homo sapiens OX=9606 GN=EIF3D PE=1 SV=1	3.520541576	0.004552221
Q8N142	Adenylosuccinate synthetase isozyme 1 OS=Homo sapiens OX=9606 GN=ADSS1 PE=1 SV=1	-2.205136702	0.004587113
Q9UBR2	Cathepsin Z OS=Homo sapiens OX=9606 GN=CTSZ PE=1 SV=1	2.793510333	0.004632088
P06744	Glucose-6-phosphate isomerase OS=Homo sapiens OX=9606 GN=GPI PE=1 SV=4	-6.231298531	0.004681098
P23141-2	Isoform 2 of Liver carboxylesterase 1 OS=Homo sapiens OX=9606 GN=CES1	7.027981387	0.004715365
P25311	Zinc-alpha-2-glycoprotein OS=Homo sapiens OX=9606 GN=AZGP1 PE=1 SV=2	2.227361912	0.004734436
P25705	ATP synthase subunit alpha, mitochondrial OS=Homo sapiens OX=9606 GN=ATP5F1A PE=1 SV=1	-2.508316344	0.004754272
P55290-4	Isoform 4 of Cadherin-13 OS=Homo sapiens OX=9606 GN=CDH13	-4.245164399	0.004801311
P02462	Collagen alpha-1(IV) chain OS=Homo sapiens OX=9606 GN=COL4A1 PE=1 SV=4	4.574301181	0.004825393
O15498	Synaptobrevin homolog YKT6 OS=Homo sapiens OX=9606 GN=YKT6 PE=1 SV=1	3.492388431	0.00486589
Q99798	Aconitate hydratase, mitochondrial OS=Homo sapiens OX=9606 GN=ACO2 PE=1 SV=2	-2.714491607	0.005029111
Q16595	Frataxin, mitochondrial OS=Homo sapiens OX=9606 GN=FXN PE=1 SV=2	4.63414187	0.005283713

A0A0C4DH31	Immunoglobulin heavy variable 1-18 OS=Homo sapiens OX=9606 GN=IGHV1-18 PE=3 SV=1	8.2722152	0.005399908
Q9GZZ1	N-alpha-acetyltransferase 50 OS=Homo sapiens OX=9606 GN=NAA50 PE=1 SV=1	3.7440533	0.005568446
P53990-5	Isoform 5 of IST1 homolog OS=Homo sapiens OX=9606 GN=IST1	1.508642761	0.005675893
O75339	Cartilage intermediate layer protein 1 OS=Homo sapiens OX=9606 GN=CILP PE=1 SV=4	4.068190895	0.005801736
Q9HB71	Calcyclin-binding protein OS=Homo sapiens OX=9606 GN=CACYBP PE=1 SV=2	3.971244604	0.005844081
Q16851	UTP--glucose-1-phosphate uridylyltransferase OS=Homo sapiens OX=9606 GN=UGP2 PE=1 SV=5	-5.929897671	0.005859471
P23434	Glycine cleavage system H protein, mitochondrial OS=Homo sapiens OX=9606 GN=GCSH PE=1 SV=2	3.528747908	0.005894558
P56181	NADH dehydrogenase [ubiquinone] flavoprotein 3, mitochondrial OS=Homo sapiens OX=9606 GN=NDUFV3 PE=1 SV=2	2.909364443	0.006022
P02774-3	Isoform 3 of Vitamin D-binding protein OS=Homo sapiens OX=9606 GN=GC	-1.735283279	0.006040994
Q86VF7	Nebulin-related-anchoring protein OS=Homo sapiens OX=9606 GN=NRAP PE=1 SV=2	-5.459429488	0.006106189
P19404	NADH dehydrogenase [ubiquinone] flavoprotein 2, mitochondrial OS=Homo sapiens OX=9606 GN=NDUFV2 PE=1 SV=2	-2.811111168	0.006127169
P27824-2	Isoform 2 of Calnexin OS=Homo sapiens OX=9606 GN=CANX	-3.836805711	0.006135648
Q16775	Hydroxyacylglutathione hydrolase, mitochondrial OS=Homo sapiens OX=9606 GN=HAGH PE=1 SV=2	-3.693667362	0.006215714
P50395	Rab GDP dissociation inhibitor beta OS=Homo sapiens OX=9606 GN=GDI2 PE=1 SV=2	-4.160123643	0.00624747
Q9Y623	Myosin-4 OS=Homo sapiens OX=9606 GN=MYH4 PE=2 SV=2	-5.322223195	0.006251673
P28066	Proteasome subunit alpha type-5 OS=Homo sapiens OX=9606 GN=PSMA5 PE=1 SV=3	-1.26111629	0.006278309
P50461	Cysteine and glycine-rich protein 3 OS=Homo sapiens OX=9606 GN=CSRP3 PE=1 SV=1	-2.222489562	0.006312196
P51659	Peroxisomal multifunctional enzyme type 2 OS=Homo sapiens OX=9606 GN=HSD17B4 PE=1 SV=3	4.480080696	0.006331898
Q8IYU8	Calcium uptake protein 2, mitochondrial OS=Homo sapiens OX=9606 GN=MICU2 PE=1 SV=2	5.227538749	0.006457879
Q14203	Dynactin subunit 1 OS=Homo sapiens OX=9606 GN=DCTN1 PE=1 SV=3	2.339607713	0.006467326
P00352	Retinal dehydrogenase 1 OS=Homo sapiens OX=9606 GN=ALDH1A1 PE=1 SV=2	-5.293468223	0.006624316
P00387-3	Isoform 3 of NADH-cytochrome b5 reductase 3 OS=Homo sapiens OX=9606 GN=CYB5R3	-3.428654537	0.006706058

Q13619	Cullin-4A OS=Homo sapiens OX=9606 GN=CUL4A PE=1 SV=3	3.281637749	0.006873358
Q03013	Glutathione S-transferase Mu 4 OS=Homo sapiens OX=9606 GN=GSTM4 PE=1 SV=3	8.253032933	0.007094322
P19652	Alpha-1-acid glycoprotein 2 OS=Homo sapiens OX=9606 GN=ORM2 PE=1 SV=2	2.574008155	0.007146436
Q8WW22-2	Isoform 2 of DnaJ homolog subfamily A member 4 OS=Homo sapiens OX=9606 GN=DNAJA4	4.590669745	0.007174615
Q6NZI2	Caveolae-associated protein 1 OS=Homo sapiens OX=9606 GN=CAVIN1 PE=1 SV=1	-4.869946061	0.007262186
P51888	Prolargin OS=Homo sapiens OX=9606 GN=PRELP PE=1 SV=1	-4.15008667	0.007492163
O15061	Synemin OS=Homo sapiens OX=9606 GN=SYNM PE=1 SV=2	7.283245184	0.007618383
O75489	NADH dehydrogenase [ubiquinone] iron-sulfur protein 3, mitochondrial OS=Homo sapiens OX=9606 GN=NDUFS3 PE=1 SV=1	-2.884957252	0.007673521
P52564	Dual specificity mitogen-activated protein kinase kinase 6 OS=Homo sapiens OX=9606 GN=MAP2K6 PE=1 SV=1	-1.407764912	0.007694121
Q14315	Filamin-C OS=Homo sapiens OX=9606 GN=FLNC PE=1 SV=3	-6.1339999	0.00774187
Q13643	Four and a half LIM domains protein 3 OS=Homo sapiens OX=9606 GN=FHL3 PE=1 SV=4	-2.497496315	0.007787901
P18206	Vinculin OS=Homo sapiens OX=9606 GN=VCL PE=1 SV=4	-5.367283617	0.007796713
Q96HC4-2	Isoform 2 of PDZ and LIM domain protein 5 OS=Homo sapiens OX=9606 GN=PDLIM5	2.654718299	0.007905498
P24043	Laminin subunit alpha-2 OS=Homo sapiens OX=9606 GN=LAMA2 PE=1 SV=4	-4.18347285	0.00794881
Q9NVD7	Alpha-parvin OS=Homo sapiens OX=9606 GN=PARVA PE=1 SV=1	6.014194724	0.008033783
P21817	Ryanodine receptor 1 OS=Homo sapiens OX=9606 GN=RYR1 PE=1 SV=3	-5.834051049	0.008295293
Q9H299	SH3 domain-binding glutamic acid-rich-like protein 3 OS=Homo sapiens OX=9606 GN=SH3BGRL3 PE=1 SV=1	5.19318485	0.008301848
Q3LXA3	Triokinase/FMN cyclase OS=Homo sapiens OX=9606 GN=TKFC PE=1 SV=2	6.905396091	0.008334675
P04844	Dolichyl-diphosphooligosaccharide--protein glycosyltransferase subunit 2 OS=Homo sapiens OX=9606 GN=RPN2 PE=1 SV=3	-2.231409711	0.008616077
A0A0A0MRZ8	Immunoglobulin kappa variable 3D-11 OS=Homo sapiens OX=9606 GN=IGKV3D-11 PE=3 SV=6	4.179772513	0.008618843
Q9Y281	Cofilin-2 OS=Homo sapiens OX=9606 GN=CFL2 PE=1 SV=1	-2.518788422	0.008791945
Q96EY8	Corrinoid adenosyltransferase OS=Homo sapiens OX=9606 GN=MMAB PE=1 SV=1	3.848574594	0.008874686
P49821	NADH dehydrogenase [ubiquinone] flavoprotein 1, mitochondrial OS=Homo sapiens OX=9606 GN=NDUFV1 PE=1 SV=4	-5.540694333	0.009081207

Q13151	Heterogeneous nuclear ribonucleoprotein A0 OS=Homo sapiens OX=9606 GN=HNRNPA0 PE=1 SV=1	3.677546945	0.009196281
Q13825	Methylglutaconyl-CoA hydratase, mitochondrial OS=Homo sapiens OX=9606 GN=AUH PE=1 SV=1	3.089158863	0.009218301
P01876	Immunoglobulin heavy constant alpha 1 OS=Homo sapiens OX=9606 GN=IGHA1 PE=1 SV=2	2.42626524	0.009230532
P05452	Tetranectin OS=Homo sapiens OX=9606 GN=CLEC3B PE=1 SV=3	4.775877732	0.009235806
Q13884	Beta-1-syntrophin OS=Homo sapiens OX=9606 GN=SNTB1 PE=1 SV=3	2.521399396	0.009249584
P61626	Lysozyme C OS=Homo sapiens OX=9606 GN=LYZ PE=1 SV=1	5.682377058	0.009317515
Q9H479	Fructosamine-3-kinase OS=Homo sapiens OX=9606 GN=FN3K PE=1 SV=1	5.331336551	0.009477326
A0A1B0GVR7	Protein FAM240C OS=Homo sapiens OX=9606 GN=FAM240C PE=3 SV=1	5.918360628	0.00949006
Q16531	DNA damage-binding protein 1 OS=Homo sapiens OX=9606 GN=DDB1 PE=1 SV=1	-1.148537099	0.009494515
P49748-3	Isoform 3 of Very long-chain specific acyl-CoA dehydrogenase, mitochondrial OS=Homo sapiens OX=9606 GN=ACADVL	-2.938973187	0.009501841
P14854	Cytochrome c oxidase subunit 6B1 OS=Homo sapiens OX=9606 GN=COX6B1 PE=1 SV=2	-2.274460077	0.009579111
Q9UH99-2	Isoform 2 of SUN domain-containing protein 2 OS=Homo sapiens OX=9606 GN=SUN2	5.066255127	0.009740863
P07339	Cathepsin D OS=Homo sapiens OX=9606 GN=CTSD PE=1 SV=1	-3.853211724	0.009780118
Q00872-7	Isoform 7 of Myosin-binding protein C, slow-type OS=Homo sapiens OX=9606 GN=MYBPC1	4.257292936	0.009899929
P27105	Erythrocyte band 7 integral membrane protein OS=Homo sapiens OX=9606 GN=STOM PE=1 SV=3	2.161835335	0.009900714
P24539	ATP synthase F(0) complex subunit B1, mitochondrial OS=Homo sapiens OX=9606 GN=ATP5PB PE=1 SV=2	-1.910359356	0.010017358
P21266	Glutathione S-transferase Mu 3 OS=Homo sapiens OX=9606 GN=GSTM3 PE=1 SV=3	-3.855640993	0.010149417
P63279	SUMO-conjugating enzyme UBC9 OS=Homo sapiens OX=9606 GN=UBE2I PE=1 SV=1	2.940877127	0.010256731
P78371	T-complex protein 1 subunit beta OS=Homo sapiens OX=9606 GN=CCT2 PE=1 SV=4	-3.604096085	0.010265442
P13861	cAMP-dependent protein kinase type II-alpha regulatory subunit OS=Homo sapiens OX=9606 GN=PRKAR2A PE=1 SV=2	-3.932015778	0.010410696
O14558	Heat shock protein beta-6 OS=Homo sapiens OX=9606 GN=HSPB6 PE=1 SV=2	-4.122003566	0.010446112
Q13011	Delta(3,5)-Delta(2,4)-dienoyl-CoA isomerase, mitochondrial OS=Homo sapiens OX=9606 GN=ECH1 PE=1 SV=2	-2.413082222	0.010559103

P07942	Laminin subunit beta-1 OS=Homo sapiens OX=9606 GN=LAMB1 PE=1 SV=2	2.407953629	0.010619266
Q6QEF8-3	Isoform 3 of Coronin-6 OS=Homo sapiens OX=9606 GN=CORO6	4.116954737	0.010701312
P26641-2	Isoform 2 of Elongation factor 1-gamma OS=Homo sapiens OX=9606 GN=EEF1G	-3.81741203	0.010973151
Q13162	Peroxiredoxin-4 OS=Homo sapiens OX=9606 GN=PRDX4 PE=1 SV=1	3.253859555	0.011022249
P07900-2	Isoform 2 of Heat shock protein HSP 90-alpha OS=Homo sapiens OX=9606 GN=HSP90AA1	-4.495862653	0.011566283
P01717	Immunoglobulin lambda variable 3-25 OS=Homo sapiens OX=9606 GN=IGLV3-25 PE=1 SV=2	4.273223991	0.01163372
Q9UKS6	Protein kinase C and casein kinase substrate in neurons protein 3 OS=Homo sapiens OX=9606 GN=PACSIN3 PE=1 SV=2	-2.801530227	0.011788864
Q96Q06-2	Isoform 2 of Perilipin-4 OS=Homo sapiens OX=9606 GN=PLIN4	-2.777577803	0.011856604
P04733	Metallothionein-1F OS=Homo sapiens OX=9606 GN=MT1F PE=1 SV=1	7.983145502	0.011862632
P22352	Glutathione peroxidase 3 OS=Homo sapiens OX=9606 GN=GPX3 PE=1 SV=2	3.571439748	0.011870417
P14649	Myosin light chain 6B OS=Homo sapiens OX=9606 GN=MYL6B PE=1 SV=1	-4.273551663	0.011872786
P48163	NADP-dependent malic enzyme OS=Homo sapiens OX=9606 GN=ME1 PE=1 SV=1	-2.190112577	0.011910934
P02461	Collagen alpha-1(III) chain OS=Homo sapiens OX=9606 GN=COL3A1 PE=1 SV=4	3.929766804	0.012426207
Q16658	Fascin OS=Homo sapiens OX=9606 GN=FSCN1 PE=1 SV=3	3.204875312	0.012582807
P35270	Sepiapterin reductase OS=Homo sapiens OX=9606 GN=SPR PE=1 SV=1	3.354792828	0.012606943
P08185	Corticosteroid-binding globulin OS=Homo sapiens OX=9606 GN=SERPINA6 PE=1 SV=1	4.78617925	0.012634761
P61224	Ras-related protein Rap-1b OS=Homo sapiens OX=9606 GN=RAP1B PE=1 SV=1	3.394295688	0.012718976
P60660	Myosin light polypeptide 6 OS=Homo sapiens OX=9606 GN=MYL6 PE=1 SV=2	2.257664419	0.012824383
P27816-4	Isoform 4 of Microtubule-associated protein 4 OS=Homo sapiens OX=9606 GN=MAP4	-4.335257366	0.012936639
P33121	Long-chain-fatty-acid--CoA ligase 1 OS=Homo sapiens OX=9606 GN=ACSL1 PE=1 SV=1	-2.69767834	0.013256606
P17661	Desmin OS=Homo sapiens OX=9606 GN=DES PE=1 SV=3	-5.861270101	0.01329379
Q04760	Lactoylglutathione lyase OS=Homo sapiens OX=9606 GN=GLO1 PE=1 SV=4	-3.307348423	0.013313217
P24666	Low molecular weight phosphotyrosine protein phosphatase OS=Homo sapiens OX=9606 GN=ACP1 PE=1 SV=3	-3.679181838	0.013330276
Q9NX63	MICOS complex subunit MIC19 OS=Homo sapiens OX=9606 GN=CHCHD3 PE=1 SV=1	-3.20482747	0.013381511

P00751	Complement factor B OS=Homo sapiens OX=9606 GN=CFB PE=1 SV=2	-4.176880829	0.0135607
O14732	Inositol monophosphatase 2 OS=Homo sapiens OX=9606 GN=IMPA2 PE=1 SV=1	2.220155903	0.013618614
Q02218	2-oxoglutarate dehydrogenase, mitochondrial OS=Homo sapiens OX=9606 GN=OGDH PE=1 SV=3	-5.597234223	0.013740016
P54646	5'-AMP-activated protein kinase catalytic subunit alpha-2 OS=Homo sapiens OX=9606 GN=PRKAA2 PE=1 SV=2	-1.490439166	0.014159646
P31943	Heterogeneous nuclear ribonucleoprotein H OS=Homo sapiens OX=9606 GN=HNRNPH1 PE=1 SV=4	3.886339569	0.014188031
P42126	Enoyl-CoA delta isomerase 1, mitochondrial OS=Homo sapiens OX=9606 GN=ECI1 PE=1 SV=1	3.450422024	0.014498217
P00558	Phosphoglycerate kinase 1 OS=Homo sapiens OX=9606 GN=PGK1 PE=1 SV=3	-2.218911136	0.014661627
P29536	Leiomodin-1 OS=Homo sapiens OX=9606 GN=LMOD1 PE=1 SV=3	6.319402266	0.015290028
Q8IYS1	Peptidase M20 domain-containing protein 2 OS=Homo sapiens OX=9606 GN=PM20D2 PE=1 SV=2	5.854152688	0.015318756
P36955	Pigment epithelium-derived factor OS=Homo sapiens OX=9606 GN=SERPINF1 PE=1 SV=4	1.261454971	0.015433226
Q01469	Fatty acid-binding protein 5 OS=Homo sapiens OX=9606 GN=FABP5 PE=1 SV=3	-3.658577958	0.015870069
Q9NWV4	CXXC motif containing zinc binding protein OS=Homo sapiens OX=9606 GN=CZIB PE=1 SV=1	-1.626034299	0.015878212
P51687	Sulfite oxidase, mitochondrial OS=Homo sapiens OX=9606 GN=SUOX PE=1 SV=2	3.43703684	0.0159299
P22830-2	Isoform 2 of Ferrochelatase, mitochondrial OS=Homo sapiens OX=9606 GN=FECH	3.665919329	0.016173255
P02452	Collagen alpha-1(I) chain OS=Homo sapiens OX=9606 GN=COL1A1 PE=1 SV=5	2.727318398	0.016177642
Q9BYS8	Leucine-rich repeat-containing protein 2 OS=Homo sapiens OX=9606 GN=LRRC2 PE=2 SV=2	3.312185664	0.016671489
O75083	WD repeat-containing protein 1 OS=Homo sapiens OX=9606 GN=WDR1 PE=1 SV=4	-3.589649243	0.016983587
Q07955-2	Isoform ASF-2 of Serine/arginine-rich splicing factor 1 OS=Homo sapiens OX=9606 GN=SRSF1	3.493810905	0.016984998
Q9H8H3	Methyltransferase-like protein 7A OS=Homo sapiens OX=9606 GN=METTL7A PE=1 SV=1	-2.655881213	0.017045154
Q0VAK6	Leiomodin-3 OS=Homo sapiens OX=9606 GN=LMOD3 PE=1 SV=1	-3.720225319	0.017050006
Q9Y263	Phospholipase A-2-activating protein OS=Homo sapiens OX=9606 GN=PLAA PE=1 SV=2	3.146012414	0.017127099
P61163	Alpha-centractin OS=Homo sapiens OX=9606 GN=ACTR1A PE=1 SV=1	3.272465885	0.018156352

Q9NSD9	Phenylalanine--tRNA ligase beta subunit OS=Homo sapiens OX=9606 GN=FARSB PE=1 SV=3	-2.597403762	0.018205054
P09382	Galectin-1 OS=Homo sapiens OX=9606 GN=LGALS1 PE=1 SV=2	-4.732730995	0.018368619
P13805-3	Isoform 3 of Troponin T, slow skeletal muscle OS=Homo sapiens OX=9606 GN=TNNT1	-5.07805974	0.018576452
Q9Y490	Talin-1 OS=Homo sapiens OX=9606 GN=TLN1 PE=1 SV=3	-4.968302738	0.01882365
P17568	NADH dehydrogenase [ubiquinone] 1 beta subcomplex subunit 7 OS=Homo sapiens OX=9606 GN=NDUFB7 PE=1 SV=4	-3.336395086	0.019333297
P00403	Cytochrome c oxidase subunit 2 OS=Homo sapiens OX=9606 GN=MT-CO2 PE=1 SV=1	-2.123573905	0.01953751
A1L0T0	Acetolactate synthase-like protein OS=Homo sapiens OX=9606 GN=ILVBL PE=1 SV=2	2.34002782	0.020195246
Q8IVN3	Musculoskeletal embryonic nuclear protein 1 OS=Homo sapiens OX=9606 GN=MUSTN1 PE=3 SV=2	5.209253937	0.020735308
P05556	Integrin beta-1 OS=Homo sapiens OX=9606 GN=ITGB1 PE=1 SV=2	1.566672222	0.020785526
P53611	Geranylgeranyl transferase type-2 subunit beta OS=Homo sapiens OX=9606 GN=RABGGTB PE=1 SV=2	2.971972757	0.020799956
Q8TD30	Alanine aminotransferase 2 OS=Homo sapiens OX=9606 GN=GPT2 PE=1 SV=1	5.856381579	0.021185332
P42330	Aldo-keto reductase family 1 member C3 OS=Homo sapiens OX=9606 GN=AKR1C3 PE=1 SV=4	2.44841971	0.021661881
Q9Y376	Calcium-binding protein 39 OS=Homo sapiens OX=9606 GN=CAB39 PE=1 SV=1	-2.837358123	0.022128794
O43674	NADH dehydrogenase [ubiquinone] 1 beta subcomplex subunit 5, mitochondrial OS=Homo sapiens OX=9606 GN=NDUFB5 PE=1 SV=1	-2.378787632	0.022189678
P11277-2	Isoform 2 of Spectrin beta chain, erythrocytic OS=Homo sapiens OX=9606 GN=SPTB	-5.173330771	0.022250033
Q53GG5	PDZ and LIM domain protein 3 OS=Homo sapiens OX=9606 GN=PDLIM3 PE=1 SV=1	-2.413621691	0.022388698
Q09028	Histone-binding protein RBBP4 OS=Homo sapiens OX=9606 GN=RBBP4 PE=1 SV=3	4.952000061	0.023268892
Q8IW45	ATP-dependent (S)-NAD(P)H-hydrate dehydratase OS=Homo sapiens OX=9606 GN=NAXD PE=1 SV=1	4.109090636	0.023404901
Q9UM00	Calcium load-activated calcium channel OS=Homo sapiens OX=9606 GN=TMCO1 PE=1 SV=2	6.308479013	0.023539385
Q9NQE9	Histidine triad nucleotide-binding protein 3 OS=Homo sapiens OX=9606 GN=HINT3 PE=1 SV=1	5.306400006	0.023600065
Q9ULV4-3	Isoform 3 of Coronin-1C OS=Homo sapiens OX=9606 GN=CORO1C	3.987789808	0.023691667
Q9Y6H1	Coiled-coil-helix-coiled-coil-helix domain-containing protein 2 OS=Homo sapiens OX=9606 GN=CHCHD2 PE=1 SV=1	-3.393749231	0.023826135

Q9NUB1	Acetyl-coenzyme A synthetase 2-like, mitochondrial OS=Homo sapiens OX=9606 GN=ACSS1 PE=1 SV=2	3.684536868	0.023925684
Q04837	Single-stranded DNA-binding protein, mitochondrial OS=Homo sapiens OX=9606 GN=SSBP1 PE=1 SV=1	-3.172023705	0.024006494
P45880-1	Isoform 1 of Voltage-dependent anion-selective channel protein 2 OS=Homo sapiens OX=9606 GN=VDAC2	-2.186294113	0.024164193
O75880	Protein SCO1 homolog, mitochondrial OS=Homo sapiens OX=9606 GN=SCO1 PE=1 SV=1	8.359734758	0.024173451
Q9NQC3	Reticulon-4 OS=Homo sapiens OX=9606 GN=RTN4 PE=1 SV=2	4.128198107	0.024307208
P31040	Succinate dehydrogenase [ubiquinone] flavoprotein subunit, mitochondrial OS=Homo sapiens OX=9606 GN=SDHA PE=1 SV=2	2.283749419	0.024595536
P01011	Alpha-1-antichymotrypsin OS=Homo sapiens OX=9606 GN=SERPINA3 PE=1 SV=2	1.148452085	0.024689812
Q9HDC5	Junctophilin-1 OS=Homo sapiens OX=9606 GN=JPH1 PE=1 SV=2	-3.286919091	0.024901831
P18124	60S ribosomal protein L7 OS=Homo sapiens OX=9606 GN=RPL7 PE=1 SV=1	-2.690219908	0.024974973
Q9BS92	Protein NipSnap homolog 3B OS=Homo sapiens OX=9606 GN=NIPSNAP3B PE=2 SV=1	3.98462883	0.025021702
O95747	Serine/threonine-protein kinase OSR1 OS=Homo sapiens OX=9606 GN=OXSR1 PE=1 SV=1	2.204578176	0.025270403
Q15365	Poly(rC)-binding protein 1 OS=Homo sapiens OX=9606 GN=PCBP1 PE=1 SV=2	-3.125068529	0.025534243
P07237	Protein disulfide-isomerase OS=Homo sapiens OX=9606 GN=P4HB PE=1 SV=3	1.484278766	0.0259604
P07437	Tubulin beta chain OS=Homo sapiens OX=9606 GN=TUBB PE=1 SV=2	-3.024714299	0.02644811
P46109	Crk-like protein OS=Homo sapiens OX=9606 GN=CRKL PE=1 SV=1	4.744385805	0.026742693
Q8NE62	Choline dehydrogenase, mitochondrial OS=Homo sapiens OX=9606 GN=CHDH PE=1 SV=2	2.73734059	0.026878264
O75955	Flotillin-1 OS=Homo sapiens OX=9606 GN=FLOT1 PE=1 SV=3	2.727424003	0.026936134
Q9P0L0	Vesicle-associated membrane protein-associated protein A OS=Homo sapiens OX=9606 GN=VAPA PE=1 SV=3	-3.549088035	0.027671305
P62191	26S proteasome regulatory subunit 4 OS=Homo sapiens OX=9606 GN=PSMC1 PE=1 SV=1	-3.143024652	0.027772107
Q9UI09	NADH dehydrogenase [ubiquinone] 1 alpha subcomplex subunit 12 OS=Homo sapiens OX=9606 GN=NDUFA12 PE=1 SV=1	-2.182421382	0.027876637
P62195	26S proteasome regulatory subunit 8 OS=Homo sapiens OX=9606 GN=PSMC5 PE=1 SV=1	-3.235176434	0.028203164

Q9BU02	Thiamine-triphosphatase OS=Homo sapiens OX=9606 GN=THTPA PE=1 SV=3	2.598933543	0.028663501
P02511	Alpha-crystallin B chain OS=Homo sapiens OX=9606 GN=CRYAB PE=1 SV=2	-4.209406768	0.028872505
O75155	Cullin-associated NEDD8-dissociated protein 2 OS=Homo sapiens OX=9606 GN=CAND2 PE=1 SV=3	3.424760838	0.029203504
Q96CV9	Optineurin OS=Homo sapiens OX=9606 GN=OPTN PE=1 SV=3	1.935405047	0.029449045
P23588	Eukaryotic translation initiation factor 4B OS=Homo sapiens OX=9606 GN=EIF4B PE=1 SV=2	2.113269847	0.029516953
Q2TBA0	Kelch-like protein 40 OS=Homo sapiens OX=9606 GN=KLHL40 PE=1 SV=2	-1.976215079	0.029630681
Q9NYL9	Tropomodulin-3 OS=Homo sapiens OX=9606 GN=TMOD3 PE=1 SV=1	3.486078619	0.029762556
O00442-2	Isoform 2 of RNA 3'-terminal phosphate cyclase OS=Homo sapiens OX=9606 GN=RTCA	2.044522674	0.030141771
P30049	ATP synthase subunit delta, mitochondrial OS=Homo sapiens OX=9606 GN=ATP5F1D PE=1 SV=2	3.064258505	0.030154618
P36578	60S ribosomal protein L4 OS=Homo sapiens OX=9606 GN=RPL4 PE=1 SV=5	-3.33194753	0.030330937
O43837-2	Isoform A of Isocitrate dehydrogenase [NAD] subunit beta, mitochondrial OS=Homo sapiens OX=9606 GN=IDH3B	2.092973826	0.030458784
P00325	All-trans-retinol dehydrogenase [NAD(+)] ADH1B OS=Homo sapiens OX=9606 GN=ADH1B PE=1 SV=2	1.944543484	0.030904557
Q9BYT8	Neurolysin, mitochondrial OS=Homo sapiens OX=9606 GN=NLN PE=1 SV=1	5.506635279	0.031045404
Q00169	Phosphatidylinositol transfer protein alpha isoform OS=Homo sapiens OX=9606 GN=PITPNA PE=1 SV=2	3.397226086	0.031066844
P19237	Troponin I, slow skeletal muscle OS=Homo sapiens OX=9606 GN=TNNI1 PE=1 SV=3	-4.185209675	0.031604071
P01782	Immunoglobulin heavy variable 3-9 OS=Homo sapiens OX=9606 GN=IGHV3-9 PE=1 SV=2	7.386410711	0.032315112
O43396	Thioredoxin-like protein 1 OS=Homo sapiens OX=9606 GN=TXNL1 PE=1 SV=3	3.415384511	0.032776931
P49747	Cartilage oligomeric matrix protein OS=Homo sapiens OX=9606 GN=COMP PE=1 SV=2	3.663087829	0.033193149
P0DOY2	Immunoglobulin lambda constant 2 OS=Homo sapiens OX=9606 GN=IGLC2 PE=1 SV=1	1.643891928	0.033331934
P12814-4	Isoform 4 of Alpha-actinin-1 OS=Homo sapiens OX=9606 GN=ACTN1	-3.655931894	0.033352982
Q99460	26S proteasome non-ATPase regulatory subunit 1 OS=Homo sapiens OX=9606 GN=PSMD1 PE=1 SV=2	-1.998982784	0.033570849
Q09666	Neuroblast differentiation-associated protein AHNAK OS=Homo sapiens OX=9606 GN=AHNAK PE=1 SV=2	-3.616817425	0.033575599

Q99623	Prohibitin-2 OS=Homo sapiens OX=9606 GN=PHB2 PE=1 SV=2	1.342053685	0.033633999
P23368	NAD-dependent malic enzyme, mitochondrial OS=Homo sapiens OX=9606 GN=ME2 PE=1 SV=1	1.881831134	0.03392219
P10644	cAMP-dependent protein kinase type I-alpha regulatory subunit OS=Homo sapiens OX=9606 GN=PRKAR1A PE=1 SV=1	-3.940325442	0.034849193
O75112-2	Isoform 2 of LIM domain-binding protein 3 OS=Homo sapiens OX=9606 GN=LDB3	-1.320288823	0.035398866
Q9H987	Synaptopodin 2-like protein OS=Homo sapiens OX=9606 GN=SYNPO2L PE=2 SV=3	-1.957453384	0.03581074
Q13283	Ras GTPase-activating protein-binding protein 1 OS=Homo sapiens OX=9606 GN=G3BP1 PE=1 SV=1	2.084748034	0.036176878
Q07021	Complement component 1 Q subcomponent-binding protein, mitochondrial OS=Homo sapiens OX=9606 GN=C1QBP PE=1 SV=1	-3.813022054	0.036243859
Q16543	Hsp90 co-chaperone Cdc37 OS=Homo sapiens OX=9606 GN=CDC37 PE=1 SV=1	0.990079929	0.036373384
P50213	Isocitrate dehydrogenase [NAD] subunit alpha, mitochondrial OS=Homo sapiens OX=9606 GN=IDH3A PE=1 SV=1	-3.234017584	0.036427241
P11177	Pyruvate dehydrogenase E1 component subunit beta, mitochondrial OS=Homo sapiens OX=9606 GN=PDHB PE=1 SV=3	-3.855420985	0.036996811
P47897	Glutamine--tRNA ligase OS=Homo sapiens OX=9606 GN=QARS PE=1 SV=1	8.821889398	0.037129552
Q13326	Gamma-sarcoglycan OS=Homo sapiens OX=9606 GN=SGCG PE=1 SV=4	-2.541298116	0.037815396
Q13698	Voltage-dependent L-type calcium channel subunit alpha-1S OS=Homo sapiens OX=9606 GN=CACNA1S PE=1 SV=4	-3.753105354	0.038608351
Q92804	TATA-binding protein-associated factor 2N OS=Homo sapiens OX=9606 GN=TAF15 PE=1 SV=1	2.950209861	0.03863616
Q08043	Alpha-actinin-3 OS=Homo sapiens OX=9606 GN=ACTN3 PE=1 SV=2	-5.011989457	0.038654787
P01591	Immunoglobulin J chain OS=Homo sapiens OX=9606 GN=JCHAIN PE=1 SV=4	3.805507618	0.038974303
P30084	Enoyl-CoA hydratase, mitochondrial OS=Homo sapiens OX=9606 GN=ECHS1 PE=1 SV=4	-3.914774054	0.038976398
P17612	cAMP-dependent protein kinase catalytic subunit alpha OS=Homo sapiens OX=9606 GN=PRKACA PE=1 SV=2	-3.370966841	0.03971184
P01031	Complement C5 OS=Homo sapiens OX=9606 GN=C5 PE=1 SV=4	3.705957132	0.040222013
P20774	Mimecan OS=Homo sapiens OX=9606 GN=OGN PE=1 SV=1	-3.369070577	0.040532896
P54652	Heat shock-related 70 kDa protein 2 OS=Homo sapiens OX=9606 GN=HSPA2 PE=1 SV=1	-5.108553776	0.040720281

P61353	60S ribosomal protein L27 OS=Homo sapiens OX=9606 GN=RPL27 PE=1 SV=2	-3.123297261	0.041015054
P46977	Dolichyl-diphosphooligosaccharide--protein glycosyltransferase subunit STT3A OS=Homo sapiens OX=9606 GN=STT3A PE=1 SV=2	2.664263803	0.041113105
P51114	Fragile X mental retardation syndrome-related protein 1 OS=Homo sapiens OX=9606 GN=FXR1 PE=1 SV=3	4.401186176	0.041181293
P43304	Glycerol-3-phosphate dehydrogenase, mitochondrial OS=Homo sapiens OX=9606 GN=GPD2 PE=1 SV=3	-2.840859695	0.041738963
Q86WU2	Probable D-lactate dehydrogenase, mitochondrial OS=Homo sapiens OX=9606 GN=LDHD PE=1 SV=1	3.569514722	0.041985103
P04004	Vitronectin OS=Homo sapiens OX=9606 GN=VTN PE=1 SV=1	1.662300081	0.042006864
P05026	Sodium/potassium-transporting ATPase subunit beta-1 OS=Homo sapiens OX=9606 GN=ATP1B1 PE=1 SV=1	-2.551458018	0.042049667
P09211	Glutathione S-transferase P OS=Homo sapiens OX=9606 GN=GSTP1 PE=1 SV=2	-3.430466953	0.042543931
P26447	Protein S100-A4 OS=Homo sapiens OX=9606 GN=S100A4 PE=1 SV=1	-3.018208073	0.043159037
O43504	Ragulator complex protein LAMTOR5 OS=Homo sapiens OX=9606 GN=LAMTOR5 PE=1 SV=1	2.421914205	0.043202516
P62266	40S ribosomal protein S23 OS=Homo sapiens OX=9606 GN=RPS23 PE=1 SV=3	2.916338794	0.043395152
P05204	Non-histone chromosomal protein HMG-17 OS=Homo sapiens OX=9606 GN=HMGN2 PE=1 SV=3	5.618714972	0.043656339
B9A064	Immunoglobulin lambda-like polypeptide 5 OS=Homo sapiens OX=9606 GN=IGLL5 PE=2 SV=2	3.362085641	0.043715376
P11940	Polyadenylate-binding protein 1 OS=Homo sapiens OX=9606 GN=PABPC1 PE=1 SV=2	2.711644291	0.045304134
P02747	Complement C1q subcomponent subunit C OS=Homo sapiens OX=9606 GN=C1QC PE=1 SV=3	2.981557142	0.04547811
P12882	Myosin-1 OS=Homo sapiens OX=9606 GN=MYH1 PE=1 SV=3	-4.475445291	0.04555219
Q9NP72	Ras-related protein Rab-18 OS=Homo sapiens OX=9606 GN=RAB18 PE=1 SV=1	3.250923467	0.045685302
Q9BXV9	EKC/KEOPS complex subunit GON7 OS=Homo sapiens OX=9606 GN=GON7 PE=1 SV=2	2.309924281	0.046101172
Q92900	Regulator of nonsense transcripts 1 OS=Homo sapiens OX=9606 GN=UPF1 PE=1 SV=2	2.605403185	0.046162063
P07384	Calpain-1 catalytic subunit OS=Homo sapiens OX=9606 GN=CAPN1 PE=1 SV=1	-2.277856817	0.046552155
Q92499	ATP-dependent RNA helicase DDX1 OS=Homo sapiens OX=9606 GN=DDX1 PE=1 SV=2	1.726786953	0.046820251

Q8IWA5-2	Isoform 2 of Choline transporter-like protein 2 OS=Homo sapiens OX=9606 GN=SLC44A2	2.921766041	0.046986783
P23526	Adenosylhomocysteinase OS=Homo sapiens OX=9606 GN=AHCY PE=1 SV=4	-3.819122126	0.046996907
Q9H3H3-3	Isoform 3 of UPF0696 protein C11orf68 OS=Homo sapiens OX=9606 GN=C11orf68	2.573413288	0.047370645
P02652	Apolipoprotein A-II OS=Homo sapiens OX=9606 GN=APOA2 PE=1 SV=1	-4.356653299	0.04922249
P23396-2	Isoform 2 of 40S ribosomal protein S3 OS=Homo sapiens OX=9606 GN=RPS3	-3.225238305	0.049840588
Q5BKX8	Caveolae-associated protein 4 OS=Homo sapiens OX=9606 GN=CAVIN4 PE=1 SV=2	-3.531866058	0.050048855
Q9HD42	Charged multivesicular body protein 1a OS=Homo sapiens OX=9606 GN=CHMP1A PE=1 SV=1	3.307975484	0.050598121
Q15772	Striated muscle preferentially expressed protein kinase OS=Homo sapiens OX=9606 GN=SPEG PE=1 SV=4	-1.10812864	0.051904835
P61970	Nuclear transport factor 2 OS=Homo sapiens OX=9606 GN=NUTF2 PE=1 SV=1	-3.066088008	0.052817389
Q9H7C9	Mth938 domain-containing protein OS=Homo sapiens OX=9606 GN=AAMDC PE=1 SV=1	-1.436514397	0.053144792
P29692-2	Isoform 2 of Elongation factor 1-delta OS=Homo sapiens OX=9606 GN=EEF1D	3.179609422	0.054024196
P00505	Aspartate aminotransferase, mitochondrial OS=Homo sapiens OX=9606 GN=GOT2 PE=1 SV=3	-3.733411217	0.054206175
P17655	Calpain-2 catalytic subunit OS=Homo sapiens OX=9606 GN=CAPN2 PE=1 SV=6	-2.737165729	0.054463498
O60256	Phosphoribosyl pyrophosphate synthase-associated protein 2 OS=Homo sapiens OX=9606 GN=PRPSAP2 PE=1 SV=1	1.892951129	0.054710158
P22314	Ubiquitin-like modifier-activating enzyme 1 OS=Homo sapiens OX=9606 GN=UBA1 PE=1 SV=3	-3.611208953	0.055008718
P36776	Lon protease homolog, mitochondrial OS=Homo sapiens OX=9606 GN=LONP1 PE=1 SV=2	2.720842341	0.055834503
Q9BWH2	FUN14 domain-containing protein 2 OS=Homo sapiens OX=9606 GN=FUNDC2 PE=1 SV=2	-2.705940374	0.056274405
P25787	Proteasome subunit alpha type-2 OS=Homo sapiens OX=9606 GN=PSMA2 PE=1 SV=2	-3.351317333	0.056928993
P01871	Immunoglobulin heavy constant mu OS=Homo sapiens OX=9606 GN=IGHM PE=1 SV=4	-3.616846333	0.057422743
P08758	Annexin A5 OS=Homo sapiens OX=9606 GN=ANXA5 PE=1 SV=2	-3.779299404	0.057459381
Q9NRG7	Epimerase family protein SDR39U1 OS=Homo sapiens OX=9606 GN=SDR39U1 PE=1 SV=3	2.772078593	0.057510955
Q9BRA2	Thioredoxin domain-containing protein 17 OS=Homo sapiens OX=9606 GN=TXNDC17 PE=1 SV=1	2.80744483	0.057730492

P22105	Tenascin-X OS=Homo sapiens OX=9606 GN=TNXB PE=1 SV=5	-2.750204486	0.058099888
Q96EY7	Pentatricopeptide repeat domain-containing protein 3, mitochondrial OS=Homo sapiens OX=9606 GN=PTCD3 PE=1 SV=3	1.822160367	0.058525752
P02790	Hemopexin OS=Homo sapiens OX=9606 GN=HPX PE=1 SV=2	-2.51931442	0.059240096
Q96S99	Pleckstrin homology domain-containing family F member 1 OS=Homo sapiens OX=9606 GN=PLEKHF1 PE=1 SV=3	2.178453024	0.059266122
P09493-4	Isoform 4 of Tropomyosin alpha-1 chain OS=Homo sapiens OX=9606 GN=TPM1	-4.700005963	0.05937517
Q9NZZ3	Charged multivesicular body protein 5 OS=Homo sapiens OX=9606 GN=CHMP5 PE=1 SV=1	4.101863556	0.059992786
Q02878	60S ribosomal protein L6 OS=Homo sapiens OX=9606 GN=RPL6 PE=1 SV=3	-3.934631672	0.0604735
P06312	Immunoglobulin kappa variable 4-1 OS=Homo sapiens OX=9606 GN=IGKV4-1 PE=1 SV=1	5.4394018	0.061263297
P63167	Dynein light chain 1, cytoplasmic OS=Homo sapiens OX=9606 GN=DYNLL1 PE=1 SV=1	6.011571233	0.062107295
P05387	60S acidic ribosomal protein P2 OS=Homo sapiens OX=9606 GN=RPLP2 PE=1 SV=1	-3.576874487	0.062419395
P09486	SPARC OS=Homo sapiens OX=9606 GN=SPARC PE=1 SV=1	2.507277938	0.062599407
P16452-2	Isoform Long of Erythrocyte membrane protein band 4.2 OS=Homo sapiens OX=9606 GN=EPB42	4.236843552	0.06292295
P02750	Leucine-rich alpha-2-glycoprotein OS=Homo sapiens OX=9606 GN=LRG1 PE=1 SV=2	1.542117837	0.06370931
Q9Y6M9	NADH dehydrogenase [ubiquinone] 1 beta subcomplex subunit 9 OS=Homo sapiens OX=9606 GN=NDUFB9 PE=1 SV=3	-2.293338165	0.063967992
Q96I99	Succinate--CoA ligase [GDP-forming] subunit beta, mitochondrial OS=Homo sapiens OX=9606 GN=SUCLG2 PE=1 SV=2	-3.641430491	0.063983294
P32119	Peroxiredoxin-2 OS=Homo sapiens OX=9606 GN=PRDX2 PE=1 SV=5	-1.550221281	0.064452238
P23246	Splicing factor, proline- and glutamine-rich OS=Homo sapiens OX=9606 GN=SFPQ PE=1 SV=2	2.660684593	0.064600171
Q3ZCQ8-2	Isoform 2 of Mitochondrial import inner membrane translocase subunit TIM50 OS=Homo sapiens OX=9606 GN=TIMM50	2.501542473	0.06463338
Q8TAE6	Protein phosphatase 1 regulatory subunit 14C OS=Homo sapiens OX=9606 GN=PPP1R14C PE=1 SV=3	5.81927113	0.065160048
Q8WZ42-9	Isoform 9 of Titin OS=Homo sapiens OX=9606 GN=TTN	-3.840929053	0.065514447
P02656	Apolipoprotein C-III OS=Homo sapiens OX=9606 GN=APOC3 PE=1 SV=1	1.967009501	0.065902567
P01008	Antithrombin-III OS=Homo sapiens OX=9606 GN=SERPINC1 PE=1 SV=1	2.210706665	0.06635325

P13693	Translationally-controlled tumor protein OS=Homo sapiens OX=9606 GN=TPT1 PE=1 SV=1	-0.703060176	0.067132126
Q9Y305	Acyl-coenzyme A thioesterase 9, mitochondrial OS=Homo sapiens OX=9606 GN=ACOT9 PE=1 SV=2	-1.16949047	0.067916093
O00410-3	Isoform 3 of Importin-5 OS=Homo sapiens OX=9606 GN=IPO5	-1.450599134	0.069266693
Q01844-5	Isoform 5 of RNA-binding protein EWS OS=Homo sapiens OX=9606 GN=EWSR1	3.243183581	0.069474119
P36871-2	Isoform 2 of Phosphoglucomutase-1 OS=Homo sapiens OX=9606 GN=PGM1	3.766547331	0.069758506
Q86UP2	Kinectin OS=Homo sapiens OX=9606 GN=KTN1 PE=1 SV=1	4.944558605	0.069880166
Q14558-2	Isoform 2 of Phosphoribosyl pyrophosphate synthase-associated protein 1 OS=Homo sapiens OX=9606 GN=PRPSAP1	1.707412869	0.070254665
P51970	NADH dehydrogenase [ubiquinone] 1 alpha subcomplex subunit 8 OS=Homo sapiens OX=9606 GN=NDUFA8 PE=1 SV=3	-2.448234654	0.070335174
P35908	Keratin, type II cytoskeletal 2 epidermal OS=Homo sapiens OX=9606 GN=KRT2 PE=1 SV=2	-3.546722571	0.070745664
O15212	Prefoldin subunit 6 OS=Homo sapiens OX=9606 GN=PFDN6 PE=1 SV=1	-2.623409566	0.070814253
P48637	Glutathione synthetase OS=Homo sapiens OX=9606 GN=GSS PE=1 SV=1	5.255705092	0.070860389
O00748-4	Isoform 3 of Cocaine esterase OS=Homo sapiens OX=9606 GN=CES2	4.393534919	0.070959425
P37837	Transaldolase OS=Homo sapiens OX=9606 GN=TALDO1 PE=1 SV=2	-3.327156105	0.07120343
P22033	Methylmalonyl-CoA mutase, mitochondrial OS=Homo sapiens OX=9606 GN=MMUT PE=1 SV=4	3.148802734	0.071395009
Q15124	Phosphoglucomutase-like protein 5 OS=Homo sapiens OX=9606 GN=PGM5 PE=1 SV=2	-1.25982319	0.072604975
O95202	Mitochondrial proton/calcium exchanger protein OS=Homo sapiens OX=9606 GN=LETM1 PE=1 SV=1	3.011671079	0.07469828
P55072	Transitional endoplasmic reticulum ATPase OS=Homo sapiens OX=9606 GN=VCP PE=1 SV=4	-4.334042649	0.074925122
Q9BX66	Sorbin and SH3 domain-containing protein 1 OS=Homo sapiens OX=9606 GN=SORBS1 PE=1 SV=3	-2.683971784	0.074982717
Q9NSE4	Isoleucine--tRNA ligase, mitochondrial OS=Homo sapiens OX=9606 GN=IARS2 PE=1 SV=2	0.882093883	0.075539214
Q9NNW7	Thioredoxin reductase 2, mitochondrial OS=Homo sapiens OX=9606 GN=TXNRD2 PE=1 SV=3	3.431786278	0.076956105
Q9NZQ9	Tropomodulin-4 OS=Homo sapiens OX=9606 GN=TMOD4 PE=2 SV=1	2.37508852	0.077461011

Q8NC51	Plasminogen activator inhibitor 1 RNA-binding protein OS=Homo sapiens OX=9606 GN=SERBP1 PE=1 SV=2	-2.315966357	0.078963496
Q9Y230	RuvB-like 2 OS=Homo sapiens OX=9606 GN=RUVBL2 PE=1 SV=3	4.719400784	0.079235803
P04080	Cystatin-B OS=Homo sapiens OX=9606 GN=CSTB PE=1 SV=2	-2.568951808	0.079934443
Q9P2J5	Leucine--tRNA ligase, cytoplasmic OS=Homo sapiens OX=9606 GN=LARS PE=1 SV=2	2.40049336	0.08048769
Q16795	NADH dehydrogenase [ubiquinone] 1 alpha subcomplex subunit 9, mitochondrial OS=Homo sapiens OX=9606 GN=NDUFA9 PE=1 SV=2	-2.566512407	0.081212089
P20807	Calpain-3 OS=Homo sapiens OX=9606 GN=CAPN3 PE=1 SV=2	-1.263957745	0.081985945
P43155	Carnitine O-acetyltransferase OS=Homo sapiens OX=9606 GN=CRAT PE=1 SV=5	-1.418218044	0.082459782
Q9BXI3	Cytosolic 5'-nucleotidase 1A OS=Homo sapiens OX=9606 GN=NT5C1A PE=1 SV=1	1.562413865	0.082691544
Q16186	Proteasomal ubiquitin receptor ADRM1 OS=Homo sapiens OX=9606 GN=ADRM1 PE=1 SV=2	4.370567464	0.082705351
P10606	Cytochrome c oxidase subunit 5B, mitochondrial OS=Homo sapiens OX=9606 GN=COX5B PE=1 SV=2	-5.219051613	0.083155252
O00217	NADH dehydrogenase [ubiquinone] iron-sulfur protein 8, mitochondrial OS=Homo sapiens OX=9606 GN=NDUFS8 PE=1 SV=1	-1.655555294	0.083893019
P27169	Serum paraoxonase/arylesterase 1 OS=Homo sapiens OX=9606 GN=PON1 PE=1 SV=3	2.870883077	0.084201266
P01024	Complement C3 OS=Homo sapiens OX=9606 GN=C3 PE=1 SV=2	-1.654751283	0.084426067
Q9BQ69	ADP-ribose glycohydrolase MACROD1 OS=Homo sapiens OX=9606 GN=MACROD1 PE=1 SV=2	-2.935283956	0.084982518
P14678-3	Isoform SM-B1 of Small nuclear ribonucleoprotein-associated proteins B and B' OS=Homo sapiens OX=9606 GN=SNRPB	2.544159331	0.085204564
P26196	Probable ATP-dependent RNA helicase DDX6 OS=Homo sapiens OX=9606 GN=DDX6 PE=1 SV=2	4.260014856	0.085842012
P18859-2	Isoform 2 of ATP synthase-coupling factor 6, mitochondrial OS=Homo sapiens OX=9606 GN=ATP5PF	-1.634679172	0.086470963
P62906	60S ribosomal protein L10a OS=Homo sapiens OX=9606 GN=RPL10A PE=1 SV=2	-1.659451286	0.086911748
P40227	T-complex protein 1 subunit zeta OS=Homo sapiens OX=9606 GN=CCT6A PE=1 SV=3	-2.757857609	0.0892184
Q9NVS9	Pyridoxine-5'-phosphate oxidase OS=Homo sapiens OX=9606 GN=PNPO PE=1 SV=1	3.553233317	0.090869205
P61201-2	Isoform 2 of COP9 signalosome complex subunit 2 OS=Homo sapiens OX=9606 GN=COPS2	-2.623503494	0.092593651

Q00G26	Perilipin-5 OS=Homo sapiens OX=9606 GN=PLIN5 PE=1 SV=2	3.097543322	0.092741541
P27816	Microtubule-associated protein 4 OS=Homo sapiens OX=9606 GN=MAP4 PE=1 SV=3	-3.797306685	0.093938712
Q9UBQ0-2	Isoform 2 of Vacuolar protein sorting-associated protein 29 OS=Homo sapiens OX=9606 GN=VPS29	1.811345493	0.093947029
Q16539	Mitogen-activated protein kinase 14 OS=Homo sapiens OX=9606 GN=MAPK14 PE=1 SV=3	2.509804679	0.094281442
Q9NZA1	Chloride intracellular channel protein 5 OS=Homo sapiens OX=9606 GN=CLIC5 PE=1 SV=3	-2.589787544	0.094607271
Q9NZU5	LIM and cysteine-rich domains protein 1 OS=Homo sapiens OX=9606 GN=LMCD1 PE=1 SV=1	-3.33633848	0.094650731
P16671	Platelet glycoprotein 4 OS=Homo sapiens OX=9606 GN=CD36 PE=1 SV=2	-2.526192697	0.095815111
P28838	Cytosol aminopeptidase OS=Homo sapiens OX=9606 GN=LAP3 PE=1 SV=3	-2.312219419	0.096582951
Q8IWX7	Protein unc-45 homolog B OS=Homo sapiens OX=9606 GN=UNC45B PE=1 SV=1	-3.204679564	0.098661022
Q8N5G0-2	Isoform 2 of Small integral membrane protein 20 OS=Homo sapiens OX=9606 GN=SMIM20	3.050603843	0.10008294
P08621	U1 small nuclear ribonucleoprotein 70 kDa OS=Homo sapiens OX=9606 GN=SNRNP70 PE=1 SV=2	4.488795757	0.101445071
Q9NRG4	N-lysine methyltransferase SMYD2 OS=Homo sapiens OX=9606 GN=SMYD2 PE=1 SV=2	2.624305129	0.102578053
P51812	Ribosomal protein S6 kinase alpha-3 OS=Homo sapiens OX=9606 GN=RPS6KA3 PE=1 SV=1	1.688845045	0.103012013
Q9NZ45	CDGSH iron-sulfur domain-containing protein 1 OS=Homo sapiens OX=9606 GN=CISD1 PE=1 SV=1	1.925984452	0.105427166
P51884	Lumican OS=Homo sapiens OX=9606 GN=LUM PE=1 SV=2	-1.4750918	0.108257606
P51991	Heterogeneous nuclear ribonucleoprotein A3 OS=Homo sapiens OX=9606 GN=HNRNPA3 PE=1 SV=2	3.282763787	0.10877263
P28482	Mitogen-activated protein kinase 1 OS=Homo sapiens OX=9606 GN=MAPK1 PE=1 SV=3	1.710995946	0.113778854
Q9NVE7	4'-phosphopantetheine phosphatase OS=Homo sapiens OX=9606 GN=PANK4 PE=1 SV=1	1.667042506	0.114049542
P07954	Fumarate hydratase, mitochondrial OS=Homo sapiens OX=9606 GN=FH PE=1 SV=3	2.424798352	0.115494132
P30086	Phosphatidylethanolamine-binding protein 1 OS=Homo sapiens OX=9606 GN=PEBP1 PE=1 SV=3	-2.331560311	0.115642909
P25788	Proteasome subunit alpha type-3 OS=Homo sapiens OX=9606 GN=PSMA3 PE=1 SV=2	-2.702596208	0.116412302

Q9UHQ9	NADH-cytochrome b5 reductase 1 OS=Homo sapiens OX=9606 GN=CYB5R1 PE=1 SV=1	-4.671796874	0.116773537
Q13185	Chromobox protein homolog 3 OS=Homo sapiens OX=9606 GN=CBX3 PE=1 SV=4	6.235068301	0.117106042
Q9Y3D6	Mitochondrial fission 1 protein OS=Homo sapiens OX=9606 GN=FIS1 PE=1 SV=2	-3.138440508	0.117222433
P01861	Immunoglobulin heavy constant gamma 4 OS=Homo sapiens OX=9606 GN=IGHG4 PE=1 SV=1	1.725692192	0.118364467
O00303	Eukaryotic translation initiation factor 3 subunit F OS=Homo sapiens OX=9606 GN=EIF3F PE=1 SV=1	2.59762731	0.118582874
P61247	40S ribosomal protein S3a OS=Homo sapiens OX=9606 GN=RPS3A PE=1 SV=2	-2.395577173	0.118940797
O94905	Erlin-2 OS=Homo sapiens OX=9606 GN=ERLIN2 PE=1 SV=1	-2.635708909	0.121936725
P02743	Serum amyloid P-component OS=Homo sapiens OX=9606 GN=APCS PE=1 SV=2	1.738902719	0.122793384
P11586	C-1-tetrahydrofolate synthase, cytoplasmic OS=Homo sapiens OX=9606 GN=MTHFD1 PE=1 SV=3	-3.403377085	0.123313243
P61020	Ras-related protein Rab-5B OS=Homo sapiens OX=9606 GN=RAB5B PE=1 SV=1	3.424486332	0.123716229
O94875-7	Isoform 7 of Sorbin and SH3 domain-containing protein 2 OS=Homo sapiens OX=9606 GN=SORBS2	4.17819328	0.124199747
O43488	Aflatoxin B1 aldehyde reductase member 2 OS=Homo sapiens OX=9606 GN=AKR7A2 PE=1 SV=3	1.676840151	0.12475304
Q13363	C-terminal-binding protein 1 OS=Homo sapiens OX=9606 GN=CTBP1 PE=1 SV=2	4.256113747	0.125741728
P36871	Phosphoglucomutase-1 OS=Homo sapiens OX=9606 GN=PGM1 PE=1 SV=3	-4.457922606	0.126334157
P61586	Transforming protein RhoA OS=Homo sapiens OX=9606 GN=RHOA PE=1 SV=1	-2.467409497	0.128330564
P09936	Ubiquitin carboxyl-terminal hydrolase isozyme L1 OS=Homo sapiens OX=9606 GN=UCHL1 PE=1 SV=2	-2.844029794	0.130287467
Q9BRG1	Vacuolar protein-sorting-associated protein 25 OS=Homo sapiens OX=9606 GN=VPS25 PE=1 SV=1	1.988348469	0.13450593
Q01082	Spectrin beta chain, non-erythrocytic 1 OS=Homo sapiens OX=9606 GN=SPTBN1 PE=1 SV=2	-1.479739843	0.135044009
A0FGR8-6	Isoform 6 of Extended synaptotagmin-2 OS=Homo sapiens OX=9606 GN=ESYT2	2.004859193	0.13775058
P55795	Heterogeneous nuclear ribonucleoprotein H2 OS=Homo sapiens OX=9606 GN=HNRNPH2 PE=1 SV=1	2.550150766	0.140280076
Q702N8	Xin actin-binding repeat-containing protein 1 OS=Homo sapiens OX=9606 GN=XIRP1 PE=1 SV=1	-1.541318995	0.140812762
O43677	NADH dehydrogenase [ubiquinone] 1 subunit C1, mitochondrial OS=Homo sapiens OX=9606 GN=NDUFC1 PE=1 SV=1	-2.158651225	0.141304009

P46777	60S ribosomal protein L5 OS=Homo sapiens OX=9606 GN=RPL5 PE=1 SV=3	-2.554091152	0.141430685
P63241-2	Isoform 2 of Eukaryotic translation initiation factor 5A-1 OS=Homo sapiens OX=9606 GN=EIF5A	-2.850464537	0.142805229
O95197	Reticulon-3 OS=Homo sapiens OX=9606 GN=RTN3 PE=1 SV=2	3.704562499	0.14518644
P35555	Fibrillin-1 OS=Homo sapiens OX=9606 GN=FBN1 PE=1 SV=4	-2.171965158	0.146440674
P62318	Small nuclear ribonucleoprotein Sm D3 OS=Homo sapiens OX=9606 GN=SNRPD3 PE=1 SV=1	2.346349553	0.147741418
P40123	Adenylyl cyclase-associated protein 2 OS=Homo sapiens OX=9606 GN=CAP2 PE=1 SV=1	-2.537188712	0.148105713
P16989	Y-box-binding protein 3 OS=Homo sapiens OX=9606 GN=YBX3 PE=1 SV=4	1.923259824	0.14950616
Q14764	Major vault protein OS=Homo sapiens OX=9606 GN=MVP PE=1 SV=4	2.836851554	0.150108067
Q9UJZ1	Stomatin-like protein 2, mitochondrial OS=Homo sapiens OX=9606 GN=STOML2 PE=1 SV=1	2.185164924	0.150284817
P62714	Serine/threonine-protein phosphatase 2A catalytic subunit beta isoform OS=Homo sapiens OX=9606 GN=PPP2CB PE=1 SV=1	4.133814001	0.153048595
Q00765	Receptor expression-enhancing protein 5 OS=Homo sapiens OX=9606 GN=REEP5 PE=1 SV=3	-2.370438583	0.158423141
Q15293	Reticulocalbin-1 OS=Homo sapiens OX=9606 GN=RCN1 PE=1 SV=1	5.492619165	0.160173507
P49753	Acyl-coenzyme A thioesterase 2, mitochondrial OS=Homo sapiens OX=9606 GN=ACOT2 PE=1 SV=6	-1.336744327	0.160718269
Q9Y2Z9	Ubiquinone biosynthesis monooxygenase COQ6, mitochondrial OS=Homo sapiens OX=9606 GN=COQ6 PE=1 SV=2	4.846206038	0.162377991
Q9UBQ5	Eukaryotic translation initiation factor 3 subunit K OS=Homo sapiens OX=9606 GN=EIF3K PE=1 SV=1	1.691829211	0.163958447
P55268	Laminin subunit beta-2 OS=Homo sapiens OX=9606 GN=LAMB2 PE=1 SV=2	-1.905539518	0.16535893
Q9UII2	ATPase inhibitor, mitochondrial OS=Homo sapiens OX=9606 GN=ATP5IF1 PE=1 SV=1	-3.638079363	0.165623113
O43708	Maleylacetoacetate isomerase OS=Homo sapiens OX=9606 GN=GSTZ1 PE=1 SV=3	1.479341394	0.166052524
Q13630	GDP-L-fucose synthase OS=Homo sapiens OX=9606 GN=TSTA3 PE=1 SV=1	2.507321716	0.166802789
O75534-4	Isoform 4 of Cold shock domain-containing protein E1 OS=Homo sapiens OX=9606 GN=CSDE1	-1.654462762	0.16793988
P25786-2	Isoform Long of Proteasome subunit alpha type-1 OS=Homo sapiens OX=9606 GN=PSMA1	2.018161028	0.168078966

Q13200	26S proteasome non-ATPase regulatory subunit 2 OS=Homo sapiens OX=9606 GN=PSMD2 PE=1 SV=3	-2.443440955	0.169224159
A0A0C4DH38	Immunoglobulin heavy variable 5-51 OS=Homo sapiens OX=9606 GN=IGHV5-51 PE=3 SV=1	4.367771281	0.170579779
O75367	Core histone macro-H2A.1 OS=Homo sapiens OX=9606 GN=H2AFY PE=1 SV=4	1.154550814	0.173028274
O95822	Malonyl-CoA decarboxylase, mitochondrial OS=Homo sapiens OX=9606 GN=MLYCD PE=1 SV=3	2.439256714	0.173493518
P61313	60S ribosomal protein L15 OS=Homo sapiens OX=9606 GN=RPL15 PE=1 SV=2	1.44651226	0.178535343
Q86VB7-2	Isoform 2 of Scavenger receptor cysteine-rich type 1 protein M130 OS=Homo sapiens OX=9606 GN=CD163	2.897160569	0.179183689
P08670	Vimentin OS=Homo sapiens OX=9606 GN=VIM PE=1 SV=4	-2.205307246	0.1827751
P07910	Heterogeneous nuclear ribonucleoproteins C1/C2 OS=Homo sapiens OX=9606 GN=HNRNPC PE=1 SV=4	-2.06099434	0.182931379
P40261	Nicotinamide N-methyltransferase OS=Homo sapiens OX=9606 GN=NNMT PE=1 SV=1	4.425721273	0.183042663
P35579	Myosin-9 OS=Homo sapiens OX=9606 GN=MYH9 PE=1 SV=4	-3.020407508	0.186465104
Q96AC1-3	Isoform 3 of Fermitin family homolog 2 OS=Homo sapiens OX=9606 GN=FERMT2	-2.271747756	0.18749894
Q15848	Adiponectin OS=Homo sapiens OX=9606 GN=ADIPOQ PE=1 SV=1	1.719280412	0.188853236
P46734-3	Isoform 2 of Dual specificity mitogen-activated protein kinase kinase 3 OS=Homo sapiens OX=9606 GN=MAP2K3	2.746475492	0.189275121
P28289	Tropomodulin-1 OS=Homo sapiens OX=9606 GN=TMOD1 PE=1 SV=1	-3.453419716	0.189397136
P02689	Myelin P2 protein OS=Homo sapiens OX=9606 GN=PMP2 PE=1 SV=3	2.562249678	0.190916966
P62258	14-3-3 protein epsilon OS=Homo sapiens OX=9606 GN=YWHAE PE=1 SV=1	-4.095192053	0.191181166
P54619-3	Isoform 3 of 5'-AMP-activated protein kinase subunit gamma-1 OS=Homo sapiens OX=9606 GN=PRKAG1	-2.933477978	0.192884316
Q8TCD5	5'(3')-deoxyribonucleotidase, cytosolic type OS=Homo sapiens OX=9606 GN=NT5C PE=1 SV=2	2.47178043	0.19358317
P61221	ATP-binding cassette sub-family E member 1 OS=Homo sapiens OX=9606 GN=ABCE1 PE=1 SV=1	2.647754636	0.194189811
Q16586	Alpha-sarcoglycan OS=Homo sapiens OX=9606 GN=SGCA PE=1 SV=1	-2.742819553	0.194227077
P18577	Blood group Rh(CE) polypeptide OS=Homo sapiens OX=9606 GN=RHCE PE=1 SV=2	2.490538898	0.194839391
P13645	Keratin, type I cytoskeletal 10 OS=Homo sapiens OX=9606 GN=KRT10 PE=1 SV=6	-2.703330069	0.195380821

Q8NB12	Histone-lysine N-methyltransferase SMYD1 OS=Homo sapiens OX=9606 GN=SMYD1 PE=1 SV=1	-3.007527642	0.197308377
Q9Y4J8	Dystrobrevin alpha OS=Homo sapiens OX=9606 GN=DTNA PE=1 SV=2	-1.70598818	0.200724177
Q13564-4	Isoform 4 of NEDD8-activating enzyme E1 regulatory subunit OS=Homo sapiens OX=9606 GN=NAE1	3.396718352	0.202252816
P54577	Tyrosine--tRNA ligase, cytoplasmic OS=Homo sapiens OX=9606 GN=YARS PE=1 SV=4	3.877124108	0.202766923
P01602	Immunoglobulin kappa variable 1-5 OS=Homo sapiens OX=9606 GN=IGKV1-5 PE=1 SV=2	4.509821891	0.205272932
Q08380	Galectin-3-binding protein OS=Homo sapiens OX=9606 GN=LGALS3BP PE=1 SV=1	2.717160704	0.207412106
Q9Y3D2	Methionine-R-sulfoxide reductase B2, mitochondrial OS=Homo sapiens OX=9606 GN=MSRB2 PE=1 SV=2	2.1550174	0.208652897
P56556	NADH dehydrogenase [ubiquinone] 1 alpha subcomplex subunit 6 OS=Homo sapiens OX=9606 GN=NDUFA6 PE=1 SV=4	-1.854795132	0.208859885
Q15019-2	Isoform 2 of Septin-2 OS=Homo sapiens OX=9606 GN=SEPTIN2	2.728469127	0.220041072
Q9BXW7	Haloacid dehalogenase-like hydrolase domain-containing 5 OS=Homo sapiens OX=9606 GN=HDHD5 PE=1 SV=1	4.083668548	0.22031287
P08603	Complement factor H OS=Homo sapiens OX=9606 GN=CFH PE=1 SV=4	-2.777938963	0.220898635
P62249	40S ribosomal protein S16 OS=Homo sapiens OX=9606 GN=RPS16 PE=1 SV=2	1.286546181	0.222238005
P62277	40S ribosomal protein S13 OS=Homo sapiens OX=9606 GN=RPS13 PE=1 SV=2	-2.014477878	0.226263756
P29966	Myristoylated alanine-rich C-kinase substrate OS=Homo sapiens OX=9606 GN=MARCKS PE=1 SV=4	3.915696019	0.228355064
P62750	60S ribosomal protein L23a OS=Homo sapiens OX=9606 GN=RPL23A PE=1 SV=1	2.56845741	0.232374088
Q14738	Serine/threonine-protein phosphatase 2A 56 kDa regulatory subunit delta isoform OS=Homo sapiens OX=9606 GN=PPP2R5D PE=1 SV=1	2.988454254	0.234077523
Q13885	Tubulin beta-2A chain OS=Homo sapiens OX=9606 GN=TUBB2A PE=1 SV=1	3.050497031	0.239002789
B0YJ81	Very-long-chain (3R)-3-hydroxyacyl-CoA dehydratase 1 OS=Homo sapiens OX=9606 GN=HACD1 PE=1 SV=1	1.523531446	0.243163394
P19823	Inter-alpha-trypsin inhibitor heavy chain H2 OS=Homo sapiens OX=9606 GN=ITIH2 PE=1 SV=2	2.400436235	0.246620539
Q00688	Peptidyl-prolyl cis-trans isomerase FKBP3 OS=Homo sapiens OX=9606 GN=FKBP3 PE=1 SV=1	-1.764255945	0.247231344

P13807	Glycogen [starch] synthase, muscle OS=Homo sapiens OX=9606 GN=GYS1 PE=1 SV=2	2.091664625	0.249084716
P31939	Bifunctional purine biosynthesis protein PURH OS=Homo sapiens OX=9606 GN=ATIC PE=1 SV=3	1.15719146	0.249160662
P56537	Eukaryotic translation initiation factor 6 OS=Homo sapiens OX=9606 GN=EIF6 PE=1 SV=1	3.742142242	0.251046764
P01768	Immunoglobulin heavy variable 3-30 OS=Homo sapiens OX=9606 GN=IGHV3-30 PE=1 SV=2	2.628517047	0.253791277
O75306	NADH dehydrogenase [ubiquinone] iron-sulfur protein 2, mitochondrial OS=Homo sapiens OX=9606 GN=NDUFS2 PE=1 SV=2	-1.475079974	0.255864252
Q9NYU2	UDP-glucose:glycoprotein glucosyltransferase 1 OS=Homo sapiens OX=9606 GN=UGGT1 PE=1 SV=3	1.761338189	0.256189138
Q9UKX3	Myosin-13 OS=Homo sapiens OX=9606 GN=MYH13 PE=2 SV=2	3.712690139	0.258820534
P14618	Pyruvate kinase PKM OS=Homo sapiens OX=9606 GN=PKM PE=1 SV=4	-2.185300154	0.259525584
Q92523	Carnitine O-palmitoyltransferase 1, muscle isoform OS=Homo sapiens OX=9606 GN=CPT1B PE=1 SV=2	-2.145803342	0.279126761
P12268	Inosine-5'-monophosphate dehydrogenase 2 OS=Homo sapiens OX=9606 GN=IMPDH2 PE=1 SV=2	-1.65815739	0.279334646
O95433	Activator of 90 kDa heat shock protein ATPase homolog 1 OS=Homo sapiens OX=9606 GN=AHSA1 PE=1 SV=1	2.481064299	0.288554715
P20810-6	Isoform 6 of Calpastatin OS=Homo sapiens OX=9606 GN=CAST	0.993254728	0.289625596
Q9NTK5	Obg-like ATPase 1 OS=Homo sapiens OX=9606 GN=OLA1 PE=1 SV=2	-2.159809854	0.291399286
Q9Y262	Eukaryotic translation initiation factor 3 subunit L OS=Homo sapiens OX=9606 GN=EIF3L PE=1 SV=1	1.73087182	0.292534306
P82909	28S ribosomal protein S36, mitochondrial OS=Homo sapiens OX=9606 GN=MRPS36 PE=1 SV=2	2.111473209	0.292870974
Q99715	Collagen alpha-1(XII) chain OS=Homo sapiens OX=9606 GN=COL12A1 PE=1 SV=2	2.537231313	0.293133836
P04792	Heat shock protein beta-1 OS=Homo sapiens OX=9606 GN=HSPB1 PE=1 SV=2	-2.923277901	0.295018871
P62312	U6 snRNA-associated Sm-like protein LSm6 OS=Homo sapiens OX=9606 GN=LSM6 PE=1 SV=1	2.407870178	0.298916083
O00479	High mobility group nucleosome-binding domain-containing protein 4 OS=Homo sapiens OX=9606 GN=HMGN4 PE=1 SV=3	5.235944281	0.299054157
P46779-3	Isoform 3 of 60S ribosomal protein L28 OS=Homo sapiens OX=9606 GN=RPL28	-2.502467942	0.30176323
P01023	Alpha-2-macroglobulin OS=Homo sapiens OX=9606 GN=A2M PE=1 SV=3	-2.414388443	0.304205164

Q9NR50	Translation initiation factor eIF-2B subunit gamma OS=Homo sapiens OX=9606 GN=EIF2B3 PE=1 SV=1	1.859225267	0.304241943
P62913	60S ribosomal protein L11 OS=Homo sapiens OX=9606 GN=RPL11 PE=1 SV=2	-1.893762426	0.306390201
P16157-17	Isoform Mu17 of Ankyrin-1 OS=Homo sapiens OX=9606 GN=ANK1	4.067070998	0.31052882
Q96GG9	DCN1-like protein 1 OS=Homo sapiens OX=9606 GN=DCUN1D1 PE=1 SV=1	2.189032951	0.311344482
P23327	Sarcoplasmic reticulum histidine-rich calcium-binding protein OS=Homo sapiens OX=9606 GN=HRC PE=1 SV=1	3.750656221	0.312588628
Q9ULC4-3	Isoform 3 of Malignant T-cell-amplified sequence 1 OS=Homo sapiens OX=9606 GN=MCTS1	-1.361325413	0.31422805
P42704	Leucine-rich PPR motif-containing protein, mitochondrial OS=Homo sapiens OX=9606 GN=LRPPRC PE=1 SV=3	-3.938577135	0.315621718
Q15370-2	Isoform 2 of Elongin-B OS=Homo sapiens OX=9606 GN=ELOB	-0.837710179	0.317123099
P46940	Ras GTPase-activating-like protein IQGAP1 OS=Homo sapiens OX=9606 GN=IQGAP1 PE=1 SV=1	-1.925190505	0.319564753
P62857	40S ribosomal protein S28 OS=Homo sapiens OX=9606 GN=RPS28 PE=1 SV=1	3.581306585	0.320790859
P16118	6-phosphofructo-2-kinase/fructose-2,6-bisphosphatase 1 OS=Homo sapiens OX=9606 GN=PFKFB1 PE=1 SV=3	1.987667964	0.321585319
P62280	40S ribosomal protein S11 OS=Homo sapiens OX=9606 GN=RPS11 PE=1 SV=3	1.311572195	0.327502967
P30050	60S ribosomal protein L12 OS=Homo sapiens OX=9606 GN=RPL12 PE=1 SV=1	-1.124474413	0.329310535
P23786	Carnitine O-palmitoyltransferase 2, mitochondrial OS=Homo sapiens OX=9606 GN=CPT2 PE=1 SV=2	1.610299804	0.330520594
P22392-2	Isoform 3 of Nucleoside diphosphate kinase B OS=Homo sapiens OX=9606 GN=NME2	1.751765373	0.332077619
O75396	Vesicle-trafficking protein SEC22b OS=Homo sapiens OX=9606 GN=SEC22B PE=1 SV=4	2.355565628	0.332855537
Q12797-3	Isoform 3 of Aspartyl/asparaginyl beta-hydroxylase OS=Homo sapiens OX=9606 GN=ASPH	-3.725409995	0.335815973
Q96K17	Transcription factor BTF3 homolog 4 OS=Homo sapiens OX=9606 GN=BTF3L4 PE=1 SV=1	3.707849206	0.34281573
P14868	Aspartate--tRNA ligase, cytoplasmic OS=Homo sapiens OX=9606 GN=DARS PE=1 SV=2	-3.111492967	0.342840164
Q07065	Cytoskeleton-associated protein 4 OS=Homo sapiens OX=9606 GN=CKAP4 PE=1 SV=2	3.299496093	0.347234472
Q16891-2	Isoform 2 of MICOS complex subunit MIC60 OS=Homo sapiens OX=9606 GN=IMMT	-1.738276885	0.34802177
P04908	Histone H2A type 1-B/E OS=Homo sapiens OX=9606 GN=H2AC4 PE=1 SV=2	-2.338481589	0.351564547

O14561	Acyl carrier protein, mitochondrial OS=Homo sapiens OX=9606 GN=NDUFAB1 PE=1 SV=3	-2.776447946	0.351692862
Q8N1G4	Leucine-rich repeat-containing protein 47 OS=Homo sapiens OX=9606 GN=LRRC47 PE=1 SV=1	1.828684682	0.352430789
P49189-3	Isoform 3 of 4-trimethylaminobutyraldehyde dehydrogenase OS=Homo sapiens OX=9606 GN=ALDH9A1	-3.599143657	0.354267646
Q08209	Serine/threonine-protein phosphatase 2B catalytic subunit alpha isoform OS=Homo sapiens OX=9606 GN=PPP3CA PE=1 SV=1	2.507742351	0.354883784
Q9HBL0	Tensin-1 OS=Homo sapiens OX=9606 GN=TNS1 PE=1 SV=2	-1.866387751	0.35611734
O43852-3	Isoform 3 of Calumenin OS=Homo sapiens OX=9606 GN=CALU	-1.211884303	0.358681081
P16104	Histone H2AX OS=Homo sapiens OX=9606 GN=H2AFX PE=1 SV=2	4.41200599	0.358732238
O43741	5'-AMP-activated protein kinase subunit beta-2 OS=Homo sapiens OX=9606 GN=PRKAB2 PE=1 SV=1	2.010867311	0.36170195
P35998	26S proteasome regulatory subunit 7 OS=Homo sapiens OX=9606 GN=PSMC2 PE=1 SV=3	-2.051116243	0.368806261
P0DP25	Calmodulin-3 OS=Homo sapiens OX=9606 GN=CALM3 PE=1 SV=1	-1.514808535	0.369791038
Q13203	Myosin-binding protein H OS=Homo sapiens OX=9606 GN=MYBPH PE=1 SV=4	-2.167985661	0.371020499
P50579	Methionine aminopeptidase 2 OS=Homo sapiens OX=9606 GN=METAP2 PE=1 SV=1	2.105399258	0.372085242
O00330	Pyruvate dehydrogenase protein X component, mitochondrial OS=Homo sapiens OX=9606 GN=PDHX PE=1 SV=3	-1.930731334	0.372664707
P30044	Peroxiredoxin-5, mitochondrial OS=Homo sapiens OX=9606 GN=PRDX5 PE=1 SV=4	-3.915193018	0.373021454
P13797	Plastin-3 OS=Homo sapiens OX=9606 GN=PLS3 PE=1 SV=4	-2.575638196	0.377506054
P36969	Phospholipid hydroperoxide glutathione peroxidase OS=Homo sapiens OX=9606 GN=GPX4 PE=1 SV=3	-3.084884846	0.384175993
Q9BZQ8	Protein Niban 1 OS=Homo sapiens OX=9606 GN=NIBAN1 PE=1 SV=1	-2.687369736	0.389319092
P18621-3	Isoform 3 of 60S ribosomal protein L17 OS=Homo sapiens OX=9606 GN=RPL17	-2.691492242	0.39692107
P09104	Gamma-enolase OS=Homo sapiens OX=9606 GN=ENO2 PE=1 SV=3	-1.98443243	0.407354806
A6NDG6	Glycerol-3-phosphate phosphatase OS=Homo sapiens OX=9606 GN=PGP PE=1 SV=1	-1.603570512	0.418286047
Q99733-2	Isoform 2 of Nucleosome assembly protein 1-like 4 OS=Homo sapiens OX=9606 GN=NAP1L4	1.46122161	0.418725554
Q9Y4E8	Ubiquitin carboxyl-terminal hydrolase 15 OS=Homo sapiens OX=9606 GN=USP15 PE=1 SV=3	2.162263689	0.419874627

Q99729-2	Isoform 2 of Heterogeneous nuclear ribonucleoprotein A/B OS=Homo sapiens OX=9606 GN=HNRNPAB	2.512849183	0.420079645
Q9Y613	FH1/FH2 domain-containing protein 1 OS=Homo sapiens OX=9606 GN=FHOD1 PE=1 SV=3	2.16464515	0.423584811
P36542-2	Isoform Heart of ATP synthase subunit gamma, mitochondrial OS=Homo sapiens OX=9606 GN=ATP5F1C	4.314071756	0.4283333
Q5SSJ5	Heterochromatin protein 1-binding protein 3 OS=Homo sapiens OX=9606 GN=HP1BP3 PE=1 SV=1	-2.876818562	0.43283658
P26885	Peptidyl-prolyl cis-trans isomerase FKBP2 OS=Homo sapiens OX=9606 GN=FKBP2 PE=1 SV=2	1.7533059	0.438297386
P84085	ADP-ribosylation factor 5 OS=Homo sapiens OX=9606 GN=ARF5 PE=1 SV=2	2.811805641	0.453366705
Q14195-2	Isoform LCRMP-4 of Dihydropyrimidinase-related protein 3 OS=Homo sapiens OX=9606 GN=DPYSL3	-2.218639982	0.459158785
Q13555-6	Isoform 6 of Calcium/calmodulin-dependent protein kinase type II subunit gamma OS=Homo sapiens OX=9606 GN=CAMK2G	-2.001435038	0.463843929
P08865	40S ribosomal protein SA OS=Homo sapiens OX=9606 GN=RPSA PE=1 SV=4	-1.812216139	0.465017243
P60866-2	Isoform 2 of 40S ribosomal protein S20 OS=Homo sapiens OX=9606 GN=RPS20	3.344034104	0.46844913
O00151	PDZ and LIM domain protein 1 OS=Homo sapiens OX=9606 GN=PDLIM1 PE=1 SV=4	-3.230338835	0.472780191
P30711	Glutathione S-transferase theta-1 OS=Homo sapiens OX=9606 GN=GSTT1 PE=1 SV=4	2.030722929	0.472835472
P06727	Apolipoprotein A-IV OS=Homo sapiens OX=9606 GN=APOA4 PE=1 SV=3	-3.116348111	0.473661461
P05114	Non-histone chromosomal protein HMG-14 OS=Homo sapiens OX=9606 GN=HMGN1 PE=1 SV=3	4.565393433	0.481123268
Q04637-9	Isoform 9 of Eukaryotic translation initiation factor 4 gamma 1 OS=Homo sapiens OX=9606 GN=EIF4G1	-2.869561431	0.48829545
P48681	Nestin OS=Homo sapiens OX=9606 GN=NES PE=1 SV=2	-1.415288927	0.488438614
Q13418	Integrin-linked protein kinase OS=Homo sapiens OX=9606 GN=ILK PE=1 SV=2	-1.91081035	0.488928111
P00846	ATP synthase subunit a OS=Homo sapiens OX=9606 GN=MT-ATP6 PE=1 SV=1	4.492228556	0.494328957
P05166-2	Isoform 2 of Propionyl-CoA carboxylase beta chain, mitochondrial OS=Homo sapiens OX=9606 GN=PCCB	2.059784772	0.494607796
P39019	40S ribosomal protein S19 OS=Homo sapiens OX=9606 GN=RPS19 PE=1 SV=2	-2.417584477	0.503812116
P04083	Annexin A1 OS=Homo sapiens OX=9606 GN=ANXA1 PE=1 SV=2	-2.182801149	0.514670992
Q9GZS3	WD repeat-containing protein 61 OS=Homo sapiens OX=9606 GN=WDR61 PE=1 SV=1	2.644343859	0.526693369

P55083-2	Isoform 2 of Microfibril-associated glycoprotein 4 OS=Homo sapiens OX=9606 GN=MFAP4	4.537286617	0.52732123
O00499-2	Isoform IIB of Myc box-dependent-interacting protein 1 OS=Homo sapiens OX=9606 GN=BIN1	-2.470287227	0.530770502
P41222	Prostaglandin-H2 D-isomerase OS=Homo sapiens OX=9606 GN=PTGDS PE=1 SV=1	2.298615722	0.535080087
Q5VXT5	Synaptophysin-like protein 2 OS=Homo sapiens OX=9606 GN=SYPL2 PE=2 SV=1	-0.805452641	0.536284816
O75369-8	Isoform 8 of Filamin-B OS=Homo sapiens OX=9606 GN=FLNB	1.779084816	0.537458655
P61088	Ubiquitin-conjugating enzyme E2 N OS=Homo sapiens OX=9606 GN=UBE2N PE=1 SV=1	-1.699993333	0.539455807
P51116	Fragile X mental retardation syndrome-related protein 2 OS=Homo sapiens OX=9606 GN=FXR2 PE=1 SV=2	1.689668642	0.543273627
P26640	Valine--tRNA ligase OS=Homo sapiens OX=9606 GN=VARS PE=1 SV=4	1.613603591	0.551666816
P62269	40S ribosomal protein S18 OS=Homo sapiens OX=9606 GN=RPS18 PE=1 SV=3	-1.788595971	0.551963486
Q9NZN4	EH domain-containing protein 2 OS=Homo sapiens OX=9606 GN=EHD2 PE=1 SV=2	-2.930108952	0.55366561
O95817	BAG family molecular chaperone regulator 3 OS=Homo sapiens OX=9606 GN=BAG3 PE=1 SV=3	-1.912202531	0.556311836
Q8NDH3	Probable aminopeptidase NPEPL1 OS=Homo sapiens OX=9606 GN=NPEPL1 PE=1 SV=3	2.173651025	0.562075951
Q7L5N1	COP9 signalosome complex subunit 6 OS=Homo sapiens OX=9606 GN=COPS6 PE=1 SV=1	-1.985896304	0.571957364
P49755	Transmembrane emp24 domain-containing protein 10 OS=Homo sapiens OX=9606 GN=TMED10 PE=1 SV=2	-2.273200768	0.572204133
P55854-2	Isoform 2 of Small ubiquitin-related modifier 3 OS=Homo sapiens OX=9606 GN=SUMO3	1.031634434	0.576986796
Q9BV79	Enoyl-[acyl-carrier-protein] reductase, mitochondrial OS=Homo sapiens OX=9606 GN=MECR PE=1 SV=2	1.511913042	0.582320424
P29144	Tripeptidyl-peptidase 2 OS=Homo sapiens OX=9606 GN=TPP2 PE=1 SV=4	2.509485852	0.586103513
Q12904-2	Isoform 2 of Aminoacyl tRNA synthase complex-interacting multifunctional protein 1 OS=Homo sapiens OX=9606 GN=AIMP1	-1.240507189	0.587079541
Q5XKP0	MICOS complex subunit MIC13 OS=Homo sapiens OX=9606 GN=MICOS13 PE=1 SV=1	-1.670815678	0.605471685
P63173	60S ribosomal protein L38 OS=Homo sapiens OX=9606 GN=RPL38 PE=1 SV=2	-1.759644658	0.611924805
Q86TC9	Myopalladin OS=Homo sapiens OX=9606 GN=MYPN PE=1 SV=2	-2.886185661	0.613502701
P11532	Dystrophin OS=Homo sapiens OX=9606 GN=DMD PE=1 SV=3	-3.593612772	0.618235875

P49721	Proteasome subunit beta type-2 OS=Homo sapiens OX=9606 GN=PSMB2 PE=1 SV=1	2.978048751	0.619765326
P35637	RNA-binding protein FUS OS=Homo sapiens OX=9606 GN=FUS PE=1 SV=1	3.373511265	0.621452908
O75223	Gamma-glutamylcyclotransferase OS=Homo sapiens OX=9606 GN=GGCT PE=1 SV=1	-2.206736837	0.621544416
Q9UNM6-2	Isoform 2 of 26S proteasome non-ATPase regulatory subunit 13 OS=Homo sapiens OX=9606 GN=PSMD13	2.346117566	0.622232895
P00450	Ceruloplasmin OS=Homo sapiens OX=9606 GN=CP PE=1 SV=1	-2.913045227	0.623730896
P07741	Adenine phosphoribosyltransferase OS=Homo sapiens OX=9606 GN=APRT PE=1 SV=2	3.761478715	0.627716226
Q7L1Q6-3	Isoform 3 of Basic leucine zipper and W2 domain-containing protein 1 OS=Homo sapiens OX=9606 GN=BZW1	1.83048867	0.631265575
O94979-8	Isoform 8 of Protein transport protein Sec31A OS=Homo sapiens OX=9606 GN=SEC31A	1.55489598	0.632335928
Q96AN5	Transmembrane protein 143 OS=Homo sapiens OX=9606 GN=TMEM143 PE=2 SV=1	1.605147182	0.634213859
P60891	Ribose-phosphate pyrophosphokinase 1 OS=Homo sapiens OX=9606 GN=PRPS1 PE=1 SV=2	-1.307493161	0.644762788
Q9Y3F4-2	Isoform 2 of Serine-threonine kinase receptor-associated protein OS=Homo sapiens OX=9606 GN=STRAP	2.299592175	0.64560726
P0DME0	Protein SETSIP OS=Homo sapiens OX=9606 GN=SETSIP PE=1 SV=1	3.749422077	0.645876293
P62263	40S ribosomal protein S14 OS=Homo sapiens OX=9606 GN=RPS14 PE=1 SV=3	1.591912455	0.650112572
O60506-3	Isoform 3 of Heterogeneous nuclear ribonucleoprotein Q OS=Homo sapiens OX=9606 GN=SYNCRIP	-2.095976898	0.650363341
O75569	Interferon-inducible double-stranded RNA-dependent protein kinase activator A OS=Homo sapiens OX=9606 GN=PRKRA PE=1 SV=1	2.275244636	0.651795143
P20042	Eukaryotic translation initiation factor 2 subunit 2 OS=Homo sapiens OX=9606 GN=EIF2S2 PE=1 SV=2	1.34684996	0.65835859
Q7Z3D6-2	Isoform 2 of D-glutamate cyclase, mitochondrial OS=Homo sapiens OX=9606 GN=DGLUCY	2.354052235	0.662424841
O60664	Perilipin-3 OS=Homo sapiens OX=9606 GN=PLIN3 PE=1 SV=3	-1.87745242	0.663557252
Q16698	2,4-dienoyl-CoA reductase, mitochondrial OS=Homo sapiens OX=9606 GN=DECR1 PE=1 SV=1	-2.023780247	0.675653611
P26440	Isovaleryl-CoA dehydrogenase, mitochondrial OS=Homo sapiens OX=9606 GN=IVD PE=1 SV=2	1.232869488	0.680531179
Q9NR12-2	Isoform 2 of PDZ and LIM domain protein 7 OS=Homo sapiens OX=9606 GN=PDLIM7	-1.128226515	0.695186063

Q9Y4L1	Hypoxia up-regulated protein 1 OS=Homo sapiens OX=9606 GN=HYOU1 PE=1 SV=1	1.553821515	0.696392484
Q96CM8-2	Isoform 2 of Medium-chain acyl-CoA ligase ACSF2, mitochondrial OS=Homo sapiens OX=9606 GN=ACSF2	-1.424742072	0.707844531
O14980	Exportin-1 OS=Homo sapiens OX=9606 GN=XPO1 PE=1 SV=1	3.274818188	0.724377941
Q92734	Protein TFG OS=Homo sapiens OX=9606 GN=TFG PE=1 SV=2	1.802270284	0.731376877
P17987	T-complex protein 1 subunit alpha OS=Homo sapiens OX=9606 GN=TCP1 PE=1 SV=1	-1.524797139	0.73617252
Q9BZW5	Transmembrane 6 superfamily member 1 OS=Homo sapiens OX=9606 GN=TM6SF1 PE=1 SV=2	1.723130892	0.753693198
P04179	Superoxide dismutase [Mn], mitochondrial OS=Homo sapiens OX=9606 GN=SOD2 PE=1 SV=3	-2.088721479	0.758192936
P13533	Myosin-6 OS=Homo sapiens OX=9606 GN=MYH6 PE=1 SV=5	-2.06184929	0.758279833
Q8N3V7	Synaptopodin OS=Homo sapiens OX=9606 GN=SYNPO PE=1 SV=2	-1.964452356	0.807432555
P35527	Keratin, type I cytoskeletal 9 OS=Homo sapiens OX=9606 GN=KRT9 PE=1 SV=3	1.810781297	0.808448298
Q9Y394	Dehydrogenase/reductase SDR family member 7 OS=Homo sapiens OX=9606 GN=DHRS7 PE=1 SV=1	-2.694417154	0.810588955
P07355-2	Isoform 2 of Annexin A2 OS=Homo sapiens OX=9606 GN=ANXA2	-3.32997949	0.813505129
Q9Y4W6	AFG3-like protein 2 OS=Homo sapiens OX=9606 GN=AFG3L2 PE=1 SV=2	-2.538731599	0.814001392
P63244	Receptor of activated protein C kinase 1 OS=Homo sapiens OX=9606 GN=RACK1 PE=1 SV=3	-2.847346356	0.816986709
P54289	Voltage-dependent calcium channel subunit alpha-2/delta-1 OS=Homo sapiens OX=9606 GN=CACNA2D1 PE=1 SV=3	1.942594337	0.81788656
Q13442	28 kDa heat- and acid-stable phosphoprotein OS=Homo sapiens OX=9606 GN=PDAP1 PE=1 SV=1	-2.648994548	0.825829137
P48556	26S proteasome non-ATPase regulatory subunit 8 OS=Homo sapiens OX=9606 GN=PSMD8 PE=1 SV=2	1.632467464	0.834578878
Q14118	Dystroglycan OS=Homo sapiens OX=9606 GN=DAG1 PE=1 SV=2	-1.929342769	0.835492431
P60228	Eukaryotic translation initiation factor 3 subunit E OS=Homo sapiens OX=9606 GN=EIF3E PE=1 SV=1	1.406551423	0.846263293
P04114	Apolipoprotein B-100 OS=Homo sapiens OX=9606 GN=APOB PE=1 SV=2	-2.939635732	0.84655918
P62333	26S proteasome regulatory subunit 10B OS=Homo sapiens OX=9606 GN=PSMC6 PE=1 SV=1	1.927482382	0.852035543
P26038	Moesin OS=Homo sapiens OX=9606 GN=MSN PE=1 SV=3	-2.533678677	0.879100257

Q15404	Ras suppressor protein 1 OS=Homo sapiens OX=9606 GN=RSU1 PE=1 SV=3	1.737277673	0.889462447
Q9HCP6	Protein-cysteine N-palmitoyltransferase HHAT-like protein OS=Homo sapiens OX=9606 GN=HHATL PE=2 SV=1	-2.57527346	0.889677812
Q9BRF8	Serine/threonine-protein phosphatase CPPED1 OS=Homo sapiens OX=9606 GN=CPPED1 PE=1 SV=3	1.268497162	0.891277281
Q99714	3-hydroxyacyl-CoA dehydrogenase type-2 OS=Homo sapiens OX=9606 GN=HSD17B10 PE=1 SV=3	-1.96272396	0.909716166
P62820	Ras-related protein Rab-1A OS=Homo sapiens OX=9606 GN=RAB1A PE=1 SV=3	2.084684023	0.923826362
Q9UPY8	Microtubule-associated protein RP/EB family member 3 OS=Homo sapiens OX=9606 GN=MAPRE3 PE=1 SV=1	3.036344547	0.94144857
P10768	S-formylglutathione hydrolase OS=Homo sapiens OX=9606 GN=ESD PE=1 SV=2	-1.753604975	0.951114042
Q16563	Synaptophysin-like protein 1 OS=Homo sapiens OX=9606 GN=SYPL1 PE=1 SV=1	-1.345002676	0.967938208
Q02543	60S ribosomal protein L18a OS=Homo sapiens OX=9606 GN=RPL18A PE=1 SV=2	-1.267242879	0.969339071
P09972	Fructose-bisphosphate aldolase C OS=Homo sapiens OX=9606 GN=ALDOC PE=1 SV=2	-2.206466289	0.985220924
Q9NPC6	Myozenin-2 OS=Homo sapiens OX=9606 GN=MYOZ2 PE=1 SV=1	1.686847589	0.987158064
P27816-5	Isoform 5 of Microtubule-associated protein 4 OS=Homo sapiens OX=9606 GN=MAP4	1.300139868	0.98855263
P52815	39S ribosomal protein L12, mitochondrial OS=Homo sapiens OX=9606 GN=MRPL12 PE=1 SV=2	2.048616744	0.993732755
P00568	Adenylate kinase isoenzyme 1 OS=Homo sapiens OX=9606 GN=AK1 PE=1 SV=3	-1.370724053	1
Q9BTV4	Transmembrane protein 43 OS=Homo sapiens OX=9606 GN=TMEM43 PE=1 SV=1	1.729971287	1
P02763	Alpha-1-acid glycoprotein 1 OS=Homo sapiens OX=9606 GN=ORM1 PE=1 SV=1	1.609223958	1
Q16134	Electron transfer flavoprotein-ubiquinone oxidoreductase, mitochondrial OS=Homo sapiens OX=9606 GN=ETFDH PE=1 SV=2	-1.836997547	1
O14880	Microsomal glutathione S-transferase 3 OS=Homo sapiens OX=9606 GN=MGST3 PE=1 SV=1	-1.895716522	1
P51452	Dual specificity protein phosphatase 3 OS=Homo sapiens OX=9606 GN=DUSP3 PE=1 SV=1	1.608846928	1
Q8N3K9	Cardiomyopathy-associated protein 5 OS=Homo sapiens OX=9606 GN=CMYA5 PE=1 SV=3	-2.613666481	1
Q16555	Dihydropyrimidinase-related protein 2 OS=Homo sapiens OX=9606 GN=DPYSL2 PE=1 SV=1	-1.252420277	1
Q9UNF0	Protein kinase C and casein kinase substrate in neurons protein 2 OS=Homo sapiens OX=9606 GN=PACSIN2 PE=1 SV=2	1.735692209	1

P21980	Protein-glutamine gamma-glutamyltransferase 2 OS=Homo sapiens OX=9606 GN=TGM2 PE=1 SV=2	-2.218741613	1
O43707	Alpha-actinin-4 OS=Homo sapiens OX=9606 GN=ACTN4 PE=1 SV=2	-1.604338448	1
Q9ULX7	Carbonic anhydrase 14 OS=Homo sapiens OX=9606 GN=CA14 PE=1 SV=1	1.382846095	1
O43598	2'-deoxynucleoside 5'-phosphate N-hydrolase 1 OS=Homo sapiens OX=9606 GN=DNPH1 PE=1 SV=1	1.972253747	1
Q6H8Q1-9	Isoform 9 of Actin-binding LIM protein 2 OS=Homo sapiens OX=9606 GN=ABLIM2	-2.447573595	1
O75128-2	Isoform 2 of Protein cordon-bleu OS=Homo sapiens OX=9606 GN=COBL	3.130962838	1
Q8IWW8	Hydroxyacid-oxoacid transhydrogenase, mitochondrial OS=Homo sapiens OX=9606 GN=ADHFE1 PE=1 SV=1	-1.526513297	1
Q6UXV4	MICOS complex subunit MIC27 OS=Homo sapiens OX=9606 GN=APOOL PE=1 SV=1	1.168497451	1
Q9BVK6	Transmembrane emp24 domain-containing protein 9 OS=Homo sapiens OX=9606 GN=TMED9 PE=1 SV=2	2.53101515	1
Q13061	Triadin OS=Homo sapiens OX=9606 GN=TRDN PE=1 SV=4	-1.080366734	1
Q53FT3	Protein Hikeshi OS=Homo sapiens OX=9606 GN=HIKESHI PE=1 SV=2	1.257492278	1
Q13409	Cytoplasmic dynein 1 intermediate chain 2 OS=Homo sapiens OX=9606 GN=DYNC1I2 PE=1 SV=3	-1.592562977	1
Q8NE86	Calcium uniporter protein, mitochondrial OS=Homo sapiens OX=9606 GN=MCU PE=1 SV=1	-1.294089309	1
P48449	Lanosterol synthase OS=Homo sapiens OX=9606 GN=LSS PE=1 SV=1	1.967229853	1
P31153	S-adenosylmethionine synthase isoform type-2 OS=Homo sapiens OX=9606 GN=MAT2A PE=1 SV=1	1.69475785	1
Q9UNE7	E3 ubiquitin-protein ligase CHIP OS=Homo sapiens OX=9606 GN=STUB1 PE=1 SV=2	-1.109644115	1
P02647	Apolipoprotein A-I OS=Homo sapiens OX=9606 GN=APOA1 PE=1 SV=1	-1.964481552	1
O43678	NADH dehydrogenase [ubiquinone] 1 alpha subcomplex subunit 2 OS=Homo sapiens OX=9606 GN=NDUFA2 PE=1 SV=3	-0.961261929	1
Q8WUM4	Programmed cell death 6-interacting protein OS=Homo sapiens OX=9606 GN=PDCD6IP PE=1 SV=1	-2.008070266	1
P62805	Histone H4 OS=Homo sapiens OX=9606 GN=H4C1 PE=1 SV=2	-2.685176082	1
Q5JWF2	Guanine nucleotide-binding protein G(s) subunit alpha isoforms XLas OS=Homo sapiens OX=9606 GN=GNAS PE=1 SV=2	2.757115287	1
P04632	Calpain small subunit 1 OS=Homo sapiens OX=9606 GN=CAPNS1 PE=1 SV=1	2.458596118	1

Q96CX2	BTB/POZ domain-containing protein KCTD12 OS=Homo sapiens OX=9606 GN=KCTD12 PE=1 SV=1	1.640676572	1
Q9NYL2-2	Isoform 2 of Mitogen-activated protein kinase kinase kinase 20 OS=Homo sapiens OX=9606 GN=MAP3K20	-2.561207982	1
Q08722	Leukocyte surface antigen CD47 OS=Homo sapiens OX=9606 GN=CD47 PE=1 SV=1	2.404455239	1
Q14240-2	Isoform 2 of Eukaryotic initiation factor 4A-II OS=Homo sapiens OX=9606 GN=EIF4A2	-3.029776379	1
Q13263	Transcription intermediary factor 1-beta OS=Homo sapiens OX=9606 GN=TRIM28 PE=1 SV=5	1.754239954	1
Q8TBC4	NEDD8-activating enzyme E1 catalytic subunit OS=Homo sapiens OX=9606 GN=UBA3 PE=1 SV=2	2.433527522	1
Q5SRD1	Putative mitochondrial import inner membrane translocase subunit Tim23B OS=Homo sapiens OX=9606 GN=TIMM23B PE=5 SV=2	1.164644206	1
P07477	Trypsin-1 OS=Homo sapiens OX=9606 GN=PRSS1 PE=1 SV=1	-2.249072131	1
Q9H0P0	Cytosolic 5'-nucleotidase 3A OS=Homo sapiens OX=9606 GN=NT5C3A PE=1 SV=3	-1.855653607	1
Q7KZF4	Staphylococcal nuclease domain-containing protein 1 OS=Homo sapiens OX=9606 GN=SND1 PE=1 SV=1	2.010142656	1
Q9NTM9	Copper homeostasis protein cutC homolog OS=Homo sapiens OX=9606 GN=CUTC PE=1 SV=1	-1.859850698	1
P22626	Heterogeneous nuclear ribonucleoproteins A2/B1 OS=Homo sapiens OX=9606 GN=HNRNPA2B1 PE=1 SV=2	-1.822394982	1
Q9HC38-2	Isoform 2 of Glyoxalase domain-containing protein 4 OS=Homo sapiens OX=9606 GN=GLOD4	-1.16680546	1
Q99832	T-complex protein 1 subunit eta OS=Homo sapiens OX=9606 GN=CCT7 PE=1 SV=2	1.887308271	1
O75347	Tubulin-specific chaperone A OS=Homo sapiens OX=9606 GN=TBCA PE=1 SV=3	1.455837123	1
Q13561-2	Isoform 2 of Dynactin subunit 2 OS=Homo sapiens OX=9606 GN=DCTN2	-1.347800164	1
P24534	Elongation factor 1-beta OS=Homo sapiens OX=9606 GN=EEF1B2 PE=1 SV=3	-2.160072754	1
O75874	Isocitrate dehydrogenase [NADP] cytoplasmic OS=Homo sapiens OX=9606 GN=IDH1 PE=1 SV=2	-1.093002483	1
Q9UHB6-4	Isoform 4 of LIM domain and actin-binding protein 1 OS=Homo sapiens OX=9606 GN=LIMA1	1.595347442	1
P55822	SH3 domain-binding glutamic acid-rich protein OS=Homo sapiens OX=9606 GN=SH3BGR PE=2 SV=3	-2.581626704	1
P02655	Apolipoprotein C-II OS=Homo sapiens OX=9606 GN=APOC2 PE=1 SV=1	2.171246631	1

Q15063-5	Isoform 5 of Periostin OS=Homo sapiens OX=9606 GN=POSTN	1.612794004	1
Q9Y508	E3 ubiquitin-protein ligase RNF114 OS=Homo sapiens OX=9606 GN=RNF114 PE=1 SV=1	1.539206037	1
P49368	T-complex protein 1 subunit gamma OS=Homo sapiens OX=9606 GN=CCT3 PE=1 SV=4	1.137374151	1
P30085-3	Isoform 3 of UMP-CMP kinase OS=Homo sapiens OX=9606 GN=CMPK1	1.516439343	1
O14818	Proteasome subunit alpha type-7 OS=Homo sapiens OX=9606 GN=PSMA7 PE=1 SV=1	0.829323841	1
Q14108	Lysosome membrane protein 2 OS=Homo sapiens OX=9606 GN=SCARB2 PE=1 SV=2	1.326412195	1
O95810	Caveolae-associated protein 2 OS=Homo sapiens OX=9606 GN=CAVIN2 PE=1 SV=3	-1.886003136	1
P19338	Nucleolin OS=Homo sapiens OX=9606 GN=NCL PE=1 SV=3	-1.391361044	1
P35080-2	Isoform IIb of Profilin-2 OS=Homo sapiens OX=9606 GN=PFN2	2.600005318	1
P35613	Basigin OS=Homo sapiens OX=9606 GN=BSG PE=1 SV=2	-1.607953526	1
P16298-4	Isoform 4 of Serine/threonine-protein phosphatase 2B catalytic subunit beta isoform OS=Homo sapiens OX=9606 GN=PPP3CB	2.400207401	1
Q9BXN1	Asporin OS=Homo sapiens OX=9606 GN=ASPN PE=1 SV=2	-1.619084833	1
P49591	Serine--tRNA ligase, cytoplasmic OS=Homo sapiens OX=9606 GN=SARS PE=1 SV=3	0.985163761	1
P11021	Endoplasmic reticulum chaperone BiP OS=Homo sapiens OX=9606 GN=HSPA5 PE=1 SV=2	-1.333403829	1
P14543	Nidogen-1 OS=Homo sapiens OX=9606 GN=NID1 PE=1 SV=3	-1.878238093	1
Q9NSC5	Homer protein homolog 3 OS=Homo sapiens OX=9606 GN=HOMER3 PE=1 SV=2	2.206992156	1
Q9UPQ0-3	Isoform 3 of LIM and calponin homology domains-containing protein 1 OS=Homo sapiens OX=9606 GN=LIMCH1	2.224806484	1
O60313-10	Isoform 4 of Dynamin-like 120 kDa protein, mitochondrial OS=Homo sapiens OX=9606 GN=OPA1	-1.019496777	1
P52209	6-phosphogluconate dehydrogenase, decarboxylating OS=Homo sapiens OX=9606 GN=PGD PE=1 SV=3	-1.151509636	1
P41236	Protein phosphatase inhibitor 2 OS=Homo sapiens OX=9606 GN=PPP1R2 PE=1 SV=2	1.19997399	1
P07602-3	Isoform Sap-mu-9 of Prosaposin OS=Homo sapiens OX=9606 GN=PSAP	-3.276566671	1
P38646	Stress-70 protein, mitochondrial OS=Homo sapiens OX=9606 GN=HSPA9 PE=1 SV=2	-1.120417178	1
P30626	Sorcin OS=Homo sapiens OX=9606 GN=SRI PE=1 SV=1	-1.955934763	1

Q15819	Ubiquitin-conjugating enzyme E2 variant 2 OS=Homo sapiens OX=9606 GN=UBE2V2 PE=1 SV=4	3.492162598	1
P46778	60S ribosomal protein L21 OS=Homo sapiens OX=9606 GN=RPL21 PE=1 SV=2	-1.060794715	1
P62424	60S ribosomal protein L7a OS=Homo sapiens OX=9606 GN=RPL7A PE=1 SV=2	-2.252870162	1
P16083	Ribosyldihydronicotinamide dehydrogenase [quinone] OS=Homo sapiens OX=9606 GN=NQO2 PE=1 SV=5	1.994914222	1
Q8N4T8	Carbonyl reductase family member 4 OS=Homo sapiens OX=9606 GN=CBR4 PE=1 SV=3	0.704254191	1
Q8TAT6-2	Isoform 2 of Nuclear protein localization protein 4 homolog OS=Homo sapiens OX=9606 GN=NPLOC4	-0.948513069	1
O00232	26S proteasome non-ATPase regulatory subunit 12 OS=Homo sapiens OX=9606 GN=PSMD12 PE=1 SV=3	-1.018275454	1
P04040	Catalase OS=Homo sapiens OX=9606 GN=CAT PE=1 SV=3	-2.043083868	1
O75832	26S proteasome non-ATPase regulatory subunit 10 OS=Homo sapiens OX=9606 GN=PSMD10 PE=1 SV=1	2.600852468	1
P83111	Serine beta-lactamase-like protein LACTB, mitochondrial OS=Homo sapiens OX=9606 GN=LACTB PE=1 SV=2	2.000025036	1
P51858	Hepatoma-derived growth factor OS=Homo sapiens OX=9606 GN=HDGF PE=1 SV=1	-2.167852073	1
P63027	Vesicle-associated membrane protein 2 OS=Homo sapiens OX=9606 GN=VAMP2 PE=1 SV=3	1.775349365	1
P50995	Annexin A11 OS=Homo sapiens OX=9606 GN=ANXA11 PE=1 SV=1	-1.269050476	1
P02545	Prelamin-A/C OS=Homo sapiens OX=9606 GN=LMNA PE=1 SV=1	-1.727616406	1
P56181-2	Isoform 2 of NADH dehydrogenase [ubiquinone] flavoprotein 3, mitochondrial OS=Homo sapiens OX=9606 GN=NDUFV3	1.360144138	1
Q86Y39-2	Isoform 2 of NADH dehydrogenase [ubiquinone] 1 alpha subcomplex subunit 11 OS=Homo sapiens OX=9606 GN=NDUFA11	1.279201553	1
O95299	NADH dehydrogenase [ubiquinone] 1 alpha subcomplex subunit 10, mitochondrial OS=Homo sapiens OX=9606 GN=NDUFA10 PE=1 SV=1	-1.6881864	1
P39687	Acidic leucine-rich nuclear phosphoprotein 32 family member A OS=Homo sapiens OX=9606 GN=ANP32A PE=1 SV=1	3.109202155	1
O75298	Reticulon-2 OS=Homo sapiens OX=9606 GN=RTN2 PE=1 SV=1	-1.534782405	1
P54578	Ubiquitin carboxyl-terminal hydrolase 14 OS=Homo sapiens OX=9606 GN=USP14 PE=1 SV=3	-0.915544419	1

P67775	Serine/threonine-protein phosphatase 2A catalytic subunit alpha isoform OS=Homo sapiens OX=9606 GN=PPP2CA PE=1 SV=1	1.176930829	1
P52566	Rho GDP-dissociation inhibitor 2 OS=Homo sapiens OX=9606 GN=ARHGDIB PE=1 SV=3	-1.821683057	1
Q05682	Caldesmon OS=Homo sapiens OX=9606 GN=CALD1 PE=1 SV=3	1.644842212	1
P10599	Thioredoxin OS=Homo sapiens OX=9606 GN=TXN PE=1 SV=3	-1.170405835	1
P09651	Heterogeneous nuclear ribonucleoprotein A1 OS=Homo sapiens OX=9606 GN=HNRNPA1 PE=1 SV=5	-1.812656531	1
Q9UQM7-2	Isoform B of Calcium/calmodulin-dependent protein kinase type II subunit alpha OS=Homo sapiens OX=9606 GN=CAMK2A	1.549853951	1
P51553	Isocitrate dehydrogenase [NAD] subunit gamma, mitochondrial OS=Homo sapiens OX=9606 GN=IDH3G PE=1 SV=1	-2.072252643	1
P10253	Lysosomal alpha-glucosidase OS=Homo sapiens OX=9606 GN=GAA PE=1 SV=4	2.447755681	1
P35443	Thrombospondin-4 OS=Homo sapiens OX=9606 GN=THBS4 PE=1 SV=2	-2.274109839	1
P03886	NADH-ubiquinone oxidoreductase chain 1 OS=Homo sapiens OX=9606 GN=MT-ND1 PE=1 SV=1	1.034319222	1
Q92506	Estradiol 17-beta-dehydrogenase 8 OS=Homo sapiens OX=9606 GN=HSD17B8 PE=1 SV=2	1.294344667	1
P61086	Ubiquitin-conjugating enzyme E2 K OS=Homo sapiens OX=9606 GN=UBE2K PE=1 SV=3	1.25097214	1
Q86WA6	Valacyclovir hydrolase OS=Homo sapiens OX=9606 GN=BPHL PE=1 SV=1	1.263526922	1
Q00839	Heterogeneous nuclear ribonucleoprotein U OS=Homo sapiens OX=9606 GN=HNRNPU PE=1 SV=6	-1.871375855	1
Q7Z7G0	Target of Nesh-SH3 OS=Homo sapiens OX=9606 GN=ABI3BP PE=1 SV=1	-1.111315072	1
P62701	40S ribosomal protein S4, X isoform OS=Homo sapiens OX=9606 GN=RPS4X PE=1 SV=2	0.952687375	1
Q5VWP3-3	Isoform 3 of Muscular LMNA-interacting protein OS=Homo sapiens OX=9606 GN=MLIP	-0.866847626	1
O95183	Vesicle-associated membrane protein 5 OS=Homo sapiens OX=9606 GN=VAMP5 PE=1 SV=1	-0.891663144	1
Q6P1A2	Lysophospholipid acyltransferase 5 OS=Homo sapiens OX=9606 GN=LPCAT3 PE=1 SV=1	0.623044323	1
O95861-2	Isoform 2 of 3'(2'),5'-bisphosphate nucleotidase 1 OS=Homo sapiens OX=9606 GN=BPNT1	1.805409904	1

P02788	Lactotransferrin OS=Homo sapiens OX=9606 GN=LTF PE=1 SV=6	1.912357728	1
P17152	Transmembrane protein 11, mitochondrial OS=Homo sapiens OX=9606 GN=TMEM11 PE=1 SV=1	2.17919763	1
P55735-3	Isoform 3 of Protein SEC13 homolog OS=Homo sapiens OX=9606 GN=SEC13	1.420209651	1
P16615-2	Isoform 2 of Sarcoplasmic/endoplasmic reticulum calcium ATPase 2 OS=Homo sapiens OX=9606 GN=ATP2A2	2.570501092	1
Q9Y450	HBS1-like protein OS=Homo sapiens OX=9606 GN=HBS1L PE=1 SV=1	0.695065411	1
P30101	Protein disulfide-isomerase A3 OS=Homo sapiens OX=9606 GN=PDIA3 PE=1 SV=4	-2.082298689	1
P34896	Serine hydroxymethyltransferase, cytosolic OS=Homo sapiens OX=9606 GN=SHMT1 PE=1 SV=1	0.91746295	1
P21333	Filamin-A OS=Homo sapiens OX=9606 GN=FLNA PE=1 SV=4	-1.269589485	1
Q9GZV1	Ankyrin repeat domain-containing protein 2 OS=Homo sapiens OX=9606 GN=ANKRD2 PE=1 SV=3	-1.711317799	1
P12429	Annexin A3 OS=Homo sapiens OX=9606 GN=ANXA3 PE=1 SV=3	-1.139437326	1
Q7Z4W1	L-xylulose reductase OS=Homo sapiens OX=9606 GN=DCXR PE=1 SV=2	1.184509856	1
P02794	Ferritin heavy chain OS=Homo sapiens OX=9606 GN=FTH1 PE=1 SV=2	1.508565134	1
P06748	Nucleophosmin OS=Homo sapiens OX=9606 GN=NPM1 PE=1 SV=2	-1.932312814	1
Q14152	Eukaryotic translation initiation factor 3 subunit A OS=Homo sapiens OX=9606 GN=EIF3A PE=1 SV=1	1.857839821	1
P62736	Actin, aortic smooth muscle OS=Homo sapiens OX=9606 GN=ACTA2 PE=1 SV=1	1.412065619	1
Q99757	Thioredoxin, mitochondrial OS=Homo sapiens OX=9606 GN=TXN2 PE=1 SV=2	1.129321735	1
P29401-2	Isoform 2 of Transketolase OS=Homo sapiens OX=9606 GN=TKT	1.943711534	1
P14314	Glucosidase 2 subunit beta OS=Homo sapiens OX=9606 GN=PRKCSH PE=1 SV=2	1.091342739	1
Q02641-2	Isoform 2 of Voltage-dependent L-type calcium channel subunit beta-1 OS=Homo sapiens OX=9606 GN=CACNB1	-1.892060355	1
Q9HCC0	Methylcrotonoyl-CoA carboxylase beta chain, mitochondrial OS=Homo sapiens OX=9606 GN=MCCC2 PE=1 SV=1	1.140418592	1
Q9Y3C6	Peptidyl-prolyl cis-trans isomerase-like 1 OS=Homo sapiens OX=9606 GN=PPIL1 PE=1 SV=1	2.288240598	1
Q6DD88	Atlastin-3 OS=Homo sapiens OX=9606 GN=ATL3 PE=1 SV=1	2.156575567	1
P01619	Immunoglobulin kappa variable 3-20 OS=Homo sapiens OX=9606 GN=IGKV3-20 PE=1 SV=2	2.44273478	1

P21926	CD9 antigen OS=Homo sapiens OX=9606 GN=CD9 PE=1 SV=4	-1.619016021	1
Q13618	Cullin-3 OS=Homo sapiens OX=9606 GN=CUL3 PE=1 SV=2	-1.099353034	1
O14983	Sarcoplasmic/endoplasmic reticulum calcium ATPase 1 OS=Homo sapiens OX=9606 GN=ATP2A1 PE=1 SV=1	-1.790060364	1
Q00872-6	Isoform 6 of Myosin-binding protein C, slow-type OS=Homo sapiens OX=9606 GN=MYBPC1	-1.948180029	1
P31150	Rab GDP dissociation inhibitor alpha OS=Homo sapiens OX=9606 GN=GDI1 PE=1 SV=2	-0.747321256	1
P02765	Alpha-2-HS-glycoprotein OS=Homo sapiens OX=9606 GN=AHSG PE=1 SV=2	-0.781426812	1
P22234-2	Isoform 2 of Multifunctional protein ADE2 OS=Homo sapiens OX=9606 GN=PAICS	0.695827569	1
P63104	14-3-3 protein zeta/delta OS=Homo sapiens OX=9606 GN=YWHAZ PE=1 SV=1	-1.040097958	1
P67936	Tropomyosin alpha-4 chain OS=Homo sapiens OX=9606 GN=TPM4 PE=1 SV=3	3.459182462	1
P35749	Myosin-11 OS=Homo sapiens OX=9606 GN=MYH11 PE=1 SV=3	-2.234344545	1
Q04446	1,4-alpha-glucan-branching enzyme OS=Homo sapiens OX=9606 GN=GBE1 PE=1 SV=3	-1.122711782	1
P62753	40S ribosomal protein S6 OS=Homo sapiens OX=9606 GN=RPS6 PE=1 SV=1	-1.505484678	1
P50914	60S ribosomal protein L14 OS=Homo sapiens OX=9606 GN=RPL14 PE=1 SV=4	-1.517060793	1
Q12906-7	Isoform 7 of Interleukin enhancer-binding factor 3 OS=Homo sapiens OX=9606 GN=ILF3	-0.598328267	1
A8MU46	Smoothelin-like protein 1 OS=Homo sapiens OX=9606 GN=SMTNL1 PE=1 SV=2	-1.25512196	1
P84098	60S ribosomal protein L19 OS=Homo sapiens OX=9606 GN=RPL19 PE=1 SV=1	1.959345139	1
Q5XPI4-2	Isoform 2 of E3 ubiquitin-protein ligase RNF123 OS=Homo sapiens OX=9606 GN=RNF123	-1.686690182	1
Q15631	Translin OS=Homo sapiens OX=9606 GN=TSN PE=1 SV=1	2.165442147	1
P05091	Aldehyde dehydrogenase, mitochondrial OS=Homo sapiens OX=9606 GN=ALDH2 PE=1 SV=2	-0.671710168	1
Q13557-6	Isoform Delta 9 of Calcium/calmodulin-dependent protein kinase type II subunit delta OS=Homo sapiens OX=9606 GN=CAMK2D	-1.601557681	1
P61513	60S ribosomal protein L37a OS=Homo sapiens OX=9606 GN=RPL37A PE=1 SV=2	-1.534855115	1
P31751	RAC-beta serine/threonine-protein kinase OS=Homo sapiens OX=9606 GN=AKT2 PE=1 SV=2	1.239844848	1
P41250	Glycine--tRNA ligase OS=Homo sapiens OX=9606 GN=GARS1 PE=1 SV=3	1.942749408	1

Q9NUJ1	Mycophenolic acid acyl-glucuronide esterase, mitochondrial OS=Homo sapiens OX=9606 GN=ABHD10 PE=1 SV=1	1.84725216	1
Q07866-9	Isoform I of Kinesin light chain 1 OS=Homo sapiens OX=9606 GN=KLC1	1.78648782	1
Q08495	Dematin OS=Homo sapiens OX=9606 GN=DMTN PE=1 SV=3	1.359966928	1
Q1KMD3	Heterogeneous nuclear ribonucleoprotein U-like protein 2 OS=Homo sapiens OX=9606 GN=HNRNPUL2 PE=1 SV=1	2.52415219	1
Q00872-4	Isoform 4 of Myosin-binding protein C, slow-type OS=Homo sapiens OX=9606 GN=MYBPC1	-0.738475205	1
P30048	Thioredoxin-dependent peroxide reductase, mitochondrial OS=Homo sapiens OX=9606 GN=PRDX3 PE=1 SV=3	-2.204214573	1
P63000-2	Isoform B of Ras-related C3 botulinum toxin substrate 1 OS=Homo sapiens OX=9606 GN=RAC1	1.885282779	1
P06703	Protein S100-A6 OS=Homo sapiens OX=9606 GN=S100A6 PE=1 SV=1	-0.643645243	1
P23381	Tryptophan--tRNA ligase, cytoplasmic OS=Homo sapiens OX=9606 GN=WARS PE=1 SV=2	-1.837308822	1
Q15369	Elongin-C OS=Homo sapiens OX=9606 GN=ELOC PE=1 SV=1	-2.659376274	1
O95298	NADH dehydrogenase [ubiquinone] 1 subunit C2 OS=Homo sapiens OX=9606 GN=NDUFC2 PE=1 SV=1	-0.968109113	1
P28072	Proteasome subunit beta type-6 OS=Homo sapiens OX=9606 GN=PSMB6 PE=1 SV=4	-0.586521687	1
P04439-2	Isoform 2 of HLA class I histocompatibility antigen, A alpha chain OS=Homo sapiens OX=9606 GN=HLA-A	2.013573883	1
Q9UMS4	Pre-mRNA-processing factor 19 OS=Homo sapiens OX=9606 GN=PRPF19 PE=1 SV=1	0.862540758	1
Q96HC4	PDZ and LIM domain protein 5 OS=Homo sapiens OX=9606 GN=PDLIM5 PE=1 SV=5	3.274227174	1
P30519	Heme oxygenase 2 OS=Homo sapiens OX=9606 GN=HMOX2 PE=1 SV=2	0.883831812	1
Q9UHP9	Small muscular protein OS=Homo sapiens OX=9606 GN=SMPX PE=2 SV=3	-1.936392019	1
P62241	40S ribosomal protein S8 OS=Homo sapiens OX=9606 GN=RPS8 PE=1 SV=2	-1.695844455	1
O75608	Acyl-protein thioesterase 1 OS=Homo sapiens OX=9606 GN=LYPLA1 PE=1 SV=1	1.697232624	1
Q08257	Quinone oxidoreductase OS=Homo sapiens OX=9606 GN=CRYZ PE=1 SV=1	-2.26528619	1
Q86X55	Histone-arginine methyltransferase CARM1 OS=Homo sapiens OX=9606 GN=CARM1 PE=1 SV=3	-1.252447424	1
Q93052	Lipoma-preferred partner OS=Homo sapiens OX=9606 GN=LPP PE=1 SV=1	-1.372368218	1
P09429	High mobility group protein B1 OS=Homo sapiens OX=9606 GN=HMGB1 PE=1 SV=3	-1.977248316	1

P00492	Hypoxanthine-guanine phosphoribosyltransferase OS=Homo sapiens OX=9606 GN=HPRT1 PE=1 SV=2	1.364905726	1
O75439	Mitochondrial-processing peptidase subunit beta OS=Homo sapiens OX=9606 GN=PMPCB PE=1 SV=2	1.900805537	1
P31942	Heterogeneous nuclear ribonucleoprotein H3 OS=Homo sapiens OX=9606 GN=HNRNPH3 PE=1 SV=2	-0.427477548	1
Q4G0N4	NAD kinase 2, mitochondrial OS=Homo sapiens OX=9606 GN=NADK2 PE=1 SV=2	0.687305917	1
O75915	PRA1 family protein 3 OS=Homo sapiens OX=9606 GN=ARL6IP5 PE=1 SV=1	-1.214320141	1
P43243	Matrin-3 OS=Homo sapiens OX=9606 GN=MATR3 PE=1 SV=2	-0.404142991	1
P62899	60S ribosomal protein L31 OS=Homo sapiens OX=9606 GN=RPL31 PE=1 SV=1	0.933239736	1
Q96IU4	Protein ABHD14B OS=Homo sapiens OX=9606 GN=ABHD14B PE=1 SV=1	1.061083408	1
Q8WTS6	Histone-lysine N-methyltransferase SETD7 OS=Homo sapiens OX=9606 GN=SETD7 PE=1 SV=1	1.562228441	1
Q96DH6	RNA-binding protein Musashi homolog 2 OS=Homo sapiens OX=9606 GN=MSI2 PE=1 SV=1	0.728998473	1
P47985	Cytochrome b-c1 complex subunit Rieske, mitochondrial OS=Homo sapiens OX=9606 GN=UQCRFS1 PE=1 SV=2	-0.826362598	1
Q9Y2R0	Cytochrome c oxidase assembly factor 3 homolog, mitochondrial OS=Homo sapiens OX=9606 GN=COA3 PE=1 SV=1	1.476642789	1
Q15435	Protein phosphatase 1 regulatory subunit 7 OS=Homo sapiens OX=9606 GN=PPP1R7 PE=1 SV=1	-1.304890271	1
Q15149-3	Isoform 3 of Plectin OS=Homo sapiens OX=9606 GN=PLEC	1.462378247	1
Q9H0U4	Ras-related protein Rab-1B OS=Homo sapiens OX=9606 GN=RAB1B PE=1 SV=1	-1.068784823	1
P16403	Histone H1.2 OS=Homo sapiens OX=9606 GN=H1-2 PE=1 SV=2	2.309878381	1
Q86TP1	Exopolyphosphatase PRUNE1 OS=Homo sapiens OX=9606 GN=PRUNE1 PE=1 SV=2	1.374237301	1
P14406	Cytochrome c oxidase subunit 7A2, mitochondrial OS=Homo sapiens OX=9606 GN=COX7A2 PE=1 SV=1	-1.384763745	1
P27635	60S ribosomal protein L10 OS=Homo sapiens OX=9606 GN=RPL10 PE=1 SV=4	-0.336698886	1
P35237	Serpin B6 OS=Homo sapiens OX=9606 GN=SERPINB6 PE=1 SV=3	-0.69780876	1
P35659	Protein DEK OS=Homo sapiens OX=9606 GN=DEK PE=1 SV=1	1.797535042	1
P10909-2	Isoform 2 of Clusterin OS=Homo sapiens OX=9606 GN=CLU	-0.775951784	1
P07327	Alcohol dehydrogenase 1A OS=Homo sapiens OX=9606 GN=ADH1A PE=1 SV=2	1.479865012	1

P56134	ATP synthase subunit f, mitochondrial OS=Homo sapiens OX=9606 GN=ATP5MF PE=1 SV=3	1.458989844	1
P22059	Oxysterol-binding protein 1 OS=Homo sapiens OX=9606 GN=OSBP PE=1 SV=1	0.524932203	1
P62851	40S ribosomal protein S25 OS=Homo sapiens OX=9606 GN=RPS25 PE=1 SV=1	-1.19390011	1
Q9Y3C8	Ubiquitin-fold modifier-conjugating enzyme 1 OS=Homo sapiens OX=9606 GN=UFC1 PE=1 SV=3	1.389661305	1
Q8NCA5	Protein FAM98A OS=Homo sapiens OX=9606 GN=FAM98A PE=1 SV=2	1.097559879	1
Q00341	Vigilin OS=Homo sapiens OX=9606 GN=HDLBP PE=1 SV=2	0.634796362	1
Q16082	Heat shock protein beta-2 OS=Homo sapiens OX=9606 GN=HSPB2 PE=1 SV=2	-1.586150755	1
Q00013	55 kDa erythrocyte membrane protein OS=Homo sapiens OX=9606 GN=MPP1 PE=1 SV=2	0.571571648	1
Q16181	Septin-7 OS=Homo sapiens OX=9606 GN=SEPTIN7 PE=1 SV=2	-1.193586052	1
P53004	Biliverdin reductase A OS=Homo sapiens OX=9606 GN=BLVRA PE=1 SV=2	-1.590417646	1
O00429-6	Isoform 6 of Dynamin-1-like protein OS=Homo sapiens OX=9606 GN=DNM1L	-0.376317084	1
P01859	Immunoglobulin heavy constant gamma 2 OS=Homo sapiens OX=9606 GN=IGHG2 PE=1 SV=2	1.573734736	1
P60981	Destrin OS=Homo sapiens OX=9606 GN=DSTN PE=1 SV=3	-2.47473394	1
P11766	Alcohol dehydrogenase class-3 OS=Homo sapiens OX=9606 GN=ADH5 PE=1 SV=4	-1.637266363	1
Q9UKG1	DCC-interacting protein 13-alpha OS=Homo sapiens OX=9606 GN=APPL1 PE=1 SV=1	-0.334289693	1
P00734	Prothrombin OS=Homo sapiens OX=9606 GN=F2 PE=1 SV=2	-1.724804851	1
Q9NZ01	Very-long-chain enoyl-CoA reductase OS=Homo sapiens OX=9606 GN=TECR PE=1 SV=1	-1.560370594	1
Q6IAN0	Dehydrogenase/reductase SDR family member 7B OS=Homo sapiens OX=9606 GN=DHRS7B PE=1 SV=2	-0.260674841	1
O43583	Density-regulated protein OS=Homo sapiens OX=9606 GN=DENR PE=1 SV=2	0.992654475	1
Q16836-2	Isoform 2 of Hydroxyacyl-coenzyme A dehydrogenase, mitochondrial OS=Homo sapiens OX=9606 GN=HADH	1.826675769	1
P52943	Cysteine-rich protein 2 OS=Homo sapiens OX=9606 GN=CRIP2 PE=1 SV=1	-0.898116429	1
P02751-1	Isoform 1 of Fibronectin OS=Homo sapiens OX=9606 GN=FN1	-0.402486215	1
Q9BRX2	Protein pelota homolog OS=Homo sapiens OX=9606 GN=PELO PE=1 SV=2	-0.236773577	1

Q96MF2	SH3 and cysteine-rich domain-containing protein 3 OS=Homo sapiens OX=9606 GN=STAC3 PE=1 SV=1	-1.577171468	1
Q8NBN7	Retinol dehydrogenase 13 OS=Homo sapiens OX=9606 GN=RDH13 PE=1 SV=2	1.594308059	1
O43491	Band 4.1-like protein 2 OS=Homo sapiens OX=9606 GN=EPB41L2 PE=1 SV=1	-0.91549242	1
O95865	N(G),N(G)-dimethylarginine dimethylaminohydrolase 2 OS=Homo sapiens OX=9606 GN=DDAH2 PE=1 SV=1	-0.387493709	1
P52758	2-iminobutanoate/2-iminopropanoate deaminase OS=Homo sapiens OX=9606 GN=RIDA PE=1 SV=1	0.64766485	1
P10515	Dihydrolipoyllysine-residue acetyltransferase component of pyruvate dehydrogenase complex, mitochondrial OS=Homo sapiens OX=9606 GN=DLAT PE=1 SV=3	1.011792339	1
O14737	Programmed cell death protein 5 OS=Homo sapiens OX=9606 GN=PDCD5 PE=1 SV=3	1.227962584	1
O95831	Apoptosis-inducing factor 1, mitochondrial OS=Homo sapiens OX=9606 GN=AIFM1 PE=1 SV=1	-0.977826178	1
P21291	Cysteine and glycine-rich protein 1 OS=Homo sapiens OX=9606 GN=CSRP1 PE=1 SV=3	1.319899633	1
O15061-2	Isoform 2 of Synemin OS=Homo sapiens OX=9606 GN=SYNM	1.087941378	1
P21912	Succinate dehydrogenase [ubiquinone] iron-sulfur subunit, mitochondrial OS=Homo sapiens OX=9606 GN=SDHB PE=1 SV=3	1.153056079	1
Q14894	Ketimine reductase mu-crystallin OS=Homo sapiens OX=9606 GN=CRYM PE=1 SV=1	0.696743886	1
P41091	Eukaryotic translation initiation factor 2 subunit 3 OS=Homo sapiens OX=9606 GN=EIF2S3 PE=1 SV=3	-0.209709923	1
P04843	Dolichyl-diphosphooligosaccharide--protein glycosyltransferase subunit 1 OS=Homo sapiens OX=9606 GN=RPN1 PE=1 SV=1	-0.627621282	1
Q04917	14-3-3 protein eta OS=Homo sapiens OX=9606 GN=YWHAH PE=1 SV=4	-0.227037244	1
P31937	3-hydroxyisobutyrate dehydrogenase, mitochondrial OS=Homo sapiens OX=9606 GN=HIBADH PE=1 SV=2	1.341602655	1
Q9HBL7	Plasminogen receptor (KT) OS=Homo sapiens OX=9606 GN=PLGRKT PE=1 SV=1	-1.183125187	1
P16152	Carbonyl reductase [NADPH] 1 OS=Homo sapiens OX=9606 GN=CBR1 PE=1 SV=3	-1.007512356	1
Q49B96	Cytochrome c oxidase assembly protein COX19 OS=Homo sapiens OX=9606 GN=COX19 PE=1 SV=1	-0.150495976	1
P33176	Kinesin-1 heavy chain OS=Homo sapiens OX=9606 GN=KIF5B PE=1 SV=1	0.494761414	1
Q15907	Ras-related protein Rab-11B OS=Homo sapiens OX=9606 GN=RAB11B PE=1 SV=4	-1.620950801	1
P53621-2	Isoform 2 of Coatomer subunit alpha OS=Homo sapiens OX=9606 GN=COPA	-1.446068349	1

Q9UL46	Proteasome activator complex subunit 2 OS=Homo sapiens OX=9606 GN=PSME2 PE=1 SV=4	0.882859298	1
P61978-2	Isoform 2 of Heterogeneous nuclear ribonucleoprotein K OS=Homo sapiens OX=9606 GN=HNRNPK	-0.196386236	1
Q9H061	Transmembrane protein 126A OS=Homo sapiens OX=9606 GN=TMEM126A PE=1 SV=1	1.344387319	1
P23946	Chymase OS=Homo sapiens OX=9606 GN=CMA1 PE=1 SV=1	-1.427548444	1
O60841	Eukaryotic translation initiation factor 5B OS=Homo sapiens OX=9606 GN=EIF5B PE=1 SV=4	1.06973389	1
Q14714	Sarcospan OS=Homo sapiens OX=9606 GN=SSPN PE=2 SV=3	1.790378683	1
P52294	Importin subunit alpha-5 OS=Homo sapiens OX=9606 GN=KPNA1 PE=1 SV=3	1.097707404	1
Q9BRX8	Peroxiredoxin-like 2A OS=Homo sapiens OX=9606 GN=PRXL2A PE=1 SV=3	-1.009309971	1
P61925	cAMP-dependent protein kinase inhibitor alpha OS=Homo sapiens OX=9606 GN=PKIA PE=1 SV=2	1.627447464	1
Q9BVC6	Transmembrane protein 109 OS=Homo sapiens OX=9606 GN=TMEM109 PE=1 SV=1	-0.095752245	1
Q96AQ6	Pre-B-cell leukemia transcription factor-interacting protein 1 OS=Homo sapiens OX=9606 GN=PBXIP1 PE=1 SV=1	-1.990145492	1
P07814	Bifunctional glutamate/proline--tRNA ligase OS=Homo sapiens OX=9606 GN=EPRS PE=1 SV=5	-0.979715321	1
Q03135	Caveolin-1 OS=Homo sapiens OX=9606 GN=CAV1 PE=1 SV=4	-0.210954715	1
Q9Y3E1	Hepatoma-derived growth factor-related protein 3 OS=Homo sapiens OX=9606 GN=HDGFL3 PE=1 SV=1	0.533574025	1
P35080	Profilin-2 OS=Homo sapiens OX=9606 GN=PFN2 PE=1 SV=3	1.463142217	1
P05109	Protein S100-A8 OS=Homo sapiens OX=9606 GN=S100A8 PE=1 SV=1	-0.172048286	1
Q9Y224	RNA transcription, translation and transport factor protein OS=Homo sapiens OX=9606 GN=RTRAF PE=1 SV=1	-0.01934249	1
Q13126-2	Isoform 2 of S-methyl-5'-thioadenosine phosphorylase OS=Homo sapiens OX=9606 GN=MTAP	-0.086996565	1
O15173-2	Isoform 2 of Membrane-associated progesterone receptor component 2 OS=Homo sapiens OX=9606 GN=PGRMC2	-1.489065809	1
P62140	Serine/threonine-protein phosphatase PP1-beta catalytic subunit OS=Homo sapiens OX=9606 GN=PPP1CB PE=1 SV=3	1.210923915	1
P46459	Vesicle-fusing ATPase OS=Homo sapiens OX=9606 GN=NSF PE=1 SV=3	0.534431346	1

P26583	High mobility group protein B2 OS=Homo sapiens OX=9606 GN=HMGB2 PE=1 SV=2	1.905631065	1
P00738	Haptoglobin OS=Homo sapiens OX=9606 GN=HP PE=1 SV=1	1.183339858	1
A0A0B4J2D5	Glutamine amidotransferase-like class 1 domain-containing protein 3B, mitochondrial OS=Homo sapiens OX=9606 GN=GATD3B PE=1 SV=1	1.104378505	1
P63208	S-phase kinase-associated protein 1 OS=Homo sapiens OX=9606 GN=SKP1 PE=1 SV=2	1.14358522	1
P01857	Immunoglobulin heavy constant gamma 1 OS=Homo sapiens OX=9606 GN=IGHG1 PE=1 SV=1	-1.13702609	1
Q02252	Methylmalonate-semialdehyde dehydrogenase [acylating], mitochondrial OS=Homo sapiens OX=9606 GN=ALDH6A1 PE=1 SV=2	1.527366496	1
P63098	Calcineurin subunit B type 1 OS=Homo sapiens OX=9606 GN=PPP3R1 PE=1 SV=2	1.372164489	1
P61158	Actin-related protein 3 OS=Homo sapiens OX=9606 GN=ACTR3 PE=1 SV=3	1.593947614	1
P16219	Short-chain specific acyl-CoA dehydrogenase, mitochondrial OS=Homo sapiens OX=9606 GN=ACADS PE=1 SV=1	-2.332002326	1
O94826	Mitochondrial import receptor subunit TOM70 OS=Homo sapiens OX=9606 GN=TOMM70 PE=1 SV=1	1.347355673	1
P00491	Purine nucleoside phosphorylase OS=Homo sapiens OX=9606 GN=PNP PE=1 SV=2	0.517371663	1
P04003	C4b-binding protein alpha chain OS=Homo sapiens OX=9606 GN=C4BPA PE=1 SV=2	-0.130365234	1
Q9H7Z7	Prostaglandin E synthase 2 OS=Homo sapiens OX=9606 GN=PTGES2 PE=1 SV=1	-2.052658287	1
Q96CN7	Isochorismatase domain-containing protein 1 OS=Homo sapiens OX=9606 GN=ISOC1 PE=1 SV=3	-1.131670861	1
Q15417	Calponin-3 OS=Homo sapiens OX=9606 GN=CNN3 PE=1 SV=1	-0.154716827	1
Q9BSH4	Translational activator of cytochrome c oxidase 1 OS=Homo sapiens OX=9606 GN=TACO1 PE=1 SV=1	1.405646233	1
Q15746	Myosin light chain kinase, smooth muscle OS=Homo sapiens OX=9606 GN=MYLK PE=1 SV=4	1.361184737	1
Q9BPX6-3	Isoform 3 of Calcium uptake protein 1, mitochondrial OS=Homo sapiens OX=9606 GN=MICU1	0.887701616	1
O15230	Laminin subunit alpha-5 OS=Homo sapiens OX=9606 GN=LAMA5 PE=1 SV=8	1.542477843	1
P20340	Ras-related protein Rab-6A OS=Homo sapiens OX=9606 GN=RAB6A PE=1 SV=3	0.985072265	1
P00338-3	Isoform 3 of L-lactate dehydrogenase A chain OS=Homo sapiens OX=9606 GN=LDHA	-1.190376934	1

Q14061	Cytochrome c oxidase copper chaperone OS=Homo sapiens OX=9606 GN=COX17 PE=1 SV=2	-1.495137667	1
Q9NY65	Tubulin alpha-8 chain OS=Homo sapiens OX=9606 GN=TUBA8 PE=1 SV=1	-1.877418049	1
P06396	Gelsolin OS=Homo sapiens OX=9606 GN=GSN PE=1 SV=1	-0.921450184	1
Q9H2U2-2	Isoform 2 of Inorganic pyrophosphatase 2, mitochondrial OS=Homo sapiens OX=9606 GN=PPA2	-0.27203244	1
Q03591	Complement factor H-related protein 1 OS=Homo sapiens OX=9606 GN=CFHR1 PE=1 SV=2	0.668771489	1
Q9Y3A5	Ribosome maturation protein SBDS OS=Homo sapiens OX=9606 GN=SBDS PE=1 SV=4	0.070511772	1
Q9UMR2	ATP-dependent RNA helicase DDX19B OS=Homo sapiens OX=9606 GN=DDX19B PE=1 SV=1	1.643608487	1
Q16762	Thiosulfate sulfurtransferase OS=Homo sapiens OX=9606 GN=TST PE=1 SV=4	1.573892587	1
Q9Y4G6	Talin-2 OS=Homo sapiens OX=9606 GN=TLN2 PE=1 SV=4	-0.05096914	1
Q9UNS2	COP9 signalosome complex subunit 3 OS=Homo sapiens OX=9606 GN=COPS3 PE=1 SV=3	1.438417521	1
P08572	Collagen alpha-2(IV) chain OS=Homo sapiens OX=9606 GN=COL4A2 PE=1 SV=4	1.434482207	1
P46439	Glutathione S-transferase Mu 5 OS=Homo sapiens OX=9606 GN=GSTM5 PE=1 SV=3	1.124311982	1
P83731	60S ribosomal protein L24 OS=Homo sapiens OX=9606 GN=RPL24 PE=1 SV=1	0.248710862	1
Q15942	Zyxin OS=Homo sapiens OX=9606 GN=ZYX PE=1 SV=1	-0.714411546	1
P55036	26S proteasome non-ATPase regulatory subunit 4 OS=Homo sapiens OX=9606 GN=PSMD4 PE=1 SV=1	-1.013148429	1
Q96AE4-2	Isoform 2 of Far upstream element-binding protein 1 OS=Homo sapiens OX=9606 GN=FUBP1	1.53116948	1
Q02338	D-beta-hydroxybutyrate dehydrogenase, mitochondrial OS=Homo sapiens OX=9606 GN=BDH1 PE=1 SV=3	-1.788598942	1
Q96L96	Alpha-protein kinase 3 OS=Homo sapiens OX=9606 GN=ALPK3 PE=1 SV=2	-0.944045109	1
Q16585	Beta-sarcoglycan OS=Homo sapiens OX=9606 GN=SGCB PE=1 SV=1	1.099797952	1
Q9BRC7	1-phosphatidylinositol 4,5-bisphosphate phosphodiesterase delta-4 OS=Homo sapiens OX=9606 GN=PLCD4 PE=1 SV=1	0.126105726	1
P02671	Fibrinogen alpha chain OS=Homo sapiens OX=9606 GN=FGA PE=1 SV=2	0.97571397	1
Q9NVJ2	ADP-ribosylation factor-like protein 8B OS=Homo sapiens OX=9606 GN=ARL8B PE=1 SV=1	1.91688838	1

P02675	Fibrinogen beta chain OS=Homo sapiens OX=9606 GN=FGB PE=1 SV=2	-0.757909563	1
Q8N5K1	CDGSH iron-sulfur domain-containing protein 2 OS=Homo sapiens OX=9606 GN=CISD2 PE=1 SV=1	0.606161317	1
O95373	Importin-7 OS=Homo sapiens OX=9606 GN=IPO7 PE=1 SV=1	0.610265125	1
P34932	Heat shock 70 kDa protein 4 OS=Homo sapiens OX=9606 GN=HSPA4 PE=1 SV=4	-0.716820267	1
Q9H993	Damage-control phosphatase ARMT1 OS=Homo sapiens OX=9606 GN=ARMT1 PE=1 SV=1	0.754530163	1
Q5VYK3	Proteasome adapter and scaffold protein ECM29 OS=Homo sapiens OX=9606 GN=ECPAS PE=1 SV=2	0.111403521	1
Q5VWZ2	Lysophospholipase-like protein 1 OS=Homo sapiens OX=9606 GN=LYPLAL1 PE=1 SV=3	1.378585326	1
P28074	Proteasome subunit beta type-5 OS=Homo sapiens OX=9606 GN=PSMB5 PE=1 SV=3	-0.794270837	1
Q9BR39	Junctophilin-2 OS=Homo sapiens OX=9606 GN=JPH2 PE=1 SV=2	1.274456001	1
P14174	Macrophage migration inhibitory factor OS=Homo sapiens OX=9606 GN=MIF PE=1 SV=4	0.152974226	1
Q5VST9-3	Isoform 3 of Obscurin OS=Homo sapiens OX=9606 GN=OBSCN	0.298837585	1
P43686	26S proteasome regulatory subunit 6B OS=Homo sapiens OX=9606 GN=PSMC4 PE=1 SV=2	-0.026316459	1
P29218-3	Isoform 3 of Inositol monophosphatase 1 OS=Homo sapiens OX=9606 GN=IMPA1	-1.065355541	1
P49720	Proteasome subunit beta type-3 OS=Homo sapiens OX=9606 GN=PSMB3 PE=1 SV=2	2.122469176	1
Q6GMV3	Putative peptidyl-tRNA hydrolase PTRHD1 OS=Homo sapiens OX=9606 GN=PTRHD1 PE=1 SV=1	1.219961102	1
Q15233	Non-POU domain-containing octamer-binding protein OS=Homo sapiens OX=9606 GN=NONO PE=1 SV=4	-1.439418567	1
Q9P0J0	NADH dehydrogenase [ubiquinone] 1 alpha subcomplex subunit 13 OS=Homo sapiens OX=9606 GN=NDUFA13 PE=1 SV=3	0.336004085	1
Q9H4M9	EH domain-containing protein 1 OS=Homo sapiens OX=9606 GN=EHD1 PE=1 SV=2	-1.265124586	1
Q14696	LRP chaperone MESD OS=Homo sapiens OX=9606 GN=MESD PE=1 SV=2	0.621189004	1
Q9NXA8	NAD-dependent protein deacylase sirtuin-5, mitochondrial OS=Homo sapiens OX=9606 GN=SIRT5 PE=1 SV=2	0.654994832	1
P02730	Band 3 anion transport protein OS=Homo sapiens OX=9606 GN=SLC4A1 PE=1 SV=3	-2.681706539	1
Q15257-2	Isoform 1 of Serine/threonine-protein phosphatase 2A activator OS=Homo sapiens OX=9606 GN=PTPA	-1.695329987	1

E7EW31	Proline-rich basic protein 1 OS=Homo sapiens OX=9606 GN=PROB1 PE=2 SV=2	-0.735491224	1
P12111	Collagen alpha-3(VI) chain OS=Homo sapiens OX=9606 GN=COL6A3 PE=1 SV=5	0.210710349	1
P83881	60S ribosomal protein L36a OS=Homo sapiens OX=9606 GN=RPL36A PE=1 SV=2	0.247132494	1
O43169	Cytochrome b5 type B OS=Homo sapiens OX=9606 GN=CYB5B PE=1 SV=3	0.557237163	1
P01834	Immunoglobulin kappa constant OS=Homo sapiens OX=9606 GN=IGKC PE=1 SV=2	0.827005862	1
P61981	14-3-3 protein gamma OS=Homo sapiens OX=9606 GN=YWHAG PE=1 SV=2	-1.027887452	1
P05155-3	Isoform 3 of Plasma protease C1 inhibitor OS=Homo sapiens OX=9606 GN=SERPING1	0.411353341	1
O60888-2	Isoform A of Protein CutA OS=Homo sapiens OX=9606 GN=CUTA	0.047223033	1
P53041	Serine/threonine-protein phosphatase 5 OS=Homo sapiens OX=9606 GN=PPP5C PE=1 SV=1	-0.61498836	1
P14550	Aldo-keto reductase family 1 member A1 OS=Homo sapiens OX=9606 GN=AKR1A1 PE=1 SV=3	-1.142932393	1
P84243	Histone H3.3 OS=Homo sapiens OX=9606 GN=H3-3A PE=1 SV=2	2.146879934	1
Q8WW59	SPRY domain-containing protein 4 OS=Homo sapiens OX=9606 GN=SPRYD4 PE=1 SV=2	0.355442838	1
P98160	Basement membrane-specific heparan sulfate proteoglycan core protein OS=Homo sapiens OX=9606 GN=HSPG2 PE=1 SV=4	-1.403623035	1
P07108-5	Isoform 5 of Acyl-CoA-binding protein OS=Homo sapiens OX=9606 GN=DBI	-0.640005736	1
Q9BX68	Histidine triad nucleotide-binding protein 2, mitochondrial OS=Homo sapiens OX=9606 GN=HINT2 PE=1 SV=1	-0.824994046	1
P53618	Coatomer subunit beta OS=Homo sapiens OX=9606 GN=COPB1 PE=1 SV=3	0.90669236	1
O43390-2	Isoform 2 of Heterogeneous nuclear ribonucleoprotein R OS=Homo sapiens OX=9606 GN=HNRNPR	-1.256973302	1
A0A087WW87	Immunoglobulin kappa variable 2-40 OS=Homo sapiens OX=9606 GN=IGKV2-40 PE=3 SV=2	0.12688369	1
P07738	Bisphosphoglycerate mutase OS=Homo sapiens OX=9606 GN=BPGM PE=1 SV=2	0.33496792	1
Q03252	Lamin-B2 OS=Homo sapiens OX=9606 GN=LMNB2 PE=1 SV=4	-0.641217315	1
P15880	40S ribosomal protein S2 OS=Homo sapiens OX=9606 GN=RPS2 PE=1 SV=2	-1.044679671	1
Q86VP6	Cullin-associated NEDD8-dissociated protein 1 OS=Homo sapiens OX=9606 GN=CAND1 PE=1 SV=2	0.034878436	1
P60520	Gamma-aminobutyric acid receptor-associated protein-like 2 OS=Homo sapiens OX=9606 GN=GABARAPL2 PE=1 SV=1	0.381806345	1

Q5VST9-6	Isoform 5 of Obscurin OS=Homo sapiens OX=9606 GN=OBSCN	-1.183830472	1
Q9GZT3	SRA stem-loop-interacting RNA-binding protein, mitochondrial OS=Homo sapiens OX=9606 GN=SLIRP PE=1 SV=1	-1.579361868	1
O75380	NADH dehydrogenase [ubiquinone] iron-sulfur protein 6, mitochondrial OS=Homo sapiens OX=9606 GN=NDUFS6 PE=1 SV=1	0.468474456	1
Q01995	Transgelin OS=Homo sapiens OX=9606 GN=TAGLN PE=1 SV=4	1.066733725	1
Q9GZM7	Tubulointerstitial nephritis antigen-like OS=Homo sapiens OX=9606 GN=TINAGL1 PE=1 SV=1	2.299025565	1
O95278	Laforin OS=Homo sapiens OX=9606 GN=EPM2A PE=1 SV=2	0.370228007	1
P62910	60S ribosomal protein L32 OS=Homo sapiens OX=9606 GN=RPL32 PE=1 SV=2	0.149408383	1
Q3ZCW2	Galectin-related protein OS=Homo sapiens OX=9606 GN=LGALSL PE=1 SV=2	0.123319758	1
Q13616	Cullin-1 OS=Homo sapiens OX=9606 GN=CUL1 PE=1 SV=2	1.738558552	1
A0A075B6R9	Probable non-functional immunoglobulin kappa variable 2D-24 OS=Homo sapiens OX=9606 GN=IGKV2D-24 PE=5 SV=1	0.129264902	1
P60709	Actin, cytoplasmic 1 OS=Homo sapiens OX=9606 GN=ACTB PE=1 SV=1	-2.326679697	1
Q9HAV7	GrpE protein homolog 1, mitochondrial OS=Homo sapiens OX=9606 GN=GRPEL1 PE=1 SV=2	-0.567218617	1
Q9UDY4	DnaJ homolog subfamily B member 4 OS=Homo sapiens OX=9606 GN=DNAJB4 PE=1 SV=1	0.665628034	1
O43768-4	Isoform 4 of Alpha-endosulfine OS=Homo sapiens OX=9606 GN=ENSA	0.260609	1
P09417	Dihydropteridine reductase OS=Homo sapiens OX=9606 GN=QDPR PE=1 SV=2	-1.15730766	1
P14625	Endoplasmin OS=Homo sapiens OX=9606 GN=HSP90B1 PE=1 SV=1	-1.676992861	1
P37840	Alpha-synuclein OS=Homo sapiens OX=9606 GN=SNCA PE=1 SV=1	-2.045285813	1
Q14247	Src substrate cortactin OS=Homo sapiens OX=9606 GN=CTTN PE=1 SV=2	-1.470634969	1
P54136	Arginine--tRNA ligase, cytoplasmic OS=Homo sapiens OX=9606 GN=RARS PE=1 SV=2	0.468987632	1
P03915	NADH-ubiquinone oxidoreductase chain 5 OS=Homo sapiens OX=9606 GN=MT-ND5 PE=1 SV=2	-0.946980947	1
Q9UDW1	Cytochrome b-c1 complex subunit 9 OS=Homo sapiens OX=9606 GN=UQCR10 PE=1 SV=3	0.36854223	1
Q99829	Copine-1 OS=Homo sapiens OX=9606 GN=CPNE1 PE=1 SV=1	1.217926496	1

Q02978	Mitochondrial 2-oxoglutarate/malate carrier protein OS=Homo sapiens OX=9606 GN=SLC25A11 PE=1 SV=3	-0.896887528	1
A5D6W6	Fat storage-inducing transmembrane protein 1 OS=Homo sapiens OX=9606 GN=FITM1 PE=2 SV=1	-0.102360876	1
Q9UBW8	COP9 signalosome complex subunit 7a OS=Homo sapiens OX=9606 GN=COPS7A PE=1 SV=1	-0.522034974	1
P50993	Sodium/potassium-transporting ATPase subunit alpha-2 OS=Homo sapiens OX=9606 GN=ATP1A2 PE=1 SV=1	0.5748884	1
P27695	DNA-(apurinic or apyrimidinic site) lyase OS=Homo sapiens OX=9606 GN=APEX1 PE=1 SV=2	1.297434896	1
Q13510-2	Isoform 2 of Acid ceramidase OS=Homo sapiens OX=9606 GN=ASAH1	0.666702392	1
P02749	Beta-2-glycoprotein 1 OS=Homo sapiens OX=9606 GN=APOH PE=1 SV=3	0.606649989	1
Q9H6F2	Trimeric intracellular cation channel type A OS=Homo sapiens OX=9606 GN=TMEM38A PE=1 SV=1	0.620144967	1
Q6DKJ4	Nucleoredoxin OS=Homo sapiens OX=9606 GN=NXN PE=1 SV=2	0.091757819	1
P68371	Tubulin beta-4B chain OS=Homo sapiens OX=9606 GN=TUBB4B PE=1 SV=1	-0.857191985	1
O00233	26S proteasome non-ATPase regulatory subunit 9 OS=Homo sapiens OX=9606 GN=PSMD9 PE=1 SV=3	0.00278552	1
Q96HC4-3	Isoform 3 of PDZ and LIM domain protein 5 OS=Homo sapiens OX=9606 GN=PDLIM5	-2.136452306	1
P39059	Collagen alpha-1(XV) chain OS=Homo sapiens OX=9606 GN=COL15A1 PE=1 SV=2	-1.431728626	1
P45974	Ubiquitin carboxyl-terminal hydrolase 5 OS=Homo sapiens OX=9606 GN=USP5 PE=1 SV=2	-1.582498609	1
P05023	Sodium/potassium-transporting ATPase subunit alpha-1 OS=Homo sapiens OX=9606 GN=ATP1A1 PE=1 SV=1	-0.486174044	1
Q9BSE5	Agmatinase, mitochondrial OS=Homo sapiens OX=9606 GN=AGMAT PE=1 SV=2	-0.216110133	1
Q13310-3	Isoform 3 of Polyadenylate-binding protein 4 OS=Homo sapiens OX=9606 GN=PABPC4	0.710933174	1
O60749	Sorting nexin-2 OS=Homo sapiens OX=9606 GN=SNX2 PE=1 SV=2	0.632447256	1
Q96RQ3	Methylcrotonoyl-CoA carboxylase subunit alpha, mitochondrial OS=Homo sapiens OX=9606 GN=MCCC1 PE=1 SV=3	-0.413710882	1
Q0ZGT2-2	Isoform 2 of Nexilin OS=Homo sapiens OX=9606 GN=NEXN	-1.630974034	1
P14735	Insulin-degrading enzyme OS=Homo sapiens OX=9606 GN=IDE PE=1 SV=4	0.8379379	1
Q92841	Probable ATP-dependent RNA helicase DDX17 OS=Homo sapiens OX=9606 GN=DDX17 PE=1 SV=2	0.526948706	1

Q9Y6G9	Cytoplasmic dynein 1 light intermediate chain 1 OS=Homo sapiens OX=9606 GN=DYNC1LI1 PE=1 SV=3	-0.390769714	1
Q9BS40	Latexin OS=Homo sapiens OX=9606 GN=LXN PE=1 SV=2	-0.148957866	1
Q96MF6	Coenzyme Q-binding protein COQ10 homolog A, mitochondrial OS=Homo sapiens OX=9606 GN=COQ10A PE=2 SV=2	1.761686208	1
P51648-2	Isoform 2 of Aldehyde dehydrogenase family 3 member A2 OS=Homo sapiens OX=9606 GN=ALDH3A2	1.166262931	1
Q8NHH9	Atlastin-2 OS=Homo sapiens OX=9606 GN=ATL2 PE=1 SV=2	-0.2013267	1
P20962	Parathymosin OS=Homo sapiens OX=9606 GN=PTMS PE=1 SV=2	-0.517649626	1
Q96KP4	Cytosolic non-specific dipeptidase OS=Homo sapiens OX=9606 GN=CNDP2 PE=1 SV=2	2.126084406	1
Q9BSJ8-2	Isoform 2 of Extended synaptotagmin-1 OS=Homo sapiens OX=9606 GN=ESYT1	-1.199165018	1
Q13347	Eukaryotic translation initiation factor 3 subunit I OS=Homo sapiens OX=9606 GN=EIF3I PE=1 SV=1	-0.284662539	1
P61019	Ras-related protein Rab-2A OS=Homo sapiens OX=9606 GN=RAB2A PE=1 SV=1	-2.154576228	1
P56211	cAMP-regulated phosphoprotein 19 OS=Homo sapiens OX=9606 GN=ARPP19 PE=1 SV=2	0.192381349	1
Q14103	Heterogeneous nuclear ribonucleoprotein D0 OS=Homo sapiens OX=9606 GN=HNRNPD PE=1 SV=1	1.341636513	1
P98095-2	Isoform 2 of Fibulin-2 OS=Homo sapiens OX=9606 GN=FBLN2	1.96228362	1
P35268	60S ribosomal protein L22 OS=Homo sapiens OX=9606 GN=RPL22 PE=1 SV=2	1.636370408	1
P08311	Cathepsin G OS=Homo sapiens OX=9606 GN=CTSG PE=1 SV=2	1.306467191	1
O95182	NADH dehydrogenase [ubiquinone] 1 alpha subcomplex subunit 7 OS=Homo sapiens OX=9606 GN=NDUFA7 PE=1 SV=3	-1.413274407	1
O14979	Heterogeneous nuclear ribonucleoprotein D-like OS=Homo sapiens OX=9606 GN=HNRNPDL PE=1 SV=3	-1.358053559	1
Q9UK45	U6 snRNA-associated Sm-like protein LSm7 OS=Homo sapiens OX=9606 GN=LSM7 PE=1 SV=1	-0.353627239	1
P47813	Eukaryotic translation initiation factor 1A, X-chromosomal OS=Homo sapiens OX=9606 GN=EIF1AX PE=1 SV=2	-0.338996131	1
Q9UK32	Ribosomal protein S6 kinase alpha-6 OS=Homo sapiens OX=9606 GN=RPS6KA6 PE=1 SV=1	-0.029966279	1
P50570	Dynamin-2 OS=Homo sapiens OX=9606 GN=DNM2 PE=1 SV=2	1.427862688	1
Q9UKV8	Protein argonaute-2 OS=Homo sapiens OX=9606 GN=AGO2 PE=1 SV=3	0.069261594	1

O95168	NADH dehydrogenase [ubiquinone] 1 beta subcomplex subunit 4 OS=Homo sapiens OX=9606 GN=NDUFB4 PE=1 SV=3	1.074832308	1
Q9NR12-6	Isoform 6 of PDZ and LIM domain protein 7 OS=Homo sapiens OX=9606 GN=PDLIM7	0.700887874	1
O00299	Chloride intracellular channel protein 1 OS=Homo sapiens OX=9606 GN=CLIC1 PE=1 SV=4	-1.476124988	1
Q14BN4-8	Isoform 8 of Sarcolemmal membrane-associated protein OS=Homo sapiens OX=9606 GN=SLMAP	0.310561744	1
P0C0S5	Histone H2A.Z OS=Homo sapiens OX=9606 GN=H2AZ1 PE=1 SV=2	-0.130294165	1
P07305	Histone H1.0 OS=Homo sapiens OX=9606 GN=H1-0 PE=1 SV=3	-0.40146583	1
Q99873	Protein arginine N-methyltransferase 1 OS=Homo sapiens OX=9606 GN=PRMT1 PE=1 SV=3	1.002846022	1
Q9H4A4	Aminopeptidase B OS=Homo sapiens OX=9606 GN=RNPEP PE=1 SV=2	-0.262056057	1
P24844	Myosin regulatory light polypeptide 9 OS=Homo sapiens OX=9606 GN=MYL9 PE=1 SV=4	1.484690418	1
Q92616	eIF-2-alpha kinase activator GCN1 OS=Homo sapiens OX=9606 GN=GCN1 PE=1 SV=6	-0.028598252	1
O15144	Actin-related protein 2/3 complex subunit 2 OS=Homo sapiens OX=9606 GN=ARPC2 PE=1 SV=1	0.864200203	1
Q9UBX5	Fibulin-5 OS=Homo sapiens OX=9606 GN=FBLN5 PE=1 SV=1	1.437186612	1
Q13554	Calcium/calmodulin-dependent protein kinase type II subunit beta OS=Homo sapiens OX=9606 GN=CAMK2B PE=1 SV=3	0.12086804	1
P13535	Myosin-8 OS=Homo sapiens OX=9606 GN=MYH8 PE=1 SV=3	1.125432279	1
O75436	Vacuolar protein sorting-associated protein 26A OS=Homo sapiens OX=9606 GN=VPS26A PE=1 SV=2	0.035480278	1
Q9Y5Z4	Heme-binding protein 2 OS=Homo sapiens OX=9606 GN=HEBP2 PE=1 SV=1	-0.644436449	1
A8MZF0	Proline-rich protein 33 OS=Homo sapiens OX=9606 GN=PRR33 PE=4 SV=2	-0.456274935	1
P61758	Prefoldin subunit 3 OS=Homo sapiens OX=9606 GN=VBP1 PE=1 SV=4	-0.519793162	1
Q9NX40	OCIA domain-containing protein 1 OS=Homo sapiens OX=9606 GN=OCIAD1 PE=1 SV=1	-0.21330061	1
P61106	Ras-related protein Rab-14 OS=Homo sapiens OX=9606 GN=RAB14 PE=1 SV=4	-0.310927983	1
Q86VF2-5	Isoform 5 of Immunoglobulin-like and fibronectin type III domain-containing protein 1 OS=Homo sapiens OX=9606 GN=IGFN1	-0.876007659	1
P55010	Eukaryotic translation initiation factor 5 OS=Homo sapiens OX=9606 GN=EIF5 PE=1 SV=2	-0.500257575	1

Q14444	Caprin-1 OS=Homo sapiens OX=9606 GN=CAPRIN1 PE=1 SV=2	1.811285009	1
Q9BZL1	Ubiquitin-like protein 5 OS=Homo sapiens OX=9606 GN=UBL5 PE=1 SV=1	0.671789048	1
Q93008	Probable ubiquitin carboxyl-terminal hydrolase FAF-X OS=Homo sapiens OX=9606 GN=USP9X PE=1 SV=3	-0.110919541	1
P09874	Poly [ADP-ribose] polymerase 1 OS=Homo sapiens OX=9606 GN=PARP1 PE=1 SV=4	-0.210265201	1
Q96FV2	Secernin-2 OS=Homo sapiens OX=9606 GN=SCRN2 PE=1 SV=3	1.534666154	1
Q9HA64	Ketosamine-3-kinase OS=Homo sapiens OX=9606 GN=FN3KRP PE=1 SV=2	-0.19140989	1
Q9UKU7	Isobutyryl-CoA dehydrogenase, mitochondrial OS=Homo sapiens OX=9606 GN=ACAD8 PE=1 SV=1	0.658029521	1
Q13522	Protein phosphatase 1 regulatory subunit 1A OS=Homo sapiens OX=9606 GN=PPP1R1A PE=1 SV=2	0.716301872	1
P37802-2	Isoform 2 of Transgelin-2 OS=Homo sapiens OX=9606 GN=TAGLN2	0.66687926	1
P62888	60S ribosomal protein L30 OS=Homo sapiens OX=9606 GN=RPL30 PE=1 SV=2	1.130792374	1
P01742	Immunoglobulin heavy variable 1-69 OS=Homo sapiens OX=9606 GN=IGHV1-69 PE=1 SV=2	-0.460872033	1
P62847-4	Isoform 4 of 40S ribosomal protein S24 OS=Homo sapiens OX=9606 GN=RPS24	0.572711128	1
Q6QEF8	Coronin-6 OS=Homo sapiens OX=9606 GN=CORO6 PE=1 SV=2	-0.591116809	1
P08779	Keratin, type I cytoskeletal 16 OS=Homo sapiens OX=9606 GN=KRT16 PE=1 SV=4	-0.20143569	1
P50502	Hsc70-interacting protein OS=Homo sapiens OX=9606 GN=ST13 PE=1 SV=2	-0.628306406	1
P17980	26S proteasome regulatory subunit 6A OS=Homo sapiens OX=9606 GN=PSMC3 PE=1 SV=3	-0.832094248	1
P60033	CD81 antigen OS=Homo sapiens OX=9606 GN=CD81 PE=1 SV=1	1.564874543	1
P14866	Heterogeneous nuclear ribonucleoprotein L OS=Homo sapiens OX=9606 GN=HNRNPL PE=1 SV=2	0.876084048	1
Q15181	Inorganic pyrophosphatase OS=Homo sapiens OX=9606 GN=PPA1 PE=1 SV=2	-0.978914196	1
Q7Z434	Mitochondrial antiviral-signaling protein OS=Homo sapiens OX=9606 GN=MAVS PE=1 SV=2	0.881389568	1
P52788	Spermine synthase OS=Homo sapiens OX=9606 GN=SMS PE=1 SV=2	-0.102744095	1
Q06432	Voltage-dependent calcium channel gamma-1 subunit OS=Homo sapiens OX=9606 GN=CACNG1 PE=1 SV=1	-1.154535596	1
Q92629-2	Isoform 2 of Delta-sarcoglycan OS=Homo sapiens OX=9606 GN=SGCD	-1.520463553	1

Q53GQ0	Very-long-chain 3-oxoacyl-CoA reductase OS=Homo sapiens OX=9606 GN=HSD17B12 PE=1 SV=2	0.495458401	1
Q13976	cGMP-dependent protein kinase 1 OS=Homo sapiens OX=9606 GN=PRKG1 PE=1 SV=3	1.076999924	1
O75438-2	Isoform 2 of NADH dehydrogenase [ubiquinone] 1 beta subcomplex subunit 1 OS=Homo sapiens OX=9606 GN=NDUFB1	-0.838982318	1
Q9NR31	GTP-binding protein SAR1a OS=Homo sapiens OX=9606 GN=SAR1A PE=1 SV=1	-0.60075647	1
P02649	Apolipoprotein E OS=Homo sapiens OX=9606 GN=APOE PE=1 SV=1	-0.145004392	1
Q6IQ22	Ras-related protein Rab-12 OS=Homo sapiens OX=9606 GN=RAB12 PE=1 SV=3	-0.294622906	1
Q14BN4	Sarcolemmal membrane-associated protein OS=Homo sapiens OX=9606 GN=SLMAP PE=1 SV=1	-0.610638672	1
P60842	Eukaryotic initiation factor 4A-I OS=Homo sapiens OX=9606 GN=EIF4A1 PE=1 SV=1	0.817184279	1
O95571	Persulfide dioxygenase ETHE1, mitochondrial OS=Homo sapiens OX=9606 GN=ETHE1 PE=1 SV=2	-0.283406492	1
O14639	Actin-binding LIM protein 1 OS=Homo sapiens OX=9606 GN=ABLIM1 PE=1 SV=3	0.830535199	1
P01700	Immunoglobulin lambda variable 1-47 OS=Homo sapiens OX=9606 GN=IGLV1-47 PE=1 SV=2	-0.198954105	1
P63010-2	Isoform 2 of AP-2 complex subunit beta OS=Homo sapiens OX=9606 GN=AP2B1	0.030692955	1
Q9NPJ3	Acyl-coenzyme A thioesterase 13 OS=Homo sapiens OX=9606 GN=ACOT13 PE=1 SV=1	-0.579522941	1
P35606	Coatomer subunit beta' OS=Homo sapiens OX=9606 GN=COPB2 PE=1 SV=2	-0.179959926	1
Q9GZP4	PITH domain-containing protein 1 OS=Homo sapiens OX=9606 GN=PITHD1 PE=1 SV=1	-0.040111317	1
Q14697-2	Isoform 2 of Neutral alpha-glucosidase AB OS=Homo sapiens OX=9606 GN=GANAB	1.005861573	1
P62826	GTP-binding nuclear protein Ran OS=Homo sapiens OX=9606 GN=RAN PE=1 SV=3	0.672442109	1
P19827	Inter-alpha-trypsin inhibitor heavy chain H1 OS=Homo sapiens OX=9606 GN=ITIH1 PE=1 SV=3	1.342129044	1
O14949	Cytochrome b-c1 complex subunit 8 OS=Homo sapiens OX=9606 GN=UQCRQ PE=1 SV=4	-0.703090073	1
Q2TAL5	Smoothelin-like protein 2 OS=Homo sapiens OX=9606 GN=SMTNL2 PE=2 SV=1	0.927383948	1
Q15046-2	Isoform Mitochondrial of Lysine--tRNA ligase OS=Homo sapiens OX=9606 GN=KARS1	-1.021498929	1
O00487	26S proteasome non-ATPase regulatory subunit 14 OS=Homo sapiens OX=9606 GN=PSMD14 PE=1 SV=1	1.151934566	1
P52888	Thimet oligopeptidase OS=Homo sapiens OX=9606 GN=THOP1 PE=1 SV=2	-0.565842445	1

P48059-3	Isoform 3 of LIM and senescent cell antigen-like-containing domain protein 1 OS=Homo sapiens OX=9606 GN=LIMS1	0.123786418	1
P69892	Hemoglobin subunit gamma-2 OS=Homo sapiens OX=9606 GN=HBG2 PE=1 SV=2	-0.840605622	1
Q14847	LIM and SH3 domain protein 1 OS=Homo sapiens OX=9606 GN=LASP1 PE=1 SV=2	-0.862597772	1
Q13586	Stromal interaction molecule 1 OS=Homo sapiens OX=9606 GN=STIM1 PE=1 SV=3	1.43360819	1
O00231-2	Isoform 2 of 26S proteasome non-ATPase regulatory subunit 11 OS=Homo sapiens OX=9606 GN=PSMD11	0.045738075	1
P52907	F-actin-capping protein subunit alpha-1 OS=Homo sapiens OX=9606 GN=CAPZA1 PE=1 SV=3	0.950965665	1
P11171	Protein 4.1 OS=Homo sapiens OX=9606 GN=EPB41 PE=1 SV=4	-0.920619408	1
P06899	Histone H2B type 1-J OS=Homo sapiens OX=9606 GN=H2BC11 PE=1 SV=3	-0.528333093	1
P02100	Hemoglobin subunit epsilon OS=Homo sapiens OX=9606 GN=HBE1 PE=1 SV=2	-1.420343688	1
Q15555	Microtubule-associated protein RP/EB family member 2 OS=Homo sapiens OX=9606 GN=MAPRE2 PE=1 SV=1	0.939693803	1
P37108	Signal recognition particle 14 kDa protein OS=Homo sapiens OX=9606 GN=SRP14 PE=1 SV=2	-0.916467112	1
P00747	Plasminogen OS=Homo sapiens OX=9606 GN=PLG PE=1 SV=2	0.828436469	1
Q93034	Cullin-5 OS=Homo sapiens OX=9606 GN=CUL5 PE=1 SV=4	-1.111251327	1
O60925	Prefoldin subunit 1 OS=Homo sapiens OX=9606 GN=PFDN1 PE=1 SV=2	0.963381437	1
P10636-9	Isoform Tau-G of Microtubule-associated protein tau OS=Homo sapiens OX=9606 GN=MAPT	0.835077277	1
Q15661	Tryptase alpha/beta-1 OS=Homo sapiens OX=9606 GN=TPSAB1 PE=1 SV=1	-0.738931541	1
Q9UBQ7	Glyoxylate reductase/hydroxypyruvate reductase OS=Homo sapiens OX=9606 GN=GRHPR PE=1 SV=1	-0.638380258	1
Q13425	Beta-2-syntrophin OS=Homo sapiens OX=9606 GN=SNTB2 PE=1 SV=1	1.141154131	1
P27338	Amine oxidase [flavin-containing] B OS=Homo sapiens OX=9606 GN=MAOB PE=1 SV=3	-0.752861181	1
Q9NY33	Dipeptidyl peptidase 3 OS=Homo sapiens OX=9606 GN=DPP3 PE=1 SV=2	-0.556485446	1
Q9UHY7	Enolase-phosphatase E1 OS=Homo sapiens OX=9606 GN=ENOPH1 PE=1 SV=1	0.075949324	1
P32969	60S ribosomal protein L9 OS=Homo sapiens OX=9606 GN=RPL9 PE=1 SV=1	-0.951265253	1
P63220	40S ribosomal protein S21 OS=Homo sapiens OX=9606 GN=RPS21 PE=1 SV=1	0.515458051	1

P61769	Beta-2-microglobulin OS=Homo sapiens OX=9606 GN=B2M PE=1 SV=1	1.901342209	1
P41252	Isoleucine--tRNA ligase, cytoplasmic OS=Homo sapiens OX=9606 GN=IARS PE=1 SV=2	-0.60441857	1
P05386	60S acidic ribosomal protein P1 OS=Homo sapiens OX=9606 GN=RPLP1 PE=1 SV=1	0.683157113	1
P05141	ADP/ATP translocase 2 OS=Homo sapiens OX=9606 GN=SLC25A5 PE=1 SV=7	4.17686985	1
Q08211	ATP-dependent RNA helicase A OS=Homo sapiens OX=9606 GN=DHX9 PE=1 SV=4	1.588829439	1
P51572-2	Isoform 2 of B-cell receptor-associated protein 31 OS=Homo sapiens OX=9606 GN=BCAP31	0.615157693	1
P61026	Ras-related protein Rab-10 OS=Homo sapiens OX=9606 GN=RAB10 PE=1 SV=1	-1.103615436	1
P07585	Decorin OS=Homo sapiens OX=9606 GN=DCN PE=1 SV=1	-1.023246507	1
P08697	Alpha-2-antiplasmin OS=Homo sapiens OX=9606 GN=SERPINF2 PE=1 SV=3	-0.805842256	1
P42167	Lamina-associated polypeptide 2, isoforms beta/gamma OS=Homo sapiens OX=9606 GN=TMPO PE=1 SV=2	0.861663741	1
P20618	Proteasome subunit beta type-1 OS=Homo sapiens OX=9606 GN=PSMB1 PE=1 SV=2	0.554449012	1
P60900	Proteasome subunit alpha type-6 OS=Homo sapiens OX=9606 GN=PSMA6 PE=1 SV=1	-0.986653046	1
P01009	Alpha-1-antitrypsin OS=Homo sapiens OX=9606 GN=SERPINA1 PE=1 SV=3	0.772877388	1
P27797	Calreticulin OS=Homo sapiens OX=9606 GN=CALR PE=1 SV=1	-0.469761553	1
Q06323-2	Isoform 2 of Proteasome activator complex subunit 1 OS=Homo sapiens OX=9606 GN=PSME1	-0.720369519	1
B5ME19	Eukaryotic translation initiation factor 3 subunit C-like protein OS=Homo sapiens OX=9606 GN=EIF3CL PE=1 SV=1	0.656333708	1
P40429	60S ribosomal protein L13a OS=Homo sapiens OX=9606 GN=RPL13A PE=1 SV=2	0.973226284	1
Q14974	Importin subunit beta-1 OS=Homo sapiens OX=9606 GN=KPNB1 PE=1 SV=2	-0.526017263	1
O75821	Eukaryotic translation initiation factor 3 subunit G OS=Homo sapiens OX=9606 GN=EIF3G PE=1 SV=2	-0.881270974	1
Q86SX6	Glutaredoxin-related protein 5, mitochondrial OS=Homo sapiens OX=9606 GN=GLRX5 PE=1 SV=2	-1.448722685	1
P84103	Serine/arginine-rich splicing factor 3 OS=Homo sapiens OX=9606 GN=SRSF3 PE=1 SV=1	-0.561744706	1
Q13404-1	Isoform 1 of Ubiquitin-conjugating enzyme E2 variant 1 OS=Homo sapiens OX=9606 GN=UBE2V1	-1.064396604	1

Q15582	Transforming growth factor-beta-induced protein ig-h3 OS=Homo sapiens OX=9606 GN=TGFBI PE=1 SV=1	-0.697796953	1
P45954	Short/branched chain specific acyl-CoA dehydrogenase, mitochondrial OS=Homo sapiens OX=9606 GN=ACADSB PE=1 SV=1	1.048276077	1
Q07020	60S ribosomal protein L18 OS=Homo sapiens OX=9606 GN=RPL18 PE=1 SV=2	-0.826134352	1
P56539	Caveolin-3 OS=Homo sapiens OX=9606 GN=CAV3 PE=1 SV=1	0.31054677	1
P00390	Glutathione reductase, mitochondrial OS=Homo sapiens OX=9606 GN=GSR PE=1 SV=2	0.842860881	1
O43765	Small glutamine-rich tetratricopeptide repeat-containing protein alpha OS=Homo sapiens OX=9606 GN=SGTA PE=1 SV=1	-0.511364857	1
Q9HBI1-2	Isoform 2 of Beta-parvin OS=Homo sapiens OX=9606 GN=PARVB	-0.849849077	1
P21810	Biglycan OS=Homo sapiens OX=9606 GN=BGN PE=1 SV=2	0.723835935	1
P12277	Creatine kinase B-type OS=Homo sapiens OX=9606 GN=CKB PE=1 SV=1	-0.714106812	1
O95479	GDH/6PGL endoplasmic bifunctional protein OS=Homo sapiens OX=9606 GN=H6PD PE=1 SV=2	1.550156365	1
Q07507	Dermatopontin OS=Homo sapiens OX=9606 GN=DPT PE=1 SV=2	0.563809418	1
P25189-2	Isoform L-MPZ of Myelin protein P0 OS=Homo sapiens OX=9606 GN=MPZ	1.996056329	1
Q13424	Alpha-1-syntrophin OS=Homo sapiens OX=9606 GN=SNTA1 PE=1 SV=1	-0.410452224	1
P38606	V-type proton ATPase catalytic subunit A OS=Homo sapiens OX=9606 GN=ATP6V1A PE=1 SV=2	1.380054731	1
Q00610	Clathrin heavy chain 1 OS=Homo sapiens OX=9606 GN=CLTC PE=1 SV=5	-0.849044523	1
P15088	Mast cell carboxypeptidase A OS=Homo sapiens OX=9606 GN=CPA3 PE=1 SV=2	-0.990290393	1
P18669	Phosphoglycerate mutase 1 OS=Homo sapiens OX=9606 GN=PGAM1 PE=1 SV=2	-0.771183011	1
P12109	Collagen alpha-1(VI) chain OS=Homo sapiens OX=9606 GN=COL6A1 PE=1 SV=3	-0.711789095	1
P53778	Mitogen-activated protein kinase 12 OS=Homo sapiens OX=9606 GN=MAPK12 PE=1 SV=3	-1.116479593	1
Q9Y5L4	Mitochondrial import inner membrane translocase subunit Tim13 OS=Homo sapiens OX=9606 GN=TIMM13 PE=1 SV=1	-4.54470101	1
Q9H0N5	Pterin-4-alpha-carbinolamine dehydratase 2 OS=Homo sapiens OX=9606 GN=PCBD2 PE=1 SV=4	-1.637126572	1
P11055	Myosin-3 OS=Homo sapiens OX=9606 GN=MYH3 PE=1 SV=3	1.199044537	1
P11047	Laminin subunit gamma-1 OS=Homo sapiens OX=9606 GN=LAMC1 PE=1 SV=3	-0.369996761	1

P01019	Angiotensinogen OS=Homo sapiens OX=9606 GN=AGT PE=1 SV=1	-0.94919429	1
Q15642	Cdc42-interacting protein 4 OS=Homo sapiens OX=9606 GN=TRIP10 PE=1 SV=3	-1.39112395	1
O75935	Dynactin subunit 3 OS=Homo sapiens OX=9606 GN=DCTN3 PE=1 SV=1	-1.078870533	1
P56385	ATP synthase subunit e, mitochondrial OS=Homo sapiens OX=9606 GN=ATP5ME PE=1 SV=2	-0.750245346	1
Q9UEY8	Gamma-adducin OS=Homo sapiens OX=9606 GN=ADD3 PE=1 SV=1	1.256812985	1
P07203	Glutathione peroxidase 1 OS=Homo sapiens OX=9606 GN=GPX1 PE=1 SV=4	0.617334729	1
Q8NBX0	Saccharopine dehydrogenase-like oxidoreductase OS=Homo sapiens OX=9606 GN=SCCPDH PE=1 SV=1	-0.473742578	1
P78417	Glutathione S-transferase omega-1 OS=Homo sapiens OX=9606 GN=GSTO1 PE=1 SV=2	-0.526575231	1
P22307	Non-specific lipid-transfer protein OS=Homo sapiens OX=9606 GN=SCP2 PE=1 SV=2	1.626465759	1
Q8WWV3	Reticulon-4-interacting protein 1, mitochondrial OS=Homo sapiens OX=9606 GN=RTN4IP1 PE=1 SV=2	-1.035434609	1
Q9UHV9	Prefoldin subunit 2 OS=Homo sapiens OX=9606 GN=PFDN2 PE=1 SV=1	1.68357119	1
P30837	Aldehyde dehydrogenase X, mitochondrial OS=Homo sapiens OX=9606 GN=ALDH1B1 PE=1 SV=3	0.675639663	1
Q6IAA8	Ragulator complex protein LAMTOR1 OS=Homo sapiens OX=9606 GN=LAMTOR1 PE=1 SV=2	4.221025565	1
O95563	Mitochondrial pyruvate carrier 2 OS=Homo sapiens OX=9606 GN=MPC2 PE=1 SV=1	0.538348155	1
Q8N8N7	Prostaglandin reductase 2 OS=Homo sapiens OX=9606 GN=PTGR2 PE=1 SV=1	-0.843689938	1
O60869	Endothelial differentiation-related factor 1 OS=Homo sapiens OX=9606 GN=EDF1 PE=1 SV=1	-1.087545315	1
P04196	Histidine-rich glycoprotein OS=Homo sapiens OX=9606 GN=HRG PE=1 SV=1	0.90622693	1
P50402	Emerin OS=Homo sapiens OX=9606 GN=EMD PE=1 SV=1	0.589379025	1
P62834	Ras-related protein Rap-1A OS=Homo sapiens OX=9606 GN=RAP1A PE=1 SV=1	0.684050239	1
P51911	Calponin-1 OS=Homo sapiens OX=9606 GN=CNN1 PE=1 SV=2	-1.168653218	1
Q00059	Transcription factor A, mitochondrial OS=Homo sapiens OX=9606 GN=TFAM PE=1 SV=1	-0.619716971	1
P05198	Eukaryotic translation initiation factor 2 subunit 1 OS=Homo sapiens OX=9606 GN=EIF2S1 PE=1 SV=3	-1.243099231	1
O60784	Target of Myb protein 1 OS=Homo sapiens OX=9606 GN=TOM1 PE=1 SV=2	0.531954913	1

O76003	Glutaredoxin-3 OS=Homo sapiens OX=9606 GN=GLRX3 PE=1 SV=2	-0.687154353	1
P16949-2	Isoform 2 of Stathmin OS=Homo sapiens OX=9606 GN=STMN1	1.670146728	1
P16989-2	Isoform 2 of Y-box-binding protein 3 OS=Homo sapiens OX=9606 GN=YBX3	-1.083983976	1
O15382	Branched-chain-amino-acid aminotransferase, mitochondrial OS=Homo sapiens OX=9606 GN=BCAT2 PE=1 SV=2	-1.443255269	1
P16930	Fumarylacetoacetase OS=Homo sapiens OX=9606 GN=FAH PE=1 SV=2	-1.296054038	1
Q6PI78	Transmembrane protein 65 OS=Homo sapiens OX=9606 GN=TMEM65 PE=1 SV=2	-1.148882584	1
P02753	Retinol-binding protein 4 OS=Homo sapiens OX=9606 GN=RBP4 PE=1 SV=3	-1.414366893	1
P36507	Dual specificity mitogen-activated protein kinase kinase 2 OS=Homo sapiens OX=9606 GN=MAP2K2 PE=1 SV=1	0.554594748	1
Q99436	Proteasome subunit beta type-7 OS=Homo sapiens OX=9606 GN=PSMB7 PE=1 SV=1	-0.225866762	1
P13647	Keratin, type II cytoskeletal 5 OS=Homo sapiens OX=9606 GN=KRT5 PE=1 SV=3	-2.776168296	1
Q9BSL1	Ubiquitin-associated domain-containing protein 1 OS=Homo sapiens OX=9606 GN=UBAC1 PE=1 SV=1	-1.493319581	1
Q15149-7	Isoform 7 of Plectin OS=Homo sapiens OX=9606 GN=PLEC	-1.477293161	1
Q7LBR1	Charged multivesicular body protein 1b OS=Homo sapiens OX=9606 GN=CHMP1B PE=1 SV=1	0.374064734	1
Q9H0R4	Haloacid dehalogenase-like hydrolase domain-containing protein 2 OS=Homo sapiens OX=9606 GN=HDHD2 PE=1 SV=1	-1.548072568	1
Q9UJ70-2	Isoform 2 of N-acetyl-D-glucosamine kinase OS=Homo sapiens OX=9606 GN=NAGK	2.120997573	1
Q92901	60S ribosomal protein L3-like OS=Homo sapiens OX=9606 GN=RPL3L PE=2 SV=3	0.463504468	1
Q15172	Serine/threonine-protein phosphatase 2A 56 kDa regulatory subunit alpha isoform OS=Homo sapiens OX=9606 GN=PPP2R5A PE=1 SV=1	1.496778971	1
P46020	Phosphorylase b kinase regulatory subunit alpha, skeletal muscle isoform OS=Homo sapiens OX=9606 GN=PHKA1 PE=1 SV=2	0.565289291	1
P15090	Fatty acid-binding protein, adipocyte OS=Homo sapiens OX=9606 GN=FABP4 PE=1 SV=3	0.36290751	1
Q9NR19-2	Isoform 2 of Acetyl-coenzyme A synthetase, cytoplasmic OS=Homo sapiens OX=9606 GN=ACSS2	-0.580313787	1
P43490	Nicotinamide phosphoribosyltransferase OS=Homo sapiens OX=9606 GN=NAMPT PE=1 SV=1	0.439942457	1
Q9NUQ9	Protein FAM49B OS=Homo sapiens OX=9606 GN=FAM49B PE=1 SV=1	1.93294334	1

P50454	Serpin H1 OS=Homo sapiens OX=9606 GN=SERPINH1 PE=1 SV=2	-1.245731881	1
Q92890-1	Isoform Long of Ubiquitin recognition factor in ER-associated degradation protein 1 OS=Homo sapiens OX=9606 GN=UFD1	0.402232211	1
P25789	Proteasome subunit alpha type-4 OS=Homo sapiens OX=9606 GN=PSMA4 PE=1 SV=1	0.532237925	1
P11182	Lipoamide acyltransferase component of branched-chain alpha-keto acid dehydrogenase complex, mitochondrial OS=Homo sapiens OX=9606 GN=DBT PE=1 SV=3	0.661356877	1
P61160-2	Isoform 2 of Actin-related protein 2 OS=Homo sapiens OX=9606 GN=ACTR2	0.576719096	1
O43776	Asparagine--tRNA ligase, cytoplasmic OS=Homo sapiens OX=9606 GN=NARS1 PE=1 SV=1	0.777175312	1
P59665	Neutrophil defensin 1 OS=Homo sapiens OX=9606 GN=DEFA1 PE=1 SV=1	-1.0389104	1
O95372	Acyl-protein thioesterase 2 OS=Homo sapiens OX=9606 GN=LYPLA2 PE=1 SV=1	-0.935000839	1
P09960	Leukotriene A-4 hydrolase OS=Homo sapiens OX=9606 GN=LTA4H PE=1 SV=2	0.501357269	1
P53999	Activated RNA polymerase II transcriptional coactivator p15 OS=Homo sapiens OX=9606 GN=SUB1 PE=1 SV=3	0.685406618	1
O43615	Mitochondrial import inner membrane translocase subunit TIM44 OS=Homo sapiens OX=9606 GN=TIMM44 PE=1 SV=2	-0.563572309	1
P11166	Solute carrier family 2, facilitated glucose transporter member 1 OS=Homo sapiens OX=9606 GN=SLC2A1 PE=1 SV=2	-3.304176377	1
P00167	Cytochrome b5 OS=Homo sapiens OX=9606 GN=CYB5A PE=1 SV=2	1.690385791	1
Q96FW1	Ubiquitin thioesterase OTUB1 OS=Homo sapiens OX=9606 GN=OTUB1 PE=1 SV=2	-0.297603468	1
Q8TDX7	Serine/threonine-protein kinase Nek7 OS=Homo sapiens OX=9606 GN=NEK7 PE=1 SV=1	-0.27699042	1
Q7Z406-6	Isoform 6 of Myosin-14 OS=Homo sapiens OX=9606 GN=MYH14	0.867222548	1
Q9NRX4	14 kDa phosphohistidine phosphatase OS=Homo sapiens OX=9606 GN=PHPT1 PE=1 SV=1	0.340724866	1
P04899-4	Isoform sGi2 of Guanine nucleotide-binding protein G(i) subunit alpha-2 OS=Homo sapiens OX=9606 GN=GNAI2	0.796085856	1
O75112-6	Isoform 6 of LIM domain-binding protein 3 OS=Homo sapiens OX=9606 GN=LDB3	-3.09849128	1
Q9NQR4	Omega-amidase NIT2 OS=Homo sapiens OX=9606 GN=NIT2 PE=1 SV=1	0.494729224	1
P43897	Elongation factor Ts, mitochondrial OS=Homo sapiens OX=9606 GN=TSFM PE=1 SV=2	-1.351537273	1

Q5QNW6-2	Isoform 2 of Histone H2B type 2-F OS=Homo sapiens OX=9606 GN=HIST2H2BF	0.917121346	1
P20929-2	Isoform 2 of Nebulin OS=Homo sapiens OX=9606 GN=NEB	-2.170448819	1
Q5HYK3	2-methoxy-6-polyprenyl-1,4-benzoquinol methylase, mitochondrial OS=Homo sapiens OX=9606 GN=COQ5 PE=1 SV=2	-0.556439916	1
Q01518	Adenylyl cyclase-associated protein 1 OS=Homo sapiens OX=9606 GN=CAP1 PE=1 SV=5	0.584392057	1
P24298	Alanine aminotransferase 1 OS=Homo sapiens OX=9606 GN=GPT PE=1 SV=3	0.544503568	1
P10301	Ras-related protein R-Ras OS=Homo sapiens OX=9606 GN=RRAS PE=1 SV=1	0.711757653	1
P17931	Galectin-3 OS=Homo sapiens OX=9606 GN=LGALS3 PE=1 SV=5	0.297387096	1
Q96FJ2	Dynein light chain 2, cytoplasmic OS=Homo sapiens OX=9606 GN=DYNLL2 PE=1 SV=1	0.841514884	1
O14773	Tripeptidyl-peptidase 1 OS=Homo sapiens OX=9606 GN=TPP1 PE=1 SV=2	-1.197079218	1
Q9UIJ7	GTP:AMP phosphotransferase AK3, mitochondrial OS=Homo sapiens OX=9606 GN=AK3 PE=1 SV=4	0.344584619	1
P49773	Histidine triad nucleotide-binding protein 1 OS=Homo sapiens OX=9606 GN=HINT1 PE=1 SV=2	-0.354873961	1
P49419	Alpha-aminoadipic semialdehyde dehydrogenase OS=Homo sapiens OX=9606 GN=ALDH7A1 PE=1 SV=5	0.673966117	1
P61081	NEDD8-conjugating enzyme Ubc12 OS=Homo sapiens OX=9606 GN=UBE2M PE=1 SV=1	-0.482222777	1
P61077-3	Isoform 3 of Ubiquitin-conjugating enzyme E2 D3 OS=Homo sapiens OX=9606 GN=UBE2D3	1.188981139	1
Q13098-7	Isoform 2 of COP9 signalosome complex subunit 1 OS=Homo sapiens OX=9606 GN=GPS1	0.567248818	1
O43681	ATPase ASNA1 OS=Homo sapiens OX=9606 GN=ASNA1 PE=1 SV=2	2.282663601	1
O60884	DnaJ homolog subfamily A member 2 OS=Homo sapiens OX=9606 GN=DNAJA2 PE=1 SV=1	-0.526834405	1
Q99426	Tubulin-folding cofactor B OS=Homo sapiens OX=9606 GN=TBCB PE=1 SV=2	0.984675531	1
Q14624	Inter-alpha-trypsin inhibitor heavy chain H4 OS=Homo sapiens OX=9606 GN=ITIH4 PE=1 SV=4	-0.534645733	1
P35542	Serum amyloid A-4 protein OS=Homo sapiens OX=9606 GN=SAA4 PE=1 SV=2	0.580950669	1
P27348	14-3-3 protein theta OS=Homo sapiens OX=9606 GN=YWHAQ PE=1 SV=1	-0.508139583	1
P35914	Hydroxymethylglutaryl-CoA lyase, mitochondrial OS=Homo sapiens OX=9606 GN=HMGCL PE=1 SV=2	2.218084173	1

P13716-2	Isoform 2 of Delta-aminolevulinic acid dehydratase OS=Homo sapiens OX=9606 GN=ALAD	1.951646207	1
P02549	Spectrin alpha chain, erythrocytic 1 OS=Homo sapiens OX=9606 GN=SPTA1 PE=1 SV=5	-1.33153066	1
P16070	CD44 antigen OS=Homo sapiens OX=9606 GN=CD44 PE=1 SV=3	0.6549844	1
Q9H0E2	Toll-interacting protein OS=Homo sapiens OX=9606 GN=TOLLIP PE=1 SV=1	1.932249658	1
P09488	Glutathione S-transferase Mu 1 OS=Homo sapiens OX=9606 GN=GSTM1 PE=1 SV=3	1.657227902	1
P16157-3	Isoform Er5 of Ankyrin-1 OS=Homo sapiens OX=9606 GN=ANK1	1.132461905	1
O00505	Importin subunit alpha-4 OS=Homo sapiens OX=9606 GN=KPNA3 PE=1 SV=2	-0.601541551	1
Q9UHD8	Septin-9 OS=Homo sapiens OX=9606 GN=SEPTIN9 PE=1 SV=2	0.540873991	1
P36269-3	Isoform 3 of Glutathione hydrolase 5 proenzyme OS=Homo sapiens OX=9606 GN=GGT5	1.831711965	1
Q14254	Flotillin-2 OS=Homo sapiens OX=9606 GN=FLOT2 PE=1 SV=2	-0.877175506	1
Q15366-3	Isoform 3 of Poly(rC)-binding protein 2 OS=Homo sapiens OX=9606 GN=PCBP2	-1.708099415	1
O14841	5-oxoprolinase OS=Homo sapiens OX=9606 GN=OPLAH PE=1 SV=3	-1.647435333	1
P13667	Protein disulfide-isomerase A4 OS=Homo sapiens OX=9606 GN=PDIA4 PE=1 SV=2	-0.48611275	1
Q9Y3I0	RNA-splicing ligase RtcB homolog OS=Homo sapiens OX=9606 GN=RTCB PE=1 SV=1	-0.347638617	1
O75368	SH3 domain-binding glutamic acid-rich-like protein OS=Homo sapiens OX=9606 GN=SH3BGRL PE=1 SV=1	0.775342061	1
P07858	Cathepsin B OS=Homo sapiens OX=9606 GN=CTSB PE=1 SV=3	-0.791924241	1
O75964	ATP synthase subunit g, mitochondrial OS=Homo sapiens OX=9606 GN=ATP5MG PE=1 SV=3	0.3282386	1
P08123	Collagen alpha-2(I) chain OS=Homo sapiens OX=9606 GN=COL1A2 PE=1 SV=7	-0.70484769	1
P43121	Cell surface glycoprotein MUC18 OS=Homo sapiens OX=9606 GN=MCAM PE=1 SV=2	2.395509499	1
P30533	Alpha-2-macroglobulin receptor-associated protein OS=Homo sapiens OX=9606 GN=LRPAP1 PE=1 SV=1	0.69450458	1
P02654	Apolipoprotein C-I OS=Homo sapiens OX=9606 GN=APOC1 PE=1 SV=1	0.668714439	1
P60903	Protein S100-A10 OS=Homo sapiens OX=9606 GN=S100A10 PE=1 SV=2	-0.818425403	1
P84090	Enhancer of rudimentary homolog OS=Homo sapiens OX=9606 GN=ERH PE=1 SV=1	-0.481776045	1
O43399-7	Isoform 7 of Tumor protein D54 OS=Homo sapiens OX=9606 GN=TPD52L2	0.351883174	1

P08294	Extracellular superoxide dismutase [Cu-Zn] OS=Homo sapiens OX=9606 GN=SOD3 PE=1 SV=2	2.684928828	1
Q8WWM9	Cytoglobin OS=Homo sapiens OX=9606 GN=CYGB PE=1 SV=1	1.999616883	1
P06702	Protein S100-A9 OS=Homo sapiens OX=9606 GN=S100A9 PE=1 SV=1	0.62030658	1
P13987-2	Isoform 2 of CD59 glycoprotein OS=Homo sapiens OX=9606 GN=CD59	-0.259390031	1
P62633-6	Isoform 6 of Cellular nucleic acid-binding protein OS=Homo sapiens OX=9606 GN=CNBP	-2.208538392	1
O94760	N(G),N(G)-dimethylarginine dimethylaminohydrolase 1 OS=Homo sapiens OX=9606 GN=DDAH1 PE=1 SV=3	-0.268123259	1
P36543	V-type proton ATPase subunit E 1 OS=Homo sapiens OX=9606 GN=ATP6V1E1 PE=1 SV=1	2.345864555	1
P62861	40S ribosomal protein S30 OS=Homo sapiens OX=9606 GN=FAU PE=1 SV=1	-1.710157384	1
P39023	60S ribosomal protein L3 OS=Homo sapiens OX=9606 GN=RPL3 PE=1 SV=2	2.08263952	1
P06737	Glycogen phosphorylase, liver form OS=Homo sapiens OX=9606 GN=PYGL PE=1 SV=4	-2.162522015	1
Q9NP79	Vacuolar protein sorting-associated protein VTA1 homolog OS=Homo sapiens OX=9606 GN=VTA1 PE=1 SV=1	2.261910363	1
O75131	Copine-3 OS=Homo sapiens OX=9606 GN=CPNE3 PE=1 SV=1	-0.469382832	1
P35754	Glutaredoxin-1 OS=Homo sapiens OX=9606 GN=GLRX PE=1 SV=2	0.258068362	1
Q00577	Transcriptional activator protein Pur-alpha OS=Homo sapiens OX=9606 GN=PURA PE=1 SV=2	-0.40210955	1
Q8WXH0-2	Isoform 2 of Nesprin-2 OS=Homo sapiens OX=9606 GN=SYNE2	0.953584474	1
P46776	60S ribosomal protein L27a OS=Homo sapiens OX=9606 GN=RPL27A PE=1 SV=2	2.310077963	1
Q8TCJ2	Dolichyl-diphosphooligosaccharide--protein glycosyltransferase subunit STT3B OS=Homo sapiens OX=9606 GN=STT3B PE=1 SV=1	5.287670481	1
P00367	Glutamate dehydrogenase 1, mitochondrial OS=Homo sapiens OX=9606 GN=GLUD1 PE=1 SV=2	0.248304554	1
P59998-3	Isoform 3 of Actin-related protein 2/3 complex subunit 4 OS=Homo sapiens OX=9606 GN=ARPC4	2.040739459	1
P21964	Catechol O-methyltransferase OS=Homo sapiens OX=9606 GN=COMT PE=1 SV=2	0.496063572	1
Q96CT7	Coiled-coil domain-containing protein 124 OS=Homo sapiens OX=9606 GN=CCDC124 PE=1 SV=1	-1.711110642	1

O60504	Vinexin OS=Homo sapiens OX=9606 GN=SORBS3 PE=1 SV=2	0.661342217	1
Q9Y6C9	Mitochondrial carrier homolog 2 OS=Homo sapiens OX=9606 GN=MTCH2 PE=1 SV=1	-2.21047926	1
Q8N3L3	Beta-taxilin OS=Homo sapiens OX=9606 GN=TXLNB PE=1 SV=3	-0.813010943	1
P42766	60S ribosomal protein L35 OS=Homo sapiens OX=9606 GN=RPL35 PE=1 SV=2	-1.919017161	1
O60936-3	Isoform 3 of Nucleolar protein 3 OS=Homo sapiens OX=9606 GN=NOL3	-0.604874692	1
P12829	Myosin light chain 4 OS=Homo sapiens OX=9606 GN=MYL4 PE=1 SV=3	1.272855217	1
O60237-6	Isoform 6 of Protein phosphatase 1 regulatory subunit 12B OS=Homo sapiens OX=9606 GN=PPP1R12B	-0.323201822	1
Q12988	Heat shock protein beta-3 OS=Homo sapiens OX=9606 GN=HSPB3 PE=1 SV=2	-0.58369562	1
P12955	Xaa-Pro dipeptidase OS=Homo sapiens OX=9606 GN=PEPD PE=1 SV=3	-1.681805638	1
P68366	Tubulin alpha-4A chain OS=Homo sapiens OX=9606 GN=TUBA4A PE=1 SV=1	0.333201608	1
Q02221	Cytochrome c oxidase subunit 6A2, mitochondrial OS=Homo sapiens OX=9606 GN=COX6A2 PE=2 SV=2	-2.651513008	1
Q9NR28	Diablo homolog, mitochondrial OS=Homo sapiens OX=9606 GN=DIABLO PE=1 SV=1	0.344339391	1
Q9Y512	Sorting and assembly machinery component 50 homolog OS=Homo sapiens OX=9606 GN=SAMM50 PE=1 SV=3	-0.686132577	1
Q13492	Phosphatidylinositol-binding clathrin assembly protein OS=Homo sapiens OX=9606 GN=PICALM PE=1 SV=2	3.082747961	1
O76076	WNT1-inducible-signaling pathway protein 2 OS=Homo sapiens OX=9606 GN=WISP2 PE=1 SV=1	1.362376557	1
P43034	Platelet-activating factor acetylhydrolase IB subunit alpha OS=Homo sapiens OX=9606 GN=PAFAH1B1 PE=1 SV=2	0.284168353	1
P01042	Kininogen-1 OS=Homo sapiens OX=9606 GN=KNG1 PE=1 SV=2	-0.576267122	1
P61916	NPC intracellular cholesterol transporter 2 OS=Homo sapiens OX=9606 GN=NPC2 PE=1 SV=1	2.874432243	1
O60739	Eukaryotic translation initiation factor 1b OS=Homo sapiens OX=9606 GN=EIF1B PE=1 SV=2	0.327606998	1
P05455	Lupus La protein OS=Homo sapiens OX=9606 GN=SSB PE=1 SV=2	0.828347169	1
P02686	Myelin basic protein OS=Homo sapiens OX=9606 GN=MBP PE=1 SV=3	1.319715364	1
Q86VN1	Vacuolar protein-sorting-associated protein 36 OS=Homo sapiens OX=9606 GN=VPS36 PE=1 SV=1	1.250850993	1

P55209	Nucleosome assembly protein 1-like 1 OS=Homo sapiens OX=9606 GN=NAP1L1 PE=1 SV=1	-0.477166222	1
P13796	Plastin-2 OS=Homo sapiens OX=9606 GN=LCP1 PE=1 SV=6	1.699765127	1
P55060	Exportin-2 OS=Homo sapiens OX=9606 GN=CSE1L PE=1 SV=3	0.378568464	1
P06730-2	Isoform 2 of Eukaryotic translation initiation factor 4E OS=Homo sapiens OX=9606 GN=EIF4E	-1.367304919	1
P49207	60S ribosomal protein L34 OS=Homo sapiens OX=9606 GN=RPL34 PE=1 SV=3	1.442084559	1
Q96A26	Protein FAM162A OS=Homo sapiens OX=9606 GN=FAM162A PE=1 SV=2	-0.453844561	1
Q13361	Microfibrillar-associated protein 5 OS=Homo sapiens OX=9606 GN=MFAP5 PE=1 SV=1	0.522149917	1
P20073	Annexin A7 OS=Homo sapiens OX=9606 GN=ANXA7 PE=1 SV=3	0.262498144	1
Q8TAE8	Growth arrest and DNA damage-inducible proteins-interacting protein 1 OS=Homo sapiens OX=9606 GN=GADD45GIP1 PE=1 SV=1	3.219595719	1
Q3SXM5	Inactive hydroxysteroid dehydrogenase-like protein 1 OS=Homo sapiens OX=9606 GN=HSDL1 PE=1 SV=3	-1.463553557	1
O14791-2	Isoform 2 of Apolipoprotein L1 OS=Homo sapiens OX=9606 GN=APOL1	-0.96334763	1
Q01130	Serine/arginine-rich splicing factor 2 OS=Homo sapiens OX=9606 GN=SRSF2 PE=1 SV=4	-1.302896726	1
Q9Y316-3	Isoform 3 of Protein MEMO1 OS=Homo sapiens OX=9606 GN=MEMO1	0.945453949	1
Q9UJY1	Heat shock protein beta-8 OS=Homo sapiens OX=9606 GN=HSPB8 PE=1 SV=1	0.66494262	1
Q13813-3	Isoform 3 of Spectrin alpha chain, non-erythrocytic 1 OS=Homo sapiens OX=9606 GN=SPTAN1	-0.275805311	1
Q8NCW5	NAD(P)H-hydrate epimerase OS=Homo sapiens OX=9606 GN=NAXE PE=1 SV=2	0.320511397	1
O14950	Myosin regulatory light chain 12B OS=Homo sapiens OX=9606 GN=MYL12B PE=1 SV=2	1.469371853	1
Q9NQX3-2	Isoform 2 of Gephyrin OS=Homo sapiens OX=9606 GN=GPHN	1.218761525	1
Q03154	Aminoacylase-1 OS=Homo sapiens OX=9606 GN=ACY1 PE=1 SV=1	-0.251363617	1
Q13683	Integrin alpha-7 OS=Homo sapiens OX=9606 GN=ITGA7 PE=1 SV=3	-0.478674486	1
Q9Y277	Voltage-dependent anion-selective channel protein 3 OS=Homo sapiens OX=9606 GN=VDAC3 PE=1 SV=1	-2.177508221	1
Q9NZL9	Methionine adenosyltransferase 2 subunit beta OS=Homo sapiens OX=9606 GN=MAT2B PE=1 SV=1	3.130393746	1

Q13526	Peptidyl-prolyl cis-trans isomerase NIMA-interacting 1 OS=Homo sapiens OX=9606 GN=PIN1 PE=1 SV=1	-0.827049834	1
P36405	ADP-ribosylation factor-like protein 3 OS=Homo sapiens OX=9606 GN=ARL3 PE=1 SV=2	-0.772798145	1
Q8WYQ3	Coiled-coil-helix-coiled-coil-helix domain-containing protein 10, mitochondrial OS=Homo sapiens OX=9606 GN=CHCHD10 PE=1 SV=1	-0.402182481	1
P07996	Thrombospondin-1 OS=Homo sapiens OX=9606 GN=THBS1 PE=1 SV=2	2.979160551	1
P18085	ADP-ribosylation factor 4 OS=Homo sapiens OX=9606 GN=ARF4 PE=1 SV=3	-0.945251331	1
P54819	Adenylate kinase 2, mitochondrial OS=Homo sapiens OX=9606 GN=AK2 PE=1 SV=2	-0.839451077	1
Q9BXS1	Isopentenyl-diphosphate delta-isomerase 2 OS=Homo sapiens OX=9606 GN=IDI2 PE=1 SV=1	-0.714012682	1
P62993	Growth factor receptor-bound protein 2 OS=Homo sapiens OX=9606 GN=GRB2 PE=1 SV=1	0.209703541	1
P54920	Alpha-soluble NSF attachment protein OS=Homo sapiens OX=9606 GN=NAPA PE=1 SV=3	-0.297127332	1
O00764	Pyridoxal kinase OS=Homo sapiens OX=9606 GN=PDXK PE=1 SV=1	0.668946198	1
Q16881	Thioredoxin reductase 1, cytoplasmic OS=Homo sapiens OX=9606 GN=TXNRD1 PE=1 SV=3	0.315544472	1
Q9H444	Charged multivesicular body protein 4b OS=Homo sapiens OX=9606 GN=CHMP4B PE=1 SV=1	-0.266164037	1
P55263	Adenosine kinase OS=Homo sapiens OX=9606 GN=ADK PE=1 SV=2	-0.890727323	1
P35611-3	Isoform 3 of Alpha-adducin OS=Homo sapiens OX=9606 GN=ADD1	1.028352152	1
Q9BXK5	Bcl-2-like protein 13 OS=Homo sapiens OX=9606 GN=BCL2L13 PE=1 SV=1	-0.412662641	1
P30622	CAP-Gly domain-containing linker protein 1 OS=Homo sapiens OX=9606 GN=CLIP1 PE=1 SV=2	-0.115890711	1
Q9BT78	COP9 signalosome complex subunit 4 OS=Homo sapiens OX=9606 GN=COPS4 PE=1 SV=1	0.417436658	1
P47756-2	Isoform 2 of F-actin-capping protein subunit beta OS=Homo sapiens OX=9606 GN=CAPZB	-0.405732221	1
Q93100	Phosphorylase b kinase regulatory subunit beta OS=Homo sapiens OX=9606 GN=PHKB PE=1 SV=3	0.211249506	1
P51665	26S proteasome non-ATPase regulatory subunit 7 OS=Homo sapiens OX=9606 GN=PSMD7 PE=1 SV=2	0.803488498	1

Q9Y2S6	Translation machinery-associated protein 7 OS=Homo sapiens OX=9606 GN=TMA7 PE=1 SV=1	-1.290561845	1
O94973-2	Isoform 2 of AP-2 complex subunit alpha-2 OS=Homo sapiens OX=9606 GN=AP2A2	0.585151019	1
Q9H4G4	Golgi-associated plant pathogenesis-related protein 1 OS=Homo sapiens OX=9606 GN=GLIPR2 PE=1 SV=3	0.416604915	1
P07919	Cytochrome b-c1 complex subunit 6, mitochondrial OS=Homo sapiens OX=9606 GN=UQCRH PE=1 SV=2	-0.228095458	1
P61254	60S ribosomal protein L26 OS=Homo sapiens OX=9606 GN=RPL26 PE=1 SV=1	0.108514895	1
P49588	Alanine--tRNA ligase, cytoplasmic OS=Homo sapiens OX=9606 GN=AARS PE=1 SV=2	0.359708956	1
O00186	Syntaxin-binding protein 3 OS=Homo sapiens OX=9606 GN=STXBP3 PE=1 SV=2	-0.550652295	1
P12110	Collagen alpha-2(VI) chain OS=Homo sapiens OX=9606 GN=COL6A2 PE=1 SV=4	0.211764737	1
P15531-2	Isoform 2 of Nucleoside diphosphate kinase A OS=Homo sapiens OX=9606 GN=NME1	0.778279631	1
P53814-6	Isoform B3 of Smoothelin OS=Homo sapiens OX=9606 GN=SMTN	3.141288878	1
Q07666	KH domain-containing, RNA-binding, signal transduction-associated protein 1 OS=Homo sapiens OX=9606 GN=KHDRBS1 PE=1 SV=1	0.671521335	1
Q5JTJ3-2	Isoform 2 of Cytochrome c oxidase assembly factor 6 homolog OS=Homo sapiens OX=9606 GN=COA6	0.228857586	1
P07099	Epoxide hydrolase 1 OS=Homo sapiens OX=9606 GN=EPHX1 PE=1 SV=1	0.218422329	1
P09497-2	Isoform Non-brain of Clathrin light chain B OS=Homo sapiens OX=9606 GN=CLTB	-0.429116868	1
P54922	[Protein ADP-ribosylarginine] hydrolase OS=Homo sapiens OX=9606 GN=ADPRH PE=1 SV=1	0.632626668	1
P62081	40S ribosomal protein S7 OS=Homo sapiens OX=9606 GN=RPS7 PE=1 SV=1	-0.450723166	1
P02775	Platelet basic protein OS=Homo sapiens OX=9606 GN=PPBP PE=1 SV=3	0.83674202	1
Q9H223	EH domain-containing protein 4 OS=Homo sapiens OX=9606 GN=EHD4 PE=1 SV=1	0.195182116	1
Q15691	Microtubule-associated protein RP/EB family member 1 OS=Homo sapiens OX=9606 GN=MAPRE1 PE=1 SV=3	0.659087694	1
Q9Y5J7	Mitochondrial import inner membrane translocase subunit Tim9 OS=Homo sapiens OX=9606 GN=TIMM9 PE=1 SV=1	-0.72927577	1
Q14011	Cold-inducible RNA-binding protein OS=Homo sapiens OX=9606 GN=CIRBP PE=1 SV=1	0.260755649	1
Q14258	E3 ubiquitin/ISG15 ligase TRIM25 OS=Homo sapiens OX=9606 GN=TRIM25 PE=1 SV=2	2.034817086	1

Q687X5	Metalloreductase STEAP4 OS=Homo sapiens OX=9606 GN=STEAP4 PE=1 SV=1	0.634215069	1
Q9HDC9	Adipocyte plasma membrane-associated protein OS=Homo sapiens OX=9606 GN=APMAP PE=1 SV=2	1.741247494	1
P54725	UV excision repair protein RAD23 homolog A OS=Homo sapiens OX=9606 GN=RAD23A PE=1 SV=1	-0.819602778	1
P68036-3	Isoform 3 of Ubiquitin-conjugating enzyme E2 L3 OS=Homo sapiens OX=9606 GN=UBE2L3	-0.293031753	1
P05165	Propionyl-CoA carboxylase alpha chain, mitochondrial OS=Homo sapiens OX=9606 GN=PCCA PE=1 SV=4	-0.419927232	1
Q02952	A-kinase anchor protein 12 OS=Homo sapiens OX=9606 GN=AKAP12 PE=1 SV=4	0.494487247	1
P55809	Succinyl-CoA:3-ketoacid coenzyme A transferase 1, mitochondrial OS=Homo sapiens OX=9606 GN=OXCT1 PE=1 SV=1	0.30956427	1
P28331-2	Isoform 2 of NADH-ubiquinone oxidoreductase 75 kDa subunit, mitochondrial OS=Homo sapiens OX=9606 GN=NDUFS1	0.199046664	1
Q6P5Q4	Leiomodin-2 OS=Homo sapiens OX=9606 GN=LMOD2 PE=1 SV=2	-0.407248715	1
P17096-3	Isoform HMG-R of High mobility group protein HMG-I/HMG-Y OS=Homo sapiens OX=9606 GN=HMGA1	-0.872197283	1
O60763-2	Isoform 2 of General vesicular transport factor p115 OS=Homo sapiens OX=9606 GN=USO1	-0.246690604	1
Q9BW30	Tubulin polymerization-promoting protein family member 3 OS=Homo sapiens OX=9606 GN=TPPP3 PE=1 SV=1	-0.160562708	1
Q9BQS8-4	Isoform 4 of FYVE and coiled-coil domain-containing protein 1 OS=Homo sapiens OX=9606 GN=FYCO1	-2.178233418	1
P28070	Proteasome subunit beta type-4 OS=Homo sapiens OX=9606 GN=PSMB4 PE=1 SV=4	-0.191614624	1
O94903	Pyridoxal phosphate homeostasis protein OS=Homo sapiens OX=9606 GN=PLPBP PE=1 SV=1	0.256404542	1
P63316	Troponin C, slow skeletal and cardiac muscles OS=Homo sapiens OX=9606 GN=TNNC1 PE=1 SV=1	-0.186039342	1
P01824	Immunoglobulin heavy variable 4-39 OS=Homo sapiens OX=9606 GN=IGHV4-39 PE=1 SV=2	-0.250228544	1
Q02750	Dual specificity mitogen-activated protein kinase kinase 1 OS=Homo sapiens OX=9606 GN=MAP2K1 PE=1 SV=2	-0.196181857	1
Q8TDC0	Myozenin-3 OS=Homo sapiens OX=9606 GN=MYOZ3 PE=1 SV=2	-0.193294155	1
P01780	Immunoglobulin heavy variable 3-7 OS=Homo sapiens OX=9606 GN=IGHV3-7 PE=1 SV=2	-0.639338066	1

P21953	2-oxoisovalerate dehydrogenase subunit beta, mitochondrial OS=Homo sapiens OX=9606 GN=BCKDHB PE=1 SV=2	-0.293928981	1
P62873	Guanine nucleotide-binding protein G(I)/G(S)/G(T) subunit beta-1 OS=Homo sapiens OX=9606 GN=GNB1 PE=1 SV=3	0.452623141	1
Q6PIU2-2	Isoform 2 of Neutral cholesterol ester hydrolase 1 OS=Homo sapiens OX=9606 GN=NCEH1	-0.19746782	1
Q02790	Peptidyl-prolyl cis-trans isomerase FKBP4 OS=Homo sapiens OX=9606 GN=FKBP4 PE=1 SV=3	-0.459010283	1
P63218	Guanine nucleotide-binding protein G(I)/G(S)/G(O) subunit gamma-5 OS=Homo sapiens OX=9606 GN=GNG5 PE=1 SV=3	-1.992279898	1
P04275	von Willebrand factor OS=Homo sapiens OX=9606 GN=VWF PE=1 SV=4	0.142519548	1
P49458	Signal recognition particle 9 kDa protein OS=Homo sapiens OX=9606 GN=SRP9 PE=1 SV=2	-0.139030566	1
A4UGR9-8	Isoform 8 of Xin actin-binding repeat-containing protein 2 OS=Homo sapiens OX=9606 GN=XIRP2	0.141295495	1
P46783	40S ribosomal protein S10 OS=Homo sapiens OX=9606 GN=RPS10 PE=1 SV=1	-0.210087143	1
Q99536	Synaptic vesicle membrane protein VAT-1 homolog OS=Homo sapiens OX=9606 GN=VAT1 PE=1 SV=2	0.086756719	1
Q9BZV1	UBX domain-containing protein 6 OS=Homo sapiens OX=9606 GN=UBXN6 PE=1 SV=1	0.100814313	1
P02679	Fibrinogen gamma chain OS=Homo sapiens OX=9606 GN=FGG PE=1 SV=3	0.126078199	1
Q9Y265	RuvB-like 1 OS=Homo sapiens OX=9606 GN=RUVBL1 PE=1 SV=1	2.942794579	1
O15372	Eukaryotic translation initiation factor 3 subunit H OS=Homo sapiens OX=9606 GN=EIF3H PE=1 SV=1	1.807235109	1
Q9Y6B6	GTP-binding protein SAR1b OS=Homo sapiens OX=9606 GN=SAR1B PE=1 SV=1	-0.206964833	1
P03928	ATP synthase protein 8 OS=Homo sapiens OX=9606 GN=MT-ATP8 PE=1 SV=1	-2.881078482	1
Q05707	Collagen alpha-1(XIV) chain OS=Homo sapiens OX=9606 GN=COL14A1 PE=1 SV=3	-0.523386638	1
P08574	Cytochrome c1, heme protein, mitochondrial OS=Homo sapiens OX=9606 GN=CYC1 PE=1 SV=3	-0.153435327	1
O75531	Barrier-to-autointegration factor OS=Homo sapiens OX=9606 GN=BANF1 PE=1 SV=1	0.162552927	1
Q92905	COP9 signalosome complex subunit 5 OS=Homo sapiens OX=9606 GN=COPS5 PE=1 SV=4	0.23999927	1
Q9H0W9	Ester hydrolase C11orf54 OS=Homo sapiens OX=9606 GN=C11orf54 PE=1 SV=1	-2.699525029	1

P52272	Heterogeneous nuclear ribonucleoprotein M OS=Homo sapiens OX=9606 GN=HNRNPM PE=1 SV=3	-0.238190768	1
P30043	Flavin reductase (NADPH) OS=Homo sapiens OX=9606 GN=BLVRB PE=1 SV=3	0.218277366	1
Q8TCA0	Leucine-rich repeat-containing protein 20 OS=Homo sapiens OX=9606 GN=LRRC20 PE=1 SV=1	-0.107285001	1
Q16610-4	Isoform 4 of Extracellular matrix protein 1 OS=Homo sapiens OX=9606 GN=ECM1	2.341690571	1
P46782	40S ribosomal protein S5 OS=Homo sapiens OX=9606 GN=RPS5 PE=1 SV=4	-2.212998324	1
O95169	NADH dehydrogenase [ubiquinone] 1 beta subcomplex subunit 8, mitochondrial OS=Homo sapiens OX=9606 GN=NDUFB8 PE=1 SV=1	0.234498201	1
O95139	NADH dehydrogenase [ubiquinone] 1 beta subcomplex subunit 6 OS=Homo sapiens OX=9606 GN=NDUFB6 PE=1 SV=3	-0.148725875	1
Q92945	Far upstream element-binding protein 2 OS=Homo sapiens OX=9606 GN=KHSRP PE=1 SV=4	2.703062304	1
P51149	Ras-related protein Rab-7a OS=Homo sapiens OX=9606 GN=RAB7A PE=1 SV=1	0.102760942	1
P52565	Rho GDP-dissociation inhibitor 1 OS=Homo sapiens OX=9606 GN=ARHGDIA PE=1 SV=3	-0.274615948	1
P09525	Annexin A4 OS=Homo sapiens OX=9606 GN=ANXA4 PE=1 SV=4	0.252229786	1
Q86YB7	Enoyl-CoA hydratase domain-containing protein 2, mitochondrial OS=Homo sapiens OX=9606 GN=ECHDC2 PE=1 SV=2	2.304233763	1
P01034	Cystatin-C OS=Homo sapiens OX=9606 GN=CST3 PE=1 SV=1	2.652328389	1
Q96DB5	Regulator of microtubule dynamics protein 1 OS=Homo sapiens OX=9606 GN=RMDN1 PE=1 SV=1	-0.233195794	1
P50990	T-complex protein 1 subunit theta OS=Homo sapiens OX=9606 GN=CCT8 PE=1 SV=4	0.095648029	1
P48147	Prolyl endopeptidase OS=Homo sapiens OX=9606 GN=PREP PE=1 SV=2	-0.073313267	1
P46781	40S ribosomal protein S9 OS=Homo sapiens OX=9606 GN=RPS9 PE=1 SV=3	-0.0917081	1
P55769	NHP2-like protein 1 OS=Homo sapiens OX=9606 GN=SNU13 PE=1 SV=3	-0.129560349	1
O95336	6-phosphogluconolactonase OS=Homo sapiens OX=9606 GN=PGLS PE=1 SV=2	1.699684993	1
P07358	Complement component C8 beta chain OS=Homo sapiens OX=9606 GN=C8B PE=1 SV=3	2.277839702	1
P05546	Heparin cofactor 2 OS=Homo sapiens OX=9606 GN=SERPIND1 PE=1 SV=3	0.018666862	1
Q07954	Prolow-density lipoprotein receptor-related protein 1 OS=Homo sapiens OX=9606 GN=LRP1 PE=1 SV=2	0.220886202	1

P51668	Ubiquitin-conjugating enzyme E2 D1 OS=Homo sapiens OX=9606 GN=UBE2D1 PE=1 SV=1	1.982421692	1
P13798	Acylamino-acid-releasing enzyme OS=Homo sapiens OX=9606 GN=APEH PE=1 SV=4	-0.120342524	1
P62917	60S ribosomal protein L8 OS=Homo sapiens OX=9606 GN=RPL8 PE=1 SV=2	0.135387991	1
Q12905	Interleukin enhancer-binding factor 2 OS=Homo sapiens OX=9606 GN=ILF2 PE=1 SV=2	-0.205322546	1
Q8NE71	ATP-binding cassette sub-family F member 1 OS=Homo sapiens OX=9606 GN=ABCF1 PE=1 SV=2	0.282617999	1
P13010	X-ray repair cross-complementing protein 5 OS=Homo sapiens OX=9606 GN=XRCC5 PE=1 SV=3	-0.443684913	1
O60271	C-Jun-amino-terminal kinase-interacting protein 4 OS=Homo sapiens OX=9606 GN=SPAG9 PE=1 SV=4	1.693415397	1
Q16853	Membrane primary amine oxidase OS=Homo sapiens OX=9606 GN=AOC3 PE=1 SV=3	-0.202221334	1
O75663	TIP41-like protein OS=Homo sapiens OX=9606 GN=TIPRL PE=1 SV=2	0.143629163	1
Q9Y3A3	MOB-like protein phocein OS=Homo sapiens OX=9606 GN=MOB4 PE=1 SV=1	-0.099417679	1
P10412	Histone H1.4 OS=Homo sapiens OX=9606 GN=H1-4 PE=1 SV=2	0.303524308	1
Q9Y696	Chloride intracellular channel protein 4 OS=Homo sapiens OX=9606 GN=CLIC4 PE=1 SV=4	2.49906213	1
Q13228-4	Isoform 4 of Methanethiol oxidase OS=Homo sapiens OX=9606 GN=SELENBP1	0.085860405	1
P35813-3	Isoform 3 of Protein phosphatase 1A OS=Homo sapiens OX=9606 GN=PPM1A	-1.447769445	1
Q07960	Rho GTPase-activating protein 1 OS=Homo sapiens OX=9606 GN=ARHGAP1 PE=1 SV=1	-0.486249657	1
Q96MG2	Junctional sarcoplasmic reticulum protein 1 OS=Homo sapiens OX=9606 GN=JSRP1 PE=1 SV=1	0.145794723	1
Q92947	Glutaryl-CoA dehydrogenase, mitochondrial OS=Homo sapiens OX=9606 GN=GCDH PE=1 SV=1	-1.278828739	1
Q9Y6N5	Sulfide:quinone oxidoreductase, mitochondrial OS=Homo sapiens OX=9606 GN=SQOR PE=1 SV=1	0.539862732	1
P35221-2	Isoform 2 of Catenin alpha-1 OS=Homo sapiens OX=9606 GN=CTNNA1	0.646751503	1
P09543	2',3'-cyclic-nucleotide 3'-phosphodiesterase OS=Homo sapiens OX=9606 GN=CNP PE=1 SV=2	-0.258093103	1
Q9Y285	Phenylalanine--tRNA ligase alpha subunit OS=Homo sapiens OX=9606 GN=FARSA PE=1 SV=3	-0.11530127	1

Q16629	Serine/arginine-rich splicing factor 7 OS=Homo sapiens OX=9606 GN=SRSF7 PE=1 SV=1	0.705600171	1
P50897	Palmitoyl-protein thioesterase 1 OS=Homo sapiens OX=9606 GN=PPT1 PE=1 SV=1	2.464118463	1
P62829	60S ribosomal protein L23 OS=Homo sapiens OX=9606 GN=RPL23 PE=1 SV=1	-1.749301312	1
P58546	Myotrophin OS=Homo sapiens OX=9606 GN=MTPN PE=1 SV=2	0.090713256	1
P10321	HLA class I histocompatibility antigen, C alpha chain OS=Homo sapiens OX=9606 GN=HLA-C PE=1 SV=3	0.547461722	1
Q9UHG3	Prenylcysteine oxidase 1 OS=Homo sapiens OX=9606 GN=PCYOX1 PE=1 SV=3	0.065565813	1
Q5SV97	PGC-1 and ERR-induced regulator in muscle protein 1 OS=Homo sapiens OX=9606 GN=PERM1 PE=2 SV=4	0.179325541	1
P20020-1	Isoform D of Plasma membrane calcium-transporting ATPase 1 OS=Homo sapiens OX=9606 GN=ATP2B1	0.619743292	1
P51398	28S ribosomal protein S29, mitochondrial OS=Homo sapiens OX=9606 GN=DAP3 PE=1 SV=1	0.628197107	1
Q9Y3U8	60S ribosomal protein L36 OS=Homo sapiens OX=9606 GN=RPL36 PE=1 SV=3	2.5075974	1
Q9C0E8-4	Isoform 4 of Endoplasmic reticulum junction formation protein lunapark OS=Homo sapiens OX=9606 GN=LNPK	-0.706571766	1
P47756	F-actin-capping protein subunit beta OS=Homo sapiens OX=9606 GN=CAPZB PE=1 SV=4	0.058419562	1
O00743-3	Isoform 3 of Serine/threonine-protein phosphatase 6 catalytic subunit OS=Homo sapiens OX=9606 GN=PPP6C	0.659678261	1
Q8NBF2	NHL repeat-containing protein 2 OS=Homo sapiens OX=9606 GN=NHLRC2 PE=1 SV=1	0.450760301	1
P34949	Mannose-6-phosphate isomerase OS=Homo sapiens OX=9606 GN=MPI PE=1 SV=2	0.037291042	1
P08708	40S ribosomal protein S17 OS=Homo sapiens OX=9606 GN=RPS17 PE=1 SV=2	0.601719995	1
Q92522	Histone H1x OS=Homo sapiens OX=9606 GN=H1FX PE=1 SV=1	2.96500337	1
P39656	Dolichyl-diphosphooligosaccharide--protein glycosyltransferase 48 kDa subunit OS=Homo sapiens OX=9606 GN=DDOST PE=1 SV=4	-0.112157702	1
Q16401	26S proteasome non-ATPase regulatory subunit 5 OS=Homo sapiens OX=9606 GN=PSMD5 PE=1 SV=3	0.735058286	1
P53634	Dipeptidyl peptidase 1 OS=Homo sapiens OX=9606 GN=CTSC PE=1 SV=2	1.19612207	1
Q9NP97	Dynein light chain roadblock-type 1 OS=Homo sapiens OX=9606 GN=DYNLRB1 PE=1 SV=3	-1.160249933	1

Q9UL25	Ras-related protein Rab-21 OS=Homo sapiens OX=9606 GN=RAB21 PE=1 SV=3	0.697970229	1
P78344	Eukaryotic translation initiation factor 4 gamma 2 OS=Homo sapiens OX=9606 GN=EIF4G2 PE=1 SV=1	0.908007906	1
Q04828	Aldo-keto reductase family 1 member C1 OS=Homo sapiens OX=9606 GN=AKR1C1 PE=1 SV=1	0.044037426	1
P16284	Platelet endothelial cell adhesion molecule OS=Homo sapiens OX=9606 GN=PECAM1 PE=1 SV=2	0.659667778	1
Q9P0V9-2	Isoform 2 of Septin-10 OS=Homo sapiens OX=9606 GN=SEPTIN10	1.109727846	1
Q96PU8	Protein quaking OS=Homo sapiens OX=9606 GN=QKI PE=1 SV=1	1.349785811	1
P61204	ADP-ribosylation factor 3 OS=Homo sapiens OX=9606 GN=ARF3 PE=1 SV=2	-0.038872888	1
Q16816-2	Isoform 2 of Phosphorylase b kinase gamma catalytic chain, skeletal muscle/heart isoform OS=Homo sapiens OX=9606 GN=PHKG1	-0.089062166	1
P0C0L5	Complement C4-B OS=Homo sapiens OX=9606 GN=C4B PE=1 SV=2	-1.235683795	1
Q15084-2	Isoform 2 of Protein disulfide-isomerase A6 OS=Homo sapiens OX=9606 GN=PDIA6	-0.049190504	1
Q9NZJ6	Ubiquinone biosynthesis O-methyltransferase, mitochondrial OS=Homo sapiens OX=9606 GN=COQ3 PE=1 SV=3	0.084262521	1
P48729-2	Isoform 2 of Casein kinase I isoform alpha OS=Homo sapiens OX=9606 GN=CSNK1A1	1.157695059	1
Q9UNH7	Sorting nexin-6 OS=Homo sapiens OX=9606 GN=SNX6 PE=1 SV=1	1.545608968	1
P35222	Catenin beta-1 OS=Homo sapiens OX=9606 GN=CTNNB1 PE=1 SV=1	0.982669056	1
P45985-2	Isoform 2 of Dual specificity mitogen-activated protein kinase kinase 4 OS=Homo sapiens OX=9606 GN=MAP2K4	1.10219117	1
Q86UT6	NLR family member X1 OS=Homo sapiens OX=9606 GN=NLRX1 PE=1 SV=1	-0.06867493	1
P14621	Acylphosphatase-2 OS=Homo sapiens OX=9606 GN=ACYP2 PE=1 SV=2	-0.027714692	1
Q8ND76	Cyclin-Y OS=Homo sapiens OX=9606 GN=CCNY PE=1 SV=2	1.180941155	1
P22087	rRNA 2'-O-methyltransferase fibrillarin OS=Homo sapiens OX=9606 GN=FBL PE=1 SV=2	-1.082176	1
Q6IBS0	Twinfilin-2 OS=Homo sapiens OX=9606 GN=TWF2 PE=1 SV=2	-0.045806952	1
Q9H1E5	Thioredoxin-related transmembrane protein 4 OS=Homo sapiens OX=9606 GN=TMX4 PE=1 SV=1	-0.710980682	1
P12236	ADP/ATP translocase 3 OS=Homo sapiens OX=9606 GN=SLC25A6 PE=1 SV=4	1.009370753	1
P10176	Cytochrome c oxidase subunit 8A, mitochondrial OS=Homo sapiens OX=9606 GN=COX8A PE=1 SV=2	0.014373577	1

Q14914	Prostaglandin reductase 1 OS=Homo sapiens OX=9606 GN=PTGR1 PE=1 SV=2	-1.329618371	1
P20929-3	Isoform 3 of Nebulin OS=Homo sapiens OX=9606 GN=NEB	-0.022758927	1
O95167	NADH dehydrogenase [ubiquinone] 1 alpha subcomplex subunit 3 OS=Homo sapiens OX=9606 GN=NDUFA3 PE=1 SV=1	-0.029070919	1
P13489	Ribonuclease inhibitor OS=Homo sapiens OX=9606 GN=RNH1 PE=1 SV=2	-0.006946597	1
Q562R1	Beta-actin-like protein 2 OS=Homo sapiens OX=9606 GN=ACTBL2 PE=1 SV=2	1.157902901	1
P23109	AMP deaminase 1 OS=Homo sapiens OX=9606 GN=AMPD1 PE=1 SV=2	0.008921726	1
P51608-2	Isoform B of Methyl-CpG-binding protein 2 OS=Homo sapiens OX=9606 GN=MECP2	1.205228892	1
P13671	Complement component C6 OS=Homo sapiens OX=9606 GN=C6 PE=1 SV=3	0.008623461	1
P08754	Guanine nucleotide-binding protein G(i) subunit alpha OS=Homo sapiens OX=9606 GN=GNAI3 PE=1 SV=3	-0.664248646	1
P09669	Cytochrome c oxidase subunit 6C OS=Homo sapiens OX=9606 GN=COX6C PE=1 SV=2	-0.01205155	1
Q14112	Nidogen-2 OS=Homo sapiens OX=9606 GN=NID2 PE=1 SV=3	0.003976976	1
Q14157-5	Isoform 5 of Ubiquitin-associated protein 2-like OS=Homo sapiens OX=9606 GN=UBAP2L	0.974574574	1
P62942	Peptidyl-prolyl cis-trans isomerase FKBP1A OS=Homo sapiens OX=9606 GN=FKBP1A PE=1 SV=2	-1.112731005	1
Q9Y277-2	Isoform 2 of Voltage-dependent anion-selective channel protein 3 OS=Homo sapiens OX=9606 GN=VDAC3	-3.919682021	1
Q15121-2	Isoform 2 of Astrocytic phosphoprotein PEA-15 OS=Homo sapiens OX=9606 GN=PEA15	1.105471475	1
Q13451	Peptidyl-prolyl cis-trans isomerase FKBP5 OS=Homo sapiens OX=9606 GN=FKBP5 PE=1 SV=2	-0.855747983	1
